# The chloroplast genome of black pepper (*Piper nigrum* L.) and its comparative analysis with related *Piper* species

**DOI:** 10.3389/fpls.2022.1095781

**Published:** 2023-01-12

**Authors:** Ambika Baldev Gaikwad, Tanvi Kaila, Avantika Maurya, Ratna Kumari, Parimalan Rangan, Dhammaprakash Pandhari Wankhede, K. V. Bhat

**Affiliations:** ^1^ Division of Genomic Resources, Indian Council of Agricultural Research (ICAR)-National Bureau of Plant Genetic Resources, New Delhi, India; ^2^ Indian Council of Agricultural Research (ICAR)-National Institute for Plant Biotechnology, New Delhi, India

**Keywords:** black pepper, chloroplast genome, gene content, gene order, phylogeny

## Abstract

*Piper nigrum*, also known as black pepper, is an economically and ecologically important crop of the genus *Piper*. It has been titled as the king of spices due to its wide consumption throughout the world. In the present investigation, the chloroplast genome of *P. nigrum* has been assembled from a whole genome sequence by integrating the short and long reads generated through Illumina and PacBio platforms, respectively. The chloroplast genome was observed to be 161,522 bp in size, having a quadripartite structure with a large single copy (LSC) region of 89,153 bp and a small single copy (SSC) region of 18,255 bp separated by a copy of inverted repeats (IRs), each 27,057 bp in length. Taking into consideration all the duplicated genes, a total of 131 genes were observed, which included 81 protein-coding genes, 37 tRNAs, 4 rRNAs, and 1 pseudogene. Individually, the LSC region consisted of 83 genes, the SSC region had 13 genes, and 18 genes were present in each IR region. Additionally, 216 SSRs were detected and 11 of these were validated through amplification in 12 species of *Piper*. The features of the chloroplast genome have been compared with those of the genus *Piper*. Our results provide useful insights into evolutionary and molecular studies of black pepper which will contribute to its further genetic improvement and breeding.

## Introduction

Chloroplasts are essential intracellular organelles having an independent genome with several genes responsible for the process of photosynthesis. Most higher plants harbor a single, circular, double-stranded chloroplast DNA with a quadripartite structure that includes a long single copy (LSC) region, a small single copy (SSC) region, and two copies of large inverted repeat (IRa and IRb) regions (Bock, 2007). Chloroplast genomes are highly conserved in terms of their size, gene content, and structure with a few exceptions (Dong et al., 2013; Daniell et al., 2016). According to the Organelle Genome Resource-NCBI (www.ncbi.nlm.nih.gov/genome/browse#!/organelles/), 4,557 chloroplast genomes of land plants are reported until December 2020.

Magnoliidae is one of the largest and early-diverging Angiosperm clade representing four orders—Canellales, Piperales, Laurales, and Magnoliales—that comprise around 10,000 species in 20 families (Massoni et al., 2015; Chase et al., 2016; Sinn et al., 2018). So far, 384 chloroplast genomes belonging to Magnoliidae have been submitted to NCBI-Organelle Genome Resource of which 48 belong to Piperales and only 15 to the genus *Piper*. Piperaceae is considered to be the most diverse group of magnoliids with around more than 4,300 species. The genus *Piper* possesses more than 2,000 species (Divya et al., 2015) including the economically important crop black pepper (*Piper nigrum*).

Black pepper has been titled as the “king of spices” due to its wide consumption throughout the world for inducing a pungent flavor in culinary dishes. The Western Ghats region of India is known to be the center of origin and diversity for this species with around 30 countries growing it commercially, mainly India, Indonesia, Malaysia, China, Thailand, and Brazil (Kubitzki and Rohwer, 1993; Ravindran, 2000). The medicinal value of secondary metabolites produced by *P. nigrum* reveals its role in digestive disorders, hepatoprotection, antithyroidism, anti-inflammation, antidepressant, immunomodulator, antispasmodic, antioxidant, and many more (Ahmad et al., 2012).

In the present investigation, we report on the complete sequence of the *P. nigrum* chloroplast genome. Detailed characterization of the black pepper chloroplast including size, gene content, structure repeats, and GC content and comparative genomics analysis of the chloroplast genome provide insights into functional genomics (Li et al., 2019). Moreover, phylogenetic analysis of 58 genes and 37 taxa consisting of 34 angiosperms and 3 gymnosperms provides insight into the phylogenetic position of *P. nigrum* in accordance with other magnoliids, monocots, and eudicots.

## Material and methods

### DNA extraction and next-generation sequencing

Leaves of black pepper landrace ‘Thottumuriyan’ were used to extract high-quality genomic DNA that was assessed for its quality and quantity using Qubit. Two paired-end libraries were prepared from genomic DNA using TruSeq DNA PCR-Free LT sample Prep Kit (Illumina, San Diego, USA) as per standard protocol and quantified using Bioanalyzer (Agilent Technologies, USA) and qPCR (Kapa Biosystems, Wilmington, MA, USA) sequenced on HiSeq1000 and MiSeq (Illumina, San Diego, CA, USA) using 2 × 100 and 2 × 150 bases paired-end chemistry, respectively. Pacific Biosciences (PacBio) SMRT bell sequencing libraries were prepared for long read generation and were sequenced on PacBio Sequel. A total of 82.74- and 14.65-GB data were generated through Illumina sequencing and PacBio, respectively.

### Chloroplast genome assembly and annotation

The FastQC software (Andrews, 2010) was used to check the quality of the sequenced reads, and then the Trimmomatic software (Bolger et al., 2014) was used to trim and filter the low-quality reads. Filtered data were mapped to the reference chloroplast genome using CLC Genomics workbench 9.0. Mapped reads of PacBio sequence data were extracted and assembled using the Organelle PBA software (Soorni et al., 2017) which was polished through the Pilon software (Walker et al., 2014) using Illumina sequence data.

For the preliminary annotation of the chloroplast genome, the D online program GeSeq (Tillich et al., 2017) offered by MPI-MP Chlorobox was used and the output was curated. The tRNA genes were identified by the ARAGORN v1.2.38 tRNA annotator (Laslett and Canback, 2004), which was available at the GeSeq server. The OGDraw (Greiner et al., 2019) online server of Chlorobox was used to construct a gene map of the chloroplast genome with default settings and checked manually. The black pepper chloroplast genome sequence was assembled to 161,522 bp and submitted to NCBI, GenBank accession No. MK883818 (**Supplementary File 1**).

### Repeat structure and comparative genome analysis

Codon usage and GC content were analyzed using CodonW and Molecular Evolutionary Genetics Analysis (MEGA 7.0) (Kumar et al., 2016), respectively. To investigate the size and location of repeat sequences comprising forward, reverse, palindromic, and complement repeats, REPuter online server was used with a minimal size of 30 bp, >90% identity, and hamming distance of 3 between two repeat copies. Also, Tandem Repeats Finder (Benson, 1999) was used to extract the tandem repeats present in the chloroplast genome of *P. nigrum* and the other reported species of *Piper*. Simple sequence repeats (SSRs) were detected using the MISA software (Beier et al., 2017) with parameters of eight, four, four, three, three, and three repeat units for mono-, di-, tri-, tetra-, penta-, and hexanucleotide SSRs, respectively. mVISTA (Mayor et al., 2000) was used to perform whole genome sequence alignment of *P. nigrum* along with other members of Piperaceae comprising *Piper kadsura*, *Piper laetispicum*, *Piper cenocladum*, and *Piper auritum.*

### Validation of SSRs

A set of 11 primer pairs were synthesized using the software Primer3 (Untergasser et al., 2012) and validated in 21 accessions of *Piper* representing 12 different species (*Piper nigrum*, *Piper longum*, *Piper arboreum*, *Piper argyrophyllum*, *Piper attenuatum*, *Piper betel*, *Piper chaba*, *Piper hymenophyllum*, *Piper trichostachyon*, *Piper wallichi*, *Piper columbrinum*, and *Piper sarmentosum*). Genomic DNA was isolated from the leaf using the cTAB DNA extraction method. The PCR reaction consisted of 1× PCR buffer, 2.5 mM of MgCl_2_, 1 µM of primer, 0.2 mM of each dNTP, 1 U of *Taq* DNA polymerase (NEB), and 15 ng of template DNA in a total volume of 20 µl and cycled at 95°C for 5 min followed by 35 cycles of denaturation at 95°C for 1 min, annealing at 55°C for 1 min and extension at 72°C for 1 min followed by a final extension at 72°C for 10 min. The amplification products were resolved on a QIAxcel multicapillary system using QIAxcel High Resolution Kit 1200 (QIAGEN, No. 929002, New Delhi,Qiagen India Pvt. Ltd.) 50-800 bp v2.0 QX DNA size marker (QIAGEN, No. 929561, New Delhi,Qiagen India Pvt. Ltd.) and 15 bp/1,000 bp Qx alignment marker (QIAGEN No. 929521, New Delhi,Qiagen India Pvt. Ltd.) with the high-resolution run method OM700. The allelic sizes of each sample were calculated in the form of gel profiles and peaks using the QIAxcel ScreenGel software (QIAGEN, v1.5).

### Synonymous substitution

The numbers of synonymous substitutions per synonymous sites (*K*s) and non-synonymous substitutions per non-synonymous sites (*K*a) and functional polymorphism within magnoliids were calculated using the DnaSP software (Rozas et al., 2017).

### Phylogenetic *a*nalysis

Phylogenetic analysis was performed with 58 genes common to all 37 complete chloroplast genomes downloaded from NCBI (**Supplementary File 2**). The 37 complete chloroplast genomes used in the study are those of species representing 31 orders of the phylum Tracheophyta. These include 3 orders from the gymnosperms; those of Amborella, Austrobaileyales, and Chloranthales; 4 orders of the magnoliids; the Alismatid, Lilioid, and Commelinid monocots; and 17 orders of the eudicots. Firstly, each gene from all 37 taxa was aligned individually using ClustalW 2.0 (Thompson et al., 1994). On an online server, gBlocks was used to create conserved blocks with default settings, eliminating the poorly aligned regions. Maximum likelihood analysis was performed with PhyML using the subtree pruning and regrafting (SPR) topological moves with Smart Model Selection (SMS) of the substitution model based on Akaike information criterion (AIC) and 100 bootstrapping.

## Results and discussion

### Features of the *Piper nigrum* chloroplast genome

**Figure 1:**
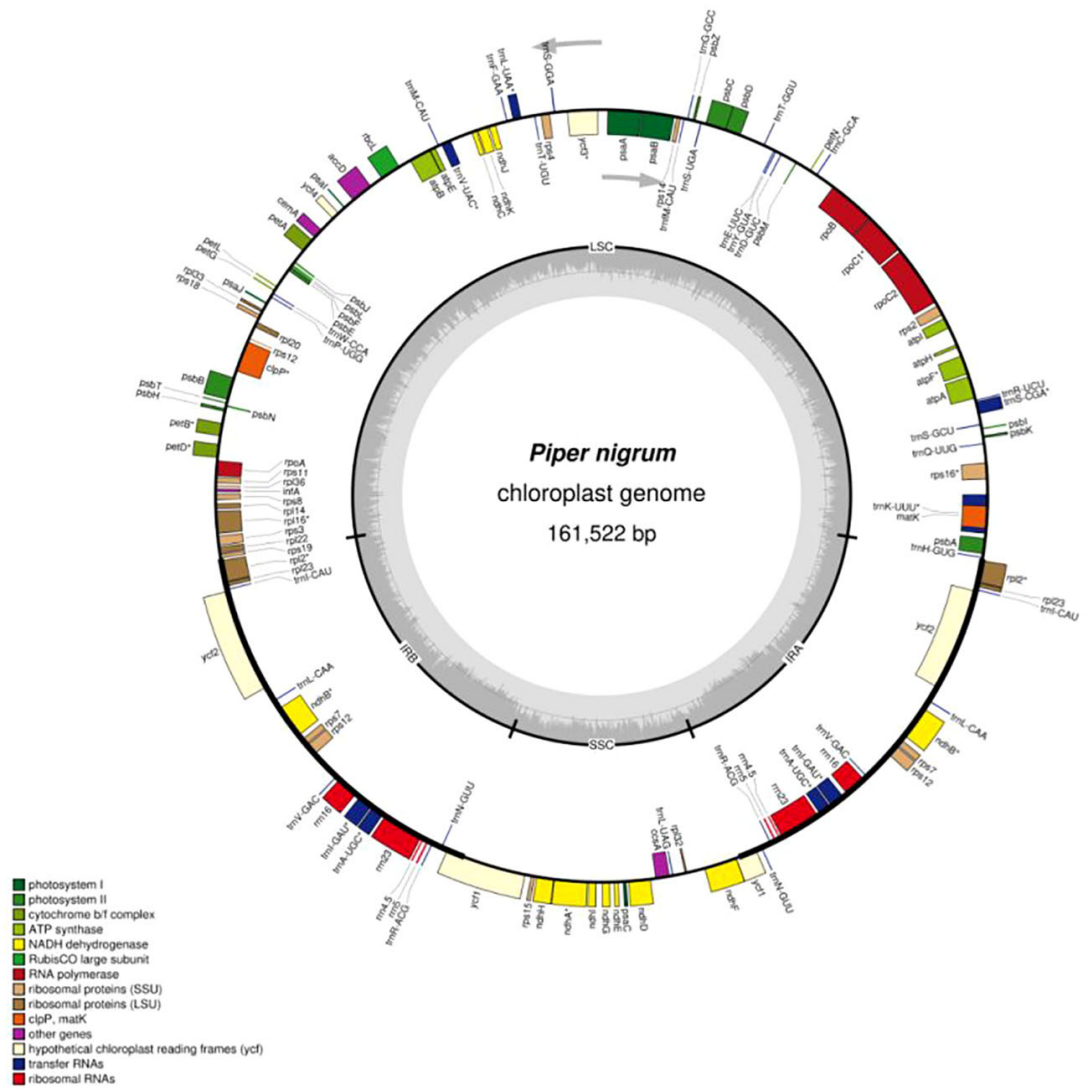
Circular map of the *Piper nigrum* chloroplast genome.

With the increased use of next-generation sequencing, the number of reported angiosperm chloroplast genomes has increased. Nonetheless, only 15 chloroplast genomes belonging to the genus *Piper* have been sequenced. Recently, the complete chloroplast genome sequences of *Piper hancei* (Zhang et al., 2021) and *P. sarmentosum* (Geng et al., 2022) have been reported. A comparative analysis of eight *Piper* species with *P. nigrum* has been also reported (Li et al., 2022). In the present investigation, the chloroplast genome of *P. nigrum* was sequenced and analyzed. The complete assembled chloroplast genome of *P. nigrum* is a circular model with the length of 161,522 bp, having a typical quadripartite structure comprising an LSC region of 89,153 bp and an SSC region of 18,255 bp separated by a copy of inverted repeats (IRs) each 27,057 bp in length (**Figure 1**). *Piper nigrum* differs slightly from other reported *Piper* species in terms of the total length of the chloroplast genome. It is smaller than *P. laetispicum* (161,721 bp), but larger than *P. kadsura* (161,486 bp), *P. cenocladum* (160,624 bp), *P. auritum* (160,624 bp), and *P. hancei* (161,476 bp) in terms of length.

The *P. nigrum* chloroplast genome was observed to have 131 genes, which include 81 protein-coding genes, 37 tRNAs, 4 rRNAs, and 1 pseudogene (**Table 1**). On considering each region individually, it was observed that the LSC consists of 83 genes, the SSC consists of 13 genes, and 18 genes were present in the IR region. The duplication of tRNA genes in the LSC region has been observed in various angiosperms (Tsudzuki et al., 1994; Kaila et al., 2016; Kaila et al., 2017). However, as with other reported Piperaceae chloroplast genomes (Cai et al., 2006), the tRNA gene was not observed to be duplicated in *P. nigrum*. The gene *rps12* was observed to be trans-spliced with one end present in the LSC region and another duplicated end present in the IR region. Trans-splicing of the *rps12* gene has also been reported in other chloroplast genomes like *Pinus taeda*, *P. kadsura*, and *Fagus crenata* (Lee et al., 2016; Asaf et al., 2018; Worth et al., 2019). In consonance with the previous studies, the *trnK-UUU* gene was observed to be harboring the largest intron (2,548 bp) which includes the *matK* gene.

**Table 1:** List of genes in the chloroplast genome of *Piper nigrum*.

Category of genes	Group of genes	Gene name	Total number
Photosynthesis	Photosystem I	*psaA*, *psaB*, *psaC*, *psaI*, *psaJ*	5
	Photosystem II	*psbA*, *psbB*, *psbC*, *psbD*, *psbE*, *psbF*, *psbH*, *psbI*, *psbJ*, *psbK*, *psbL*, *psbM*, *psbN*, *psbT*, *psbZ*	15
	Cytochrome b/f complex	*petA*, *petB^a^*, *petD* ^a^, *petG*, *petL*, *petN*	6
	ATP synthase	*atpA*, *atpB*, *atpE*, *atpF^a^*, *atpH*, *atpI*	6
	NADH dehydrogenase	*ndhA* ^a^, *ndhB* ^a^ (×2) 1, *ndhC*, *ndhD*, *ndhE*, *ndhF*, *ndhG*, *ndhH*, ndhI, *ndhJ*, *ndhK*	12
	RubisCO large subunit	*rbcL* 1	1
Self-replication	RNA polymerase	*rpoA*, *rpoB*, *rpoC1* ^a^, *rpoC2*	4
	Ribosomal proteins	*rps2*, *rps3*, *rps4*, rps7 (×2), *rps8*, *rps11*, *rps12* ^b^ (×2), *rps14*, *rps15*, *rps16* ^a^, *rps18*, *rps19*, *rpl2* ^a^ (×2), *rpl14*, *rpl16* ^a^, *rpl20*, *rpl22*, *rpl23* (×2), *rpl32*, *rpl33*, *rpl36*	25
	Ribosomal RNAs	*rrn4*.5 (×2), *rrn5* (×2), *rrn16* (×2), *rrn23* (×2) 8 (4)	8
	Transfer RNAs	37 tRNAs (6 contain an intron, 7 in the IRs)	37
Other genes	Hypothetical proteins	*ycf1*, *ycf2* (×2), *ycf3* ^b^, *ycf4*	5
	Translational initiation factor	*infA*	1
	Maturase	*matK*	1
	Protease	*clpP* ^b^	1
	Envelope membrane protein	*cemA*	1
	Subunit of Acetyl-CoA-Carboxylase	*accD*	1
	C-type cytochrome synthesis gene	*ccsA*	1
	Putative pseudogene	*ycf1*	1
Total genes			131

a One intron.

b Two introns.

Pseudogenes are similar to functional genes except that they do not produce functional proteins. One reason for this may be a disrupted open reading frame due to the presence of an internal stop codon(s). In *P. nigrum*, the *ycf1* gene was identified to be the only pseudogene, similar to *P. kadsura* (Lee et al., 2016) and *P. laetispicum* (Wang et al., 2018), while *Cinnamomum camphora* and *Persea americana* are reported to have three (*rpl23*, *ycf1*, and *ycf2*) and two (*ycf1* and *ycf2*) pseudogenes, respectively (Li et al., 2019; Liu et al., 2019).

The average AT content of the *P. nigrum* chloroplast genome was observed to be 61.7% which is similar to other reported *Piper* species (Cai et al., 2006; Lee et al., 2016; Wang et al., 2018; Li et al., 2022). The single copy regions LSC (63.2%) and SSC (67.9%) possess higher AT content than the repeat regions (57.1%) (**Table 2**). The lower AT content of the IR region can be associated with the presence of rRNA genes, which possesses a decreased number of AT nucleotides, thereby contributing to genome stabilization and sequence complexity. Also, the AT content for the CDS region (protein-coding region) was observed to be 60.9% (**Table 2**). Coding regions have higher GC content and therefore lower AT content than non-coding regions. While if we consider the different regions of the chloroplast genome, the IR regions have the lowest AT content and the SSC regions have the highest AT content. This is due to the presence of NADH genes present in the SSC region that are reported to have the lowest GC content as compared with any class of genes (Cai et al., 2006).

**Table 2:** Nucleotide composition in different regions of the chloroplast genome of *Piper nigrum*.

Regions	Position	T(U)	C	A	G	Length
IRa	–	28.8	22.1	28.3	20.8	27,057
IRb	–	28.3	20.8	28.8	22.1	27,057
LSC	–	32.2	18.8	31.0	18.0	89,153
SSC	–	33.9	15.3	34.0	16.9	18,255
Total	–	31.2	19.3	30.5	19.0	161,522
CDS region	–	30.9	18.2	30.3	20.7	79,099
	Position 1	32	16.5	30.2	21.0	26,366
	Position 2	26	19.4	30.6	23.6	26,366
	Position 3	34	18.6	29.9	17.4	26,366

The chloroplast genome of *P. nigrum* consists of 18 intron-containing genes, viz., *ycf3*, *rpoC1*, *atpF*, *rps16*, *rpl2*, *ndhB*, *ndhA*, *rpl16*, *petB*, *petD*, *clpP*, *rps12*, and 6 tRNA genes (**Table 3**). Three genes, viz., *ycf3*, *rps12*, and *clpP*, consist of two introns, while the rest have one intron. Similar observations have been made for other plants belonging to the Magnoliid clade like *P. cenocladum* and *Drimys granadensis* (Cai et al., 2006) which harbor 18 intron-containing genes with three genes spanning 2 introns. The recently reported chloroplast genome of *P. hancei* also harbors 18 intron-containing genes, 16 of which contain one intron and 2 genes (*clpP* and *ycf3*) possess two introns (Zhang et al., 2021). In contrast, a recent analysis of eight *Piper* chloroplast genomes reveal 14 intron-containing genes of which 10 protein-coding genes and 2 tRNA genes had a single intron and 2 genes had two introns (Li et al., 2022). Different from these results, the chloroplast genome of *P. sarmentosum* was reported to have 21 intron-containing genes, consisting of 8 tRNA genes and 13 protein-coding genes of which *ycf3* and *clpP* possess two introns each and the others contain only one intron (Geng et al., 2022). Similarly, *C. camphora* (Li et al., 2019) which also belongs to the magnoliids consists of 17 intron-containing genes which include 3 genes having two introns. In the case of the *P. nigrum* chloroplast genome, the protein-coding region accounts for 49.6% of the genome, while the tRNA and rRNA regions comprise 1.7% and 5.5% of the whole genome, respectively (**Table 4**). Therefore, the remaining genome consists of introns, intergenic regions, and pseudogenes. Within the Piperaceae, no change is observed in the number of protein-coding genes, rRNA, and GC percentage. Except for *P. auritum* which is reported to have 36 tRNAs and no pseudogene (NC_034697, unpublished), *P. cenocladum* (Cai et al., 2006) that contains no pseudogene, and *P. hancei* (Zhang et al., 2021) that consists of 36 tRNAs, the rest of the *Piper* species contain 37 tRNAs and 1 pseudogene (*ycf1*), respectively.

**Table 3:** Length of exons and introns in chloroplast genes of *Piper nigrum*.

Gene	Location	Exon1	Intron1	Exon2	Intron2	Exon3
*trnk-UUU*	LSC	37	2,548	35		
*trnS-CGA*	LSC	31	736	62		
*rps16*	LSC	40	823	254		
*atpF*	LSC	145	752	410		
*rpoC1*	LSC	432	771	1,620		
*ycf3*	LSC	124	823	230	726	153
*trnL-UAA*	LSC	35	489	50		
*trnV-UAC*	LSC	39	599	34		
*rps12*	LSC	114	–	31	537	137
*clpP*	LSC	71	882	293	639	245
*petB*	LSC					
*petD*	LSC					
*rpl16*	LSC					
*rpl2*	IR	385	665	431		
*ndhB*	IR	777	700	756		
*trnI-GAU*	IR	37	947	35		
*trnA-UGC*	IR	38	822	35		
*ndhA*	SSC	552	1,129	540		

**Table 4:** Gene content comparison of *Piper nigrum* chloroplast with reported *Piper* species and other magnoliids.

Species	Total length of the cp genome (bp)	GC%	Protein-coding genes	rRNA-coding genes	tRNA-coding genes	Total number of genes	Number of pseudogenes
*Calycanthus chinensis*	153,596	39.3	84	8	37	129	0
*Cinnamomum camphora*	152,570	39.1	84	8	36	130	2
*Drimys granadensis*	160,604	38.8	85	8	44	137	0
*Liriodendron tulipifera*	159,886	39.2	84	8	37	129	0
*Persea americana*	152,723	39.1	81	8	36	127	2
*Piper auritum*	159,909	38.3	85	8	36	129	0
*Piper cenocladum*	160,624	38.3	85	8	37	130	0
*Piper kadsura*	161,486	38.3	85	8	37	131	1
*Piper laetispicum*	161,721	38.3	85	8	37	131	1
*Piper nigrum*	161,522	38.3	85	8	37	131	1

Considering the other members of the Magnoliid clade, *Persea* was found to have 81 protein-coding genes, whereas *Cinnamomum* and *Calycanthus* of Laurales (Chen et al., 2018) and *Liriodendron* of Magnoliales (Park et al., 2019) consist of 84 protein-coding genes. *Cinnamomum* and *Persea* comprise 36 tRNA, whereas *Calycanthus* and *Liriodendron* have 37 tRNAs. *Drimys* of the order Canellales consists of 85 protein-coding genes and 44 tRNAs which is the maximum number of tRNAs present within magnoliids (**Table 4**). In comparison to other magnoliids, *ycf1* is present as a pseudogene at the SSC/IRa junction in *Piper* species, whereas in *Drimys*, *Calycanthus*, and *Liriodendron*, the 3' fragment of *ycf1* is present at the IRb/SSC junction and a complete copy of *ycf1* is present at the SSC/IRa junction.

### Codon usage

**Figure 2:**
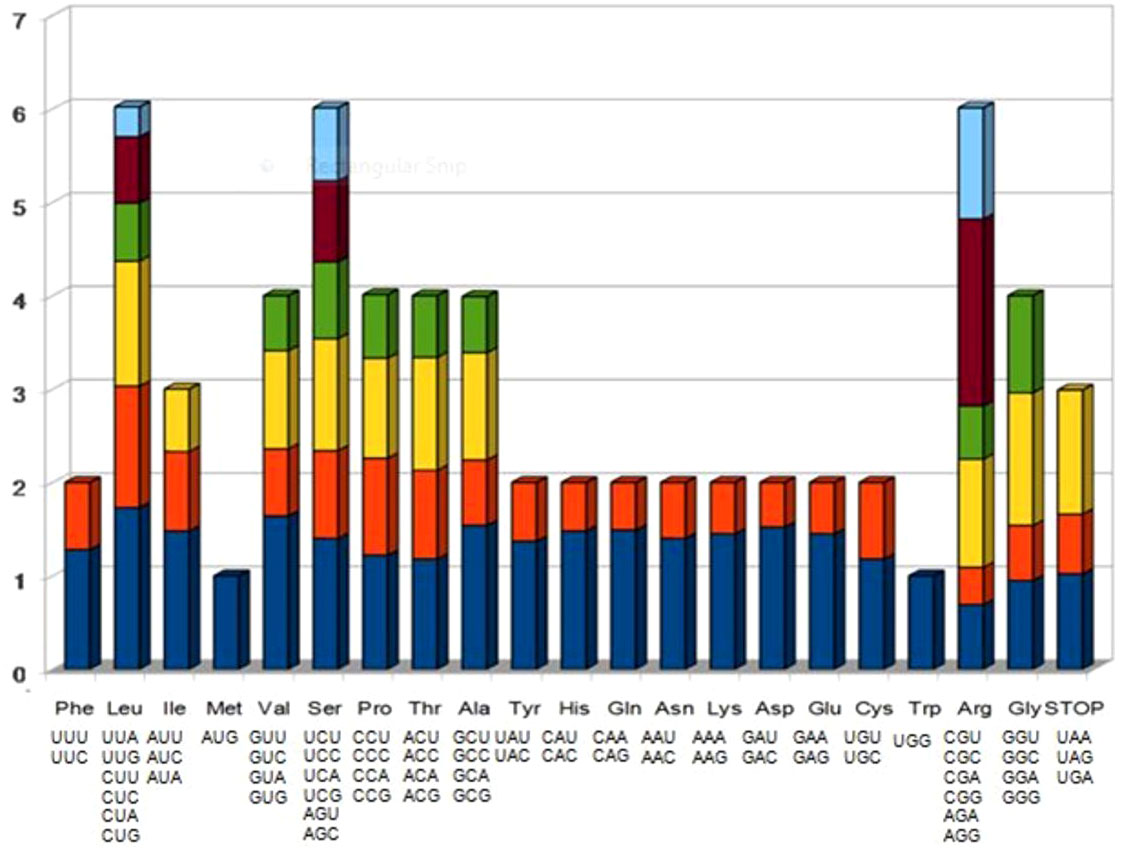
Codon usage of the CDS region of the chloroplast genome of *Piper nigrum*.

Codon usage for the *P. nigrum* chloroplast genome was calculated with the CodonW software. It was observed that 61 codons encoded for 20 amino acids and 3 stop codons. A total of 26,366 codons represented 85 protein-coding genes, 79,099 nucleotides in length. Among the amino acids, serine was the most (2,687 codons) and tryptophan was the least (550 codons) encoded amino acids (**Figure 2**). Codon usage bias is a phenomenon in which certain codons are preferred repeatedly over the others. This usage varies for different genomes and genes. Biasness was also observed in the presentation of a nucleotide at the third codon position. The AT content varies according to codon position with the highest at the third codon position, followed by the first and second codon positions (Morton, 2003). The above observation is also supported by relative synonymous codon usage (RSCU) values. It was found that 40.7% of codons ended with A and T, whereas 23.4% of codons ended with C and G. It is known that organellar proteins are primarily encoded by codons having A or U at the third codon position. Similar results have been reported for various plastid genomes in the past (Cai et al., 2006; Asaf et al., 2018).

### Gene content

The chloroplast genomes have evolved over a period of time. On comparing the algal and embryophyte plastomes, it is shown that a number of genes (*tufA*, *ftsH*, *odpB*, and *rpl5*) have been lost or transferred to the nucleus and a few genes, namely, *matK*, *ycf1*, and *ycf2*, have been gained by the abovementioned plastomes (Turmel et al., 2006). There also exist differences in the gene content among the angiosperms as genes like *infA*, *accD*, and *rpl22* have been reported to be lost in legumes (Doyle et al., 1995), and on the other hand, *accD*, *ycf1*, and *ycf2* have been lost in *Poaceae* (Guisinger et al., 2010). Complete loss or pseudogenization of *ndh* genes has been shown in heterotrophic plants in the past. Nonetheless, events showing *ndh* gene loss have also been observed in Pinaceae, gnetophytes, and autotrophic orchids (Wakasugi et al., 1994; Braukmann et al., 2009; Kim et al., 2015).

Among the Mesangiospermae, magnoliids are the third largest group after the eudicots and monocots. Except for a few differences in gene content, the magnoliids are identical to tobacco and many unarranged angiosperms in terms of gene order. The *ACRS* gene, which has been reported in a number of plastid genomes as the *ycf68* gene, was identified in *Calycanthus* based on its similarity to a mitochondrial ACR sensitivity toxin gene of *Citrus jambhiri* (Ohtani et al., 2002). Due to the presence of internal stop codons, it has been reported as a pseudogene in a number of plastomes (Curci et al., 2015; Yan et al., 2015). However, it was not annotated in the *P. nigrum* chloroplast genome and hence observed to be absent from the plastid genome. Similar observations were made for *D. granatenis* and *P. cenocladum* (Cai et al., 2006), belonging to the magnoliid clade. Likewise, another gene, namely, *ycf15*, was not annotated in the *P. nigrum* plastid genome and hence was found to be absent. The *ycf15* has also been reported to be a non-functional protein-coding gene (Shi et al., 2013) and hence a pseudogene due to the presence of internal stop codons (Yan et al., 2015; Kaila et al., 2017). It is found to be present in the *Calycanthus* plastome but absent in the *D. granatenis* and *P. cenocladum* plastid genomes (Cai et al., 2006). If we consider the phenomenon of nuclear substitution, then one such gene which is well-studied is *rps16*. The *rps16* gene has been found to be present as a pseudogene in some plastid genomes (Roy et al., 2010; Kaila et al., 2016) or has been completely lost from the plastid genome due to non-functional protein sequence or incorrect splicing of intron due to mutation at the 5' or 3' splicing site. Like in certain legumes, the loss of *rps16* from the chloroplast genome and its replacement by nuclear copy have been reported (Ueda et al., 2008). However, in *P. nigrum*, its copy has been retained in the chloroplast genome similar also to *P. cenocladum*, *P. laetispicum*, and *P. auritum.* As mentioned above, the chloroplast genome of *Piper* is similar to tobacco, and it has been shown that *rps16* is indispensable for tobacco plastome function (Fleischmann et al., 2011). A better regulation of plastome *rps16* gene in certain conditions or the non-presence of chloroplast-targeting sequence in nuclear-encoded peptide has been proposed as the reasons behind the retention of plastid *rps16* gene in some plants (Keller et al., 2017). Some differences in gene content may also lead to the expansion of the IR region, which is a common phenomenon occurring in the plastid genome. One such example is duplication and inclusion of the *trnH* gene in the IR region of the *D. granatenis* chloroplast genome, hence leading to the expansion of the IR region (Cai et al., 2006). Such duplication of the *trnH* gene has not been observed in any other plant belonging to magnoliids or basal angiosperms. Similarly, such duplication was not observed in the *P. nigrum* plastid genome.

### Repeat structures

**Figure 3:**
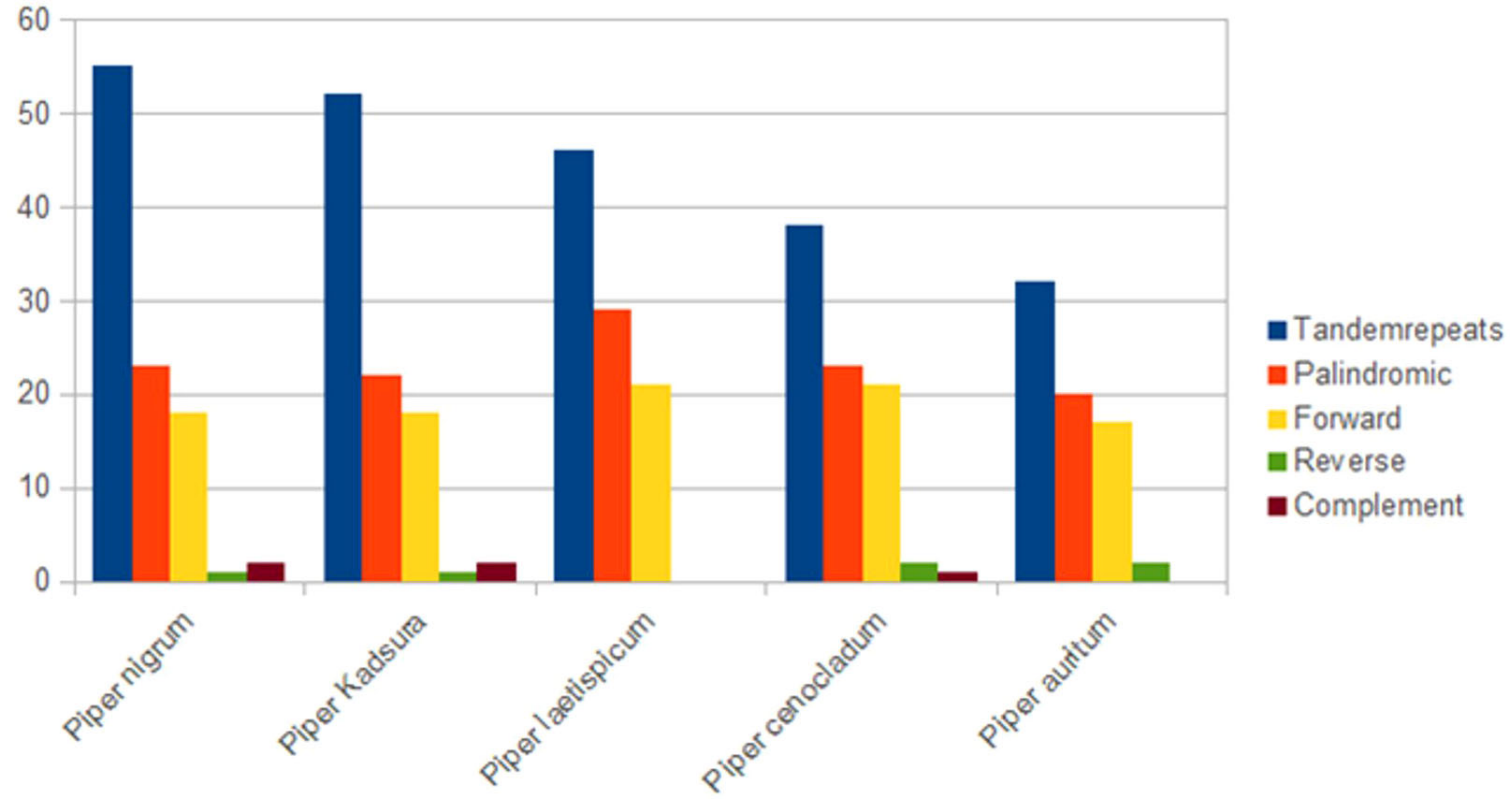
Repeat sequences in five *Piper* species plastid genomes calculated using REPuter and Tandem Repeats Finder.

Repeat structure analysis through the REPuter software (Kurtz et al., 2001) revealed that *P. nigrum* comprises 23 palindromic, 18 forward, 2 complement, and 1 reverse repeat along with 55 tandem repeats that were predicted using the Tandem Repeats Finder. The length of the largest forward and palindromic repeat was 52 bp each. Among the tandem repeats, 64% of the repeats were present in the intergenic region, 28% in the coding region, and 8% in the intronic region. Palindromic repeats were observed to be present in majority within all the reported species of *Piper* (**Figure 3**). Reverse and complement repeats were absent in *P. laetispicum*, whereas no complement repeat was present in *P. auritum. Piper auritum* had a minimum number of tandem repeats (Wang et al., 2018; NC_034697). Likewise, palindromic and forward repeats were the most abundant repeat types in other plant types like *P. americana* (Liu et al., 2019) and *Aristolochia contorta* (Zhou et al., 2017). These repeats have been reported to be responsible for chloroplast genome rearrangement, gene duplication, and gene expression (Do et al., 2014; Vieira et al., 2014). It is due to their role in genome rearrangement that they have proved to be helpful in phylogenetic studies (Cavalier-Smith, 2002). Improper recombination and slipped strand mispairing of these repeat units result in plastome rearrangement and sequence variation among different plastid genomes. These repeats are also responsible for substitutions and indels in the chloroplast genome (Yi et al., 2013). They have also proved to be an informational source for developing markers and therefore play an important role in population and phylogenetic studies (Nie et al., 2012).

### Simple sequence repeats

**Figure 4:**
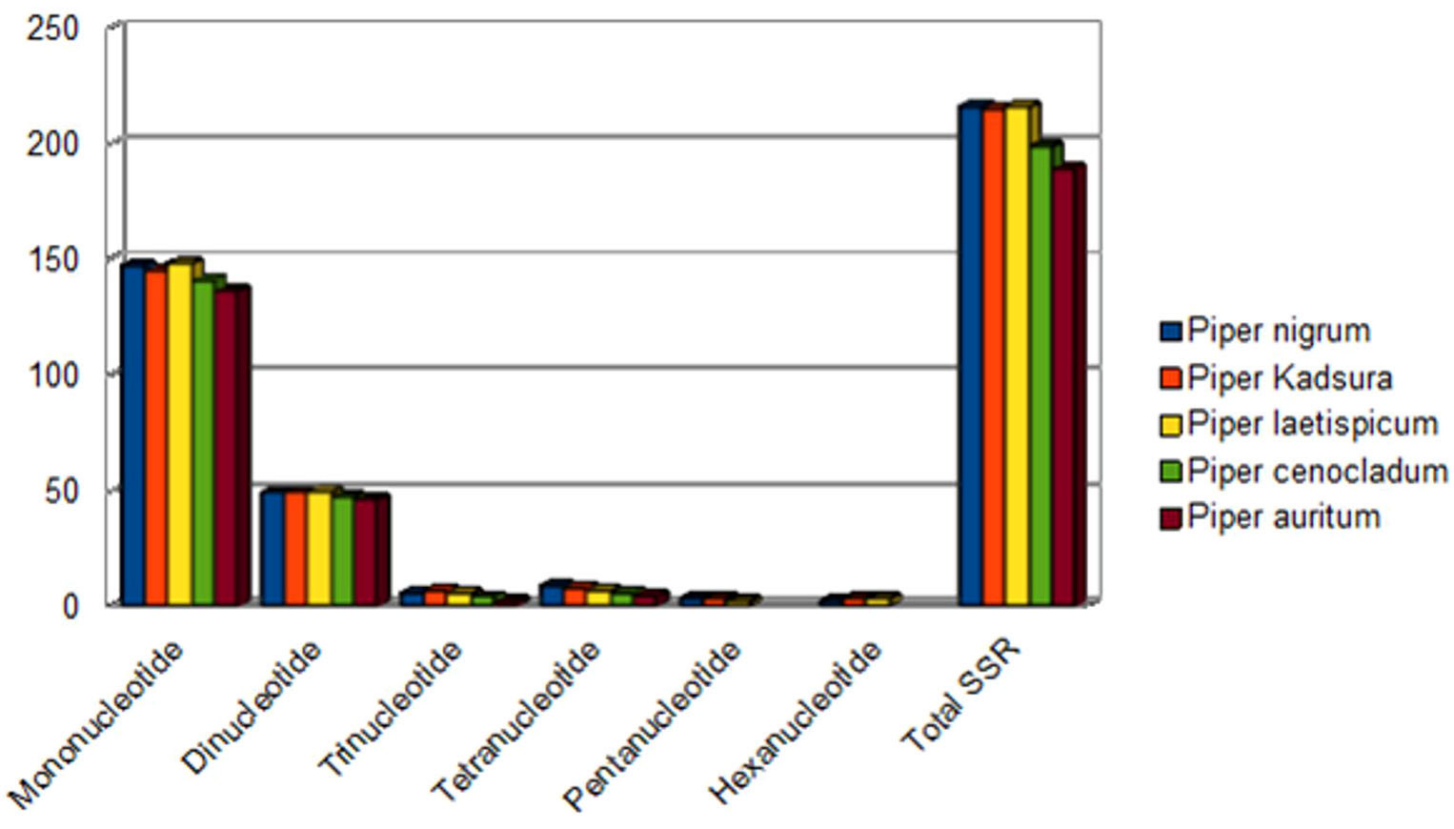
Simple sequence repeats (SSRs) in plastid genomes of five *Piper* species.

**Figure 5:**
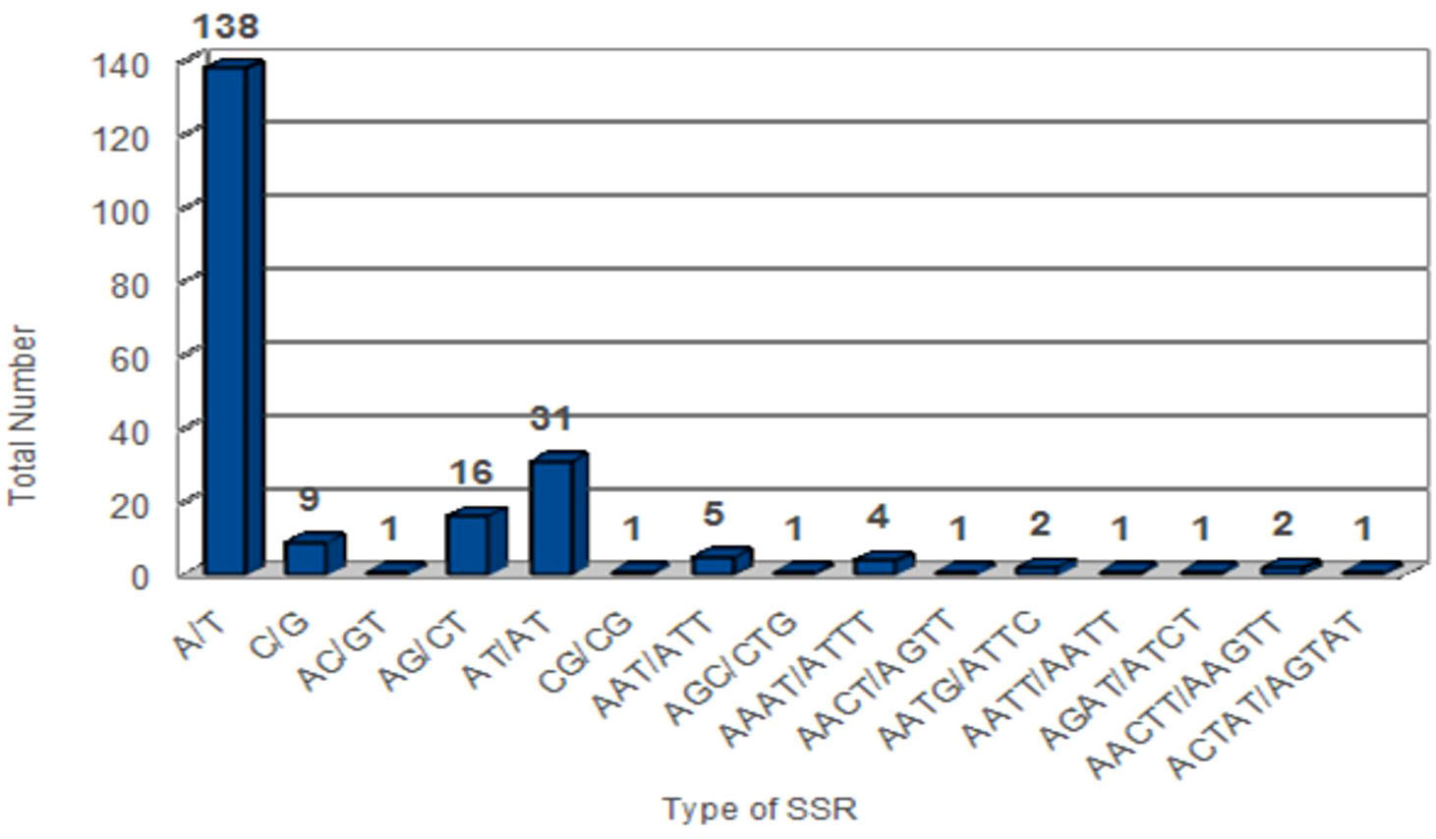
Distribution of SSRs based on their repeat type in *Piper nigrum.*.

A group of tandem repeats comprising DNA sequences of 1-6 nucleotides long are called microsatellites or SSRs. They are present in large numbers in both coding and non-coding regions. They are highly reproducible, abundant in number, polymorphic, and uniparentally inherited. Variation in SSRs at a specific locus contributes to its use as a molecular marker (Olmstead and Palmer, 1994). Therefore, they are used in evolutionary studies, phylogenetic relationships, and plant population genetics. A total of 216 SSRs were found using the MISA perl script [http://pgrc.ipk-gatersleben.de/misa/] (Beier et al., 2017). The number of SSRs extracted was similar to those reported in other *Piper* species like *P. kadsura* (215 SSRs), *P. laetispicum* (216 SSRs), *P. cenocladum* (198 SSRs), and *P. auritum* (189 SSRs). Among the abovementioned 216 SSRs, 65% were present in the LSC region, 13% in the SSC region, and 11% in the IR regions. It was also observed that 32% of the SSRs were present in the coding region and 13% in the non-coding region, and the maximum SSRs (53%) were present in the intergenic region. Thus, the maximum number of SSRs is present in the non-coding region than in the coding region. Similar observations have also been made for *P. taeda* (Asaf et al., 2018) and *Cyamopsis tetragonoloba* (Kaila et al., 2017). If we look individually at the coding region, then it is observed that the *ycf1* gene had a maximum number of repeats. Previous studies have also reported the *ycf1* gene as the most variable locus, and similar observations have been made for *Glycine* species, *Cynara cardunculus*, and *Camellia* species (Huang et al., 2014; Curci et al., 2015; Ozyigit et al., 2015). Among the 216 SSRs found, the most abundant were mononucleotide repeats (147), followed by dinucleotide (49), trinucleotide (6), tetranucleotide (9), pentanucleotide (3), and hexanucleotide repeats (2). On comparison, it was found that all the repeat types were present in different *Piper* species except pentanucleotides and hexanucleotides, which were absent in *P. cenocladum* and *P. auritum* (**Figure 4**). In *P. nigrum*, the A/T repeats were found to be in majority with 138 in number out of 147 mononucleotide repeats present. The AT/AT was the most common among the dinucleotide repeats and five out of six trinucleotide repeats were AAT/ATT (**Figure 5**). Such A/T richness of mononucleotides has been observed earlier by Wheeler et al. (2014). This biasness in base composition and the presence of polyadenine (A) or polythymine (T) rich repeats have been reported earlier in species like *C. tetragonoloba* (Kaila et al., 2017), *P. taeda* (Asaf et al., 2018), *P. bournei* (Li et al., 2017), and *Aristolochia debilis* (Zhou et al., 2017).

**Figure 6:**
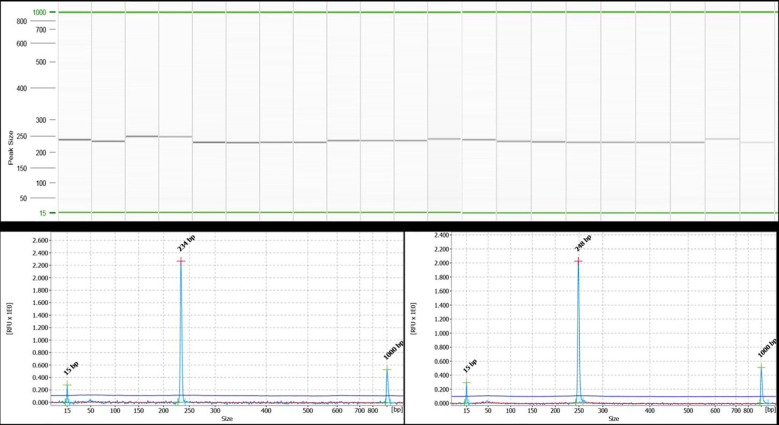
QIAxcel gel image of PCR amplification using the black pepper chloroplast SSR marker BPC5 in 21 accessions of 12 different species of black pepper as captured on the QIAxcel ScreenGel software. Lanes 1 and 2: *Piper longum*; lanes 3 and 4: *Piper arboreum*; lanes 5 and 6: *Piper argyrophylum*; lane 7: *Piper attenuatum*; lanes 8–10: *Piper betel*; lanes 11 and 12: *Piper chaba*; lane 13: *Piper hymenophyllum*; lanes 14 and 15: *Piper trichostachyon*; lane 16: *Piper wallichi*; lanes 17–19: *Piper nigrum*; lane 20: *Piper columbrinum*; lane 21: *Piper sarmentosum*. The lane marked as “M” is DNA molecular weight standard 50-800 bp v2.0 Qx DNA size marker. A representative electropherogram showing allele size of 234 bp for lane 2 and 248 bp for lane 3 has been shown below the gel image.

Wet lab validation at 11 SSR loci across 12 *Piper* species (including *P. nigrum*) resulted in the amplification of 60 alleles (**Figure 6**). The 11 loci included di-, tri-, tetra-, and pentanucleotide repeat core motifs (**Table 5**). The number of alleles detected ranged from 4 to 11, with the maximum number of alleles detected at the locus at BPC8 with a pentanucleotide core repeat. Amplification at this locus indicates the presence of pentanucleotide repeats in *P. longum*, *P. arboreuum*, *P. argyrophyllum*, *P. attenuatum*, *P. betel*, *P. chaba*, *P. trichostachyon*, *P. sarmentosum*, *P. columbrinum*, *P. wallichi*, and *P. hymenophyllum*. The high degree of polymorphism detected across the 12 *Piper* species allows for the identification of hypervariable regions informative for DNA barcoding and is a source for further evolutionary and phylogenetic studies in *Piper* species.

**Table 5:** Details of 11 chloroplast SSR primers validated in 12 *Piper* species (21 accessions).

Primer ID	Primer sequence	Repeat motif	No. of alleles	Annealing temp. (°C)	Allele size (bp)
BPC1	f-CGAATACACCAGCTACGCCT r-AGTTTCCGTCTGGGTATGCG	(CAG)4	4	55	220
BPC2	f-CTGGGCGCGAGGACTAAAAA r-TGAGTGGACTAAAGCGGCAG	(GA)4	7	55	280
BPC3	f-CCCCCAACTCAACTAGGTCG r-GGTATTTCGGCGAGTCCTCA	(AT)4	5	55	271
BPC4	f-TCCAAATCAATCCTGCGGGT r-GGCTTGGCCGAAGAACTTCA	(TC)4	5	55	178
BPC5	f-TTGGCCCACTCTTCATCGAC r-GTGATGGAGTTTTGTTTCCAGGA	(TG)5	7	55	231
BPC6	f-TTCCACTCACCCCATGGTTG r-TCAGATTCAAAACGGCTTGCT	(AATG)3	5	55	280
BPC7	f-GTCCTGCGAAGGGAAGGAAA r-ACTTTGGGATTGCTGAATTGCA	(AG)4	4	55	272
BPC8	f-GAACTTTTTGGTTTGGGGGCT r-GTGCTGATCGTTACAGGCCT	(ATAGT)3	11	55	278
BPC9	f-TGAGTTGGGCGCTTTAACCA r-ACTCCAAATTCGGGGGTCAA	(GA)4	4	55	273
BPC10	f-ACGACAGGCAAACCTCTAGA r-GCACCAATCCCGCTTTCTTG	(TTTA)3	4	55	26
BPC11	f-CGCCAGACAAAAGTCAGAACA r-ATGCGGTTAAACCAGCTGTG	(CT)4	4	55	210

### Comparison of IR boundaries

**Figure 7:**
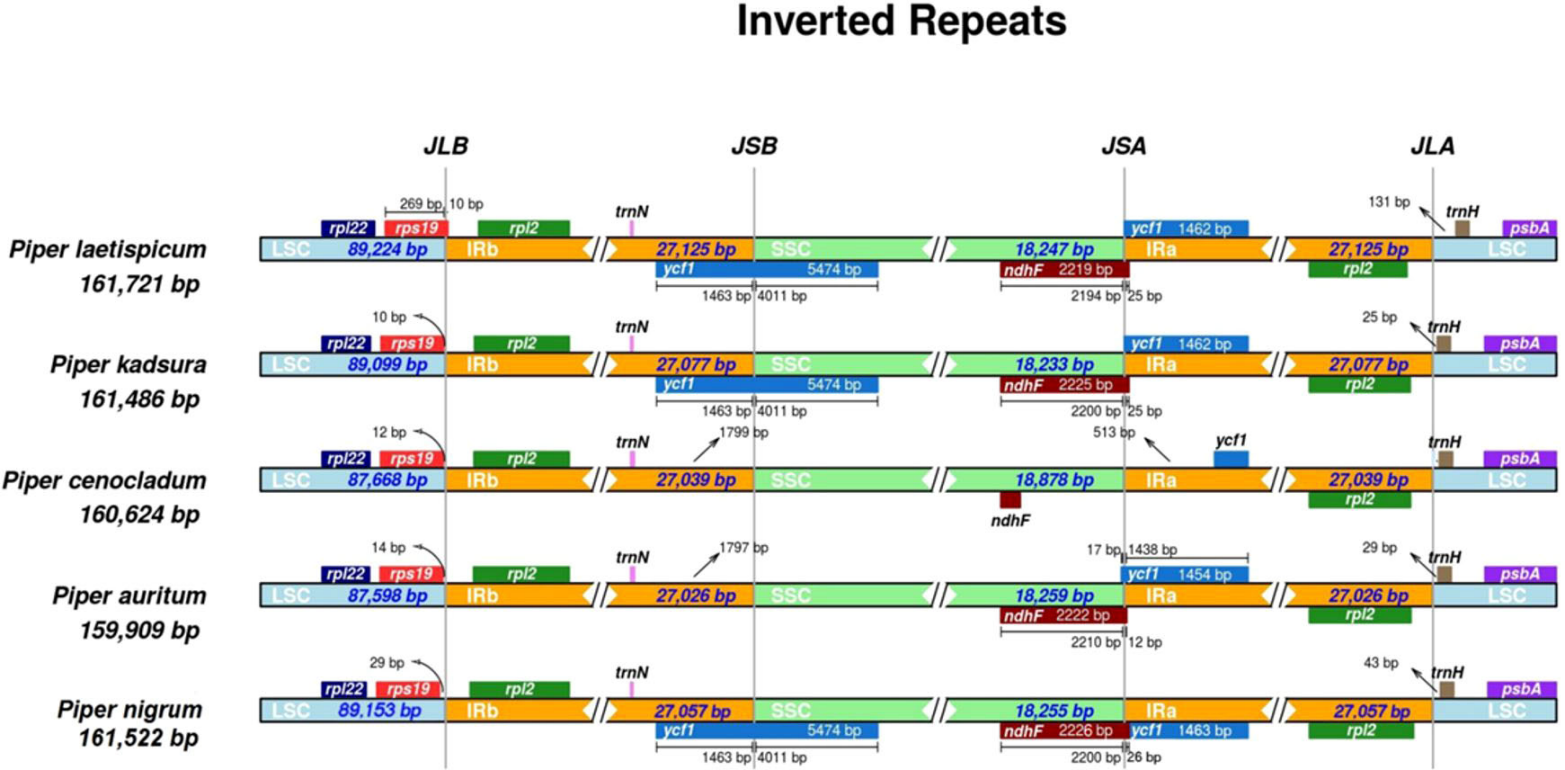
Contraction and expansion at junctions of four regions (LSC, SSC, IRa, IRb) of the plastid genome of five *Piper* species.

The IR regions provide stability to the chloroplast genome by exhibiting intramolecular recombination between the two IR copies (Palmer et al., 1987; Palmer, 1991). The expansion and contraction of IRs lead to the conversion of single-copy genes to duplicate copies and vice versa, respectively, thereby leading to variation in the plastome size among the angiosperms (Sabir et al., 2014). The chloroplast genomes of all *Piper* species are highly conserved, but expansion and contraction at the IR/SSC boundary regions leads to variation in the size of the genomes (Goulding et al., 1996). Here, we have compared the IR/SSC boundaries of *P. nigrum* with four other *Piper* species, namely, *P. kadsura*, *P. laetispicum*, *P. cenocladum*, and *P. auritum.* It was observed that 10 completely duplicated genes were present in the IR region of the *P. nigrum* chloroplast genome. In **Figure 7**, it can be seen that an intergenic region of 1,106 bp is present between *ndhF* and *ycf1* genes at the SSC/IRa junction in *P. cenocladum*, whereas *ndhF* and *ycf1* genes are overlapping at the SSC/Ira junction in other *Piper* species. The size of an overlapping fragment of the *ndhF* gene varies in different *Piper* species, hence contributing to fluctuating IR boundaries among different plastomes. The *ycf1* gene (5,474 bp) is present at the IRb/SSC junction in *P. nigrum*, *P. kadsura*, and *P. laetispicum*, whereas it is absent in *P. cenocladum* and *P. auritum.* A fragment of the *ycf1* gene of different sizes is present as a pseudogene at the SSC/IRa junction in all the species. The IR region of *P. nigrum* (27,057 bp) was observed to be larger than *P. kadusra* (24,657 bp), *P. laetispicum* (26,133 bp), and *P. cenocladum* (27,039 bp).

### Gene order

**Figure 8:**
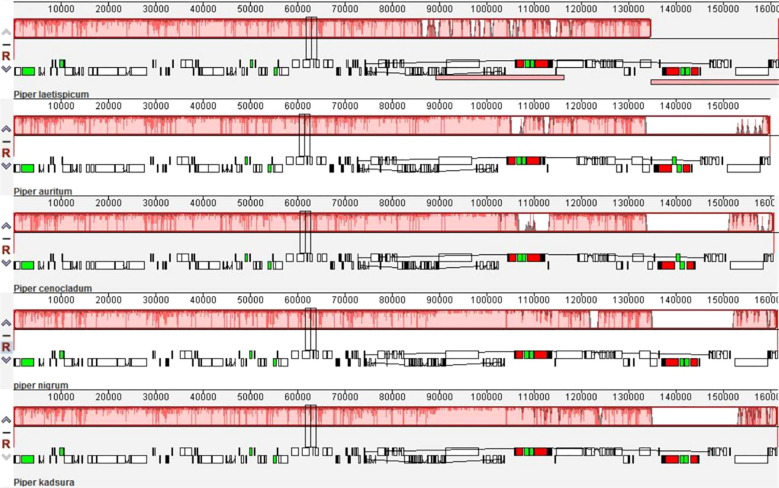
Gene order comparison of the chloroplast genome within Piperaceae.

The plastomes of five *Piper* species, viz., *P. laetispicum* (Wang et al., 2018), *P. auritum* (NC_034697), *P. cenocladum* (Cai et al., 2006), *P. nigrum*, and *P. kadsura* (Lee et al., 2016) were compared with the help of the Mauve software (Darling et al., 2004). In the comparison of different *Piper* species, no change was observed in the gene order within Piperaceae except for the absence of the *ycf1* gene at the IRb/SSC junction in *P. cenocladum* and *P. auritum* (**Figure 8**). On analysis, it was found that the sequence of the *ycf1* gene was present at the IRb/SSC junction in both the *P. cenocladum* and *P. auritum* plastid genomes, but it is not being annotated. Therefore, it can be inferred that a significant level of synteny can be seen between the plastid genome of different *Piper* species. One difference that can be accounted for is the inclusion of the *ycf1* gene in the IR region, which varies among different plastid genomes and therefore contributes to variation in IR size and ultimately to the chloroplast genome size.

### Phylogenetic analysis

**Figure 9:**
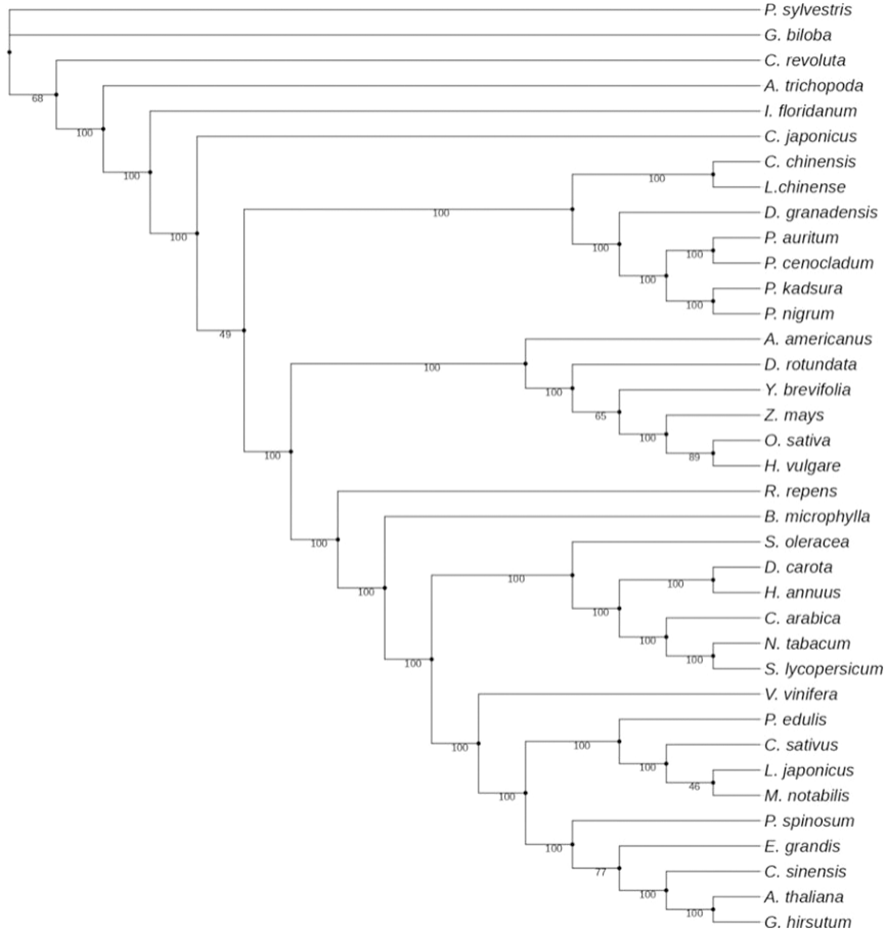
Phylogenetic tree of 37 different species based on 58 protein-coding genes.

Chloroplast genomes have proved to be useful in evolutionary, phylogenetic, and molecular systematic studies. They are an important resource for exploring evolutionary histories within and among different species. Various studies have compared intergenic spacers, protein-coding genes, and complete chloroplast genome sequences and have, therefore, contributed to our understanding of evolutionary relationships among different angiosperms and gymnosperms. In the present investigation, the phylogenetic data set included 58 protein-coding genes (38,262 bp after the removal of poorly aligned regions) from 37 taxa, comprising 34 angiosperms and 3 gymnosperms. Maximum likelihood analysis resulted in a single tree with higher bootstrap values (**Figure 9**). Twenty-eight out of 35 nodes showed 100% bootstrap value. The 100% bootstrap value strongly supports the monophyly of magnoliids. Magnoliids with 100% support value form two distinct clades: one with Canellales and Piperales and the other Magnoliales and Laurales. Piperales with four species of *Piper* got further divided into two clades with 100% bootstrap value. *Piper nigrum* is positioned with *P. kadsura* in one clade, and *P. auritum* and *P. cenocladum* were observed to be more similar. Our findings were in agreement with the results reported by Wang et al. (2018), where *P. nigrum* and *P. kadsura* were placed in one clade.

## Conclusion

In the present investigation, the chloroplast genome of *P. nigrum* was sequenced by integrating two platforms: Illumina and PacBio. The sequenced chloroplast genome was assembled using the Organelle PBA software and the Pilon software. The chloroplast genome having 161,522 bp length showed a typical quadripartite structure with 81 protein-coding genes, 37 tRNAs, 4 rRNAs, and 1 pseudogene. Codon usage analysis revealed that serine was the most coded amino acid, and 61 codons were present for coding 20 amino acids. The gene *rps16*, which has been observed to be lost from some of the plastid genomes, is present in the *P. nigrum* chloroplast genome similar also to other *Piper* species. Repeat analysis was also done, and there were 23 palindromic, 18 forward, 2 complement, and 1 reverse repeat along with 55 tandem repeats that were predicted. Of the 216 SSRs identified, the majority were present in the intergenic region. Phylogenetic analysis done with 58 protein-coding genes for 37 taxa, comprising 34 angiosperms and 3 gymnosperms, revealed the presence of *P. nigrum* and *P. kadsura* in one clade. This study has laid the foundation for future evolutionary and molecular studies on Piperales species.

## Data availability statement

The datasets presented in this study can be found in online repositories. The names of the repository/repositories and accession number(s) can be found in the article/**Supplementary Material**.

## Author contributions

Conceptualization and funding acquisition: AG and KB. Investigation and data analysis: AG, TK, AM, and RK. Manuscript preparation: AG, TK, PR and DW. All authors contributed to the article and approved the submitted version.
