# Supplementary material for: The chloroplast genome of black pepper (Piper nigrum L.) and its comparative analysis with related Piper species
Source: Front Plant Sci. 2023 Jan 12;13:1095781. doi: 10.3389/fpls.2022.1095781 (PMC9878596; doi:10.3389/fpls.2022.1095781)
Supplement: Supplementary file 1 [file Table_1.doc]

**Supplementary file1** *Piper nigrum* chloroplast genome sequence

>piper_nigrum_chloroplast

NCBI GenBank accession number MK883818

CAAGTCGTTATGTTTTGTTGGTTATGGGCGAACGACGGGAATTGAACCCGCGCGTGGTGG

ATTCACAATCCACTGCCTTGATCCACTTGGTTACATCCGCCCCTTAAATCTAGGTTTCCT

GTATTCTAGTAATAATAATATACTATACCTAAACGATTAAAAAAAATTATGCCCGCGTCG

ATTAAATGTAAATAAAACACTAGGAAATTCGGGGGAGCAATACCAAAACCTTGAAAAACA

AGAAATTGGGTATTGCTCCTTCAACAACTCCTATACATTAACATAAGGAGGAAGCATTAT

CCATTTGTAGATGGAGCTTCAACAGCAGCCAAGTCTAGAGGGAAGTTGTGTGCATTACGC

TCATGCATAACTTCCATACCAAGGTTTGCACGGTTAATGATATCAGCCCAAGTGTTAATA

ACACGACCTTGACTGTCAACTACGGATTGATTAAAGTTGAATCCATTTAGGTTGAAAGCC

ATGGTGCTGATACCTAAAGCAGTGAACCAAATACCCACTACAGGCCAAGCAGCTAAAAAG

AAATGTAAGGAGCGAGAATTGTTGAAACTAGCATATTGGAAGATCAATCGGCCAAAATAA

CCATGAGCAGCAACGATATTATAAGTTTCTTCCTCTTGACCGAATCTGTAACCTTCATTA

GCAGATTCATTTTCTGTGGTTTCCCTGATCAAACTAGAGGTTACCAAGGAACCATGCATA

GCACTGAATAGGGAGCCGCCGAATACACCAGCTACGCCTAGCATGTGAAATGGATGCATA

AGGATGTTGTGCTCAGCCTGGAATACAATCATGAAATTGAAAGTACCAGAAATTCCTAAA

GGCATACCATCAGAGAAGCTTCCTTGACCAATAGGGTAAATCAAGAAAACAGCAGCAGCA

GCTGCAACAGGAGCTGAATATGCAACAGCAATCCAAGGGCGCATACCCAGACGGAAACTA

AGTTCCCATTCACGACCCATGTAACAAGCTACACCAAGCAAGAAGTGTAGAACAATTAGC

TCATAAGGACCACCATTGTATAGCCATTCATCAACCGATGCTGCTTCCCAGATCGGGTAA

AAATGCAAACCGATAGCTGCAGAAGTAGGAATAATAGCGCCAGAGATAATATTGTTTCCA

TAAAGTAGGGAACCGGAAACAGGTTCACGAATACCATCAATATCCACTGGAGGAGCAGCA

ATGAAGGCGATAATCCATAGAGAAGTTGCGGTCAATAAAGTAGGGATCATCAAAACACCA

AACCATCCAATATAAAGACGGTTTTCAGTGCTAGTAATCCAGTTACAGAAGCGGCCCCAT

AGGCTTGTGCTTTCGCGTCTCTCTAAAATTGCAGTCATGGTAAGATCTTGGTTTATTTAA

TTTAATCATCAGGAACTCCCAAGCGCACGGATTATCTATAAATCCATATAGATAATTGAA

TGGAAGGCTTGTTATTCAACAGTATAACATGCCCGACATATCTGTGTCAACCAATATCAA

CACCATATTTAGATCCATATATCTATATGGATCAGCATTCTTTGTGAATTTAATTCTGTG

AGTAAAAAAAGAAGAAATGTTATACAAATTGATTTTACACAATAAAATAAAAGAAAGGAA

AAGAAATATCTATTTATTTTTCGCATTTCCGTACGGAAATGGGTTGCCCGGGACTCGAAC

CCGGAACTAGTCGGACGGAGTAGAAAAATTCCTGCCCTTGTTACAATAGTCGAAGGAATC

CCTCCCCAAACCGTGCTTGCATTTTTCATTGCACACGGCTTTACCTATGTATACATCAAA

AACCCATCCCCTAGATGAGTTTCAAGACACTTGAATAATCAATGAATTCAAGCACTACTA

CTGCACCGTATCAACATTTCAGAATGAAAATGAAAAAAAAAAAAAAGATAATACTAATTT

TCTTATTTTCTTACTTCATTTGAGTATTCCTATTAAATTAATGGAGTCTTATGACTTAAT

GGAGTCTTATGATCAATCATTCATGATTGGTCAAGTCATTTGTATGGGTAATATCCAAAT

ACCAAATCCGTTCTTTATCTTTATTTGACCTATACGAAGTAGGGTAGGATCTTGGGAAGA

AATATGAAAGAACTTGTTCCTCTTCGGCAAGGAATTCCTTCAAAAATCCCGAACCTAATT

TTTTCAGAAAGGTGCGTACTGTACTTTTATGTTTACGAGCCAAAGTTCTAGCACAAGAAA

GTCGAAGTATATACTTGATTCGATACAAAATTTGTTTTTTTGAACATCCACTGTAATAAT

GAGAAAGATTTCTGCATACGCGCCCAAATCGTTCCATAATATCAGAATCTGACAAATCCG

CCCAAATCGCCTTACTAGTGGGATGCCCTGATAGATTACAAAATTTTTCTTTTGACAATG

AATCAATGAGAGAAATAATTGGGACTATGGTTTCGAACTTCTTAACGGAAGTCTCCATTA

GAAATGAATTCTCTAGCATTTGACTTTTTACCACCGAAATATTTCTTCGTACACTTGAAA

CATAGCCCATGATGTGGTAGGTACGATGATCTAATTGGTTTATATGGATCCTGTGCAGTT

GAGACCACAAGTGAAAATTACATTGCCATAAATAGATAAAGTGATATTTCCATTTTTTCA

TCAAAAGATCAGTTCCCTTTGAAATCAAAAGGGATTTTTCCCGATATCTAACATAATGCA

TGAAAGAATCTTTGAACAACCGCAAAGCCTTCTGAAAATCATTACAAAGCACTACTACAA

GATGTTCTATTTTCCCATAGAAATGGACTCGTTCAAGAGTGGCTTCCCAGGATATTGATC

GTAAATGAGAAGATTGTTTACGGATAAAAAGGAATACGGATTCCCATTCATATACATGAG

AATTATATATGAATAAGGATAATCTTTCATTTTCTTTTTTTGAAAAAGAAAAGCATTTTT

TTTTGCTAATGAGACTGCCCGAATGACAATACTCGTATAAAAAGATTCGCAATAAATGCA

AAGAGGGGACGTCTTGTATCCAGTAACGAAGGGTTTGAACTAAAATTTCTGGATGGATAG

GGTAGGGTATTAATACATCCGATATATGATATAAATAGAATATATTGTCCTCAAAAAAGG

AAAATATTGAGTGGATAGATCGTAAATTATGAGATTTTGCTATTTCTCTTTTTTTTTCTA

GGCAAGACACTAATTGGAAGGAGAATGGAATTTCCACAATAATTGAAAAAGCCTCCGATA

TCATTTTCGAATGCAAATTGTTTTTTCGCCCCAACAATTGATTTTGTTGAAAATCATTTT

CATTACAAGAAATAAGAAAATGATTCTGTTTATGCATTCGAATAATTAAACGTTTCACAA

TTAGTGAACTAGATTTATCGCCATAACCTAAATTTTCTATGGATTCGTAAGGAATCGGTC

TATTTAAACCATGATCATGGCCGAGTGCGTAGATATATTCCTGAAAAAGAAGTGGATACA

GGAAGTGCTTTTGCTCAGATCTAAATGTTTCTAAATATCCTTTTAATTTTTCCATTTGAA

ATGACCAAAAAGTGTGGAAGGTTTCTTAGATTAACAAATGATACATAGTGCGATACGGTC

AAAACGGGGTATCCAATAAGAAAGGAATACCTCGAAGATAAGTAGCCAATCCACGGATCT

TCCCTTTTCTTCTATCCCACCTTTCGTTCCACTTACAGGAAGTGGTTAGAAATCCTTTAT

TTTTGCAATCCTATCGCTCTTTTGATTTTGGAATTTATTTTTTTCTCAGTATACCGTTTC

TTCTACACATTGCTTTTCCCATAAGAATCTAAAAAATGAGAGAATAATGAATAATTAGGA

TTCAAAAAAAGGGAATCGATGATTCACTCGCAGGAGAACCCCCCCCTTTTCCGCATCAGA

CACTAATATATTTTTAACCTCTAATTAGACCGGGCAATTATTCGAATTAAGAGAAAAAGC

TCGTTACTTCGGGTTTCCCTATAATTGGAGCTTTAGAGCTCTATCCATTTATTGACTCAA

CCCAATGATGAATTGATTTGATCCCCTTCCAAGAATCAAAACAAGATTTTGTAATGATCC

GGTCTAGTGAGGATTAAGAATGGGGTATTCTTAGAATTCTCCATTGAAACGACATGCTGT

TTTTTCCATTCATCCCCTTCAGGATCAGTCGTGGTCTTACAAACTTTACTAATAGTATGT

ACAAATACGTTGCTTCATCCAAATGTGTAAAAGATCATAGTCGCACTTAAAAGCCGAGTA

CTCTACCGTTGAGTTAGCAACCCATATATGTATATGTTAAGTAAATAGGATGTATAAATA

CGATCGTAATATAATAATATATTTTATATATATTTTAAAAATATATGATAAGGAAATCCC

CGACCTACCTAACAAAACTAAAACTAGTAAATAGTGAAAAATCTAAAGAAATCATTTAAG

AATCTATCAGGAAAATCAAAAAGAAACAAAAACGAACAGATCAGACTAAAAAACGGATTG

TAGTGCAGGGATCCATAGAAAAAGGTACTCTACTAACACTATAAAATAAAAAAATAAAAC

ACTATAAAAAAATAAAAATAAAATATAAAAAAATAAAATATAAAAAGGTATATAAATGGG

CGGAACCCTATCCAATTTTTCATGAATTTTTTCCATTCAACTAAAAACGAAAAAGAGACT

TCTTTTGTTCTAGTGAGTTTTGTGAATCCACCATTTTCGTGAAGTGAAGTGTGAAGTGAA

TAAACGAAGTGTTTGATCGTGGAGAAATCGATCGATTTCTCCACGATCAAATTATATTTG

ATCGATACACCATTGTCAATATGAATTGAATGTTGAAAAAAAAATAATAAATAAAAAGAA

AATGAATAAGGATAATATAATAATGAATAAAACTATTCAATTAATGAAAAAACGAATTTC

AAAGAATTCCATTTTTGTTGGATTGGCACTACATAGATAAGAAACGAAGTACGGATAGGG

GAATAAATTAAGGAAAAGAAGGATAAATCTAGAATTTTTCCAATATAGGTAAGTACAATA

AGTAAGATTGACTCTTTACCTGTTAAAATGAAATCCAATAGTAAAAACCCCTTCATGGTA

ATACCTACACCTCATAGATCCAGTTATTTCATCTATTCACTTGATCACCTTTACCGTCTC

ATATCAATACAATAAGATTTTTAAAATTATAAAATATACATACGATCTTAGAATTTATAT

AGGATTAAGTATGAATAGAACAAGCCCATAAGGGGGTAGAGGAGTGGAGAAACAATTCCC

AAAAATTAGATATGGAGCCCCCCTTTTGTTTGATTAACTCGAATTGTTTGATTAACTCGA

AGTTCTTTAAATATCTCCGCCTTCTTTGAAATATCATGAACAGTTCCTGTAGGTTGAGCA

CCCTTTCCGAGGAAGTATAGAACAGCAGGAACATTTAAATATGTTTGATTCTTGATCGGA

TCATAAAAACCCACTTTCTGAAGATCCCTTCCCTCTCTTCGTGATCGAACATCAATTGCA

ACGATTCGATAGACGGCCCATTGGGATAGATGTGGATTAACCCCCCCCCCTGGAAACGTA

TAAGAGGCTTTCTCCTCGTACGGCTCGAGAAAGAATGATGAGAACTTATGCATATATGCG

GTAAATGCATTTATGAAATAATCAATTAGACTAGAATTGAAGTCGTTTTTTGTTCTTCCT

TCTTAAAAAAAGTATCATTCATACTCATAACTCAAATTAGTTAATTCTCAAGGAGCTCAA

GGGCTTATACATTTATTGAGTCGTCTCTAGCATCTTTTGTTTGTCTTACCTAGGATCCAT

TTCCTCACTTTTTTGAATATTTGGTTCAATCCAGCTGGTAAGGCAATTGCAAAAGAGTGT

TTCCTTGTTTCAATATCTGTTATCTTTACATTGATCCTTGGGTTTAGAACATTGATCCTT

GGGTTTAGACATTACTTCGGTGGTTCTTAATCTCTCAAAAAAGCAGCAACATACCCTTTA

TTGTTTCTTTTTATTGAAGAATCATAGGAATGATTGATTCCTCTGCAATACACTTATAAT

CGAAATAGTTTTATTAATTTCGATCGATTTCACCTTTTTTATTTGAAACTTATTCGAAAT

TGACCCCTTTGATCCTATAGTGATCGTATGTTTACGAAGTTTGTTCAATTTATTGATTGG

TACTAACCCTAGACCCCCTCTCCTGCTAAAAAAATCATTTGGACTCGAGCTCCATCATGT

ACCCTAAAATTCTCATTAGGGATCGTTAGAACAAACTAAACTATGTCGAGCCAAGAGCAT

CTTCGAGGATATAGAAAGTGGCGGATTCTTGCATCCACTGCCAATGATGTCCTTCAAGTC

GCACGTTGCTTTCTACCACATCGTTTCAAACGAAGTTTTACCATAACATTCCCCTAATTT

GAAATTCTAGAACCAGTGTGGAATTGATTCAATATAGAATCAAATCATGAATAGTCATTG

GATTTATTTAATCGGTGCTCCATAATTTCTTTTTCGATATACTTTTTCGATATAGAAGTA

TAAGTATTCATATAGAGAAGCTTTAACAAAGATTTCATTCCATTTATTTGAATGATAAAA

GATGGAATGAAATCTTTGTTAAAAGACATAAAAATGGGGTTGCTTAACGCTTATTTACAC

CTAATCACAAAAAAGAAAAATAGATGGAGAAAATCGAACTTATTTGTTTGTTTTTTATTT

TTATAAGGATAAATATAAAAGAATTGGTGAAATATAAGATTAAGGTCGTTATTCTTTAAT

TGAATTGGAAATTCAATTAAAGTTAATTAGTAATTATTGAAATGAAAAAAATACAAGTCT

GATAAAATCAGAATTGTAGGAGTATGATCTTTCCATATATACATCAGATATAAGGAGCAC

TTGTCAATAGAGTATACTAATGACATTGTCATTATTTATGTTTGGGAAATCACGGGCCTT

TTTCGCCGAAATAGAAATTGCCACGTTACTGAAATACAAGAACAGGAATACATATAAACA

ATGTTTATAGTGTATTAAATAATGAATATAGTGCATTAATGGCTCATTTTCTAAAGCTTT

TCTTTTGCTTTACTTGAAAGTAAAAAACAAAAAAACAAAAATTCAAGTTGAAATTTTGTT

TTTCAAGTTGATAAACCACATTTCCATAAATGACAAATTCCATCAAACTCGAGTGGAAAA

AAAAATAATTGACTTTCCAAAACGATAGAAAACCCCTCTCTTTCCCATTTTGCCACAAAA

CAAAAGCATGTGGTGAGGGAATCATTTTTTCTTTAAAAATACGGAACAACCCATTTTTTA

ACTAACTAACCTCACTCAATATGAATAGAACCCGGGTGAAAGGAGCGGGCATACAATGAA

TAGTCTACCCTCCTTTTCCAAAAAAGTAGTGATTTATTTAATTTATGTCCACATCCCAAC

AAAAAAAGATTTTTGCTTCCATCCACGCGGCATTTAGATTTCTACCCTTGTGAGAAACAA

CGTCGAAGAGGATTATAAATATATATATAACAATCATTAAAAACCCAAAGGAAAAAGAAA

ATGACATTGTATCCACCACTGATAGATACGCAGAAAAGTATGTTCTTTAAGTTTCTTCTG

TTCTGAGGGAACAGCTCTGGGACGGAAGGATTCGAACCTCCGAGTAACGGGACCAAAACC

CGGTGCCTTACCGCTTGGCCACGCCCCATTTATATGTCGATTAGACAATAATAGACAATA

ATATTGTTATTGTCTGTTCGTCAATTCCAACCTAAATATCTACCGAATTGGTTGTTGCTA

AGATTCGATACGTGGAAACGTAGAATGAAAATAAATTCATTGATCATTGCATAGAATTCC

ATTAAGATATTGTATGAAAATAGAATTCCATCTTGATTTTCATTTTACAATTGAAGGGTT

TTGGTTGGGGGAGGGAAAAGAAAAAAGAAGGATTTTTTTCTTTTCCCCCATATCAATAAC

TCAATAAAAAATGAAATTATCTCTAATCCAATCCAAGAACGAAATGTTTGTTATGCTTAA

TATCTTTAGTTTGATCTGTCTTAGTTCTGCCCTTCATTCGAGTAACTTTTTCTTCGCTAA

ATTGCCGGAAGCTTATGCCCTTTTCAATCCAATCGTGGATGTTATGCCAGTCATACCTGT

GTTCTTTTTTCTCTTAGCCCTTGTTTGGCAAGCTGCTGTAAGTTTTCGATGAGATTTTAA

ATTGGAATACTAAAATATTCACCACGATAGATTCGAAGAAAAAATATGTGTTCTAACAAA

ATGGGGAAAAACAATTGCTAATATCGGGTAAATCATACATTATGAACCTTCGATTCATAC

ATAGAAATTCTCTATGGATTGGATTATGAATATGGAGATCCGCGCGCGGGGTCTGGATCA

GCTAATGTCTTCTTCATTCTGATCTTACGAGCTATTCTAAATAAGGTTGCCTCAAACAAA

ATACTAAATTGGGGAGATCCTTTTGATAACCAAGGATAGAAGTCGAATTTTAGGACAGAG

AAATTTCTTAAAATCCTTTTCTTGGTGCAAGATATTTGGGACAAAAATAGAGAATCTATT

CCCTTATTTTCCACAATATAATTTGGAGATTGTGTAATGCTTACTCTCAAACTCTTCGTT

TACACAGTAGTGATATTCTTTGTTTCTCTCTTCATCTTCGGATTCCTATCTAATGATCCA

GGACGTAATCCTGGGCGCGAGGACTAAAAAACCCCCCTTTCTTCTTGTTTTTTTTTTTTT

TATTTTCTTTATTCTTTAAAAAAAAATGATTTTTTTTACATTTCATCCATTTAACTATGA

AAATGGAACTAACTATGAGAAATAAAGCCGAGACTCTCGATTTTTTCGAATTCCAACGAA

TTCCCGATAGAAACTAAAAAGGATCCGAAGAAACGGAGAGAGAGGGATTCGAACCCTCGG

TACGAATAACTCGTACAACAGATTAGCAATCTGCCGCTTTAGTCCACTCAGCCATCTCTC

CCAATTCCTAATTCAAAAATTAGATAATTCACATGTCAATTATGAATGAAAAATAGATAA

AAGTCTTTTTCTTTCTTTATTTATTCTCTTTTTTATTCTAAATAAAGAATTTTTCTATCT

ATTTAGAAAGATAGAAATTTCATAAGCAATTGCAATTTTATACCCATTTTTTTAGCAATT

CCGTTTGAATAATGATTCAGAACAAAAATTTCCAATAGAAAAAAAAGTACCTCTTTGATT

TACTACGAAAGAGTTTTTTACTCCCCCAGCCTGGCTAGTACCTAGCCGGGCCATTCTTTG

TTTTGCTTCTTGTCATTTTCATTTCATTTCTATGATTCGTATAATCTTTATGTATGATAA

AGGTTCTTTTTCTTACTTCTTTTTCTTTTATCCATTTTTTTATTCAAAAGAAAAATCTAT

ATCAGATTTCGATTCCGACGAGAATCCCCCCTTTTCCTCACGGAAAAACTTCGTTTGTTT

GAAATGAAATCCACAAACAAAGCGAATACTATCTTTATTAGATCTAATGAAATTAACTCA

ATTCATAAGATCTGGGGAGTGTATGAGAATAAATGAAGTAAGGAGGAATCGCTCCGAAAA

GGAAGAGAAAATACAACCAACCCCCCTCCCCCTATTCGACAAATGATGTATTTATATACA

TTATATATATTATAGCGGGTATAGTTTAGTGGTAAAAGTGTGATTCGTTCTATTAATAAC

TGAAATAGTTAAGGGATCCTTTAAGTTTAACCGAGATTCCATTCAAAAACTTTATTTCTT

AGAAAAGGTTTAATCCTTTACCTCTCAATGCGCCATTTGGAGAAGAAGATAGATTCTCGT

GATTTATATCCAATCCAAAAAGTCAATTAGAAATTGAAAAATGAGATTATGAAATCGCGA

AACATAATTTTTTTTAGTTAGATCAACCCTTCAAAAAGGCTCCATGGATGAAGATCCAAA

AGTTTATTTCTAATCGTAACTAGATCTTCAACTTTTTTTGAATGTAAAAAGAGAGATTGA

AGCCAAATAGCTAGTAAACAATGACTTTGGTTTACTAGAGTCATTAACATATTCTTTTAG

CTCGGTGGAAAGAAAACTCTTTTCCTAAGGAACCTCGCGAGTAGAAATAAGGAACGAAGT

AACTAGAAGGATTTGTTAGAATCGATATCTTCTAGAGGGATCGTCTAGAAAGTGAGTCGT

TTTGGGTGCAGGTGCATTCAAACGCAAAAGCTGACATAGGTGGTATGAATCGAATTTTTC

TTTGAATTCGGATTAGTTATGATTCCCCTTTCAATACATCGAAAAAATTTTCTTTTTTTT

TTTGTTTTGTTTATTCCTTAGGTCTGGGAGTTTTTCCATCTTCTATAGAGGAGCCGAATG

AAACCAAAGTTTCATGTTCAGTTTTGAATTAGAGGCGTTAATTTAAAATGATGAATCGAC

GTCGACTATAACCCCTAGCCTTCCAAGCTAACGATGCGGGTTCGATTCCCGCTACCCGCT

CCATACAAATTCTTATTATGTAAGAACAAAATGGGAAAGAAAAAGATTGGAAAGAAAATA

AGCGTCCATTGTCTAACGGATAGGACAGAGGTCTTCTAAACCTTTGGTATAGGTTCAAGT

CCTATTGGACGCAATTTATTTCTATCTATTTTTTTGTGGATCTCTATACCAAGAAGCTGT

TTTTTTGAACGATTCGAGTCATAGAGACTTCTGAACGATTCTTTTTTTTGTACTAAAATT

TCTTTATGTTTGTTCTTGAAGTAGAAACTGTTCTATCTGTTCTGGAATTACTTCCTTTAA

AATGGCTTCTGCGTCTTTGGTGAATATCTTGGTCGAAGATATTATTTCTTGGAACTGAGG

TTTATTTCTTTTTAAGTAAGTACGTAATTGAACTAGAAATTTCTTTACCTGCCAAATTTC

TAAGGGATCAAGATACCCATTCGCTCCAGTATAAATAGTAACTATCTGTTCTTCGACCGC

AAGGGGAGCTGATTGAGATTGTTTAAGCAACTCGCGTAATCGTTGACCTCTTGCCAATTG

GTTTTGGGTGGCTTTATCAAGATCAGAAGCAAATTGCGCAAAGGCTTCTAGCTCTGCGAA

TTGAGCTAGTTCCAATTTTAATTTACCAGCTACCTGTTTCATGGCTTTAATTTGAGCCGC

GGATCCTACTCTGGAGACCGAAATACCCACGTTAATAGCCGGACGAATTCCAGCATTGAA

GAGATCCGCGGATAAGAATATTTGTCCATCTGTAATCGAAATCACATTAGTGGGAATATA

AGCCGAAACGTCCCCGGATTGAGTCTCAACTATTGGTAAAGCGGTCATACTTCCTTCACC

TAAACGAGAATTTAATTTAGCAGCTCTTTCCAAAAGGCGTGAATGTAAATAAAAAACATC

TCCTGGATAAGCTTCGCGCCCCGGCGGCCTTCTTAATAGAAGAGACATTTGGCGATATGC

TTGTGCTTGTTTGGAGGGATCATCATAAATTATTGAAGTATGTCGTTCACGGTACATAAA

ATATTCAGCAAGAGCCGCCCCTGTATAAGGCGCAAGGTATTGTAATGTCGCAGGGGAATC

CGCCGTTTCGGCTACTACAATAGTATATTCCATTGCCCCTTGCTCCTGGAAAGTAGTCAC

TACCTGAGCCACGGAGGATGCTTTTTGACCAATAGCTACATAAACACATATTACATTTTG

CCCTTTTTGATTAAGAATAGTATCTGTGGCTACTGCTGTTTTTCCAGTCTGTCTGTCCCC

AATAATTAATTCTCGCTGGCCGCGTCCTATAGGGATCATCGAATCAATAGCAATAAGACC

TGTTTGAAGAGGCTCATATACGGAACGTCTTGAAATAATGCCCGGAGCGGGAGATTCAAT

TAACCGAGATTCAGAAGCAGAAATTGCACCTCTCCCATCAATGGGTTTAGCCAGAGCATT

TATAACACGCCCCAAATAAGCCTCACTCACCGGTATTTGAGCAATTCTTCCTGTTGCTTT

TACAGAACTTCCTTCTTGTATCATCAAACCGTCGCCCATTAATACAACGCCAACATTATT

TGATTCCAAATTTAGAGCAATGCCTATTGTACCCTCATCAAATTCTACTAATTCTCCTGC

CATTACTTCATCAAGACCGTGAATACGAGCAATGCCATCGCCCACTTGAAGTACGGTGCC

GGTATTCACAATATTGACTTCTCTATTATATTTTTCAATACGCTCGCGGATAATATTACT

AATTTCGTCAGCTCGAAGGGTTTCCATTAATGTCTTTTAATTATTTTTCGGAAGCGAAGG

GGAAAAATAAAAATAATACCTTATCTTAATAATACCTTATCTTATAGTATATTATGGTTG

CTTCATATTATATTATAGTTTTTTTCGTTTTGTTTTATAGTAGATAGAGTTATAGTTTCT

GATAGATAGGGCGGCTAATCCATTATTTCTTTCATGGCACCGAGAATGCTAATATTAGAA

CTGATAGTTCGTAGATGTAACTCTTTGTTCAAACAACTATTCAAGGTTCCTAGAGCTCCT

TGTAAGGCTTGTTGGAAAACTCGTTGTCGGACCTGATTAATTGCTTTTTGTTGTTCAAAA

TGAATGGTTTCATTTTTATAATTTTCTAATCGTTCCAAATTCTCAGAAGCAGCATTAATC

AAATTTGATTTTTCGCGTTCTATCTCAGAGTACCCATTCACTCGAAACTCATCTGCCTCG

ATTTCTACTTTCCGCAAGCGAGTTCGGGCTTTTTCGAGCTGTTCAATGGCTGCTCCACGT

AGTTCTTCTGAATTTCGAATAGTGCTCAAGATCCTCTGTTTTCGATTATCTAATAAATCA

CTTAATGAAAGTAGATTTCCGTCCTATTTATTTCACAACCTTCATGATCTCTTCCCGAAC

CAAACATGAATCTTTCGATTCATTTGGCTCTCACGCTCAGTTACTTATAGGGCGTTCCTA

TATCTTTTCATGTAATGAGCCTACCCTTTCCTCTCTGTTCGTATTCCAAGATAGTGAAAC

GGAAACAAGATCAGGATATTCTGAGGGTTCTTCCGACCAAACAAAATTTTGTAATTGCCA

GCAAAGTTGTTTCTTTTTTTGTCTTTTTTCTTCAAATCCAAAAAATTTTTCTTATTTTAT

TGATACTTTCGCTTTATATACATAATAACTATAATATAGGTTGCCGATTCAGCATTGTTT

AAACAAGCAGGTGTCTCACCCATTTCCCTTTTCCAGTAAATAGTTCAGTTCAAATCACTT

TATTGATATGAGTGTTCTATATCAGATAAATTGACAACTTTTTTGAAGCCGTCTCCTACT

AACTCCTACTAACGTAGTGGTAGAAAGAGTACCACGCTGTGTCTTGACTTCAAACGGTTT

TGCTTTAACCATGTTAATGATACTACATTATTGGTTTATAGAGAAGAGAGTCAAAGTGGA

TTTACCAATAAAAAAGTCACGAAATGCTATAGTTCTTACATATGATTTCTTAATTTATTC

AGAAGTAATTCGTCGAGATTGTACACCCCCCCCTCCTATAAAAAAAAGTGCAGCTGGTTA

GATCCAGCCTTTTTTTTGAAATAAACAACTCGCACACACTCCCTTTCCAAAAAAGATCAA

TACACCAAGCACTACACTTAGATTTATTGGATTTGTTGCTAAAATATCGGTATTAAACCC

GAAACTCCCGGCGCATGGCCAGTGACCTAAGGAAACGAAAGAATCGGTTACATTTTTCAT

ATGCTCTCCTCTTATAAATAGAACTAACAAAATCGAACAGAGTTCTGTATAAGTTCGCCG

CCACTGTTTGGATTGATTTCTTTTTTTTTTTTTTCTGAATTTCCTTATTTAATAAATAGA

TTGATTTTCTTTTTCATCTATTCGTTATGATACGAATAGTTTCATATTCATTAATATGAA

TAATTTTATTCATATTAATAATGAAGATTTCAATGAAAAAATAAGATACTTATTGCCTTT

CTAGCCACACGGAAATCGCGAAAGACCTCATTTGTTACAATGCTTCTTATTAAATTAAAT

AATAAAAAAAAAGAAAGAATTTTCTATTTTCTATTAAATAAATTCACTAGGGGGGGGGGG

CAAAAGCGGGTGGATCCTCTAATTCCTTATCCTCAAGCTAGCCCTTCCCCTACCTCTAGG

GTTAGGGTGATTGTCCCAACGAATAAATAGAAAAAAGAATTCGATTAATTCATTTTAGGG

GTAAAGCGTCGGTATAATGCGAAAAGCGGGGGTTCAAGTACATGGCAATAAAATACTAAA

ATGAAATACGTATTTTTATTTTCAAATTTCTAGGATTAAACAAAAGGATTTGCAAATAAA

AGGGCTAATGCCACAACCAGTCCATAAATTGTTAAAGCTTCCATAAAAGCCAGACTAAGC

AATAAAGTACCTCGTATTTTACCTTCTGCTTCTGGTTGTCTCGCGATACCTTCTACAGCT

TGGCCTGCAGCAGTACCTTGACCAACTCCAGGTCCAATGGAAGCAAGCCCTACTGCCAAT

CCGGCAGCAATAACAGAAGCGGCAGAAATCAGTTGATTCATGGTAAATTTTATTCCTCGT

ACAAAAAAAGAAATGGTTAATGATACAATCAACCAATGAATTATCGCCTCCCATTTGATT

TCATCATTAAGACCTAACCCAGCAAAAACAAACAAGTAGAGGTAACTAAGAACTCAAAAC

GAAAATGATGCTATTTCTGAATCAGCAGAACTGCTTCGGCATCTTGTTTGTTTATAGTTC

CCACTATCTAATGTAGTCTTTCTTTGGAAATCCATATGGTTCCGGTTCTTCGGTTTCTTT

ATTCCAAAACACCCTTTCATCCGACTTTTGATTTTACTTTTTTTTTGTTCGTTCTCTTCT

ATATGCTTGACTTCATCTATTTATTATAAAAATACACAGTCAGAGATCAAATAGAAAGAC

TTCCGATAGACGGATTCATCTGTTTATGGGCAGTCCGTATCTAACTAATGAATTTCGAAT

ATTACATATACATTTGTTTCGTCCATAACGTAAACCGCTCGTTCGATTGAGTCGGATTCT

ATCTAGTCTTAAAACATTCTTCGAAACAGCCATAGGAATTGGCTTATGCTTATAGACATT

ACATATACCCAACGAAATCCCTCTAATCAACTTTTTTTGAAATCTTCTTTGTTTGTAAAC

TCTTCTCTTCCTTTTCTTTATAATTATCACACAATTACAATCCCGCAAAAATATAAACGA

ATGGGTGGGGTCATTAGCCTAGCTAGAATTGAATGGTTCTATATCAAGATTTTATTATCC

AATTGCAGACCATTTTGGTCAATCGTTGAATTCAATTGAAAAAGGAATGTTTCCTTCTAT

GGAAAGAACGTTGTTTTTGTTTATAAACACATGTTGGAAAAGAAAAAAATAATATAAAAA

TGGATATTATATGAATCCTAATATCGTAATTGACTCGGCAAATCTGGAAAAAGAATATTT

ATTAGATTAGAGTGGAAAGATATGATCGAAATATACCAAAAGGGGGGTTAGGAAAAAGAT

CTTTTTGATCTCTTTCCTTTTTTTACAATTCCCCAATATCGTATATCTTTCTTGTTCCTG

CTCTATAGATCGTATCTATCCAATTTTTATACCTATTTTTATATATAAATTCCTTCATTA

GATTATCTTGAGTCACGCTTGAGTTTTGGAATAAGCTTTTTTTCCAAAACAAAAAGAAAC

TTTTGGGAAGCTAGTTAATGATGACCCTCCATGGATTCGCCTATATAAGCCGCAGCTAAA

GTTGCAAAAATAAGAGCTTGAATTCCGCTTGTGAATAATCCAAGAAACATGACAGGTATG

GGAACTACTGAAGGTACTAAAGAAACAAGAACAACAACTACTAATTCATCAGCCAATATA

TTCCCAAAAAGTCGAAAACTAAGTGATAAAGGCTTAGTGAAATCTTCTAGGACGTTAATT

GGTAAAAGTATTGGGGTTGGTTGAATGTATTTACTAAAATAACCCAATCCTTTTTTTGTA

AGACCCGCATAGAAATATGCCACTGACGTGGGTAAAGCTAAAGCAACAGTAGTATTTATA

TCATTCGTGGGTGCAGCTAACTCTCCGTGAGGCAACTGTATGATTTTCCAAGGTAAAAGA

GCACCCGACCAGTTCGAAACAAAAATGAATAGAAACATAGTTCCAATAAAGGGAACCCAA

GGGCCATAATCTTCTCCAATCTGAGTTTTGCTCAAATCTCGAATGAATTCGAGAACATAT

TCGAAGAAATTCTGGCTATTGGTTGGAATTGTTTGTGGATTTCGAACCGCTATAGTGACT

GAACCTAATAAGATAGCAATTACGACCCAAGAAGTGATAAGTACTTGGCCATGAACTTGG

AAACCCCCTATTTGCCAATAGAGGTGTTGACCTACTTCCACACCAGATATATCGTACAGC

CCCTTTAGTGTGTTGATGGAACATGGTAGAACATTCATATTACCCTCTGTGACAGAAATA

GAACTTTTAAAAGAATTATTTTGATTCAAACATCTCTTCTCTCATCTCTTCTCTTTCTCG

CTTCGCCTAGCATCGTCGATTTTGCATACCAATACCAAAGAATCACATAATATCCCCAGA

AATTTTGATCTCTTTTTTGCGATTCAGGATATAGTAACCGATTCAATGAATCAATCTATT

GAGTTCCAAAAGTTTATTGACTTATCTTATTATTAATCAACGGTTTCTTATATAGCTAGA

ATGGCCCTCACAAAGTGCAGATACTAATTTGTTAAGAATCAAGCGGATTGAAGCGATAGC

GTCGTCATTAGCTGGAATCGAAATATCTGCCAGATCTGGGTCACAATTTGTATCGATTAA

ACAAATCGTCGGAATCCCTAAAGTGATACATTCCCGAAGAGCCGTATATTCTTCATGCTG

ATCAACGATTATTACAATATCAGGCAACCCCGTCATATACTTGATTCCACCCAGATATGT

TTGCAAATGAGATAATTTTCTCTTCAACATTGCAGCATCTCTTTTCGGTAGACAGTTGAG

TTTCCCCGCCCTTTGTTCCGCTCTCAAATCCCTGAACTTATGAAGTCTCGTTTCTGTAGT

GGACCAATTCGTTGACATACCACCGAGCCATTTTTTATTAACATAATGACAGCGAGCCCT

TATTGCAGCTAATGCTACTGAATCCGCAGCCTTACTTTTTGTACCAACTATTAAAAAGTG

CTTTCCCCTACTTGCTGCGTCAAAAACTAAATCACAGGTTTCTGATAAAAAACGAGCAGT

TCTAGTAAGATTTGTAATATGAATACCTTTACGCTTTGCAGAGATGTAAGGTGCCATTCT

AGGATTCCATTTCCTAGTACCATGACCGAAATGAACTCCCGCTTCCATCATCTCTTCTAA

ATTGATGTTCCAATATTTTTTTGTCATTTCTCCCCACATTTTCTTTTTTTTTTTTGTTTA

TACCCTGAAAATAAATAATTGTTCCGAAGGAACTTTCTCCCGTAAATGGACCGTGCATCA

ACGGCCCAGGCCAAAATAAAACGGGGAATATCTTTCCGTTTGGTATTATCTTTAATACCA

AATCAAACGACTGGTTCAATTCGAATTAGTTAAAAGAAACGAATAAGTCCGCTTTTAGAA

ATTTTGAATTCCTGTCAAAAATTTCTCTTATGTGTTGTATCATGAAAATTTATTTTTGTT

TTTGGATATGCAAGAACTAAAAAAGTCTCTGTGGTGAAACAAAATATTGCTTATTTCCCC

CTTAACCAAATTATTCCTTTTTTTTTCCAAAGAAATGTTGTTGTGTTGCCTTGAGCGGCG

GACAATTCCTTTGAATCCGGTACCAACGGGTATCATCCCACCCAGAACAACATTCTCTTT

CAGACCTTTCAACCAATCAATACGACCCCGCAGAGCAGCTTTTGCTAAAACTCGGGCGGT

TTCTTGAAAACTCGCTTCGGATATGAAACTTTGAGTATTCAAAGATGCCCTCGTTAATCC

CAATAAGATTGCTCGGTAACAGATTGTTTCTTCCAAAGCGCGCCCTGTCCGCTCTGCTCG

CAACAACCCGATTAGTTCTCCGGGTGAAAAAGCATTTGACATTCCATCTTCTGAAACCAA

AACTTTTGATGTTATTTGGCGTACAATAATCTCTATATGCCTATTATGGATCTGCACCCC

CTGGGATCGATAAACCTTTTGAATCTTATTAACCAAAGAGATACGACTTTGCGCTATGGT

TAACTCAGCGCCAATTAAAAATCCCCAAGGAATTCCAAGAATTTTGGTTATATTTTCATT

CCAACCCTCAACCCTCTTTTCTAAATTCATCGATATTGAATCAATCGAACGAACTTCCAA

CACCTGTTCTACTTTTGGAAGACCTTGCGTTATATCACCCGATCTCGATTTTTCGTATAT

AAATGTAACTAATGTATCTCCTTCGTAAATTATTTCTCCATAATGGCCATGAACGGTTGC

TCCTGGAGTGGCCAAATGAGGCTTGGCCGATCTGATTACTAAGGAGTCACCATGAACGGT

TACAATTTGTCCCGATTTGATGTGTGATCTATATTTGTATATAGATCCATTTTCATAAAA

AAGCTGTCCAAGGCTAATTATTGAGAATGTCTTCTCACAAGAATTGTGATGTAGAAAACA

CCAATTCAAATCGAATGCATTAAAAATGGTGTTACTACATGGATCGGGATTCAAAATTCT

ACCCTTTTCATCTATTAAATAATAGAGAACGTCTTGGAAAAGGTTTTTGAAATTGTCAAG

TATTAAATATTTCTTTAATAAGATCTGATTATCAGTCAGATAATAGTTTCGTGAATCAAA

ATTCGTAATTTTAGCTATAGTCCCTAAGGGCCCGAAGGAATTTCTAATCAAAATAGCGGG

ATCCTCCTTAATTGATTTTTTTTTCCCATTGTCAGATTTCGAACCATTGACTTTGAGGGG

ACCAACTCGAAAACAATTCGATGATGACAAAATGAGAAGGGATGGTAATTGCTTATTGAT

ATTCAACGACGTATGAATAGTCCCGTGATCTTGAGTAAGAGATTGAATCTTAATCTTGGA

ATAAAAAGGATTTCTATTGGGGCAATCTGATCCATTATCTGGATTCAATCCGTAGCCTGC

CGTATCATTTCTTTTTCCAGTATTCAAAACGCGGGGCTTCACTAAATCAACTCTTATGAA

ATCTCGAATCAGATCATTTGCCCTTACTTCAACAAAGGAAGCACAAACTTCTTCTATAGA

ACTTGTTTTTTTGTTGTCTTGATCCCAATTCAATACTAAACAAGTTCGAACTAATTGAAT

ACTTGTGTGAGAAATTCCTCGAATGGGTTTGCCATTTCCGTAAAGAATATAATTGACGAC

TCGGAGTTGTACATTATCCCGTTCTTGCAACGAATCCTGGGGAAAAAGTGTTGCTAAGTT

TATGCCATCTGCTATTTCGTATGTAACTACGGGTCGAACCGAAACAAAATACTTTTTCTT

GATAGGTGTAACCCGTTGGACATAGATCCAATTTTTCCATTTTTTGGATTCCTTAGAGTT

TTTTTTTTCCATTCCTGGCGGTAGCAAGATTCCGCTATGTCGAGAGATTTTATTTGTCTC

TCCAGGAAAATGAATATTTCCAGAAAAGATTTTGAGTTCAATCCTTTTTTTTTTTCTCTC

CACTCGGACTAATCCACCTACCTGACTTCTTGTATTTAAGGCGATCTCTGTATCGACTCT

AATGATACTATTGTTCCGTACCATTATGGACGAAGACCCAGGTAAGATATGCACTTCCTC

GGGAATGAAAAAAAATCGATCTACTTTCGTTTGGTATTTCGTCCTCAATTCAAAAGCTCC

TCGATATTCAACCAAATCCTCTTTTTTTACAAGAGAATCCACCTCTACGGTCCCATATTT

AGTAATTCCCGAGCCGCTTCTTCTGTATCGGGGATCGTCGAAGTAAGCAAGAATACTATT

TCTACGTAAAATACCAGTTTTGGGTATTTCAATGGAGATACCAGAATAAGGCATTGGTTC

TTTCTCTCCTTCTTGCGAATATTGGAATGGAATGATGAATCTATTTCTTCGCCTCTTCGC

CAAGAAATCCGAATTCTCGTGGAGAATGGCAGAATATATAAAATTCCAACAATCGTTGGG

TATGCTTTTGTCAGGTCCTGAATAATCAAAAAACCTACCCCCCCTCCCCCCACTGGAAGG

ATCCGAACTAAACGATTTGCGTCTCACTCGATCATTAGTCACTGAGAAGTCAGAAATTGA

TCTTTGTTCTAAAGAAAGAGAATAAACATTCATTTGATCTTGATCTTTGTGTAGAGAAAA

GAGTGCTCGATTAGATTTGCACGGATTTCCTGATAATATCCATAAATGGCTTGTTTTTGG

TAATAGATGAACATTACCATACGTGTATTCGGGGGTATGGTATACATTGGTACTCCAATG

CATTTCTCCCTCTGAATCAGAATAAATATGTTTTCGAACTTTTTCTTTAAAACTTAAAGC

GGATGCTCCGGCACGAATCTCAGCAATCACTTGTTCCGATTCTACATATTGATTGTTTTG

AACTAAAATAAAACTTTTTGGGGGAATATTCACTTTATGTAGAATATCCCGACTTTCAAT

AGTTACATAAAGGTCTATATAACATAGAAAGGCAGGATGCCCATGCCGTGTACGTGTGGG

ATGAACCAAATCCTCATGAAATTTTATTTTTCCATTCGAAGGAGCTCGTACATATTCTGC

AGTACCACCTGTGAACACTCCACCGGTATGAAACGTTCTTAATGTTAGTTGAGTGCCCGG

TTCTCCAATTGATTGACCCGCAATGACACCCACCGCTTCCCCCAACTCAACTAGGTCGCC

ATGAGTGGGACTCCGACCATAACATAATCGACAGATCCAAGAAGTGCTCCTACAAATAAA

AGGAGTTCGAATATATATTGATTTCGCTTGAAAGGTTATGAAGCGATTGACAAGTCCAAT

CCCAATATCCTGGTTTCGGGCAGCAATGCACCGTGGACCCATATATATGTCATATGCTAA

TACATGACCAATTAGTCTTTGGATCCAAATTTTTTCTTCCATACTATTTTGAGGACTCGC

CGAAATACCTCGGATAGTACCACAATCTGTTCTACGTACAACAATGTGTTGAACTACTTC

AACAAGTCTACGTGTGAGGTATCCAGCATCGGATGTTCGTACAGCAGTATCCACAACTCC

CTTGCGAGCTCCGTAGTAGGAAATAATATATTCTGTTAAAGAGAGTCCTTCGCGTAAATT

ACTTTGAATGGGTAAATCAATCATCTGTCCTTGGGGATCCGACATTAATCCTCTCATACC

TACTAATTGATGCACCTGAGATGCATTTCCCCTAGCTCCTGAAAAAGACATTATATGGAC

TGGATTAGAAGGATCGGTCATTCGAAAATTAGTATGCATTTCTTGTCTCAAATATTCACT

TGTAGCATACCATATCTCAATAGATTGACGTAATTTTTCTACAGCGTGTACATTCCCATA

ATGATGGTGTTTTTCCAAAATCAAACCTTGTTGTTCAGCATCTCGGACTAGCCATCCCTT

AGAGGGTATTGTTAAAAGATCATCAATTCCTAATGAAATGGATGTAGCGGTGGCTTGTTG

GAAACCCAGAGTCTTTACTTGATCCAGTATATGTGATGTATATGCCATTCCGAAGTGATC

TATTAATCTGCTAATAAGTCGTTTCATGGCAGTTCCATCTATCGCTTTATTGCGAAAGGC

CAGATCTGCCCGTTGTTCCGCCATAAGTACCTCCGTAGTCCGCTTAGTAGGATTCAACAA

TGAGTTTTGAGCCAGTGGTTCGAAGACTTCCTTTCCTCGATCTTTATTCACATAGAAATT

CGGGAATTGTTATAAGGTAACAGTTGAACCAGAGAGGCAAAAATAGCTACAGATAGTACA

TAATTACCTAGGTACCGCCGTATGAGTAGGCCCGACAAAATCCCTGTATGGCTTCTTCTA

TTTCTCGATAGAAAGAAATATGGCCAACAGTGGTTCGAATGTATATACAAAGGATTTCTT

TTTTGACACTTTTTACTATTAGATAATGCCCATAAATTTCATGATAGGTACCCAAGGATT

CATATTGAACTTCGATTGGAACTTCTCTTAAGGAAATGACACGTTGATCTAGTCGCCACC

GGAGCCACAAGGGAGTATCTAAATGGATTCGTTTCTGCCGATAAGCGCCAAGTACATCAT

AGGAACTACAAAAATAGGGTTCTTTTTCTTTTGTATACCGATATTTAGTATGGTCAACTG

TTTCATTTTGATAGTTCCTGCGATTCCATGGATTATACCTATTTGCACAAACACCTTGAC

GATTTCCGATCGTTAATACATAGAGTCCAATAAGTATATCTTGAGTTGGTACGGAAATAG

GACTTCCTATAGCTGGAGACAGGAGATTCATATGAGAAAACATAAGTAAACGAGCCTCCG

CTTGCGCTTCCAAAGATAAAGGTACATGAACAGCCATTTGATCCCCATCAAAGTCTGCAT

TGAATCCCTTACAAACTAATGGATGTAAACAAATAGCACGTCCCTCTACTAAAATGGGTT

GGAACGCCTGTATACCTAATCTATGGAGGGTGGGTGCTCGATTTAGCAATACGGGATGAC

CCTGCATAACTTCTTGAAGTATTTCCCATACAATGGGCTCTTTTTCTCGAATTTTACTTT

TCGCAATCCCTATGTTGGAAGCAACGCGCTGCCTTATTAGACCTCGAATTAAAAATGTCT

GGAAAAGCTCTATTGCTATTTCTCGAGGCAATCCACATCGATGTAATGAAAGCGAGGGGC

CCACGACAATGACGGAACGCCCCGAATAATCAACTCGCTTACCAAGCAGAGTCTCGCGAA

ATCTTCCTTCTTTCCCTTCAATTACATCTGAAAACGACTTGTAAACTTTATTATAACCAT

CCCTCATGGGTTGACCGCGAATACCATTATCAAGAAGTGTATCCACGGCTTCTTGCACCA

ACTTCTCTTGACACATTACTAATTCCCCTGGCGTGGATCTGTGTGTTGTTAATAGATCAG

TAAGGGTATTGTTCCGATAAATAACTCTTCGATAGAGTTCATTAATATCCGAACTCATTG

GCTTACCCCCATCTATCTGAATGATTGGTCTCAACTCGGGAGGAAGAACTGGTAATAGGG

ACAAAACCATCCGTTCTGGTTCTACATTTGTTCGAATAAAATGTTTAGCTAATTCTATAC

GTCTAACCAAAAAATCCTTTCGTCTTCCTATTTTTCTATCTTCCCAGTCATTGCCAGTGG

ACCCTTCTTCCCCTAATTCCTTCCATTCCACCAATGAACAATCTATAATAATTCGCAAAT

CTAGATCGGCTAATTGTTCTCTGATAGCACCTGCTCCAGTAGATATTTCTCGATTTCGAA

ATGTATCGAAGCCTTGGGTAGTAAAAAAAAGTGGGATGCTATATTTCCGAGATTGGATTT

CATATTCGAATAAACCTCGTAATCGTAAGAAAGTAGGTTTTTTAGATACGGGCCTAGCAA

AAGAAAAATCTGGATAGGTTCCCTATGGGATTCCCCCCCTTAAAAATCGGACGTGAGTGT

TTCCTCTCATCCGGCTCAAGTAGTTACATCAAATAAGGAAAGGACTTCCTACTTTAAATT

TTTAAAAATTTAAAAATTTTTGTTCTAAAGAACCTCATAGAGATCTACCCATTACTCAAG

TTAGCGGCGAAAACCAACCAATATTTTATTGATTCATTCTTACTTTGACTTTTTGAATTA

GTTATTCAATCACGACATAAATGAAATTTCATTGAATTGAGTAGTCTATTTCCCTTCAAA

TTAAAAATGAAAACCCCCTTCCTTTTAGTTAAAGGGATAGCTTGGAACTCATAAGGGATT

TACTTGTCTATTTTTTGTTCCGCTCGGTCGTTTAGGTCCCTACTTCACCTCGACGGTTAT

ACCATGATGTCCCTTGAAGCATATATGCGATAGATAGACTCCTGTAACCGTGTTAGATTC

GCTTACTTGAAAGGAATCTCTTTCGAAAAGAAATAGAATGGTTAATTCCACGAAAGAAGT

TTCTTTGTTAACGAGGTATACCTAGCAATTCCTATTCCTATATTAATTGACCATGGACCA

ACCCCCCTTCCTTCCTATTTCGATATTTCGAAGTATTGATCACACCCATACATAATTCTG

AGCTTCATGTTCCTCCTCTAAAAAGACATGTCAGAGCCGGGGGCATCCCCAATCGGATTG

AATGGGATGACAGTTTATCGTCCAAATCTGTAAAATCAAAATTTTGATCAAATCACACAT

CGCAATAGACTAGGCCTTCTAATTCCTTAAGGGGTTTATCTAACAGATTTGCGATATAAC

TAGGAAGACGTTTCAAATACCACACATGAGTCACTGGACATGTCAGTTTGATGTATCCCA

TTTGATATCTTCGTGCCCGAGAATCCGCAAATTCGACTCCGCATTGTTCACAAAAATTCG

GGTCTTCTTTTTTACCTCCGATCACTCGATAATTTCCACAAGCACAAATTCCACTTTTTA

TGGGCCCAGAAATTCTTTCACAAAACAATCCATCCTTTTCCGGTTTATTCGTTTTGTAAT

GAAAAGTATAGGGTTTTGTCACCTCTCCAACAATCTCGCCATTAGGTAGGATTTTGTTGG

CCCACGCACTTATTTGTTGAGGAGAAACTGATCCAATTCGAAGTTGTTGATGTTTATACC

GGTCGATCATAGAACAAAAATAATGATTCATTCCGATCAAGCTTCCTTCCTATGAATCTG

GAAGTCCTTCTCAGATAAAAGGAAATGATTCAGTTCCAAAGCCAAAGATCGTAGTTCTCG

AACGAGTAGTCGAAAAGATTCTGGAGCATCCCCGGGATTAGCTATTGTTCCTCCAATGAT

CGTAGTACCAAGTACTTCCTGGCGAGATCTAATATGATCAGATTTATAAGTAAGCATCTC

TTGTAAAATATGAGCAACACCAAACCCCTCTAGGGCCCAAACCTCCATTTCTCCTACGCG

TTGTCCCCCTTGCTTCGCCCTTCCCCTAAGGGGTTGTTGTGTAACAAGTGCATAATGCCC

ACTGGAGCGTCCATGGATTTTATCATCAACTTGATGAATTAACTTCAGGATATAGGACTT

TCCTATTAAAACAGGTTGTTCAAAAGGATCCCCTGTTCTTCCATCAAATATTCTGCTTTT

TCCCGGATACTCGGGCTCAAATACCCATGGATTTGCTGTCTGCTTACTAGCCTCATATAA

TTCAGAAAACACTAGTTTTCTCGAAGCTTCTTGCTCATATCTTTCATCAAAAGGTGCTAC

TCTATAATGTCTGCCTAGAAAGTCCCCCGCTAACCCTAGCGAGCATTCAAATATCTGTCC

CACATTCATTCGTGAAGGTACTCCCAATGGGTTGAAGACCATATCAACCGGTGTTCCATC

TTGCAAATAAGGCATATCTTGCCTAGGCAGAATTTTGGAAATGATACCCTTATTCCCATG

TCTTCCAGCTACTTTATCACCTACTTTCATTTCACGTTTCTGTGAAATATATACACGAAT

TGTTTCTGGATTATAACTGGAACCCCCCTTTTTCTGTATCCATCTCACATCAATAACTCG

ACCCCTTCCTCCTATAGGTAGTTTTAGACAAGTTTCCTTTGAAGTGGATACCTGAATGCC

AAGTATAGCTCGTAATAACCGATCTTCCGGGGCATACGACGATTCTTTCGCTGTCTGAGG

TGTTAATTTACCTACTAAAATATCGCCTGTCTCTACCCAAGACCCCAGCATCACAATTCC

ATTTCTGTCTAAATTTCGGAGTAAATGAGTTTCTAAATGCGGTATTTCCTTAGTAATTCT

TTCAGGACCTTGGCTTGTTACATGAGTCTGAATTTCATATTTCCGTATATGAAAAGAAGT

ATAAATATCTTCATATACCAGACGTTCACTAATGAGTACTGCATCTTCAAAATTGTAGCC

CTCCCATGGCATATACGCTACTAATACATTTTTTCCCAAAGCGAGTTCACCCCCAACAGT

AGCTGCACCATCCGCTAAAATTTGTCCCTTTTTAATGCACTTACCCCGCGAAACCGTAGT

TTTTTGATGCATACAAGTATTTTTGTTGGAACGTTGATACATAACCAATGGAATGGTTAT

AGTGTCTCCATTACCTGATAAAACGATCTTAGCTGTATTGGTATAAATGACCTTTCCTTC

GCGTTCGGATATAGCGGAAACCCCTGAATCTAGAGCCGCCTGTCGTTCCAACCCAGTTCC

CACAATGCACTTCTCGGACCGAGAAAGCGGAACTGCTTGGCGCTGCATATTCGAACTCAT

TAAAGCCCGATTCGCGTCATTATGCTCAATAAAAGGGATGAGGGAGGCTCCCATAGAAAA

GTATTGGAAGGGAAAAATGCTTCGAAGATGAATTTGTTCCCATGCAATAGTCAGGAATTC

TTGACGGTATCGGGCTGGAACAGCCTGTTCTTCCTGAATACCCCGATTTAGCGCCAAAGA

ATTTCCTGCCGCTACCATATGGTATTCATCTACACTTGGTGATAAATAAACCAACTGTAA

CCTTTTCGATCTCTCAGATATTTCATAAAATGGAGTCTTTATAGACCCCCAATGACCAAC

CCTTGCATGAATGGCTAAAGATCCAATAAGCCCAACATTGATTCCTTCGGACGTGTCAAT

TGGGCAAATACGCCCATAGTGACTAGTATGGATATCTCGTATCCGAAAGCTGGCGGTTCG

CCCTGTCAATCCTCCAGGACCCAAATAACTCGATTTTCGCCCATGAACTATTTGAGTCAA

TGGATTCGTTCGATCCAAAACTTGAGATAAAGGGTGTAGCCCGAAAAACGATTCATAAGT

GGTTGTTAATGGAGTTGAAGTTACCAAATTCTGAGGAGTCGGTATCAATTTATGCCGGAT

TGCTCCGCATATAGTTCCTCGAACCACATTTTCTAAACGAACCAGAGCCAATCCAAATTG

ATCCTGTAATAGATCTGCTACAGAACGAATACGTTTATTTTTCAAGTGGTTCATATCGTC

AAGTGTGCCCATTCCAAATTTCATGCCAATCAAATGATCCGCAGCGGCCAATACATCTCG

CGGTAACAAAAACGTATTATTCTGGGGTATATCAAGATTCAGCCTCCGGTTCATATTTCG

TCGACCAATCCTACCTAATTCACACCTTTGTTGAAAAAATTTCTTTTGTAATTCCCTACA

TAAAGACTCAGAAAATACCGGGTCCCCGCCTACACACGCAAATTGTTGATAAAACTCCAA

AATTGCATTTTCCCTTGACCCAATCTTTTTTTTCTCCCTTTCATTCAGGAAAGACAAGAA

AATTTCAGGGTAACGGACATTTTCTAGAATTTCTCTTAAATTCGAACCCATAGCCGATGA

TGAAACTAGAATAGATATTTTTTGTTTCCTACTCACACGAGCCCATATTCTTGCTTTTCT

ATCAATCTCTAATTCTGATCTTCCTCCCCAATCTGATATTATAGTCCCGGTATAGATAAA

AATTCCTTTATGGTCCAATTCTGAACGATAATAAATGCCGGGACTTTGCAATATTTGATT

GATCACAATTCTGTATATTCCATTTACTATGGAGATTCCCAAGGAATTCATTAGAGGAAT

ATTTCCAATGAATATGGTCTGTTCTTGCATATCTTTACCGGGCTTCCAAATCAATCCTGC

GGGTACATACAATTCAGAAGAATATGTGAGTGATTCATACACAGCATCTCTCTCTTTTAT

CAAAGGTTCTGCCAATTGATATGTTTCTACAAATAATTGAAATTCAATTTCTTGGTCTGT

ATCTTCAATTTTTGGAAACTTATGAAGTTCTTCGGCCAAGCCCCAATCAATGAACCTACA

AAATCCCTCAAATTGTATCTGGCTAAACCCTGGTATTGTAGACATTCCCTCATTTCTATC

CCGTAGCATCTTAATTCATTTTCCCGTTTTTCGAAAATCCCACTATTGGCCCACTCTTCA

TCGACCCATATGGGTCGACCGAGCAATCATGGAATGTATATTCTTTTTACTGAATCGCAT

GAAATTGTATAGAATTTCCTATATATGAAATACGTATTGGTATGTGTGTGTGGTAGGGGG

GGGAATAAAGAGAAATTTTCATATAAAATTGCAATTTGTGAAGGATACAAATAGGAATGA

ATTAATAAAACATTCCTGGAAACAAAACTCCATCACTTCAACTTATGGAGTCCTATTCAA

CTTATGGAGTCCTATATAGAAGATAAAAAAAAGATCCCATTTGTACCTATCATTAGGATA

TTATTATCAGGTTGGGTAGTTGAAATAGATTCAGGATTTGATCCATTCTCTAGGAATGAG

ATAAAGATAGGATAATAGAGTGTCGAATTGATTTTAACACTTAACTTATTTCATTAATTC

CATTGTTCAAAAAAAAATGGCAAAGAAAGGGGACATTTTTGTCTCTTTTGAAATTAGTAA

AATACGATTGAAGTGCGTAAGAAGAGTTTGATTTATACTTATTAATACATATATACATGT

GGCTATTTCCCTATCTGCATATCCCATCTTTTATCGCAATTCCATTTGCAGCAACAGAGG

TGATGCTATATCTTTTGATCAAATTTCAATTCAATTCATTCAAAAAAAAATATTGAATGC

AATATTCAACAAGCACGACTATTCACTATAAGATATAAGAGGGGATGCCCGGACAAGGCA

CCGACTGGATGTCCGTGTGATAATGTATGTTCTAGGGTTTACATAAACACATAATTGTTG

TTATAATTGAAATGGAAAAAGATTTCTTATTGAAAATAATTGATACGAATTAGTCGGATA

TCCGCTTTCTTTTCTTTTTTCTTTAAGAAAAAGAAAGAATGGCAAATCAAAATACAAAAG

CCGTTCATGAATTCTCGGTGAATAGTTCATAGTTCCCATTTTTCCTTATTTTTTTGATTC

TGTGATTGGGAATCCGCTTTTTTGAATAGAAAAAAGAAAGGAGAAGTTATTATTCGATTA

AGAATCGCTCAAATTCTGATTATATCAGATTATATTAAGTTAAGTGCTGTATGTCTGTTT

TGAAAGGCTATACAATCAAGAGATCCACTAGATCTAAAACAGAAGGATTTTGTCCTTTTT

GAAAAGGATAAAGAAACCGTAAGATCCAATTCAAATCAGAGAGAAAAGGAAGGGTTTAAT

AAATCCTCCAAAAGAAGATTTCCATCTATATCGATTTTGTTTGTATCGTTTTGGCGGCAT

GGCCGAGTGGTAAGGCGGGGGACTGCAAATCCCTTTTTCCCCAGTTCAAATCCGGGTGTC

GCCTGATCAACAAAAGATATAAAATCCCTTACCGCCAAAATAGAAGTAAAAAAAAATATT

GCCCGGCCCCGGCGAAAGTGTAGTCATATGCATATATAGGTATAAGGGCTGTTCATACTA

GATTTATACAATCTGGAGGTTCCATAAAAGACTTGCATTTTTTTTATGTTCTTATGAAAG

GCAACAAAACAAACAATGGATTTTTCCTACATGTTCCATTAATAACCATTTCCTTAAGAC

TTCCGAAATAGTTGTATATCTTCTTTATGCTTAATACTTTCTGATTCTACCAGAAATCAA

ATCACAAGAAATCTTGTTAAGAATACATTTTTTGTATTAGTTCATCAATTAGGTCAGACA

TTTTGACTCTTCCCCACAGATTTCGCTAAAATTAGTTATCAATAATGGAATAATTCCTTC

ATTTTCATAAAGATAGGGGACATAATTGGCATGGATATAGTAAGTCTCGCTTGGGCTGCT

TTAATGGTAGTCTTTACCTTTTCCCTTTCGCTTGTAGTATGGGGAAGAAGTGGACTCTAG

CGGCACTATTAATAAAGTTAAGTTGAATAATTGAACTGTATCAATTTGGAATAGATCGTT

ATGAGAAGGGTTTTGTTGTTTTTTTTTTAATTTTGATTTTCCAAAGAAATTTTATTGGAA

GACGGTAATATATGAATGAGAACCTTTAGATCAAACAAATATGGAAATAAAATAATTGGA

TTCCGATATTACTCGTATTTATAAACTTAAATTAATATTAATAATAGTAATAGTAAAAGT

AATACAAAGAATAGGAAATTTTTCCTACCGAAGTCTTCCTATTCCGAAATATTGCAAACT

ACTATGCATAAAAAGATGTTGCTGTGTTGGGTGAGCCGCGCCTATTTACCGTTTTTACTC

CTACGAATTTTTTTTTATGCTTTATTTTTTAACGCACAACTAATCTCATATCGTGGTTCA

AGCATGAAAAATTTGCGAGGTTTACTTCTGCTTTTCCAAACCCGAAGAAGTTATTATATA

ACCCCTTGGATCGTGTGTTAACAGCTCAATCGAATTACTGGAATGCGAAAAAAACTTTTT

TGGTAATATATAACCATACAACCCTTCCTTTCCTCATTGATTGTTTCGAGAGACCCCGAG

ATCCGTGCTGAGACTAATCAGAAATCTAGGCATTAGAGGAGTTGAACTTCGACGATTTCC

GATCGATCAAAATCAACACAATGGGTATATTGAAGAGATAGAATTGGGGTGTTGTTTCTA

TGTACATATCATATATGTAATATACATGTCAGTATATGTACCTAATTCGCATGTACATAT

ATCGAGACCATATCTTTATATAATATGACTGTATAGAATATACTAACAGATAGTGTAGTA

GAAAGGACTATATGAACTTTTCTACCACACTATCCATTAGTGTACTTAGATTGTTAGTTC

CGTCCTGCTTATTTCGTTTAAATTTCTTCTTTTATTCTTATGAGGAGCTAATAATTCAAA

GTTTGAGTCAAATTAATCATTTTGACTAACCGTTTTTACGTAGATGATAAGTAAAAAAGC

AGTAGGAACTAGAATGAATAGCACAGTAGCAATAAATGCGAGAATATTAACTTCCATAAT

CTCATCGGTTTTTGCTTCGCAATAACGCGGGATCTAATCCCATAGAAGAAAGTATTTCTT

CTGTAATATAAACGCAATAGGTGGATTGCATCTTGATGATAATGAATCGGATTCATATTA

CGAATAACAATATCTGATCTATTAAATGGATTCATCGTCGAAAATGGAATAGTATAACAT

AAGAGGACCTTTTATCCATATAAAAAGTGGAATCGCCAATCCAATCTCGAATCCCATCGA

ATTTCTCCTATTTTTCCACTCTATACTTTGATCTTTATTTCTTAATTTTATAGAAACGAC

CAATCGCGGTCCTTATAACAATCATCTAATCTAAGACGGAGTGCCGTCTGACCGGTGCGA

TATTCCATTGGCCGCATACCTAAATAGTAGGAGTCAAATGGAAAAGGGACGAGTTCGAAA

CAAAGTGTTTTTTTTCGTGGTCCAATCCTCTTAGATAACCGATAAATGTAATAAAGGCGG

ATTACAGGTATAAAAAATCGAATATTCCGACAAATTCACTATTTTAGTTTAGGTTTTAGT

TAGTAACTTTTTTCTTCCTTGGATTGGAACTGGGATACGTACACCCAACTTCGACATGCA

ATTTGGATGAGATAGATAGATTGTTTTCAATTATTAATTAAGTAAGGTTCCACAAAAAAT

TGGAACTAGAAGCTAGAAAAGGGGTTTTCGTCGAAAAAATGTCCCAGTCGGGTAACTCCA

TGAATTTTATTGTGTATCGTAACAAATACCGTTTTTTTGATATGTACCCTTGGAAGCATA

TGGATACTTTATTCTATTTCATTGATCGGGAGTAATCAAAAAAGTGAAACTGGTCATGAG

TACGGCGATACCTCCAACTACACATGTCTCTTCCCCGTCAATCAATATTAAAGTAATTGA

ACTTCTTTGTTTGATTCGAATTCGAATCAAACAAAGAAGTTCAGGATCCTTCAGGTGACA

GGATCCCGCCTTGCGCCCCCTCTCTTCACAGAAAATAGAGAAAAAAAAATGGATGGATTC

ACCGCGCCGGATCCGATGTCGGGACTGACGGGGCTCGAACCCGCAGCTTCCGCCTTGACA

GGGCGGTGCTCTGACCGATTGAACTACAATCCCAGAATCCCGGGGTGAGTTTTACAGGAT

ATCTACTATTTTTTTTTAATTTCATTTGAATCATTTATATTTCAAATAGTTTTTCTTATA

ATAAAGACAAATAGTTTTTCTTATAATAAAGACAAAGACGGCTATTTGAAATACTGCACA

TGGATAGAAACTAGAGTGAGAATGACAGGATTCCTAGGAATCCTGTGGTTATTACCAATA

ACCAGATCCAAACACGGTAGAAATCAAAAAAAAGGGGATGTTACAGACAAATTTTGTCCC

CTTTTGGCTATCGGGCACTTCTAGAGGACAAATCTATTTGGTTACATCATTCTTATGGGA

ATGGCGAATTAGTGGGCCGAGCTGGATTTGAACCAGCGTAGACATATCGTCAACGAATTT

ACAGTCCGTCCCCATTAACCGCTCGGGCATCGACCCCGGAAGAAGAAATTCGAGGTTTAT

TGATAAAACACGATTTACTTCTTTTCGTGGAACCTTACCCCCAGGGGAAGTCGAATCCCC

GCTGCCTCCTTGAAAGAGAGATGTCCTGACCACTAGACGATAGGGGCATACCTGCCCGAC

CGCCATCATACTACGATCATAGTATGCTCAGTTTTTTGGAATTGTCAATATAATGAATCG

ATATTTGTCATTTATGAACATCATATAACAAAGTTATATGGTCTTTTAGGGATTGATTTG

TCCGACAGACAGAAAAAGATGAAATGTCAAATTCTATTTTATTTATTCCATTTTAACTCA

TTAATTTTAATTAACTCATCACTCGGATTGAATATGCTGATTTCTATCTCACTCTTAAGA

CGGAAAATTCATTCAAAAACGATAGCATTTTTGTTTTGATTAATTCAAAATCATTATGTA

GAAATCTAGGAAGGGATTCAATGAAATATATTCTTGGATCTATGTTAGATTAGTACACGT

ATAAATAAGTAAATCTCTTCTTGATAGGATAGAGGAATAAAAGAAATTAGGATGGCCGTG

AAAGAATTCATAGCACGGCTGCTATTAAAAAAATCCGAATTGGATTACTTTTTTTTACTA

TAGAACTTGAACTTCCGGGGTAAATTCAATTTATTATTATTTTCCTGAATGATCCCCTAT

TCATACATGTATCTATATCTTACTGTATCTATATATATAGATATATATATAATATATATA

GTATCTACATAGGTAATGCAGTAGACTCATAACAACCAATGGCTTTTTCTAAATTTTAGA

TAATGGGCCCTTTTAACTCAGCGGTAGAGTAACGCCATGGTAAGGCGTAAGTCGTCGGTT

CAAATCCGATAAAGGGCTTTTCAAAAAAATTCCGCGGTCCAAAATCTTAGACTTTTTAGC

AGATAATACAAAATACATTTTTTATCTTTATTCCCGCTTATCTTTCCTAAAGAAAATCAC

TTATTTTAGAAAAAATAAGCCCGCATGATGAATTTTTCAGTCGAATGAATTCAAGTTGAA

CGTTTATTCATTCAAACGAAATGTTTGTTCATTAAGATGGGTTCATTGGGATTCATTAGG

ATAAGATAATAAGTAATCACATTAATTAGAATTCGATTAAGAGGATTGCTATGTCACTAC

AGGTTATACTTCTCCCCATTTTTCGTTTCATTATTATTATTATCCTATTCCTATTTTCTT

TTAGCCCTGGTACATCGACCTATTATGGATACCCGACCGGGGTTATGAACCACTAAACAA

AAGTCTTCCTACTTCTCAATAAAACATATCGGAAAAATTCGAAATCAAGAAAAGAAAAAG

TAAGTGGACCTGACCCATTGAATCATGAATATATCATCTATTCTGATATTAAAAAATAGT

CGATACAGATAAAGTTGTCGAAAGCGGGCGAGCTTTTTTAGTTCCTTGGACCGCGCACGA

ATTTGTCGATATTTCCGATTGAATCCTCTTCTCTTGTTTGTTCATAAATGCTCCATAGGA

ATAAATCACTATTCCTTTCTTCCACCGAAAAGTATTTCTTTCGAATCACAAATCTATTTC

AATATCTTTCTTTTTTTATGATTCCATAACATAAAAAAGGACCCATTTCTATCTATATAG

GATTTCGATATGGATATCAGATCATCGCTCCATTTCATGTGCCTAAAATTTCCACATCGA

TATATCAGATATTTTTGTTCCACCAATGCAACGGAGAACGAATACGAGAAAACAACTTTC

ATTTTAAACCCCAATTTCCTATTTTATTTAATTTCGATTTCTTTTAGAGATAAGAACAAA

AATAAAGAGCGAATTTTTTTTCTTCTGACGGATAAAGGGAAAGGGGAAAGGGGGAAATTT

TCGAAATTTCTTTTTTTGTTCCGCCCTCAAAAAAAGGTATACTCTGGGGTTTTCGATTTA

TCCGAAGGAAATAGAACTTCGGAAAAAGGAGACAATAACAAACAAAAAACAATCCAATAA

AGAAAAGTAATGTTATATAGGGTAGGGTACTGTAGTAGTTTTAGTGATAGTTATATATAA

ATAATAAATCTATGTTGTTATATATAAATATAACTATCTATGTTATATACTGTAGTATTT

TACTATACACAAAACACAAAATGAAATTGGGAAAATCCATAGAGATTTTTATGGATCCTT

TCTCAATATCACGGATAAGATCCAAGTATCATAATACTATACTTGATCGAACGAGAAGAT

AGCTCTATCAACTAGTGGGGCTCGTTCGCTCTTCCACAGTGATTTCAAAAGGAATCATCT

GATTGATGATTCATAAGACAATTCTGGGTTCGGGTGGCTATTAAGAAGAGGAAGAAATAG

TCGAATCTGGCTCTTACCTATAGGTCAGTTTGATTTGTAGCCCCATACCGCGGGGGGGTT

TGTATCTTCGATACCCATTCATGGAAAAGGGTATCGACGACTCTCTGTCCATGTTTATAG

ATGATGGAACCGTCGTATGTATGCCCTGAGTATCTTATGAGGTGTTCGGAAATGGTTGAA

GTAGTTGAATAGGAGGATCACTATGACTATAGCCCTTGGTAGATTTACCAAAGAAGAAGA

AAAGGATTTATTTGATATTATGGATGACTGGTTACGAAGAGATCGTTTCGTTTTTGTAGG

TTGGTCTGGTCTATTGCTCTTTCCTTGTGCTTATTTCGCTTTAGGGGGTTGGTTCACGGG

TACAACCTTTGTAACTTCATGGTATACCCATGGGTTGGCGAGTTCCTATTTGGAAGGCTG

CAATTTCTTAACCGCTGCAGTTTCTACTCCTGCTAATAGTTTAGCACACTCCTTATTGTT

ACTATGGGGCCCTGAGTCGCAGGGAGATTTTACCCGTTGGTGTCAATTAGGCGGTTTGTG

GACTTTTGTTGCTCTTCATGGTGCTTTCGGACTAATTGGTTTCATGTTACGTCAATTCGA

ACTTGCTCGATCTGTTCAATTGCGACCCTATAATGCAATCGCATTCTCTGCTCCAATTGC

GGTTTTTGTTTCTGTATTCTTGATTTATCCACTAGGCCAGTCTGGTTGGTTCTTTGCACC

TAGTTTTGGAGTTGCTGCTATATTTCGATTCATCCTCTTCTTCCAAGGGTTTCATAATTG

GACCTTGAACCCATTTCATATGATGGGAGTTGCCGGAGTATTGGGCGCTGCTCTACTATG

TGCTATTCATGGTGCTACCGTCGAAAATACTTTATTCGAAGATGGTGACGGTGCAAATAC

ATTCCGTGCTTTTAACCCAACTCAAGCCGAAGAGACTTATTCGATGGTCACTGCTAACCG

CTTTTGGTCTCAAATTTTTGGGGTTGCTTTTTCCAATAAACGTTGGTTACATTTCTTTAT

GTTATTTGTACCAGTAACCGGTTTATGGATGAGCGCTCTTGGAGTAGTCGGTTTGGCTCT

GAATCTACGTGCCTATGACTTTGTTTCCCAGGAAATCCGTGCAGCGGAAGATCCTGAATT

TGAGACTTTTTACACCAAAAATATTCTTTTAAATGAAGGTATTCGTGCTTGGATGGCGGC

TCAGGATCAGCCTCATGAAAACCTTGTATTCCCTGAGGAGGTTCTACCCCGTGGAAACGC

TCTTTAATGGAACTTTAGCTTTAACCGGTCGTGACCAAGAAACTACTGGTTTCGCTTGGT

GGGCTGGTAATGCCCGACTTATCAATTTATCCGGTAAACTACTTGGGGCTCACGTAGCCC

ATGCTGGATTAATCGTATTCTGGGCCGGAGCAATGAACCTATTTGAAGTGGCTCATTTCG

TACCAGAGAAACCCATGTATGAACAAGGATTAATTTTACTTCCACATCTAGCTACTCTAG

GTTGGGGCGTAGGTCCGGGTGGGGAAGTTATCGACACCTTTCCATACTTTGTATCTGGAG

TACTTCACTTAATTTCTTCCGCAGTCTTAGGCTTTGGTGGTATTTACCATGCACTTCTAG

GACCTGAGACTCTGGAAGAATCTTTTCCATTCTTCGGTTATGTATGGAAAGATAGAAATA

AAATGACCACAATTTTGGGTATTCACTTAATTTTGTTAGGTATAGGTGCATTTCTTCTAG

TATTTAAAGCTCTTTATTTTGGAGGCGTATATGACACCTGGGCTCCCGGGGGGGGAGATG

TAAGAAAAATTACCAACCTGACCCTTAGCCCAAGTGTTATATTTGGTTATTTACTAAAAT

CTCCCTTTGGAGGAGAGGGTTGGATTGTTAGTGTAGACGACTTAGAAGATATAACTGGGG

GACATGTATGGTTAGGTTCCATTTGTATACTTGGTGGGATCTGGCATATCTTAACCAAAC

CCTTTGCATGGGCTCGTCGAGCATTTGTATGGTCTGGGGAGGCTTACTTGTCTTATAGTT

TAGGTGCCTTATCTATCTTTGGTTTCGTCGCTTGCTGCTTTGTATGGTTCAATAATACCG

CTTACCCTAGTGAGTTTTATGGTCCTACTGGCCCAGAAGCTTCTCAAGCTCAAGCATTTA

CCTTTCTAGTTAGAGACCAGCGTCTTGGGGCTAACGTGGGGTCTGCTCAAGGGCCCACTG

GTTTAGGTAAATATTTAATGCGTTCCCCAACGGGAGAGGTTATTTTTGGAGGAGAAACTA

TGCGTTTTTGGGATCTCCGTGCTCCTTGGTTGGAACCTTTAAGAGGGCCCAATGGTTTGG

ACTTGGGTCGGCTGAAAAAGGACATACAACCTTGGCAAGAACGACGTTCCGCAGAGTATA

TGACTCATGCTCCTTTAGGTTCTTTAAATTCCGTGGGTGGCGTAGCTACCGAGATCAATG

CAGTCAATTACGTCTCTCCTAGAAGTTGGTTAGCTACCTCTCATTTTGTTCTAGGATTCT

TCTTTTTCGTGGGCCATTTGTGGCATGCGGGAAGGGCCCGTGCGGCTGCGGCGGGATTTG

AAAAAGGAATCGATCGCGATTTTGAACCCGTTCTTTCCATGACCCCTCTTAACTGAGACG

GGTGATCCAATGAATGAAGCAGGAATAATTGATTTGATTCCATCTTAACTTAACAAGTAA

AGGATTCCTTTTCCTTTCTTTTTTATTTTTTTTTTTCAAAAGAAAAATTTAGATATAATA

TCTATTTATTCTATTATTTATTCTATTTAAATACTTGTTTTTCTAGCTCGGCTATTCCAC

CTAGCCGAGCTATTCTGCTTTATAACAATGGCTAGACCAAACGAATAAAAAGCCAATATT

TTCAACGAGTAAAAGGAGAGAGAGGGATTCGAACCCTCGACAGTTCTTTGTTCAGAACTA

TACCGGTTTTCAAGACCGGAGCTATCAACCACTCGGCCATCTCTCCGAGAGTAAATTTCT

ATTTTATTCCCATGAATAGAACATGGCCATATGAGTTGATACCATAACTATCTGTAGAAA

CCACACCCGGGGTGTGAATTTTGATTTTTATCGAGTCTCTCCAGATAGATGCATAAGCCG

AGCCGACATATCTGTTTGCGAAGTAAATCCAAAGAGCCTTTTTTAACTCGATGTACAAAC

AAAAAGAAAAAATACGAAATTCAATCAAACAAATAGGAATAAGTTTGAAAGGGCCAATAT

ATTTAGGGTCAAATCCATCATAGAAAGATTTGATTCCAATTGATTTGGACACAGAGAGGG

ATCAAATGGTATAGTTCATTTCTTGGTAACTTGGAGGATTACAAGCATGACTATTGCTTT

CCAATTGGCTGTTTTTGCATTAATTGCAACTTCGTTAATCTTAGTGATTGGTGTACCCGT

TGTATTTGCTTCTCCTGATGGTTGGTCAAGTAATAAAAATATTGTATTTTCAGGTACATC

GTTGTGGATTGGATTAGTTTTTCTAGTGGGTATCCTTAATTCTCTCATCTCTTGAACCTC

TTCGTTGAGAATCAAAAAAACAGACCCCCAAAATAACATTTTTTATTTTATTTATTTTGG

GGGGCTCATATTGAATTGAAATGTGTCTCACAATCCGATTCTTTCGTTCCTTTTTGGGAA

AGGTCCAAACCAAAGCCGCTTCTTGAATAAAAAAAATGTTTAAAAATTCTAATTATAGAA

ATCCTTCAGATTTTGGCTCTGCATAAATGGGTCAAGATATGTGTATATCTATATAAATAT

ATAATATATATATGGACATAATCCCAGCCTGTTAGGAACAAAAATGCGGATATGGTCGAA

TGGTAAAATTTCTCTTTGCCAAGGAGAAGATGCGGGTTCGATTCCCGCTATCCGCCCATT

CTCTTGTTATATGACAGATAATTGGTATATAGTTGACCATAATAGTGTAGTGGTTCTATC

TTCCTTTTTCCTACCATCCGCAAAAATCAGAATTTTTTACGAAAAAGGGAGTGATACTGA

TAAAGAATTCTGCCGAAAAAAAATGTATTTTGCCAAAAAATGTTGCGGAGACAGGATTTG

AACCTGTGACCTCAAGGTTATGAGCCTTGCGAGCTACCAAACTGCTCTACCCCGCGAAAA

CAAAAGAATTTCGAACTAATAGATAAACAAGGATTGGATGCGCCCCGTTACTATTCTATA

GAAATAGGATATCCTATTTCTATAGAATACTATTTCTATAGAATAGTAAAGGGGTCACTC

TATGATCTATGATCATAGGGATCAATAGAAATATGACGAAGTCGTTTTTTTATCCTTACC

AACTTGATCTTGTTGCCCCAGGTAACAAACATGCATGAAACCTTTCGCGAAGTATGTGTC

CGGATAGCCCAAAATCCCGATAGTTACCTCTAGGCCTTCCGGTCAAAAAACAACGTCGAT

GAAGGCGTATAGGTGCACTATTGCGCGGGGGGGATTGCAATTTTCCATGAATTTCCCATT

TGCCACTCAACGACGAAACCTTGCTTATTTCTTTTTTTGAGGATCGGCGAATCGAATGAA

ATTTTTGTTCTAATTTCTGCCTCTTCCTTTCCCTCTGAATCAAACTTTTCCTTGCCATAA

CATAATGGTGCAATTTTGATTATCAATTATACGGGTCGAATCCTAGATGTAGAAATAGAC

GAAGTTAGGGCCCCCCTCTCCATCGAATCAAATGAAATTTTCGCTGATAGAATAGATTAA

TTAAAAGACAACTGAATTAACCAAATTTGCCTGATGTAGAAGCAATCAAGAAAGCTGCAT

AGGTGAATATATAACCTACCGAAAAATGGGCTAATCCGACCAACCTTGCTTGCACAATGG

AAAGAGCTACAGGTTTATCCCTCCATCGAATCAAATTGGCTAAAGGTGTACGCTCATGAG

CCCATGCTAAAGTTTCAATCAATTCCTGCCAATATCCACGCCAGGAAATTAAGAACATAA

ATCCAATAGCCCAAACAAGATGACCAAATAGGAACATCCATGCCCAGACCGATAAACTAT

TCATACCAAAAGGGTTATATCCATTGATAAGTTGTGAAGAGTTTAACCATAAATAATCTC

TTAACCATCCCATCAAATAAGTGGAGGATTCATTAAATTGTGAAACATTACCCTGCCATA

ATGTGATGTGCTTCCAATGCCAATAAAAAGTGACCCATCCAATGGTATTTAACATCCAGA

AAACTGCCAAATAAAATGCGTCCCAAGCCGAAATATCACAAGTACCGCCTCGCCCCGGCC

CGTCGCAAGGAAAACTATAACCAAAATCCTTTTTATCTGGCATTAACTTGGAACCGCGCG

CATCTAAAGCACCCTTTACTAAAATCAATGTAGTTGTATGCAAGCCTAGAGCAATAGCAT

GATGAACCAAGAAGTCTCCGGGGCCTATTGTTAAGAATAGTGAATTACTATTCTCATTAA

CGGCACTCAACCAGCCCGGTAACCATATGCTTCGACCAGCATTAAATGCTGGACCGCTCG

TTGAAGATAAGAGTATATCGAACCCATATGAAGTCTTACCATGAGCAGATTGTATCCATT

GGGCAAATATGGGCTCAATCAAAATTTGTTTCTCCGGAGTACCAAAAGCGAGCATGACGT

CGTTATGAACGTAGAGTCCTAAGGTATGGAACCCCAAAAATAGGCTAGCCCAACTTAAAT

GAGATATGATAGCTTCTTTATGGTCTAACATTCTTGCCAATACATTATCCTCATTCTGTT

CCGGATTGTAATCCCGAATGAAGAATATAGCTCCATGAGCAAAGGCTCCTGTCATGATAA

ACCCTGCGATATACTGGTGATGAGTATATAACGCAGCTTGGCTAGTAAAGTCTTGTGCTA

TGAATGCATAAGCGGGTAAAGAATACATGTGTTGAGCTACCAAGGAGGTAATAACCCCTA

AAGAGGCTAGAGCAAGACCCAATTGAAAATGAAGCGAATTATTGATTGTGTCATAAAGAC

CCTTATGTCCACGTCCCAATCGGCCCCCTGGAGGAGTATGTGCTTCTAAAAGATCTTTCA

TACTGTGCCCAATTCCGAAGTTAGTTCTATACATATGACCGGCTACGAGAAAAATAAATG

CAATAGCTAAATGATGATGAGCAATGTCGGTCAACCATAAACTTTGCGTTTGTGGATGGA

ATCCTCCGAGAAGGGTTAGAATGGCAGTTCCTGCTCCTTGGGAGGTACCAAATAAATGAC

TACCGGAATCGGGGTTCTGGGCATAAAGATTCCACTGGCCTGTAAAAAGTGGGGTCAACC

CTTGGGGATACGGTAATACATCTAAGAAATTATTCCATCGGACGTACTCCCCCCTCGATC

CAGGAATGGCGACATGCACTAAATGCCCTGTCCAAGCCAAAGAACTTACTCCAAAAAGTC

CTGACAAATGATGATTGAGACGAGATTCCGCATTTTTGAACCACGAAACGCTCGGTTTCC

ATTTTGGTTGTAGGTGTAACCAACCCGCCACTAAGGATATAGCAGAAAGAAATAATAGAA

AAAGAGCTCCAGTATAAAGATCCTCATTGGTGCGTAAGCCGATTGTATACCACCACTGAT

AAACGCCGGAATAAGAGATATTCACCGGGCCGAGAGCACCTCCTCGAGTAAAGGCTTCGA

CAGCCGGTTGACCAAAATGAGGATCCCAAATTGCATGAGCAATAGGTCTTACATGTAAAG

GGTCCTTTACCCATGACTCAAAATTTCCTTGCCAAGCTACATGAAACAGATTTCCGGAAG

TCCACAGAAAGATTATTGCTAACTGCCCGAAGTGGGAAGCAAAAATGTTCTGATAAAGAC

GTTCCTCAGTAATATCATCATGACTTTCGAAATCGTGTGCGGTAGCAATACCAAACCAAA

TACGACGAGTAGTGGGGTCCTGAGCTAAGCCTTGGCTAAACCTTGGAAATCGTAATGCCA

TAATGCCTTTCAAATCCTCCTATCCTAGCCATTATCCTACTGCAATAATTCTTGCTAAGA

AGAATGCCCATGTTGTGGCAATTCCACCCAGAAGGTAATGGGTTACTCCTACAGCACGTC

CTTGTACAATGCTCAAGGCTCTAGGCTGAGTAGCAGGAGCAACTTTTAATTTGTTATGAG

CCCAGACGATGGATTCAATGAGTTCTTGCCAATAACCGCGGCCGCTGAATAGAAACATTA

AACTAAAGGCCCAGACAAAATGAGCACCTAGGAAAAAAAGACCATATGCAGATAATGAAG

AACCATAAGATTGAATTACCTGAGATGCCTGTGCCCATAAGAAGTCCCGGAGCCACCCAT

TAATAGTAATGGAACTCTGTGCAAAGTTTCCTCCCGTGATGTGAGTTACCACCCCTTGAT

CACTTATGCTACCCCAAACATCTGACTGCATTTTCCAACTGAAATGGAATATGACTACCG

AAACCGCATTGTACATCCAGAATAGGCCTAAGAAAACATGATCCCAGGCGGATACTTGGC

ATGTCCCGCCTCTTCCGGGCCCGTCACAAGGGAAACGAAAACCAAGATTTGCTTTATCAG

GTATCAACCGCGAGCTACGAGCAAATAGAACGCCTTTCAGTAATATCAATACAGTCACGT

GGATCGTAAATGCATGAATGTGATGGACCAAAAAATCTGCAGTTCCTAATGGAATAGGTA

ACAAAGCCACCTTGCCACCTACTGCCACTAAATCAGCACCTCCCCACGTCAAGCTGGTGC

TTGCTGTTGCACCGGGAGCTGTTGCACTCGGCGCTAAAGCATGGGTATTTTGTATCCATT

GAGCAAAGATAGGTTGTAATTGTATAGCGGTATCTGAAAACATATCTTGGGGACGCCCTA

AAGCACTCATAGTATCATTATGAATATACAAGCCAAAACTGTGAAAGCCTAGAAATATAC

ATGCCCAGTTAAGATGGGATATGATTGCATCACGGTGCCTAAGGACACGATCCAATAGAT

CATTATAGCGAGTAGTTGGATCGTAGTCTCTTACCATAAAAATGGCTGCATGCGCGGCAG

CACCAACTATGAGAAATCCCCCAATCCACATGTGATGTGTGAACAACGAAAGTTGTGTAC

CGTAGTCAGTAGCTAGGTATGGATAGGGGGGCATGGAATACATATGGTGAGCTACAACAA

TGGTCAAGGAGCCTAACATAGCTAGGTTAAGGGATAATTGAGCATGCCATGATGTTGTTA

GAATCTCATAGAGGCCTTTATGACCCTGACCCGTAAATGGACCTTTATGAGCCTCTAAAA

TATCTTTTAGACTGTGACCAATGCCCCAGTTAGTCCTATACATGTGACCTGCTATCAGGA

AAAGAATTGCAATAGCTAAATGATGGTATGCAATATCGGTTAGCCATAGGCCTCCTGTTG

TTGGATCTAATCCTCCACGAAAAGTAAGAAATTCCGCATATTTTGACCAATTCAAGGTAA

AAAATGGGGTTGCTCCCTCGGCAAAACTGGGATAAAGTTGAGCCAAAAGATCCCGATTCA

AGATAAATTCATGAGGAAGTGGTATCTCTTTAGGATCTACCCCAGCATCTAGAAATTGGT

TAATTGGTAAAGATACATGTACTTGGTGTCCCGCCCAAGCAAGAGACCCCAGTCCTAGTA

ACCCCGCCAAGTGGTGATTCAACATGGATTCTACATCTTGGAACCAAGACAATTTTGGAG

CAGCTTTGTGATAATGGAACCAACCGGCAAAAAGCATTAATCCTGCAAAGACCAATGCAC

CAATTGCGGTACAATAAAGTTGTAATTCGTTAGTTATTCCAGATGCGCGCCAAATTTGAA

AAAACCCGGAAGTTATTTGTATTCCTCGAAAACCTCCGCCCACATCACCATTTAATATCT

CTTGACCCACTATTGGCCAAACTACCTGCGCGCTGGGCCCAATGTGAGTAGGATCGCTCA

GCCACGCTTCATAATTGGAAAAACGAGCACCATGGAAATACATTCCACTCAGCCAAAGGA

AGATGATGGAGAGTTGACCGAAATGAGCACTAAATACTTTTCGAGAGATCTCCTCCAAAT

CACTGGTATGGCTATCAAAATCGTGAGCATCCGCATGTAAGTTCCAGATCCAAGTGGTAG

TATCCGGGCCCTTAGCTATTGTTCTTGAAAAATGGCCGGGTTTGGCCCATTCCTCGAAAG

AAGTTTTTACGGGATCCCTATCCACCACAATCTTCACTTCTGATTCCGGCGAACGAATAA

TCATTGAGTCCTCCTCTTTCCGGACAACACATACAAAGAGACCCGCCAATAGTACAGTTA

AGTAATTAGTGAACCTATGAATCTGAAAGATAGTATTTCTATTTATGATTAGTTCCTTTC

TTTTCTATTTATGATGAATTTCCTCTTTTCCTATCTCCCATCTATCTATTTTCTTTAATT

ATTAACTAGAGCAATTAGGATATGGAAGTCGATCCAGGGCAAGTGTTCGTATCTATTATG

ACATAGCCATAGGGCGCTCAACGGACACTTTAAATCTTGTCGGCATGATACAAAAACCAT

TTTTTATGCAACCTAGCTTATAAAATAAATAGCTAGGTTAGTCTACTTCATATTTAAATG

TAGATATCTAGATGTCTCTTATTAATAACTAACGAATGATCCTTTGATTCGGAATAGATT

AATACGTTCCATATAAAATAAATCATTCGCGTTTGGATTGGATAGTATACAAGAAAAGAA

TCGATTCTGTACAGTATCTGTATCATTTATACTTAGAAAATTTCAGACGAAATAGAATGA

GAAGGGATATTATGAAATTCTTTGATTTTTTCTTCCCAGAGGAACGATCTATTTGATTTT

ATTGAATTGATCAATCCATATGCTAATGCAATTCAAAAAGAAAGTCTTTTTATTCGAAGC

GCCCTGTAATCTTTAACCAATTATGTGCTTCAATATAATTACCTGGAGTAAGCGATATAG

CCTGTTTCCAATATTCAGCAGCTTGATCGGACCAAGCCTCTGCAATTTCAGAATCTCCCT

GTCGAATGGCCTGTTCTCCCCGGTTGGAATAGGTAGTTCCTTCCCTTCGAACCGTACTTG

AGAGTTTCCTAACTCATACGGCTCGCTCCGCAGTCTTTTGGTTTTAGTGTCCCAGATTAA

TCTACCATATCATATCTTTAACAGAATAGGATTTACGAGAAATCCATCTGATTTTATATT

GGGTTAACCAGAAGAAATTAATTACATAAGTTTCAAACTCTCATTTTGATCAAAAATCTG

TTTTTTCTTTTTTCCTATCTTCAGAAGAATAAAGAACAGCTACCCCTTTTTATTGTTATA

ATCTTCTGAAAGGTAACTATCTCGATTTCATATAGAAATTCGTATAGAATCTTTGAAAAA

GACTTTTCTCTAAAAAAAAGAAAGGACTTACTATCTTTGGGATCTGATGCTACACCGCTG

CTCAATACCTTAGTAGATCGACTCTATTACATAAGTTGATTCCTAACTTTTATATCATAT

CATGACATAAGTAAGCAGTTCTTATTGTATCGGCCCGAAAACAAATTTCTCTAATTGATC

TTTACGGTGTTTCTTCTCTCAATTAGATCCTTTATCCATAGAATCTAGTATATAGGCTGT

ACCCATTTATTTCGGCTCCTACGAAGTCTGTTTTTTTGCTACAGCTGATAAAAATCGTTG

CTTTAGACGATACATATGTAGAAAGCCTATTTTTGCTAGTAAATACTAGCTTGATCTTTC

TTCCCTTCTTTCTATAGTGGAGATAGCCGCGCGTAATGACAGATCACGGCCATATTATTA

AAAGCTTGCGGTAAGAATGGGTTTCGCTCTAGTGCCCGGAAATAATATTCCAAAGCTTTC

GTATGCTCCCCGTTGCTTGTGTGGATAAGTCCTATGTTATAGAGTATATAACTTCGATCA

TAGGGATCAATTTCTAGTCGCGTAGCTTCATAATAATTTTGTAAAGCTTCCGCATAATTT

CCTTCGGATTGAGCCGACATCCGTTACGGTCGTTTATTTTTTTTATTCAATGAATCTCCG

TTCCAAAACCGTACGTGAGACTTTCATCTCATACGGCTCCCCCCTTCTGTGCATAGTAAT

AAAGGGAATAATCCATACTATGTAATTCAAAATCAAAGGGGTTGGAATATTCTCATTATG

AACTGACGGGGGCTGGTGTTTTTACAAGAAATCTCTAGCCAGCCTTCCTGCAAGAGGTCC

ATCTTTTGTGTTAAGAAAAGATGTCGATTCTAGATAGAAATAAATGGTAACTCAAACAAT

TTCTTTGTCCTCAACGCCTTCTATTTCAGGAATTCGTCACTTCAACGATCTTCGGTGATT

ATACGGGTATCCAAAATACGAACGAGATGGATGTTTGTTGTCCCAACCACTCTTAGTAGT

CCCGATCCCAATAAAATGAGGAAAGGGCTAATTTATAACAAAGGTTTCGTGTTGTTGATT

CCTGGGTATAGTGTAGTGCTTCTTCCTTTATGCGACCTATTAGTTAGTACTAGTAAAGTA

GGAGTGACCTGCAATACATAAAGAACCACTAGGTTTAACCTTTCGCTCAATACTAGAATC

GACAATTGAAGCATCTGAGACTGCATCAATCGTGGATACACGACAGAAGGAATTGTTCTA

TCTCCAAACTTCACCTTCACCAAGCGTAGATTTATTTCAAGAATTCTTTTCTTTATCCCT

AATCATATCTCTTTCTTGTGGGTATAAGGATGAATGAGGGTGGTAAAGTACAAGTAAATA

AGAAATTCTATACACTATACATAATTAATATAAATATAAAAAGAGTCAAATCGCACCATC

TCTATAATAGGTAAATGCCTCTTTTTCTCCTGAAGTTGTCGGAATTATTCGTAATAAGAT

ATTAGCTACAATTGAAAAGGTCTTATCAATAAAATTTCCATTTATCTGAGATCTAGGCAT

AGTTTGCAACCCATTCTATAAATTCTTCTCATTTTATTATCCCCCTCGAGGGAAAATGAT

TTCACAAACAAAGAAATTGTACAGTACGAAATCACATAAAAAGAAAAACAAACAAATTCT

AAAAGAAAACGAATTCGAACAAAAAAGATGTTGACCTTCCACCCTAATTGTCCCAAAGGG

GCAGGGATAGATAGGAAATATTGAAATCCGATTGACTGGGGTTCATTCCAATTATACCAA

TAAACGTAACGCTTACTATTTTAGATAAGTTAGATAATAGCTATCCATTTGGATTGAATT

GAATGTATACACCAATGGGAATAAAAAAATCACAGATGACTCATTAATTTGTAATAGATC

TATGATATGGGGTAGCTCGTGAAAAGAGTTGTTGTTGAGAAACCGAAAATAACAAAGCAA

AGGAGTTTTCTAATTTCTATAGAACCATAGTCTAAGTCTAACTAAACTATAAGTCGAGTC

TAAACGTAATTAGAATAACATAATCATAGCATAAAATGGGGTTATTTGATTCATTTCAAT

TCATTGGCTTAATCTAGTATAAATATAGTAAGAAAATGATGGATCTTTGTCTTGACAAAA

TAGATATATATATTTTTGATTGGTAATATCTTTACAATCGAAAATGGTATTTTTTTCCTT

CCCTAGAAGTTGACTTTGTTTGATGAAAAGTTTTCGATTTCGAATTGATTGGCCGTGTCT

GTATTGCAATAATTCAAAAGAATATGAATAACTCGCTATTCAATCGGTTTCTGGGCCATA

ATCCTAAGATTATATAGGAAAGGTGGCCGAGTGGTTCAAGGCGTAGCACTGGAACTGCTA

TGTAGACTTTTGTTTACCGAGGGTTCGAATCCCTCTCTTTCCGAATGAATCTTATCTTAT

TCTAATTCACCAACAGTAGCGGTATCGACCACAATCTATCTAATAGCAATCGATACCATT

CTATCCAACGGTAATTCTAGAGATTCTCTATTCCTTTCCTAAGCACTTTGGTTGGTATCG

GAAATTTCGAAAAAAAAAAGAATAGACAAGGGGTGAGAATCTTACCGCTAGTTGTAATAC

AAGAATCGTAGAAAAATACGGACTTAATCCACTCTACTTCGGAAAAGGGGATCCAAATTG

TCCGAAGATTTGTGTTCTTTTTATTTGATTAGGATCAAGTCTGACGTGAATAATATTCCA

CGACTAGCAACTCATTGATTTTTAAACCGACCCATTTACTATCTATTATTTGATTTACTA

CCCCTTTATATTGGGACGAGTCAAGAGTCAAATGTTTTGGCAATTCTTCGCGGGAAGCAG

AATCCATAGAATTTTGAACCAGAACTTTGGATCTTTGTTCATTTCTCGTAGTAATAATAT

CGCGAGGTTTACAGCGATAACTTGGGATATCTACTATACGCCCATTAACTAAAACGTGTC

TGTGGTTAACTAATTGTCTGGCTCCAGGAATGGTCGAGGCCATGCCCAATCGAAAAAGGA

TGTTATCCAAGCGCATCTCAAGTAGTTGTAGTAAAACCTGACCCGTTGATCCTTTGGCTT

TTCCAGCGATACGAACATATCTAAGTAATTGTCGCTCTGTTAGCCCATAATGAAAGCGCA

ATTTTTGTTTTTCTTCTAAACGAATACGATATTGAGATCTTTTCCCGGAACGCGGTTGAC

TTCTAAGACTACTTTCAGATCTAGGTCTTTTACTAGTGAGTCCCGGTAAAGCCCCCAAAC

GGCGTATTTTTTTGAAACGAGGCCCTCGGTAACGAGACATAAAAACTCCTTATTTTAAAA

AAATCGAAAATTAAAAAAAATGAAAAATGGACAGAATAAACTTAAATTAAGACTGAACTA

AACGATAAACAAAGGCAAATCCGCTGAAGTACTACAAAGAAGAATGAAATGAATTGTATT

CTATATATGGAAAATATTGTATATGTATACATAGGAAGTTAAGTGCCCCCTCCTTATTTT

TTCTGTATGGATCTAAATCCCCCGTTTTTCTCCATAGTTGGAAGTTCCTACAACATAAAA

AATCCATTATCCGACGTTTGGAGAAAAGGGGAGGAGCCTTTTCAATATTCCTTGATCTCA

AGAAGACGTTGTTATTTTCAATCATGAAAAAAATCGAACAAATAGAACAAGCCGGCTATC

GGAATCGAACCGATGACCATCGCATTACAAATGCGATGCTCTAACCTCTGAGCTAAGCGG

GCTCGCATAAAAAGAAAAAGTGCATAGAAATTCGGAAAACGCGGGGATCTTGGCTATATT

CTATGAATTTTATTCTATTCATTAATGAATAGAATAAAATACATTTTTTCATTAAATGAG

TTATTTAACTATTAAAATTATAATACTATTAAAATTATATTATAATAGTTATAATAATTA

ACTATTAAATTAACAAGAGTATTCCAAATTTGAAGAGTTTTGTTTATGAACAATATTATC

CCTAACTATTATTAGTTCTAATAGTGATTAACTATAGATATATTATATTAGCTATAACAA

GATATATTATATTAGCTATAACAGATTATAGAAATTCTATAAGATTCGAGTGTTAATCTA

ATAGATTAGACTATTAGGTAAATTAAATAGAGGATAAGATAAGGATAAAAATAAATAGAA

AGGTAGAGATAGGGTTGCAATTCATATAATAATGAGATATTCCTACGGTTTCGTTCGGAA

AGGTAGGAGATAGGACGACAAAAAAGAAGAATCAATATCGACCGTTCCAGTATTATAAAC

AAAAAAGTGCGAGAAGAATTAGAGCGGAAAAGGCATATATATATATACGGGATAGGTCCA

CCCATATTGAATTGCAGATCTATCATTGATAGACTCTTTTTGGATTGGGCCGAACAAATA

CAAATATGCGCATCGAGTCTTCCTAACGAGACGAAAGAAGATAGACAAGAAATAGAATAT

GAAGTAGACGCTTTTTCGATATAGGGGTAAGTATATTATCTAGTGAATTACAAGGTTCCA

GCCAAACTAAATGAAAGAGGAGGGGATGTTCACAATAGGACCCCTGTCTTATAAGGATAA

GTAGGTGTAAAGGGGGATATGGCGAAATTGGTAGACGCTACGGACTTGATTAGATTGAGC

CTTGGTATGGAAACCTACTAAGTGGTAACTTCCAAATTCAGAGAAACCCTGGAATCAAAA

GTGGGCAATCCTGAGCCAAATCCTATTTTTCGGAAAACACTAGTTCAGAAAGCGAAAAAG

GGATAGGTGCAGAGACTCAATGGAAGTTGTTCTAACGAATCAAATGGGGTTGCCTTTACC

TTTATTAATTGCATTGGTATAGGAATCCTTCTACTCCATAAAATAAGAGAAAGAATTACC

CTGGATGCGTACTGAAATAGCAAAGATTCATCACGACCTGAATTCTTTTTTTTTTTCAGA

AAAATTCTGAAATTGTTCTGAATTGATTCCAGAGGGAAGAATCAAATATTCAGTGATCAA

ATCGTTTACTCCGGAGTATGATGGATCTTTTGAAGAACTGATTAATCGGACGAGAATAAA

GATAGAGTCCCATTCTACATGTCTATACTGACAACAATGAAATTTATGGTAAGAGGAAAA

TCCGTCGACTTGAAAAATCGTGAGGGTTCAAGTCCCTCTATCCCCAATAATAATAATCCC

TATTTGACTCCCTAACCATTTATCCTCTTTCTTTTCTTAGCCAGCGATTCCAAGTTTTCT

ATGTTTATTACTTATTCTACTCTTTCACAAATGGATCGGTCAGAAATCTTTATCTATTCT

CAAAAATCTTGTAATAGATATTATCTACGTTAAAATATACGGTAAAACCCATACTATGCT

ATGGTAAAAATTTCCATCCCATTTATTATGGACTCACTTACAGTCCATATCATTACTCCT

ACATTTACAAGGTCTTCTTTTTTCTGAAAATCCAAGCAAGAAATTCCAGTGCTTTTTGAA

TTTCTTTAATTGACATAGACCAAGTCCTTTAGTAGGATAGTAATAATAATGTGTCGGAAA

TGGTCGGGATAGCTCAGCTGGTAGAGCAGAGGACTGAAAATCCTCGTGTCACCAGTTCAA

ATCTGGTTCCTGGCACGTAGTTAATGTATCCAATGGATACTCATCTAAATGAATCGATAT

TGATCTGGATTGCTGTTCGTTAATGGTCTATGATACATACTTATCCATCCATATGAATAG

ATATACAGCCGCGTTTTGTAGATGGGTAAAGAAAATGTATATGAATAAAGAAAAATATAT

AAAACATATATGAGTGAAAAAGAAAAGAATTAGATGCTGCCCCGTCTTCTTTTTTGTTTG

TTCATATTGTATCTAGTTCCGATCAAAAAGAACGCTGATACTTCATACATATCACATATC

CAATCGAAGTTAGTTAGCTGAGAATCCCCAAAACCTAGTCTAGAGGGATTAAAGAAAGGA

TAATAAATAATACACAGAATTGATTTCAGATACAGTACAAAAAAATCAGACTCCTTTTCA

TTTCTGAATTTGGATTTCAGAATTTCCTATTTTCTTTCCGATTCGATTTCTCTATTCGAT

TTCTTCACTCCTTATTATGTAAGTCTCCGTGGCCCACTTAAGTAATGAGTAATGTGCGCG

GTACAAAGTTCATGGTACAGAACTCTTTGGATTCATCCTGAGGCTCCGTTTGCCCCCCAA

AAAGAAAATAGATCTATTTCAAATTGGAGAATCTGAATGAAGCTTGATCCCACCTAGAAT

ACGAATAAGAGTTCAGTTACTCTGTTTCATCTGGAGCAGAACGTAAAAGGAGTCCTTGAA

TATCTGATCTAATCTAAAATCGTAGAAGTTAATAGTAATTTATAATTATTCAATGGGCAT

CTTGTATTTCATAGAAGTTAGGGGCAATATAATCTTTACGTAAGGGCCAGCCTATCCAAC

TTTCGGGCATCAAGATACGTTTCAGACGTGGGTGATTCTCATACGAGATTCCCAGCATAT

CATAAGATTCTCGCTCTTGAAAGTCTGCGCTTTTCCAAATCCAGAAAACAGACGGGATTC

TCGTATTTGTCCTTGGGACAAATACTTTGATGCATACCTCTTCTGGTTGATCTATACCAT

ACTGTATTCTCGTAAGATAATATACGCTAGCTAACAACCCGCCAGGCGCTGCATCATAAG

CACATTGGGAACGTAGATAATTGTAACCATATGCATATAAAATGACAGCAATGGAGTACC

AATCCTCCGGCTTTATTTGTAAAGTCTCTATTCCTTGGTAATCGAAGCCCAAAGATCTAT

GAACTAGCTCGTGTTTGACTAGCCAAGCAGATAAACGACCCTGCATCTTCTTGATCTCTC

CAGCATTTGTACGAATATTTTAAATTTACGATGAAATTTATGAAGATTGATCCGCCACTT

GTTATTCTGCACAAACAAATTCCAACTTACCTAATTTACTAATTCATAATTAGGTACTAA

ACTTTTGTATTTGAAAAAGTTTTCAGAGGCTGTTTCTGGCGTAGATGAAGATTGAGAAAG

TAATCCTTGATCGTAATTTCCAATATGAATACTGCGCCCAACATGAGACTTGTGAACATG

AAACTTGTGATTGGTAGTAAAATATCGATTTTCCTGTTGAGACCCAATTCTATCTTCATA

AATTTCTCGAGAGACCTTCTTACGAAGTTTCGTTATAGCATCCATAATTGCTTCTGGTTT

AGGCGGACAGCCCGGCAAATAGACATCTACAGGAATTAGCTTATCGACTCCCCGAACAGT

ACTATAAGAATCGGTACTAAACATCCCCCCCGTAATAGTACAAGCTCCCATAGCAATGAC

ATATTTTGGTTCAGGCATTTGCTCATATAATCTTACTAAAGAAGGAGCCATTTTCATTGT

TACTGTGCCAGCTGTTAAAATGAGGTCTGCTTGCCTAGGACTTGATCTTGGTACCAGTCC

ATAACGATCAAAGTCGAATCTCGAGCCTATTAATGAAGCAAATTCAATGAAGCAACAACT

GGTACCATATAGAAGTGGCCATAAACTGGAGAGTCTTGACCAATTCGAAAGGTCATTCAA

TGTAGTTGAAATAACTGAACTTTGGGTTGTTCGATCAAGTAACGGAAACTTCATAGAGTT

CATAACTGTCTCGATGTAAGCTTTTTCTTCTTTTTTGTTTTTATTATTTGAATATTCAGG

GACTAAGACCATTCCAACGCGCCTTTTCGCCATGCATAAACTGAACCAACAATTGGGATA

AGCACGAAAATGAAAGCTTCTATAAATACAGATACACCCAATACATCGAAACTCATTGCC

CATGGGTAAAGAAAGACCGTTTCAACATCAAAAACAACAAAAACTAGAGCAAACATGTAA

TACCGAATTCGGAATTGAACCCAAGCATCCCCCATTGGTTCTATACCCGATTCATAACTA

GAGAGTTTCTCCGGTCCTTCATTAATCGGGGCTAAAACCCCGGAAATTAGAAATGCCAAA

ATAGGAATAACACTTGATATTATTAGAAATGTCCAGAAAATATCATATTCGTGAAGAAGA

AACATAGATATACTCCTATGAATGTGGAATATGTGAATTAGTATACCCAATTATTCGAAC

TCGAATTGTCAATTCATCTGTAACTGCCTAGTCAAAATAACAACTTTGATCAAACCCCAT

TTTTTTTTTTTCGTTTGTATACATTGAGCCATGTTTCGTTTCAAGACTCAGCCAACGTAA

TCCCGCTCACATTTACTTCGACCCTTTTTAAGGTATATACGTATTTTGTTCTTATACAAA

AAACCCTCCTACTTTGTTTCGAGCTCTCCCTATTTTCTATAAGAATATAAGAATTGAAAT

AAAAGAATTCATTTTTCTTTCATTTTTTATTTCAATTTATTATTAAAATAGTATGAGTTT

CAATCATATGAATATTCATAATAAAAAAAGCTTAAACAAAGAAAATGGAAATCTCATGTA

AAATAATCACAAGAAAACAGAAGGGGGGGGGTCTGGCTCCATTTTCTCTTTTTTTTTATT

TATTTAGATATTTTGATTTAGTGTATAAATTTAATGTATAAATTGTATAATATAATCAGT

AGTCGGGTAGAGCGGAATGTGTAGAAATTTCAATCTCGGGATTTATGGTACATTCAATTT

GCTTTGATGTATTCTATTAATTAATAATTATTTTAATAAATCAATTATGTTGTTAAAAAA

AATCATAATAGAGGGTCATTCTTTCTGCTGATTCAATACGTACAGGAAATGAAATGCATT

AATCAATTAGATTCTATCTCTTTGATTTGCCCTAGATTTCAAATTTCTTTTAGATTGATT

AAATCCGTTGAATTGTGAAGAATTCTTTTCTATATAACAAACAGCTCATGAATTCTTATA

CCCAAATTAGAGACTTTTGTCCTCTACTTATCCTGCACTGCAGCGGCTGCCCCAGAAGAA

ATCGAGTTAGAGTTATTGAATTTCCAATTTTGGATTTGAGTTAAGTTACAGGTAGTTTTG

AATAAACTAAGTATTGTAGAGAAGGAGAAAAATGACTACTTCGCTTACCTTTCTAGTCCG

GTTTTACAAAAAAAATTCTATTTTAGAATATTTCTAATGGCACTTACCAAAGAGCTTCGC

TTTTGAAACTCTAGCTTTTCCACAAATACAAGAAAAAGAATAGGCCCCCGTGTGGCTTAC

TATTCAATTCAATTCTATTTAGTTGGTTGGGTCAGGTTCGAGTTTTTCGTTTTTAGCCAG

ACTTATGTTATGGTTTTGACTTCCTATCATAAATTGGATTGGAGTATACCAATTCAAGGG

CGAATTACCCAATCTTTAGTCATGTCCCGTACATGAAAGAAACGGGAAAAGCAAAAAGGT

CATACATATTTCATTCAACCACAAAGATTAATGGAATGGAGAAGAATGGCTCTTCTCTTT

TTTTATTTATCTAATACTAATAATATAATAGGAATCTCCGTAAGCGCGCCGGCCGGCCAC

AAGTACCAATGAGTAAATGAAAACTATCCAAGAATACAAAAATAGAAAATTTATAGGGCT

ATACGGACTCGAACCGTAGACCTTCTCGGTAAAACAGATCAAACTTTTTATTATCGAAAT

GATTCGAACTGTTTCAAAGACCCAACATGCATTTTTTTTTTGCTTTGCATTGGGCTCTTT

CATTAACTGATATAATGATCAGTTAGTCTACCATATTTTTTCTTGACAGGAAGATAACGA

GATGGTTCCACGTGCTCTGATTTATTATTTGTATTCTGATCCAGGAGAGCAATACCAGAG

TGTTTCAAAGAAGGGTTACCTTGACGTAGGTCTGTTTCCGGCCTAGATCATCCTAAGTGA

AATGGAGTTTCTATCGCCCCGCTCCAAGAGCCAAATATGATACTTCATACACCTTAAAGT

TCATAGGACGAGAAGAGATTATTTTGAGGTCCTTATACCCATTATGCCTAACATTGAATA

GACTAAGTATTTACCTTATCAATTATCAAATCAATGATGGGTTCTATTTAGCACCTGAAT

TGGCGCCGAATTGGACCGAACAAATATTTGTCAGGCTATTTTCCTCTTTCTTGTTCTTTC

TAATCTATGGAGTAAGACATCGATTTTTAAATAAGATCAAGTTGGTTGATTGTATGATGA

ACTTCCCTGAAAAAGCATTGGCGCGCGTGTAAACGAGGTGCTCTACCTAACTGAGCTATA

GCCCTTGTCATAGACATCTTAACATCTAGATAATTTCTTGTCAAGGTGGGTATTCCATGG

TACCACACGATAGTTCTTTAATGCCAAGGAAAGCGTTGCTTGGAAGTAGTATTCCATCTA

TAATCCCCATATCTGGGGTCCTCTTTTTTGTTATAATAAATAACCTACTTAACTCAGCGG

TTAGAGTATTGCTTTCATACGGCAGAAGTCATTGGTTCAAATCCAATAGTAGGTACAACT

TATTAGATACCATTTACTCCGGTATCTAATAAGTTTTCCACCTTCTTTTCTTTTTTTTTC

TCCTTTCCCTATTGGATATTCGATTTTCGTACCCTTATTCGGATCTGGTTGTATTTTTTT

CGTTGTATCGGGTTAGATCACAATTGATTATGTCTAATTGGCGTAACCAAAGTGTATAAA

CGGAGCTTTCTTTGATTATGCTGACGCGTCAACTAGTACTACGAAATCGTATTGGCAGCT

TCCACTCGTGTCCTAGCTCGCCTAAAAGCAAGATTGGCCTCAATTACTTGTCTCTTGCCC

TCAGCTTTACTCAAGTTAGCTTCAGCTATTTCAAGAGTTTCCTGAGCTTCTTGCAGATCA

ATGTCACTACCCCTCTCCGCATCATTTACCAAAATGGTGATCTCATTATTGCCTATTCTA

GCGAAACCGCCCATCAAAGCCATCGTTACCCATTGGTCATCGGGGCGTATTCTCAAAATA

CCTATATCTACAGCTGTAGCAATGAGGGCGTGGTTTGGTAATACGCCAATTTGGCCACTA

TTAGTAGGTAAAATAATTTCTTTCACTTCTGAGTCCCAAATAATTCGATTAGGAGTCAGT

ACACAAAGATTTAAGGTCATTTCTTTAATTTGCTCTCCTCATCTAAGTTCATAGCCTTCG

CGGTAGCTTCATCGATGTTACCTACCAAATAAAAGGCTTGTTCTGGAAGACCATCTAATT

CTCCAGAAAGGATTAATTGAAACCCCCTAATTGTTTCTGGGAGACCGACGTATTTCCCCG

GGGAGCCGGTAAATACTTCTGCTACGAAGAAGGGTTGTGATAAGAAACGCTCAATTTTTC

GTGCTCTTGCTACGGTTAAACGATCCTCTTCAGATAATTCATCCAACCCAAGAATAGCTA

TAATGTCCTGAAGTTCTTTGTAACGTTGTAAAGTTTGCTTAACCCTTTGTGCAGTTTCAT

AATGTTCCTCTCCAACGATCCTGGGTTGGAGCATAGTTGACGTTGAATCTAAAGGATCTA

CTGCCGGGTAGATTCCTTTCGCGGCTAATCCCCTTGATAGTACGGTAGTAGCATCCAAAT

GCGCAAATGTCGTAGCAGGAGCAGGGTCGGTTAAATCATCTGCGGGTACATAAACTGCTT

GAATGGAAGTTATAGATCCTTCTTTGGTAGAAGTAATTCTTTCTTGCAAAGAACCCATTT

CCGTACTAAGAGTAGGTTGATAACCCACAGCAGAGGGCATTCTACCTAATAGGGCAGATA

CTTCTGATCCTGCTTGGACGAAGCGGAAGATATTGTCGATAAATAGAAGTACGTCTTGCT

CATTAACATCCCGGAAATATTCCGCCATGGTTAAGGCAGTCAAACCAACTCTCATACGAG

CTCCCGGAGGTTCATTCATCTGACCATAAACTAGAGCCACTTTTGATTCTGCAAGATTTT

GTTCGTTAATAACTCCGGATTCCTTCATTTCTATGTAAAGATCATTTCCTTCACGAGTGC

GCTCGCCTACTCCGCCAAATACGGATACACCCCCATGAGCTTTGGCAATGTTGTTGATCA

ATTCCATGATGAGTACCGTTTTACCCACTCCAGCCCCTCCGAATAGTCCGATTTTTCCTC

CACGGCGATAAGGAGCTAAAAGATCCACTACTTTAATCCCTGTTTCAAAAATAGATAATT

TGGTATCTAACTGGATAAAAGCAGGCGCAGATCTATGAATGGGAGATGTTGTACGAGTAT

CTACAGGACCTAAATTATCAACGGGCTCTCCAAGAACGTTGAAAATTCGTCCTAGAGTAG

CTCCACCGACTGGAACACTTAGAGGAGTTCCCGTGTCAATTACTTCCATTCCTCTCGTCA

GACCGTCTGTAGCACTCATAGCTACAGCTCTGACTCGATTATTTCCCAATAATTGCTGTA

CTTCACAGGTTACATTAATTTGCTTACCGGCGGTATCCTGACCCTTAACTATCAAAGCGT

TGTAAATATTAGGCATCTTGCCCGGGGGAAAGGCTACATCCAGTACCGGACCAATGATTT

GAACAATACGCCCCTGATTTTTTTCTTCGAGTGTAGAAACCCCAGGACCGGAAGTAGTAG

GATTGGTTCTCATAATAATAATCAAAAGTGAAATATGTCGAAATCGATTGCGAATAATTA

CCGAATTGAAAAGAAATGGAAATGTCCGATATCAAATGGGTCGGTTAATTGAATAAGAAT

GAATAAGAAATAGAAAGTGAGAGTTCGACCGATTTGATTTTGTTAGTACCTTACGACCAA

ATTCCATTTTTTACTCATTCAATGAATGAGTTCATTTTCAAGTTCAACCAACGCCTTTTT

TCAAACAAAAAATATCAAGTAGATAAATAAGAATCATGAGAAAGTCTTTTATTTCTCTAT

CATTAGATAGAATTCTATCCCTATTTTCTATGTAATTCGAACCGGATCTCTATTTAGAAT

ATGATTCATTATTCCTATCTTATTGACCGTTGCTCATTCCTTATTTCAGCATATTCATTT

CCGCCTACTCTTGTTTTATTTATCTTTTTCATGTTCATGTATGAATGGAATCCCGCCTAT

TTTCCCATCTAGGATTTACATATACAACATATACCGCTGTCAAGGGTGAATATTTTATTA

TTTCGGTATTTCGATGAAGAGACTTTTTGAAATTTTCAAAGATTGGGTTGCGCCATATAT

ATGAAAGAGTATACAATAATGATGTATTTGGTGAATCAAATACCATTGTCTAATAACGAA

CCGTTCAAATTAGTGGATAGTTGGTACTATTTAATTGAAAATTTTGTGAGAAATATTCTC

CTGTTTGTGAAAGGTTTCATTCACGCCTAGCTTAATCCATGTCGAGTAGACCTTGTTGTT

GTGAGAATTCTTAATTCATGAGTTGTAGGGAGGGACTTATGTCACCAAAAACAGAGACTA

AAGCTTACGTTGGATTCAAGGCTGGTGTTAAAGATTACAAATTAACTTATTATACTCCTG

AGTATGAAACCAAAGATACTGATATCTTGGCAGCATTCCGAGTAACTCCGCAACCCGGAG

TTCCGCCCGAAGAAGCAGGGGCTGCAGTAGCTGCCGAATCCTCTACTGGTACATGGACAA

CTGTATGGACCGACGGACTTACCAGCCTTGATCGTTACAAAGGACGATGCTACCACATCG

AGCCCGTTGCTGGGGAGGAAAATCAATATATTTGCTATGTAGCTTATCCTTTAGACCTTT

TTGAAGAAGGTTCTGTTACTAACATGTTTACTTCCATTGTGGGTAATGTATTTGGCTTCA

AAGCCCTACGAGCCCTACGTCTGGAAGATCTACGAATTCCTCCTGCTTATTCCAAAACTT

TCCAAGGCCCACCCCATGGAATCCAAGTTGAAAGAGATAAATTGAACAAGTATGGTCGTC

CTTTATTGGGATGTACTATTAAACCAAAGTTGGGGTTATCGGCTAAGAACTACGGTAGGG

CAGTTTATGAATGTCTCCGCGGTGGCCTTGATTTCACCAAGGATGATGAAAATGTGAACT

CCCAACCATTTATGCGCTGGAGAGACCGTTTCGTATTTTGTGCCGAAGCTCTTTATAAAG

CGCAGGCCGAAACGGGTGAAATTAAAGGACATTACTTAAATGCTACGGCAGGTACATGCG

AAGAAATGATAAAAAGGGCCGTATTTGCTAGAGAATTGGGAGTTCCTATCGTAATGCATG

ACTACTTAACGGGGGGGTTCACCGCAAATACTAGCTTGGCTCATTATTGCCGAGACAACG

GCCTACTACTTCATATCCACCGTGCAATGCACGCAGTTATTGATAGACAGAAGAATCATG

GTATGCACTTCCGTGTACTAGCAAAAGCATTACGTATGTCTGGTGGAGACCATGTTCACT

CAGGTACGGTAGTAGGTAAACTAGAAGGGGAGCGGGAAATTACTCTGGGTTTTGTTGATT

TGCTACGTGATGATTTTGTTGAAAAAGATCGAAGTCGCGGTATTTATTTCACTCAAGATT

GGGTCTCTATGCCAGGCGTTTTGCCAGTAGCTTCAGGGGGGATTCACGTTTGGCATATGC

CTGCCCTGACCGAGATCTTTGGGGATGATTCCGTACTACAGTTTGGTGGAGGAACTATAG

GACACCCTTGGGGAAATGCACCCGGCGCAGTAGCGAATCGTGTGTCTTTAGAAGCGTGTG

TACAAGCTCGTAATGAAGGACGTGATCTTGCTCGTGAGGGTAATGAAATTATTCGTGAAG

CTGCTAAATGGAGCCCCGAACTGGCGGCTGCTTGTGAAGTATGGAAAGAAATCAAATTCG

AATTCGAAGCAATGGATACCTTGTGATCCAGTAGTTCTAGTTTGTTCCTTTAGTTTCAAT

TAAACTCGGCCCAATCTTTTACTAAAAGGATTGAGCCGAATAAAATAGAATAAAATAAAG

AAAATAAAGAAAAAGCAACGAGGATCCCATGTATTTGCATCTATTTTGCATAATATTATC

TATATAGATAGATAATATATGTCCACCTTGCCTAAGATATAAATAAAAAATAAGATCTAA

GATTTAATAACTCAACGCTGCTATTGTTAGATCCATAATTAATCCTATGGATCCTTAGGA

TTGGTGGATCCTTTTATATCCCACAGTTTAGGATCATAAAAAAATCAAACAAACTAAGGG

TCACAATTTCTTCTACCCATCCTGTATATTGCCCTTTTCATTCTGTGTTGCAATAGAAAT

TTCTTATTCTCTTATATAATAATTTCTTATTCTCTTATATAATATTATAAGATATATATA

TATATATAATATTATAAGATATATAATAAGAGTGCGTATTCTATTATAATAGTATGAGAT

TTTACGAAAGAAAATGATTTCTTCATAGTATAGTGAAGAAGAATTTTGATCTTTTTCTTG

TCGATAATAATTTGTATACAACATGGGAGAAACCTCTCCCTCTTTCTATTTTATATAGAA

GAAAAGGTTCTATCATATCTATCATATATAGTAAATTACTAAACATTCCAGATTCCCATG

AGAAAATCATTTATTTATTGCAATACTTAACTCCCTAATTGCAATACTTAACTCCCTAAT

TGCAATACTTAACTCCCTATTATATTACTTAATTTCATAATCTTAGTGATTGAATTTATA

TGTTTATTACGATAGGAGATAAAATAGTGAACTGATTATTCATCGAATGACTATTCATCT

ATTGTATTTTCATTCAAATAAGGAAAGGTTCTATGGAAAGGCGGTGGTTCAATTCGGTGT

TGTCTTACGGGAAGTTAGAATACTGGCGCGGGCTAAGTAAATCAATGGGCAGTTTTAGTA

GTCCTATTGGAAATATCAGCGGAAGTGGGGGCTCCACTATAAATGATAGGGATAAAAATA

TTGAGAATTGGGGTGATAGGGGCAGTTATAGTTGCCCTAGTGTTGATTATTTATTCGGTG

TTATGGACATTTTTGGTTTGGTTTCTGACGAGACTTTTTTCGTCAGGGATGGTAGTGGTG

ACACCTATTCCGTATATTTTGATGTCGAGAGTCAGGTTTTTGAGATTGACAATGATAGTT

CTTTTCTAAGTGAACTAGAAAGCTCTTTTTCTAGTTATCTGAATAGTAGATTTGGGCCGA

AGGACGACAATCGCGATTATTATTGTTACATGTATGATACGAAATACAGTTGGAATACGC

ACATTAATAGTTGCATTGATAGCTACCTTCGCTCTGAAATCCATATTGATAGTTACATTT

CAAGTAGTAGAGACTATTACAACGATAGTTACATTTATACTTTCATTTTTAGTGAAAGTG

TAAATGATAGTGAGAGCGGGAGCTCGGGTATAAAAACTAGCACTAATGATAACGATTCCA

ATATAAGAGAAAAATCGAACGATAACGATTTCGATATAAATAAAAAATACAGACATTTAT

GGGTTCAATGCGAAAATTGTTATGGATTAAATTATAAGAAATTTTTTCAGTCAAAAATGA

ATATTTGTGAACAATGTGGATATCATTTGAAAATGAGTAGTTCAGATAGAATCGAACTTT

CGATTGATCCGGGCACTTGGGATCCTATGGATGAAGACATGGTTTCTATCGACCCTATTG

AATTTCACTCGGAGGAAGAACCCTATAAAGATCGCCTCGATTCTTATCAAAGAAAGACGG

GTTTAACTGAGGCCGTTCAAACAGGTGTGGGTCAACTAAACGGTATTCCTGTAGCAATTG

GGGTTATGGATTTTCAGTTCATGGGGGGTAGTATGGGATCTGTAGTAGGCGAGAAAATAA

CCCGTTTGATCGAGTATGCTACTAATAGATCTCTACCCGTCATTATGGTGTGTGCTTCTG

GGGGAGCGCGCATGCAAGAAGGAAGCTTGAGCTTGATGCAAATGGCGAAAATATCTTCTG

TTTCATACGATTATCAATCAAATAAAAAGTTATTCTACGTATCAATCCTTACATCTCCTA

CAACTGGTGGAGTAACCGCCAGTTTTGGTATGTTGGGGGATATCATTATTGCCGAACCCA

ACGCCTACATTGCTTTTGCGGGTAAAAGAGTAATTGAACAAACGTTGAATAAAACAGTTC

CCGAAGGTTCACAAGTGGCTGAGTATTTATTCCATAAGGGCTTATTCGATCTAATTGTAC

CACGTAATCCTTTAAAAAGTGTTCTGAGTGAATTTTTTCAGTTACATGGTTTCTTTCCTT

TGAATTCTAATTCAAAGCATTAGCCTCAATTATTTTCAACGAATTGGAGTTCATCGGAAT

AAAATCAAAATAACAATAAAAACAACGTAGTTTTTCTTTGGTTACATAAGTTCACAGTTC

GATAGTAAGAATATAAGTAAGAATCAAATCAAAAGTTTTGGATAATGACTTTTTTCGTTT

TTCTCCAGATTGAATCGATTTTTCTCCTATCCCTTATTTTAGTACAGTATTACTGATTAC

TAATCAGTAACCCCCTATATTAGGGGAAAGGGTGAATTCTTCTTTTCTAGGGATAGCATT

TTTTAGGAAAATAAAAGAAATTCTATGTTCCCTTATTACGTATTAAGATATAGAATACGA

AAAATGCAAATAATGAAAATTTCTAATCGAAGTCTTGCCTATTTTTATATCTCCTGAGAA

CCCACATTTTGGACTAGGAATTCCCGTTTTGTTAGATTGGATCCTAACGAATGAATCCTT

ATAAGAAAAAGGCCTTATTATTATTAATCAGAAGATTAAGGGGGAAAACTAATAAAAAAG

CGGATTATCTATCATAGACCCATTTCATGTAGAAAGCTAAATAGGCACACCCCCTTTTGT

TCTACATTCTTTTCACTTATGATATACTTATCATACTTATAATATACTTATTATAAAATA

TCTTTATAATATAACATAACAGGTACAAATATTTAATCGAGGCACCCGTTCTATGACAGA

TTTCAGTTTACCCTCTATTTTGGTGCCTTTAGTAGGCCTAGTCTTGCCGGCAATTGCAAT

GGCGTCTTTATCTCTTCATGTTCAAAAAAACAAAATTGTTTAGCTTTGATGTAACCAAAC

CCTATCAATTTATTTTATTATTTGGCTTTGGATGATAATGATAATATAGATATCGATTTA

GTAGAATATGGTACGACGTGTAGATCTTTACGAACACAAACAAAAAGCAGTTATGCACAT

TTTGTCTAGATACATGATAGATGAATCGAGTATATACATATAGGGATAACTGTTTTCAAA

AAAGAAATTATCGGATCGGAAATAGAAAGTCAGTTTATCTATATGTTATGTAGATATACA

TAGTAAGATCATACTATAGAGGAATATTTTCTTTCTTATTTTACTTGCTAACCTGTTTGG

TGAATTATTCCTAATGGATTGGATGGTATGATCATAGTGCTAGTTGATGAGAGTTACTTC

GAGAGCAAAAAAATAATATAAGAAAGTCAAATCTATTTCATTTGGCTTAGCTTATTCTAT

CAATTCCAATAGAATACAACTGGATCTAGTATGAACCGGCGATCAGAACGTATATGGATA

GAATTTATAACGGGGTCTCGAAAAACAAGTAATTTCTGCTGGGCTTGTATCCTTTTGTTA

GGCTCGCTAGGATTCTTATTGGTTGGAATTTCCAGCTATCTTGGTTGGAATCTCATACCC

CTATTCCCCTATCAGCAAATCGATTTTTTCCCCCAAGGGATTGTCATGTCTTTCTATGGA

ATTGCTGGTTTGTTCATTAGTTCCTATTTGTGGTCCACGATTTTGTGGAATATAGGTAGT

GGTTATGATCGATTTGATAGAAAAGAAGGAATAGTGTGTATTTTTCGTTGGGGATTCCCT

GGAATAAATCGCCGCATCTTCCTACGAGTATTTATGAGAGATATCCAGTCCATCCGAATC

GAGGTCAAGGGGGGTGTTTATCCTCGTCGTGTCCTTTATATGGACATCAGAGGCCAGGGG

TCCGTCCCCTTGACTCGTACTGATGAGAATTTCACTCCACGAGAAATTGAACAAAAAGCT

GCGGAATCGGCCTATTTTTTGCGCGTACCAATTGAAGTATTTTGAAATGAACTGCATAAT

GAAAATTTTTTTTTTCAGTATGGGCGAAGGAACTCGGGAATACCCTTTTTGAATAGGGCC

GGGGTCCCTTTGTTTATTAGAGCAAACCGCGTTCGATTCTATTTTCCCTGTTCCATTAGT

AATACCGCCGTGACCTATAGAATAAAACAAACAGACGTATATAAAAGAAAAGAATCATAA

GAAGACGCCTTTTTCAACAAGGGATTTTTTATGCATAATGGAAAACTCCATTTTTTAGAC

TAGTATCTAGTATAAAGTAAAATAAGCATGAATTGTATTCATCATACATATCAGAGCATA

ATCCTTCAGAATACTGTACAGAATTCATCTTTTTATTTTTAGGGAAATACTTTGTTTTGT

TCAATCAATATGCCAATATACCATATTACTATATACATACAGATGGGTTATCTCTCATAA

ATTATCTCTCCTTTTTCAAGAGATCTTTATTTTTATCCCCTCTTTTGTTCCTTGATAACT

AAAGTATTTGATCTCTCATAAACATCCAACTTTTCTCCCGCTTTCCCCTCGTCCATTTCG

GATTTTCTCATCAATATTCGTCGTCAGAAATATCCCTAAACTACCCCTGTGGTGTGGGCG

GCTAGTGAAACATTTTTTCAATTATTGATTACCGAGGGAATGGAATTTCTTTCGTCTAGA

AATGCGAAGAAGATTCATTACTTATTTAATGTTTAAGTGATTTAATGTTTAAGTGAAGGC

GACTTTCAAGCATTCATCGAAAGAAACAAATGAAGAGAGGGTTGATTTGATCCATTGAGA

TATCTATCTGGAACGAACAATATTGGATCATTTCTTCACTCGAAAGAAAGAGGGGCCTTA

TCTATATCTTCTACTTAATATATATATCTTTATATATATCTTCTAGATATAGATATGAGC

ACTAAACGATCCAAGGTAATAGATCCACAGGTTCCATACCTTTTTATCGAACTCATGCTT

CATAGAAATATCAGATTAGACGGAGTTTACGAATGAAGTAGGTTCATTAACAATTCAAAG

AATAGATTCAAAGTGCCGAAAAAGAAAGCATTGGCCCCGCTCCTATATCTTGTATCTATA

GTTTTTTTACCGTGGTGGATCTCTCTTTCATTTCAAAAAAGTCTGGAACCTTGGATTACT

AATTGGTGGAATAGCAGGAAATCCGAAACTTTTTTGAATGATATTCAAGAAAAGGGCGTT

CTAGAAAAATTCATAGAATTAGAGGAACTACTTCTGCTAGACCAAATAACAAAAGAGTGC

CCCGAAACACAGATACAAAAGCTTTATATAGGAATATACAAAGAAACGATTCAATTGGTG

AAAATTAAAAATGAAGAGCATATCCATATTATTTTACAGATTTCGGCGAATATAATCTGT

TTTGCTACTCTAAGTGGCTATTCTATTCTATGTAATGAAGAACTTGTGATTGTGAATTCT

TGGATTCAGGAATTCCTATATAACTTAAGCGACACAATAAAGGCTTTTTCTATTCTTTTA

GTAACGGATTTATGTATTGGATTCCACTCACCCCATGGTTGGGAACTGATGATTGGTTTG

GTCTACAAAGATTTTGGATTTGCTCATAATGAGCAAATTATATCCGGTCTTGTTTCTACC

TTTCCAGTCATTCTAGATACAATTTTGAAATATTGGATCTTTCATTATTTAAATCGTGTA

TCTCCTTCACTTGTAGTGATTTATCATTCAATGAATGAATGAACAACTCGTTTGATCCGC

TGATATGAATCAAACAATAATGTTACTTTGTATATAAACAAGCAAGCCGTTTTGAATCTG

ATTCACTTTATACTTCTACCCGTGCAGGGTATTCAATTGCTCCTATATTCCAGTACAATT

ATTTCAGTCCAATGACAGAATTGTGGGTAGGGAACTAGGCTAGCTACCTACTTAATTTAT

TGTAGAAATTTCCGGGATCAATGATTGGACCATGCAAAATAGAAATACATTTTCTTGGGT

AAAGGAACCGATAAATCGATCTATTTCTGTATTGATCATTATATATGTAATAACTCGGAC

ATCTATTTCAAATGCATATCCTATTTTTGCGCAGCAGGGTTATGAAAATCCACGAGAAGC

AACTGGACGTATTGTATGTGCCAATTGCCATTTAGCTAATAAACCAGTAGATATTGAGGT

TCCACAAGCGGTACTCCCGGATACTGTATTTGAAGCAGTTGTTCGAATCCCTTATGATTC

ACAACTGAAACAAGTTCTTGCTAATGGTAAAAAGGGGGGTTTGAATGTAGGGGCTGTTCT

TATTTTACCCGAGGGATTTGAACTAGCCCCCCCGGATCGTATATCTCCCGAGATGAAAGA

AAAGATAGGCAATCTATCTTTTCAGAGCTATCGCCCCAATAAAAAAAATATTCTTGTAAT

AGGTCCCGTTCCTGGTCAAAAATATAGGGAAATTTTATTTCCCATTCTGTCTCCGGACCC

CGCCGCTAAGAAGGACGCTCACTTCCTAAAATATCCTATATATGTAGGGGGCAACAGGGG

TAGGGGTCAGATTTATCCAGATGGAAGCAAGAGTAATAATACAGTCTATAATGCTACAGC

TGCAGGTACAATAACGAAAATTTTACGTAAAGAAAAAGGAGGATATGAAATATCCATCGC

TGATGCATCGGACGGCCGCCAGGTGGTTGACAATATACCTCCAGGGCCAGAACTTCTTGT

TTCAGAGGGTGAATCCATCAAACTTGATCAACCATTAACAAGTAATCCTAATGTAGGTGG

ATTTGGTCAGGGAGATGCAGAAATAGTACTTCAAGATCCATTACGGGTCCAAGGTTTGTT

GTTCTTTTTGGCATCTGTTACTCTGGCACAAATCTTTTTGGTTCTAAAAAAGAAACAATT

TGAGAAGGTTCAATTGTCCGAAATGAATTTCTAGGCCCGCGGATTCATCAAGTTATCAAA

AAGAGATGCATTTTGGTTGATCGACATTGTATTATCCAAAAAAATCATGGAAAGCCCTTT

TCTTGTTTTATTTATACGGTTTTCCACGCGATGTCGGAAATTACTTGTATACTACTTACT

AGTAAAAGTATGTTGCAAAGAAGACTAATTGATCTGATCCTTTTTCGACCCAAATGGAAG

TGGTTTGATTATGCCAGTCCTACTATTTTCAGATTGAAAATAGCATGTAATGTATCAATT

CCTTAACAAAAGGAAAATCATAATCGACATACGCGCGTAGAAAAAAAATAGGTGGTAAAG

GGCAAGGGGAGGGGGTAAATGAAAAGAATAAATGATTTTAGGACGAGTAGCCCGTCTTAC

TAATCTTCCGCACAAGAAAAGGAAATTTCACCCTTACCCTTTTCTTGTGCTGTGCGGAAA

TAATAATGATTTTTGATACAGTTTGTTAAAGATTACTATTTCTTTATTCTTTATTTTCCA

GGCCCATCCGGTCTTCGCATAGAATAGTATGAATCAATCAATTGATTCAATTCCTCAACT

CAAAAACGGCGTCAAAAAGGAAAGGGTGCAGTGGTTTAAAACATCGGAGCAAGGGGTCTT

TTTTCTTTTCTACAAATATACAAATGAAAGCATACTCTATTTATTTGACTGGATGAATTT

CCAACCCTTTTATGTTATGAAGTGGAAAAAAGTATCCCTCGGGTAAGAACTTAACAGGGC

CTTAGTCTCCCATTCTTTGTTTTTATTTGATTTGGAGACTAAGGCCCTGTTAAGTTCTTA

CTTTTTCATGTTTACAATCCGGTTCACTCGATTACTATTACTATACCATCCCTATTATAG

GGATGAGCCCAATCCAGAATATGAGCCGTAAAAGAAAATACCAATTAAACCAATCACAAG

AATACCGGTTACAGTGCCTATCAGCCAAAGAGGAATCCTTCCAGTAGTATCAGCCATTTA

CCCAGCTTCCCTCCACATTTCATCAAGTGGTCATGCTAGAGACATAAACAGTCATGGATA

ATTATACGAATTATATCGTTCCGAATGGGATAAAAGAATTCCTACTTAATTTCTTTCTCT

CAATTGAAGAAATAATTGGAAAATAAAACAGCAAGTACAAAAATGAGTAATAACCCCCAG

TATAGACTGGTACGATTTAATTCAACATTTTGTTCGTTCGGGTTTGATTGTGTCGTAGCT

CTATAATTTGGATTAGATTTATCGTTGGATGAACTGCATTGCTGATATTGAGCCCAAAAA

AGAAACGGTAGGTACAGCTAGTCCGTGAACAGCCAACCATCGCACTGTAAAAATTGGATA

GGTTCGATCTATGGTCATTGAGGCCTCCTAAAAGGATCTACTAAATTCATCGAGTTGTTC

CAAAGGATCAAAACGGCCAGTTATTAATGGAATCCCTTGCCGGCTCTCTGTGAAATACTC

GTTTGGCCGAGGACTTCCAAACACATCGTAAGCTAAACCCGTGCTGACAAATAACCAACC

TGCAATGAATAGGGAAGGTATAGTAATACTATGAATGACCCAGTATCGAATACTGGTAAT

AATATCAGCAAAAGAACGTTCTCCCGTGCTTCCAGACATACCGAGCTCCACATATTTTTG

TACAGTCAAAGGGGATCGATTCTGTAAAAGATGGGATCAGTAAATGAAAAACCACTGACC

TTGCACTTTTGTGAGATCGTCAATATTGCACCAAAGGCTTATTTAGAGTATACCGAATCA

GTATAGCTATCCTCCCTCTGACGCAGCAACGAAGTTTATATCAATCAATATCAAAAAAAA

AATAGGCGAATTCCCTTCTTCCTTTGTTCTTACTTGTCTATGGAGACCCATATGTTATTC

AATGATTTCGAATCAAGTGACGGATTCGAATCAAGTAATTAGTGGATTGGTTATCCAATA

GTAGATAGGATTCGATGAAAGAATGAACAGTAGTTTGAACAACCCATATTCCTGATTCAC

CATTGCTGGATTAAATCAAACGCAAGGAGTTGTTCATTCGTTGATTCGAGATCCAGCTAC

AGGTACTCATGATGTGGAAAGACAGAATATGGAATCTAAAAAGGAGTTTACTAATCTTAT

AGCCAAGTGGGTCCCAAAATACAGATTTTTTTCTTCGAGAGATCGTTGTGTAATCCTTTT

TTGTTTTCATTCGAGATATTATATGCTATTTTGAAACCCACTACTGAATACACTAAGTTA

AAGCAAAGTTAAAGTAAGAGGTCTTATCTTACTGGGTCGTGTTTACAAACAAAATGGATT

CAGATTAAATCTTATCTACTATTAAATCTTATCTACTATTTAATCTTATCTACTAAAGAT

AAAGATATATATAAATAGATATAAATAGATATATAAAAATATATAAATAGATATAAATAG

ATAATATCTATCTACAGTTGAAAAAGAAATGATCAAAATACAAATTTTTTTTTTTCAAAT

CCTATTCTTTTGTTCAATAGTTATCCAAAGACAATTTCATTTTTTTTAATATACAGAGAC

CGTTCTAATTGTTATTTTTTGATACTTAACTCACGAGTGAGTTCCCGCGGAATCGAATCT

GTTGATTCGAAATCAAGGAATCGGTAGATGTCACAGATGATGAATCAATCTTCTTTTTTC

TATCCCTACCCTTGTCAACTATATCTTTATTAATCTTTATTAGTGTCGTCTATAATAATG

ACTGATGAATCAAGAACTTTCAATTGGAACGAACTGATTCTGTCAATTAGTGTTTTTTCT

TATCATCTTATCCTGTCTTCATAAATTTTCTTGCAAAAAGGAAAACTTAGGTAAGTGCTT

TATAAAACATATGTAAAAAAAAAAGAACATATCTCATTTAGCTCTTTCATGCCTACCATA

ACTAGTTATTTTGGTTTTCTACTGGCTGCTTTAACTATAACCTCAGCTCTATTGATTGGT

CTGAGCAAGATACGACTTATTTGAAATTCATTGAATGAATGAACAATTAATTCAAATAAA

CAAACTTTTTCTGTAGGGTTTTCAGGTATTCTCTAGTTTCATCCTTCATCCATTTTCAAC

TCTTGGCTTAGTATTGAGATTCATGGGCTGATTGGAATTACTATTTATTAATATTTAGGG

ATAGCTATTACTTTGATTTTCTCCCCCTTCAAACAAATTGAAATGATTGAAGTTTTTCTA

TTTGGAATTGTATTAGGTTTAATTCCTATTACTTTGGCCGGATTATTCGTAACTGCATAT

TTACAATACAGACGCGGCGATCAGTTAGACCTTTGATTGAGTAACATCTCTTTCTTTAAT

TGACCTCCTCCTTAATCTGCAGGAGGTCAATTTCAATTTGAGTTAGTAATGTTATTTTAC

TGTAATTCGACATTACAATGATAAGAACATGATATAATCACGCTCTGTAGGATTTGAACC

TACGGCATCGGGTTTTGGAGACCCGCGTTCTACCGAACTGAACTAAGAGCGCTTTCCTAT

GACATGGCAGGAGATAGAACTAAACTAGAAAGAAAAGGATTTTTTCCTACCCCACCCCCG

CCCCAACCCAATCTATTAGTTAGCATGCATCACAGTATTCTAAAATACTAGAATATGTCC

AACTTGAGTCGACCTCGATGGATTTCCCGTTACTACCTATAAGAGAAGTAATATGGTAGG

GATGACAGGATTTGAACCTGTGACATTTTGTACCCAAAACAAACGCGCTACCAAGCTGCG

CTACATCCCTTTCCATCAGTTGTACTATGTCATTGTACAGAATCCCTGTCTTGTTTTCCA

ATATGATGATTTCCCCTATCCTATAGAGGATACTATTTCTCTTGCCATTTCTTCTTTTTG

GTTTCTTATAGTTATAATAATAATAATAAACATATAAACATACGTATAAACATATATAAA

GAAAGAATCTTTTTGGGAATGCTAGAGAAGAAGGGATTCCCTTTTGTAATTCTGTTTTAG

GAACGGGTGGATCTCGTTTACCGGATTATTCTACCTAACTTTTTTAGTTAGGAGATTCTG

TGTACAAATACAAGTGACCTTTAACTATATCTAACTATAGAAATAGTTATTCTATATAGT

TAGATGCGTCCATCTGATAATATATCATATGCATTATAATAATAATAAAAAAAAAAAAAG

GAGGCTTTGCAATGCGAGATATAAAAACATATCTCTCCGTGGCACCCGTGTTAGCTACTT

TATGGTTCGGTTCTTTAGCGGGTCTATTGATAGAGATCAATCGTTTATTCCCGGATGCGT

TGGTATTCCCCTTTTTTTCATTCTAGTTTTTGGGCTAGAAGGGGGTGAAGAAGATTATAG

ATACTAAAAATTCTTTGTTACTAATTTCCTTCTTTTTTTTTTCATTTGGTTGAGATAAGA

AGGAAAGAAAAAAGTGGATTGAACCCCAGCGAAGCTCGAGCTCAGGATAGAATTAATCGG

GGGAGAGCAAAGAAAAAATGTGGTTTTAGGGCACAGGATGTTTGAGACCACACAAGATAG

TAAATGAAATACTGAGATTAAAAAATAGTTGATAGTTAGAAAAAATTGTATTACTTAGTA

TTACTCCATTGATGGAAATAAAATCTTTCCCAATTCGATTTTATGTTAGAAACTTCGATT

TCTTTTTTGCCCCTTTTTTTTGATTCAGGTAAAAAATAGAAGAGTTGAGTAAATCAAAAA

TGTAAAGGAGGTTCATGGCCAAGGGTAAAGATGCTAGAGTAATAGTTCTTTTGGAATGTA

CCAGCTGTACCCGAAATGGTTTCAATAAACAAAAAAAACCATCGGGCGTTTCAAGATATA

TTACTCAAAGGAATCGACACAATACACCCGGTCGATTAGAATTGAGAAAATTTTGCCCAT

ATTGTCACAAGCATACGATTCACGGAGAGATAAAGAAATAGAGTGAGTAGAGCATCTGTG

TTGTGTCCCCTTTTGAAGTTAAGCAAGGAAAAGGAAGAAATAACATATTATATATATATA

TATAAATAAATAAAATCCTATTTCCGTTGGATCCGAAAAAATAAATAAATAAATGAAGAA

ATCAAATAAAATAGGATTTTAGAGATAAGGAATGAACTTATGGATAAAACCAAGCGACCC

CTTCGTAAATCCAAACGATCTTTTCGTAGGCGTTTGCCCCCACCAATTGGATCGGGGGAT

CGAATTGATTATAGAAACATGAGTTTAATTAGTCGATTTATTAGTGAACAGGGAAAAATA

TTATCTAGAAGAGTGAACAGATTGACCTTGAAACAACAACGATTAGTTACTATTGCCATA

AAACAAGCTCGTATTTTATCTTCGTTACCTTTTCTTAATAATGAAAAACAGTTTGAGAGA

CCTGAGTCAATCCCTAAAACTGCCGGCCCTAGTATCAGAAATAAATAGGCCTACTCCTGA

AGAAAAAAACTTTAATTGGAACTCAAACTTCGATTGAAGTTCTTTTGGAAAAGCCGAGAA

ATTTTATTGTCACGTCGTAATAGAAAAAAAGAATCGGTTAAGAAGAAATCTTTTTTTTTA

ACGTGTTGGTTCATTCTTTCTCTTAACATATGAATTTATACTGTACCTCCCCGGAGTTCA

TTCTCCGGGGAATTCCGTTTAAATCATTTCGTTAAATTCTTCACATTCTCCCGATTTGAT

GATCTCATTAGAAATCATGTAAAAACAATTCCTATTTGATATAGCTATTTGCGCAAGTAT

TTTACGATTTAGAAGCAATTGTCTCTTGTACAAATCGTGTATTAATCGACTATAACTATA

GGATACCCCGCCCCCCCGGATTATAGCATTTATTCGAGTGATCCACAAACGACGAAAATC

TCTCTTTTTCCGGCCTCTATCCCGATGAGCAGAAACCAAAGCTCTCATTTTCTGTTGAGT

AATAGTTCGAGTAAGTCTTGAATGAGCCCCTCGAAAAGTTGATGCAAATAAACGAATTTT

TGTTCGGCGCCTGCGAGCTATATATCCTCGTCTAACTCTGGTCATTGAATCAAATAAAAC

TTTGATGAATAACTAATTGATTTTTTTCTTTCAGTCATTCTTACCCTTCCCTAGTTTAAT

AATTAATAACAAAACGGATTATTCCGATGTATAAAATATAAATTCCAACGGCTTTTGCTA

CTATGACCTTCCCAACCACTATTTTCTATTTCTTTCAGGCATTTCACCTCCAAACAAGAA

ATTGGACCAATATAAATAGAATATTGTATTGGACATAAAAAAAAATAGTAAAAAAATAGT

AAAGTAATAAGTAATGGTAGTAAATTACGAAAAAAAAATAGCGGTTTCCATCGTTTCTAT

GGTCACTTCTTAAACGGTGAGGCCCTCTCTATACACCGGAGCCCTTTCCTCATTTAATCA

AAGTTATTGGGAACTTGTACAGTTCACACAAACCTTTTGGCTCTACCCATGAATTATCGA

GTAATAGGTCTTTTCACAATGAAGATCTATCCATACAGTAACGGCATTTAATTAGGAAGG

TTGGCTAGGTAGCTGGCCCTCTTAGTCCGTTCTTGCAAGAGTTGGAACAAAATCTTCCCG

CTCTTAAATACTATTTTCCCGCTTAATGGATAACCATTGCTACCAGTGGGGAATTGCTTT

TCAACTCCAATTGAATTGATTGGATTTGCACCAATGAAAGCCATAAATTCCCTAAAATAT

AGGATAGGGTTCCGGGCTCATCGATTCTACCCTATTCATTGCTACCGATCATTGATACTG

GGAAAATCATATTGTTTTCTTCAGCTCATGATCTAAACGAGTCGCACATACACCCTAGTA

CATGTTCCTCGGCGCTGAGGACACCCCCGAAGAGCAGGGGATTTCGTGACATTTCGGATT

GGCTGTCTTGTGTTTCTAATAAGTTGTTTAATGGTTGGCATCTTGAATTCTGAATCGTAT

ACCGAATGGGCTGGTTTAGATCCATCCTAACCGGATGATTATGAATTATTTCATACTTCA

AGTAATTTGACTGGGGTAAGAAAAAGGAAAAAATACGAAATTACAGATTATTTGAATTAT

GGCTCGAAACAGGATTGCAGGTTTATGTGAAATTCCCGCTATTTTCGACGGCTACAAGAT

CAACAATGCCATGAGCTTGGGCTTCTGTTGCTGACATAAAGACATCCCTTTCCAGGTCTT

CAGATACAACCCATAAGGGGTTGCCCGTTCTTTGTACATAAACCCTTGTGAGGGTTTCGC

GGAGTTTCAGTAATTCTTCCGCTTCCAGGATAAATTCTCCTGTAGGTGCCTCATAAAAAG

AACTAGCAGGTTGGTGGATCATAACCCTGATGATATAAGATAATAAAAGGTTCTTCTGCT

TCGCACGGTCCTGCGAAGGGAAGGAAAAATAGAACAAGAAGAAAAAAGAAAGATAGAATT

GAACAACCGTACAGGCATGTTTTGCGCATTGCATACGGCTCTGTTATGGAATTTTGTTTT

CCCTCCCCTCTTTAAGAGAGAGAAAAATAGGATCTATCAGATCCAGTAAGCCATTTACCA

TCCTTTTTTAGGAGGAATCCAAAAATACTATGATGGTTCCGTTGCTTTAGATATTTTATC

TTATTATCTATCTCGTTTGCAATTCAGCAATCCCAAAGTGTTTTCTTGATCCGAAAAACG

AAGGAAAAATGAAAATGATCTTTTTTTGTATTCTTTCATAATATAAATATTGGAAAGAGA

CTTCGAGTGTAGAAGCAAAAGGGTTTGTGACGCTGAAACGGATCCCCCATCCATAAGATC

AAATCCGGAATAACCTTTGTTTCATACTACTACCTCGATACATAATCACATGTTGTGAAA

AACAAACAAAATTGTTTGCTCATATCAAATCCCAAATGCCATGCTATTATTACTTAATAT

TCATATTTTATATGGCGAAGGCATAGTCTTCTTCTTTTTTTTTCTTAAAAAAACTCATTG

GCGCCAAGCGTGAGGGAATGCTAGACGTTTGGTAATTTCCCCTCCGACCAGGATGAAAGA

TCCCATTGAAGCTGCTAATCCCATGCATATTGTATGTACATCTGGTGACACAAATTGCAT

AGTATCATAAATAGCTATTCCCGGGATTACCCATCCGCCGGGAGAGTTTATAAACAAATA

AAGATCCCTAGTACCATCCTCTATACTGAGATATACCATCAAACCAACAATTTGATTCGA

GATCTCGCTATCAACTTCTTGGCCTAAAAAAAGTAATCTTTCTCGATGAAGTCGGTTGAT

TAGGACAAAATTTGATCCCTTAGGAACCGTACGCGCATCTTTAGATGCATACGGTTCAAA

AAAAAAATAAAAAAATTGTGTGAAAGAAAGAATCAATGTGTCGACCCCAGCCCCCCTTTT

CGTAGCAAGCTTTTACTAAGGAAAGAAACCAAGCTAAAGAGGGCTTTTCCCCGATTTTTA

AAGAAAGATGAATAAGTTTCCAATTACTTCTTTTTAACCTATAAAAAAAAGAATTCATTG

AACTTATCGAATTAACCTCTCATTGATGTATTGTCACATTGAGATTCAAATCACAATGTT

ATTTTCTTGTTCCTGAATGGGTCCCCTCAATTCTTTTTTTAGGTTTATTTTCTACTCCGG

GTAAAGATCCGCCCGAATTGGATTCGCACATATAGGACAAATGCTGCCTATACCACTTCT

TTTTGGTACAATTTTTTTTTTTCAATTTCTTTTCATCATTTCTCATGTTTTCTTTTTCAA

AATAGGCAATGTATTTATCATATTACTTGATTGATTAGCAAGTTTGAGGTCGCTTCTATG

ATTAAGATTAAATAAGAATCTTTTATGATACTAGTAGTAAATGTGTAGGATTACTAATTT

GAAATTTTCTGAACGGAGCCTGTATACTTTATACTTTAATTTTTAAATTTAAAAATTTAT

ATTTATTTTTTTTCTTGTTAATCTAACCATAAATCTTATTAATTGATAATAATCCCCAAA

ATCAACAAACAAATAAATTGATCTAATTACACTTCACGCTCCGAATTCTTGATAGTTTAT

GATATAATCAATCTTTCTTGGGGAAGACGGGGGATATCTCAATCGGGAGAGAGAACGGGG

AAATACCATATGACCCAATATGTCTGACAAGTCGCACTATACGTCAACCCAAGCCGCGTC

TTCCTCTCCAGGACTCCGAAAAGGTACTTTTGGAACACCAATGGGCATAAAATGAAAGAA

AAGGGAGTTAAGTACTATACTTTACTTTTATGTGGAAACGTAACAATAGATTTTTTTGTC

TTCCTATTTTTCTTTTCTCGTTTTCCTAGTCGAATATTGTTGTTTATCATATTTTATCCA

TAGATTTAGGTTGAATAAAGTCAAAATCTTACGAATGGATCGCCGAATGATGAGCAAGCC

TTTTTGTATTGCATTTGCTCCAAAAGTGGGCCCAATCCCCATTGCGTATTGGTACTTATC

GGGTATAGAATAGATCTGTTTCTCTTTGCTCCTACGAACGGAATTGTTCAATTATTACTA

ACGGAACGGAATAAATTAAATAATAAATATGAACTGTTGATTCGAGATAATCCAATGGAA

GGGTAAGGTGCACAGCATAGTCTTTTCCAACTCAATGCGAGAAAGTCACATAGTGTCTAT

TTTTTTTTTTCATAAAGGGGTTTTTCTATGGGTTTGCCTTGGTATCGTGTTCATACCGTC

GTATTGAATGATCCCGGCCGATTACTTTCTGTCCATATAATGCATACAGCTCTGGTTTCT

GGTTGGGCCGGCTCGATGGTTTTATACGAATTAGCGGTTTTTGATCCCTCTGACCCTGTT

CTTGATCCAATGTGGAGACAGGGTATGTTCGTTATACCCTTCATGACTCGTTTAGGAATA

ACAAATTCATGGGGTGGTTGGAGTATTACCGGGGGGACTATAACGAATCCGGGTATTTGG

AGTTATGAAGGTGTAGCCGGGGCACATATTATGCTTTCTGGTTTGTGCTTCTTAGCAGCT

ATCTGGCATTGGGTGTATTGGGACCTAGAAATATTCCGCGACGAGCGTACGGGTAAACCC

TCCTTGGATTTACCCAAGATTTTTGGAATTCATTTATTTCTTGCCGGAGTGGCTTGCTTT

GGGTTTGGAGCATTTCATGTAACAGGCTTGTATGGTCCTGGAATATGGGTGTCCGACCCT

TATGGCTTAACCGGAAAAGTACAACCTGTAAATCCATCTTGGGGGGCAGAAGGTTTTGAT

CCTTTTGTTCCAGGAGGAATAGCCTCTCATCATATTGCAGCGGGGACATTGGGTATATTA

GCGGGCCTATTCCATCTTAGTGTCCGCCCGCCCCAACGTTTATACAAAGGATTACGTATG

GGCAATATTGAAACTGTCCTTTCTAGTAGTATCGCTGCTGTCTTTTTTGCAGCTTTCGTA

GTTGCTGGAACTATGTGGTATGGTTCAGCAACTACCCCAATTGAATTATTTGGGCCCACT

CGTTATCAGTGGGATCAAGGGTACTTTCAGCAAGAAATATATAGAAGAGTTAGCGCTGGG

CTAGCCGAAAATCTGAGTTTATCAGAAGCTTGGTCTAAAATTCCCGAAAAATTAGCTTTT

TATGATTACATTGGTAATAATCCGGCAAAGGGAGGATTATTCAGAGCAGGCTCAATGGAC

AACGGCGATGGAATAGCTGTTGGCTGGTTAGGGCACCCCGTCTTTAGAGATAAAGAAGGT

CGTGAACTTTTTGTCCGCCGTATGCCTACCTTTTTTGAAACATTTCCAGTAGTTTTGGTA

GATGGAGACGGAATTGTACGAGCAGATGTTCCTTTTAGAAGGGCGGAATCCAAGTATAGT

GTGGAACAGGTAGGTGTAACTGTTGAGTTCTATGGTGGTGAACTCAATGGAGTTAGTTAT

AGTGATCCTGCTACTGTGAAAAAATATGCTAGACGTGCTCAATTGGGGGAGATTTTTGAA

TTAGATCGTGCTACTTTGAAATCAGATGGTGTTTTTCGCAGCAGTCCAAGGGGTTGGTTT

ACTTTTGGACATGCTTCATTTGCTTTGCTCTTCTTTTTCGGCCACATTTGGCATGGTGCT

AGAACCTTGTTCAGAGATGTTTTTGCTGGTATTGATCCAGATTTGGATTCTCAAGTAGAG

TTTGGAACATTCCAAAAAATCGGAGACCCAACCACAAGGAGACAAACAATCTGATACAAC

ATTGCTCTGGTATATGTATATTTCTCTTCTATTTTTTTTGCTAATCTAATATAGAGTATC

GGAAAAGTCTTGATTTGGATCATTGCTTTCCTTTGACTCTTTCCCCTTCTTCCGGGAAAC

GATTCCAAATGCACAGGTACGTAAGCTATAATTGTAAACCACGATCGAATCTATGGAAGC

ATTGGTTTATACATTCCTGTTAGTATCGACTCTAGGGATAATTTTTTTCGCTATTTTTTT

CCGAGACCCTCCTAGGATTTCTACTAAGAAGATGAAATGATTTTGGATTATCTCAATTTC

AATTAAAATAACAAACCTCCCAATATAATAGGGAGGTTTGTTATTTCAACTAGTCCCCGT

GTTCTTCGAACGGATCTCTTAATTGTTGAGAGGGTTGCCCAAAAGCGGTATATAGGGCAT

ACCCGGTAAAGCTTACAAGTGAACCAGATATGAAGATGGTGACTAGGGTTGCTGTTTCCA

TTATTATAGAATTTTAAGACCATGAATGGTGGATCTACGTTACAATAAGATCCGTTTATT

TACAACGGAATAGTATACAAAGTCAACAGATCTCAATCAATGCAATAGGATTTATGGCTA

CACAAACCGTTGAGGATAGTTCCAAATCTGGGCCAAGACGAACTGTTATAGGGGATTTAT

TGAAACCTTTGAATTCGGAATATGGTAAAGTAGCCCCTGGGTGGGGAACGACCCCCTTGA

TGGGCGTCGCAATGGCCTTATTTGCGATATTCTTATCCATTATTTTGGAGATTTACAATT

CTTCCGTTTTACTGGATGGAATTTTAGTCAGTTAGTTCTATAATAACTACGAAGTCCTGA

TGTTTCAATCAAAATCAAGAAAACCCGGGCTTTTCCATTCAATCCTAAATTTTGGAAGAC

TCGGGTTACTGGGTTGGTAGTTCGATCGTGGAATTCCCTTGTTTCGGTATTTCCGGAATA

TGAGTGTGTGACTTGTTCTAATTGATCCTATTGATAGTACAGAAAACAGATCTGTCATCT

CGATAGAGATGGGCTTTGCCTCGTCGGATATTTATTCGAGTATCTGGAGCACAGAATATA

TGGAATAGATCAAGAAATATTTGAACTATGATTCATGCGTACTATTCATACCTCGCAGCC

GGACTCCCAAAAATGTGTAAAGGGGTCTCAAATAGTTTTTCTTCTTTCCGAAGCACTCTT

ATCCTAGACTGAAGGAGATATAGCTATATTTACTTGGATTTTTATTTTTATCCATAACAC

CCATTATCCATTCATTGATAAGTGATGATCAAAGGGTTCTTACTCAAAGAACTTTTTGGT

TTGGGGGCTTTTTGAATCATCGTGGTTCTAGTATGAATCTGAGGTTTTAATCAAATCATA

GGGTCTCAACAAGAGAATTCCTATTAATTCCTATTAATTTCTAGTAAATAGTGTAAAATT

TCTAGTAAATAGTATAGTTAATAGTTAAATAGTATAGTATAGTAAAGTAAATTCGCATTT

CAAAAAAAAAATGAATTCATAAAAAAATGAAATGAATAAAATAAATAGGAGAGGAGAATA

TTCAAGAGGCCTGTAACGATCAGCACAAAGACGGATGAGCCAACTTGATATTTTGGCATT

ATCATCAAAAACGGGATTTCGGGTTTTGGTTCTTTCGTATACTCAGAGATGATTGAATCA

AGTAGTTAAGAAGTTTCAAACTTTCTATTCTATTACATATCTGTTTTAACCAGTATTTGT

GTGTTTCTGCTTGAGCCGTACGAGATGAAATTCTCATATACGGTTCTCGGAGGGGGAGTC

CTTGGGTTTACCTATCTCAATAAAGTATATGATTGGTTCGAAGAGCGTCTCGAGATTCAG

GCGATCGCAGATGATATAACCAGTAAATATGTTCCCCCCCATGTCAACATATTTTATTGT

CTAGGAGGGATCACACTTACTTGTTTTTTAGTACAAGTTGCTACGGGTTTTGCTATGACT

TTTTACTACCGTCCGACCGTTACCGAGGCTTTTGCCTCGGTTCAATACATAATGACTGAA

GCTAACTTTGGTTGGTTAATCCGATCAGTTCATCGATGGTCGGCAAGTATGATGGTCCTA

ATGATGATTCTGCACGTATTTCGTGTTTATCTCACAGGTGGATTTAAAAAACCTCGCGAA

TTAACTTGGGTTACCGGTGTAATTCTGGCTGTATTGACTGCATCCTTTGGCGTAACCGGT

TATTCCTTACCTTGGGATCAGATTGGTTATTGGGCAGTCAAAATTGTGACAGGCGTACCA

GAAGCGATTCCTGTAATCGGATCCCCTTTAGTAGAATTACTGCGTGGAAGTGCTAGCGTG

GGTCAATCCACCCTGACCCGCTTTTATAGTTTACACACTTTTGTATTACCTCTTCTTACT

GCCGTATTTATGTTAATGCACTTTTCAATGATACGTAAGCAAGGCATTTCGGGTCCTTTA

TAGAAAAAAACGGATTATAGATATGTATAATCGATCATAATTATTTCGGGGAGGAACAAT

AGGATTTCATTGCTACAAACATGGATTATTGAAAAGAATAAGACATTCTAGTTGGAAATT

TTTTCCTCAACTAACTGCGAAATATTCTTTATTTTGTATGCGTAGTTGAAGCGGATTCTC

TTCAGAGAAGATAAATGGATTATGGGAGTGTGTGACTTGAACTATTGATTGAGCTGTGCA

GATATATGATCCGCCACATTGGATTGGAATTTACAACCAAATGTGTCTCTGTTCCAACCA

CCACATAATTCCCTTACAGACAGAGAAGAGGGTAGGCCGGTTCGCTTGAGGAGAATCTTT

TCTATGATCATACCCGAATCATGTTATGCATAAACAGGCTCCGTAAGATCCAGTAGAATA

AGTAATGTGATATGACCCACGCTCCATCTATTCTATTTACTTAATACTATATACTATGGA

ATATAGTATGGAAATGCATTTATTTCCTGTGCATCAATTCCGATCTATGATACTATCGGA

GTGAAATATGGGATCTAAGGAAGAGCAAGGGCTAGACTTATTAGTAACAAGTAAATCCTT

TGTATGTAAGAATACTCGAGATATTGTGGGGATAAACACAAATTGCAAGGAGTGAGACGA

TCCAAAAAGCACTTGATCATGATCAAATGTCTAAGCTTACTTGGGTATTGAGCATTTACT

TCCATTTACCTCTAAAAATGGAATTCTTTGTAATGGATGGTTGCAATTCCATTAAAATGA

AATTCGGTAAACCTTTTCTTAACACAGAGTTTCATGTATATGCGTGGATGACATATAGAT

TTAATCAAATTTATATGGATCCATTTTTTTTCTTTCATTATTGCTCGAGCCGGATGATGA

AAAATTATCATGTCCGGTTCCCTCGGGGGATGGATCTATAATAATTCACCTATCCCAATA

ACAAAGAAACCCGATTTGAACGATCCTGTATTAAGAGCGAAATTAGCTAAAGGGATGGGG

CATAATTATTATGGCGAACCCGCATGGCCAAACGACCTTTTGTATATTTTTCCAGTAGTA

ATTCTAGGTACTATTGCATGTAACGTGGGCTTAGCAGTTCTAGAACCGTCAATGATTGGT

GAACCAGCAGATCCATTTGCAACTCCTTTGGAAATATTACCGGAATGGTACTTCTTTCCC

GTATTTCAAATTCTCCGTACAGTACCCAATAAGTTATTGGGTGTTCTTTTAATGGTTTCA

GTACCTACAGGATTGTTGACAGTGCCCTTTTTGGAGAATGTTAATAAATTCCAAAATCCG

TTTCGGCGTCCAGTGGCTACAACAGTCTTTTTGGTCGGTACTGTAGTAGCTCTTTGGTTA

GGTATTGGAGCAACATTACCTATTGATAAATCTCTAACTTTAGGTCTTTTTCAAATTGAT

TTAACCTTGAAATAAAATGAAATAAAATACCACAACTATGTATCTAGGGAATGGAATAGT

CGCTTTCAAGCGACTATTTCATTCCCTAGATACATAGTTGAAATTCCATTATAGATTCAG

TACGAATATACAGACCAGGCCGTGCTAAAGATGTAAAACCCTTTTATTTATCTTTAGAAG

AGATGAAATCATTTCAATAGATTGAAACCTTATTTTTAGGTAAATCAAATCCGAAATGTT

TCTGTAGAATGTCCAATATCCGTTTCACATCTTCCATGCAAAAATGTTCAATTTTTAGAA

GATCTTCTTGACTGTTATTCAAAAGGTCCAATAATGTATGTATATTGGACTTTTTAAGAC

AGTTATAGGTCTTGGAAGGCAATTCTGATTGATCAATAAAAATACATTTCAACGCCATTT

TTTTTTTGTTTTTCCTTATATTAGCGATAGCGAATTCATCATGAAAGGTAAAAAGAGCTA

AAGGGACCCCGTTTGCACTGTCCTCTAAATGGATTTCCCGCTCTTCCGCATGTAGAAAGG

GAATAAATAAATCAATCAAATTGCGGGAAGCTTCATAAAGTGCTTCCTTAGGAGTTAAAC

TTCCATTTGTCCATATTTCTAGAAAGAGTATCTCTTGTTTTTCATTCCCATTTCCGTAAG

AATGAACACTATGATTTGCATTTCGAACAGGCATGAATACTGCATCTATCGAATAACTTC

CATCTTCGTCGTTATTGGGGGTTTTCATACTATATCCGCGATCTCTCTCGATTTGTAATT

TAATACACAAATCAACCCGTTCCGTCAGGTTAGCTATATGCTGCGTAGCATCAACTATTT

CGACAGAAGGTGGTAAAATGATATCTTGAGCAGTTACATATCTAGGACCCCTGACGCAAA

TAGATGCGTCGAGAGTTCCATACAGATTACTTCTCAATACAATTTCTTTCAAATTCATTA

CAATTTCATGTACTGATTCTTCAATACCGACTATCGTTGAAAATTCATGAGGCACCTTCT

CAGATTTTACACGTGTAATACATGTTCCTTCTATTTCTCCAAGTAAAGCTCTTCGCATCG

CGATACCTATCATATCTGCTTGACCTTTCATAAGCGGGGACAGAATGAAACGGCTATAAT

AAAGGCGCTTACTGTCTATTCTTGATTCAACGCACTTCCACCGCGGTGCGCCGGTGGCTA

CTGCTATTTCCTCTCGAACCATGCTAATATTATTGATCTGATTTTTGAATCATTATTTAT

TTCTCTTGAAATCTGTTTAATTCTGATTTCTACACACGCCTTTTTTTAGGGGGCCTACAT

CCATTATGTGGCATTGGGGTTACATCACGTACGAAACTTAAAAGTAGACCACTTCTGCGA

ATGGCCCGCAATGCTGCATCTCTTCCGAGACCAGGACCTTTTATCATGACTTCTGCTCGT

TGCATACCCTGATCTACTACTGTACGAATAGCATTTCCCGCAGCGGTTTGGGCTGCAAAA

GGTGTCCCTCTTCTGGTACCCTTGAATCCACAAGTACCGGCGGAGGACCAAGAAACGACT

CGACCTCGTACATCTGTAACAGTCACAATGGTATTGTTAAAACTCGCTTGAACATGAATA

ACCCCTTTTGGTATTCTACGTCCAGCCTTACGTGAACCAATACGCCCACTTCTACGTGAA

CCAATCCTTGGTATAGGTTTTGTCATATATATATTTTTTCATCTTATCTTATTAAGTATG

AGTCAGAAATATACGGATATATCCATTTCATATTAAAGCGAATACTTTATTTGTATTTGT

ACATCGGATTCCTTGAAGAGTCCCTTTTAGAAAGATTATCCTTGTCTCTGTTTATGTCTC

GGGTTGGAACAAATTACTAAAATTCGTCCACGTCTACGAATCAAACGACATTTTTCACAA

ATTTTACGAACGGAGGTTCTTATTTTCATATTTTTTTTCCTTACCTTAATTCCGAATCTA

TTCTTTGGAAGAAAATAAGTCTCTTGAAATTTGGAACTCGAATCGGATCCCCACGCCCAC

GAAAGAAATGTTTAAAAGGGTGCCTAATCGTTCGAATCTTTGTTGCGAAGTCTATAAATT

ATACGTCCTCTGGTTGAATCATAACGACTTACTTCGATTTTGACTCTATCCCCTGGCAGT

ATCCGTATAAAACTGCGCCGGATTCTCCCTGAAACATAACCTAGGATTAAATCCTCATTA

TCTAAACGAACCCGGAACATACCATTGGGAAGTGATTCAGTAATTAAACCTTCATGAATC

AATTTTTGTTCTTTCATTCCAGGCAACCTCCTTGAAGTATCAACTAATGGAGGAGGGGTA

ATATTAGACAACTAGCCCTTCTTCCCCTTTTCAGAAATCGGAAGTTGCGGATCCAATTCA

GACACCTGAGCAGAGGGATTACCATATATAACACAAAATTTCTCCTCCAATGCCTTCTAG

TCGAGCTTCACGATCTGTCATTACCCCTCGAGACGTAGAAAGAATGACAATTCCCATTCC

GCCTAAAATTCTAGGTATTTTTTGATAGTTGGAATAGATTCGTAAACCCGGCCGGCTGAT

ACGCTTTAAATTTAAAATAGTTCTAGATGTTCCTTTCCTATTCCTTCTATGTCGTAGGGT

TGAAACCAAGAAATTTTTCCGATTTTCTCGATGTTTCCTAACGTTTTCAATAAAGCCCTC

TCGTAAAAGTATTCTAACTATGTTTTCGGTCATATTTGTAGATGCTATTCGAACCGTTCC

TTTTTTATCCATATGAGCATTTCTTATCGAAGTTATTATATCAGCAATAGTGTCCCTACC

CATGACAAACTAGAATTCTTATTGCCTCTTATTTAAAATATAATCAACATGTTTTCTTTT

TTTTTTTATTCCCTTTTGATTATTTTTTAAAGAATTTTTAAAGAATGAATAAAGAAAGAA

TAAATAAGGGGAAAAGTGAAGGGGTACGCGTGAGAAGGGGATATGCATGAGACATCATTT

CCATAATTGATCCATTTTAGATATCCTACTTGATTATATATCATGATCTCATCGACTAGT

ATTTATAATACCTCAGGAGCTAATGAAACTATCTTAGTAAAATTCAATTGTCTCAATTCC

CGAGCGATCGCTCCAAAAACTCGGGTTCCTTTTGGATTTCCTTCTTGATCAATGACAACT

GCTGCGTTGTCATCATATCGTATTATCATACCGTTGTCACGTTTAAGTTCTTTACATGTA

CGTACAATCACAGCCCGAATTACTTCGGATCTTTCTAGAGGCATATTTGGCACCGCTTCT

TTGATTACAGCAACAATAATATCACCAATATTAGCATATCGCCGATTACTAGCTCCTAAG

ATTCGAATACACATCAATTCTCGAGCTCCGCTGTTGTCCGCTACATTCAAATAGGTTTGA

GTTTGAATCATATAATTTGTTTTATCTGTTATTTTGATGCAAAGGGCGCAGGAAAAAAGA

AAGAAATATTTTTTGTCCAGAAATAGAAATTTGAAACAAAAAAAAGAAAATAATTCTCGT

TTTTCTTCACTATTTTTTTGTTTCAACAGCCTTATCCTGCAATAACGAATTGAGTTTGTA

TAGGCATTTTTGACGCAGCTATTGAAATCGCGGCTCTAGCTACAGTTTCTGATATTCCCC

CGATCTCATAAAGTATTCGGCCCGGTTTAACGACGGATACCCAATATTCAGGGGATCCTT

TACCCGAACCCATACGGGTTTCTGCGGGTCTTACTGTAACGGGCTTATCGGGAAATATAC

GTACCCATATTTTTCCCCCGCGACGCGCATACCGTGCCATAGCCCGTCGGCCTGCTTCTA

TTTGTCTAGATGTAATCCAAGCGGATTCAAGTGCTTGAAGAGCGTATCTACCGAAACAAA

TACGATTGCCTCGATAAGATATTCCCTTCATCCTTCCTCTATGTTGTTTACGGAATCTGG

TTCTTTTTGGGTTATAGTTGACGGGTATTTTTCCAACCCCATCTCTACTGCAGAACTGGA

CATGAGAGTTTCTTCTCATCCAGCTCCTCGCGAGCAAAATGCTCGATTGTATTTCTTCTA

TTTAATCAATAAGACGCTAAATCCTGCTATTTATTGATTTACTAAAAATCTTGCATTTTT

TTATTTGTTATACATCTGAAAATAGAGAGTCTGTATCTACATACAGATAGACATAGACTT

TTATAAGACGTCTGGTTCGATTTATAGAATAATCTCTTTCATTTTTATACCGAATCCTTG

TTTTGTTTGTTTTAACCTTTTATCGAATTGGCTCGGCCGAACAAAAAATCGGGCAATCCA

ATAAGCTATTCTTCGCGGGCGAATATTAACTCTTTTTTACGCCTCATTCGTAAGGTAAAT

CCACGACCTCGCAGAAAAATGAATTGGCTCCCGGTTGGTTTCGCCATCCCACCCAATGAA

TCATTAGGATTCTATTTACAAATAAATCCTCTGCAGTCACAGGTTCCGTCGTTCCCACTG

CTTCTCTATTAATGGATAGGCCTGAATTATGCAGTGGAGCTTTCAATGTTAATGAAATTC

ATTTCCGAGTCAATCTTCCCAGTCTCTATTGACTTTAGGCTCTCTATTTATTTGAGTTCT

CCTATCCTATGAACTGGATGATTGATCGAATTTTTATCCATATAAATGGAATTTAATGCT

TTCTTACACTGCTTTTTCTTTTTTTATGAGATGATTCGTAGGCCATACATATTGGAATCC

TATATCATTGATATTTTACTGATATCTTTCTCTCACCTTTCCATTTATCGGCATTCTTCT

ATTCTATTGTGATTCACAATACATAATTAGATTGCGTTTTCTTTTTTTATTTGATAGAAA

TCCATTTTATTTGATAGAAATCAATAATGTAGTTGCTACAACTATATGAAGTGTCAATCA

TATAGTGACTGCATATAGTGACTGATTTCTCGGATCTCGATAATACGAAGCAATGAGCTG

GTTATTAGTTCTCTAGTTGTTATTAGTTAGGGACCCCGCCGGTATGTTTATTGTTTTTTT

AATCCCAACCCTAAAGAAAAACCAACGAGTCGCACACTAAGCATAGCAATTCTTCCAAAA

GATAAATGAAATTTTTATTCAACCCTATAGAATTAAGAATTAGAATGACTAATTTTTTTT

TGAAGATAAAAACAATGAAACGGTTTTCGTTTTTTCTTCTATCGGGGTTCTATTATTTTA

TTTCTCATCCAAAAATATCCAAATTTTAATGCCTAATATCCCATAAATAGTTCGAACTGT

ATAGGAACAATAATCAATTTTAGCTCGAATGGTTTGTAGGGGAACCCTTCCTTCTCTGAT

CCATTCGACACGTGCAATTTCTTTTCCATCGATACGCCCTGCTATTTGTATTTGAATTCC

TTTTGTATCTGCTTGTTCAGTTAATTCAATAGCACTTTTCATTGCCTTTCGAAATGAAAC

TCGATTCTTTAATTGCAGAGCTATATGTTCTGCAAGAATAGCGGGTTGTGCATAAGGTTT

TGCAACTCGTATAATAGCAATGTTAAGTCTCCTGTTCACGGAAAGAAATTCTTTTTGTAC

ATTGGTCTGTAATTCGTCGATTCCCCGTGTTCGACCCTCCATTAACGAATTTGGGGATCC

CATATAGATTATTACCTGGATAAGATCCATTTTTTTTTGAATCTCTATATGGGAGATTCC

TTTTCCTTCGAAACCCGAGGATATTTTCATATTTTTTTCTACATAATTATGGATACAATC

TCGTATTTTTTCATCTTCCTGAAGACCTTTAGAATAACTTTTGGATTGTGCAAACCAAAT

GGAACGATGACTTTGATTTGCACCAAGTCGGAAACCTAGTGGATTTATTTTTTTTCCCAT

TTCTATATTTCTCTCTCCTTCTCCTCTAGATATTTTTCTATTATATATACGACCCCTAAC

TCTAAATATTAGAGATTCGCTTTTTTTTTAAGGAAGTGAAGTAGCAGTACTCATTTCATT

TAAGGTTCTATCCTTCAATACAATAGTTATATGACAGGTGGGTTTTTTTATCAGATAACT

ACGCCCTCGAGCCCGAGGCTTTAACCTTTTCAAAATAGCACCCTCATTAACTTCCGCTTT

ACTAATGTATGAGTCAGCTTCGTTGAAACCCAGATTGTGACTAGCGCTTGCTGCTGCGGA

ATAAAGCAATTTAAAAATGGGATAAGATGCTCGGTAGGGCATGAGTTCGAGTAGTGTAAG

TGTTTCCACATAAGAACGTCCACGAATCTGATCAATGACTCTTCGTGCCTTGTGAGCTGA

AATGTGTACATCTCGCGCGAAAGCTTGTACTCGAGTTCTCGCGATTCTCTTTAATCTATG

CTTTTGCTCGCTCTTCTTTGCCTTTCTCCAACTTGACATACGTTTTTTACCTCCGGCCAA

TGAATGAAAAACTCCTATTTTACGTCATTAACGACGAGATCGATTATCGTTTCTCGCGTG

TCCCCGGAAAGTAAGAGTAGGTGCGAATTCTCCCAATTTGTGACCCACCATACGGTCTGT

TATATAAATGGGTAAATGTTCTTTCCCATTATGAATAGCGATTGTATGGCCGATCATTGT

GGGGATAATGGTAGATGCCCGGGACCACGTTACTATTATTTCCTTTTCCCCCCTCATATT

GAGTTTCTCCATTTTTTTCAATAAATGATTAGCTACAAAGGGGCTTTTTTTTAATGAACG

CGCCATAATATTAATGAAATTATTCCCTTTTCTTTTTGTTTTGGTTTTGTAATTCGGAAG

GAAGATTTGTAAATACGAAACGAAGAAACGAACGAAACATAATCGAATGGATTCTTATCG

ATTTTCTTTTCCATATCCAAATTCCTATTTACGGCGGCGAAGAATAAAACGATCACTATA

TTTATTCCTTTTCCTACTTCTTCTTCCAAGCGCAGGATAACCCCAGGGGGTTGCGGGTTT

TTTTCTACCAATTGGGGCCCTTCCTTCCCCACCCCCATGGGGATGGTCTACAGGGTTCAT

AACTACTCCTCTTACTACAGGACGCTTACCTAGCCAACACTTAGAACCGGCTCTACCCAA

ACTTTTCTGGTTCACCTCAACATTACCCACTTGTCCGACTGTTGCTGAGCAGTTTTTGGA

TATCAAACGGACCTCCCCAGATGGTAATCTTAATGTAGCTGATTTACCCTCTTTTGCAAT

CAGTTTCGATACAGCCCCCGCAGCTCTAGCTAATTGTCCACCCTTTCCAAGTGTGATTTC

TATGTTATGTATGGCCGTGCCTAAGGGCATATCGGTTGAAGTAGATTCTTCTTTTTGATC

AATAAAAACCCCTTCCCAAACCGTACAAGCTTCTTCCAAAGCATACGGCTTTCCGGATGT

ATATGATGATATCTAGACAGATGGATCTTATATGAATCGTATGATGAAGTACCACACGAG

TGGATATAGAGGAATTCCAATCTGCTGAATCGCTCATGTTAGGATCTTATACATCCTAGG

TCTCCCCGTTCCGTCATCTGGCTTATGTTCTTCATGTAGCATTCAGACCGAATGACTCTA

TGAAATTACGTCGATACTTCCACATATTACGGGTAACGTAGGAGACATCTCTATTTTTCC

CCCGGGGGATCTTTAGAATTACCACTGCTTAGCTTTCAATTCGCCTCTGACCATCAAATG

AAATGTGAATAACTCGTCCTCCTCTCTTTGAAACAAGGGGCGCTTCCGGCTCTGTCCGTG

CTTCAAACAATTTTGTCTTCTCCATATTACCATATCTCTAGAGTCAATAATTTGATATGA

GGAACTACTGAACTCAATCACTTGCTGCCGTTACTCTTCAGTTTTCTGTTGAGGTCTATC

CCGTAGAGGTACTCAAATTGGATGACTGATCGATTTCTAGGTTTCGTCGTAAACCTAATT

GGTTACTTCCAATTACGTAAATCAATAGTTCAAACCGCACTCAAAGGTAGGGCATTTCCC

ATTGATATAGGAACTTCTGTACCAGAAACAATGGTATCTCCAATTATAGCCCCTCTGGGA

TGTAAAATATATCTCTTCTCACCATCCCCATAGTGTATGAGACAAATGTATGCATTTCGA

TTAGGGTCGTATTCTATGGTTACGATTCTACCAGATATGTCTTTGTCATTCCGTCGAAAA

TCGATTTTACGATATAGACGCTTATGACCTCCCCCTCTATGCCCTGCGGTAATGATTCCT

CTGGCATTACGACCTTTACCACAACGATGCTGTCCATAGATCAAATTATTTCGTGGATTG

GATTTCACTTGACTGTCTACGGCTCCTTTGCGTGTGCTCGGGATAGAAGTTTTGTATAAA

TTGATCGCCGTGTTATTAAGTATTTTCATTTAAGTTCTTTTCTCTATAAGAGGTGGAATA

GAATAACCCGGTTGAAGCGTAATGATCATACGTCTGTAATGCATTGTATGTCCTATAATA

GGTCCCATTCTTCTACCCTTTCCCTGGAGTCGATGACTATTCATAGCTATTACCTTGACA

CCAAAGAAGAGTTCGACCCAATGCTTGATTTCTGTCCTAGTTGATCCTGATTCGACATTA

GAAGTATATTGATTGTTCCCCAATAACCGAATACTTTTTTCTGTAAAGACTATATATTTG

ATTCCATCCATAAATCCATTTTATTCCCTATAAGTTCCAGTATCGATAAGAATTCTAGTT

CTTACTCTTCATATGTTATGGTATGAATATACCATACCAATTCGTTATGTATGGATGATG

AGATTCCATTGATACAGAGCCAATTCCACTAGACTTGTTGAACGTTCCCATTGGCGTGCA

TCCAGCAGGAATTGAACCTACGAATTTGCCAATTATGAGTTGGGCGCTTTAACCATTCAG

CCATGGATGCTTAACAGGGATCATCGTAAATAACCAAATTCCAATTGAAATGAAATCTTT

AGGAGGAATCAATGAAAGGACATCAATTCCAATCCTGGATCTTCGAATTGAGAGAGATAT

TGAGAGAGGTCAAGAATTCTCACTATTTCTTAGATTCATGGACGCAATTCGATTCAGTGG

GATCTTTCACTCACATTTTTTTCCACCAAGAACGTTTTATGAAACTCTTTGACCCCCGAA

TTTGGAGTATCCTACTTTCACGCGATTCACAGGGTTCAACAAGCAATCGATATTTTACGG

TCAAAGGTGTAGTACTGCTTGTAGTAGCGGTCCTTATATATCGTATTAACAATCGAAATA

TAGTCGAAAGAAAAAATCTCTATTTGAGGGGGCTTCTTCCTATACCTATTCTTCCTATAC

CTATGAATTCGATTGGACCCAGAAATGATACATTGGAAGAATCTTTTTGGTCTTCCAATA

TCAATAGGTTGATTGTTTCGCTCCTGTATCTTCCAAAAGGGAAAAAGATCTCTGAGAGTT

GTTTCATGGATTCGAAAGAGAGTGCTTGGGTTCTCCCAATAACTAAAAAAAAGTGTATCA

TGCCTGAATCTAACTGGGGTTCTCGGTGGTGGAGGAACCGGATCGGAAAAAAGAGGGATT

CTAGTTGTAAGATATCTAATGAAACCATAGCTGGAATTGAGATCTCATTCAAAGAGAAAG

ATATCCAATATCTGAAGTTTCTTTTTGTATCCTATACGGATGATCCGATCCGCAAGGACC

ATGATTGGGAATTCTTTGATCGTCTTTCTCCGAGGAAGAAGCGAAACATAATCAACTTGA

ATTCGGGACAGCTATTCGAAATCTTAGTGAAACACTTGATTTGTTATCTCATGTCTGCTT

TTCGTGAAAAAAGACCAATTGAAATGGGGGGGTTCTTCAAACAACAAGGAGGAGCTGGGG

CAACTATTCAATCAAAGGATATTGAGCATGTTTCCCATCTCTTCTCGAGAAAGAAGTGGG

GTATTTTTTTGCAAAATTGTGCTCAATTTCATATGTGGCAATTCTACCAAGATCTCTTCG

TTAGTTGGGGAAAGAATCAGCACGAGTCGGATTTTTTGAGGAACGTATCAAGGGATAATT

GGATTTGGTTAGACAATGTGTGGTTGGTAAACAAGGATCGGTTTTTTAGCAAGGTACGCA

ATGTATCGTCAAATATTCAATATGATTCCACAAGATCTATTTTCGTTCAAGTAACGGATT

CTAGCCAATTGAAAGGATCTTCTGATCAATCCGGAGATCATTTCGATTCCATTAGGAATG

AGAATTCGGAATATCACACATTGATCAAAGAGATTCAGCAACTAAAAGAAAGATCGATTC

TTTGGGATCCTTCCTTTCTTCAAACGGAACGAACAGAGATAGAATCAGATCGATTCCCGA

AATGCCTTTCTGGATATTCCTCAATATCCCGGCTATTCACGGAACGTGAGAAGCAGATGA

ATAATCATCTGCTTCCGGAAGAAATCGAAGAATTTCTTGGGAATCCTACAAGATCAATTC

GTTCTTTTTTCTCTGACAGATGGTCAGAACTTCGTCTGGGCTCGAATCCTACTGAGAGGT

CCACTAGAGATCAGAAATTGTTGAAGAAACAACAAGATGTTTCTTTTGTCCCTTCCAGGC

GATCGGAAAATAAAGAAATGGTTGCTATATTCAAGATAATTACGTATTTACAAAATACCG

TCTCAATTCATCCTATTTCATCAGATCCGGGATGTGGTATGGTTCCGAAGGATGAACCAG

ATATAGACAGTTCCAATAAGATTTCATTCTTGAATAAAAATCCATTTTTGGATTTATTTC

ATCTATTCCATGACCGGAACAAAGGGGGATACACGTTACACCACGATTTTGAATCAGAAG

AGAGATTTCAAGAAATGGCAGATCTATTCACTCTATCAATAACCGAGGCGGATCTGGTAT

ATCATAAGGGATTTACCTTTTCTATTGATTCCTACGGATTGGATCAAAAAAAATTCTTGA

ATGAGGTATTCAACTCCAGGGATGAATCGAAAAAGAAATCTTTATGGTTTCTACCTCCTA

TTTTTGATGAAGAGAATGAATCTTTTTACGGAAGGATCAGAAAAAAATCGGCCCGGATCT

CCTGCGGCAATGATTTGGAAGATCCAAACCCAAAAATAGTGGTATTTGCTAGCAACAACA

TAATGGGGGCGGTCAATCAATATAGATTGATCCGAAATCTGATTCAAATCCAATATCGCA

CCTATGAGTACATAAGAAATGTATCGAATCGATTCTTTTTAATGAATCGACCCGATCGCA

ACTTCGAATATGGAATTCAAGGGGATCCAATAGGAAATGATACTCTGAATCATCTAACTA

TAATCAAATATACGATCAACCAACATTTATCGAATTTGAAAAAGAGTCAGAAGAAATGGT

TCGATCCTCTTATTTCTCGAACCGGGAGATGCATGAATCGGGATCCTGATGCATATAGAT

ACAAATGGTCCAATGGGAGCAAGAATTTCCAGGAACATTTGGAACATTTCGTTTCTGAAC

AGAAGCGCCGTTTTCAAGTAGTGTTCGGTCGATTACGTATTAATCAATATTCGATTGATT

GGTCCGAGGTTATCGACAAACAAGATTTGTCTAAGTCACTTCGTTTCTTTTTGTCCAAGT

CACTTCTCTTTTTGTCTAAGTCACTTCGTTTCTTTTTGTCTAAGTCACTCCCTTTTTTCT

TTGTGAGTATCGGGAATATCCCCATTCATAGGTTCGAGATCCACATCTATGAATTGAAAG

GTCCGAATGATCAACTCTGCAATCCGTTGTTAGAATCAATAGGTGTTCAAATCGTTCATT

TGAATAAATTGAAACCCTTTTTATTGGATGATCATGATACTTCCCAAAGATCGAAATTCT

TGATCAATGGAGGAACAATATCACCCTTTTTGTTCAATAAGATACCAAAGTGGATGATTG

ACTCATTCCATACTAGAAAGAATCGCAGGAAATCCTTTGATAACACGGATTCCTATTTCT

CAATGATATCCCACGATCGAGACAATTGGCTGAATCCCGTGAAACCATTTCATAGAAGTT

CATCGATATCTTCTTTTTATAAAGCAAATCGACTTCGATTCTTGAATAATCCGCATCACT

TATGGTTCTATTGTAACAAAAGATTCCCTTTTGATGTGGAAAAGGCCCGTATCAATAATT

ATGATCTTACATATGGACAATTCCTCAATGTCTTGTTCATTCGCAACAAAATATTTTCTT

TGTGCGTCAGTAAAAAAAAACATGTTTTTGGGGGGAGAGATGCTATTTCACCAATCGAGT

CACAGGTATCTGACATATTCATACCTAACGATTTTCCACAAAGTGGTGACGAAACGTATA

ACTTGTACAAATCTTTCCATTTTCCAATTCGATCCGATTCATTCGTTCGTAGAGCTATTT

ACTCGATTGCAGACATTTCTGGAACACCTCTAACAGAGGAACAAATAGTCAATTTTCAAA

GAACTTATTGTCAGCCTCTTTCAGATATGAATCTATCTGATTCAGAAGGGAAGAACTTGC

ATCAGTATCTCCGTTTCAATTCAAACATGGGTTTGATTCACACTCTATGTTCTGAGAAAG

ATTTCCCATCCGGAAAGAGGAAAAAACGGACTCTTTGTCTAAAGAAATGCGTTGAGAAAT

GGCAGATGTATAGAACCTTTCAACGAGATAATGATTTTTCAAATCTCTCAAAATGGAATC

TGTTCCAAACATATATGCCATGGTTCCTTACTTCGACAGGGTGCAAATATCTAAATTTCA

CCCTTTTAGATACTTTTTCAGACCCATTGCCGATACTAAGTAACAGTCCAAAATTTGTAT

CCATTTTTCATGATATTATGCATGGATCAGATATATCATGGCCAATTCCTCAGCAAAAAG

GGCGGGCGATTCTTCCACAAAGGAATTGGATAAGTGAGATGTCGAGTAAGTGTTTACAGA

ATCTTCTTCTGTACGAAGAAATCATTCATCGAAATAATGAGTCACCCGCTCTATTAATAT

GGACACATCTGAGATCACCAAATGTTCGGGAGTTACTCTATTCAATCCTTTTCCTTCTTC

TTGTTGCTGGATATCTCGTTCGTACACATCTTCTCTTTGTTTCCCGAGCCTCTAGTGAGT

TACAGACAGAGTTAGAAAAGATCAAATCTTTAATGATTCCATCATACATGATGGAGTTGC

GAAAACTTCTGGATAGGTATCCTACACCTGAACTGAATTCTTTCTGGTTAAAGAATCTCT

TTCTAGTTGCTCTGGAACAATTAGGAGATTCTCTGGAAGAAATACGCGGTTCTGCTTCTG

GCGGCAACATACTATTGGGTGGTGGTCCCGCTTATGGGGTCAAATCAATACGTTCTAAGA

AGAAATATTTTCATATCAATCTCATCGATATCATTGATTTCATAAGTATCATACCAAATC

CCATCAATCGAATCACTTTTTCGAGAAATACGAGACATCTAAGTCATACAAGTAAAGAGA

TCTATTCATTGATAAGAAAAAGAAAAAAAAATGTGAACGGTGGTTGGATTGATGAAAAAA

TCGAATCCTGGGTCGCGAACAGTGATTCGATTGATGATGAAGAAAGAGAATTCTTGGTTC

AGTTCTCCACCTTAACGACAGAAAAAAGGATTGATCAAATTCTATTGAGTCTGACTCATA

GTGATCATTTATCAAAGAATGACTCTGGTTATCAAATGATTGAACAACCGGGATCAATTT

ACTTACGATACTTAGTTGACATTCATAAAAAGTATCTAATGAATTATGAGTTCAATAGAT

CCTGTTTAGCAGAAAGACGGATATTCCTTGCTCATTATCAGACAATCACTTATTCACAAA

CCTCGTGTGGGACTAATAGTTCTCATTTCCCATCTCATGGAAAACCCTTTTCGCTCCGCT

TAGCCCTATCCCCTTCTAGGGGTATTTTAGTGATAGGTTCTATAGGAACTGGACGGTCAT

ATTTGGTCAAATACCTAGCGACAAACTCCTATGTTCCTTTCATTACGGTATTTCCGAACA

AGTTCCTGGATGACAAGCCTAAAGGTTATCTTTTTGATAATATCGATATTGATGATAGTG

ACGATATCGATATTGATGATAGTGACGATATCGATATTGATGATAGTGACAATATTGATG

ACCTTGATACGGAGCTGCTAACTATGACGAATGTGCTAACTATGTATATGACGCCGAAAA

TAGACCGATTTGATATCACCCTTCAATTCGAATTAGCAAAAGCAATGTCTCCTTGCATCA

TATGGATTCCAAACATTCATGATCTGTATGTGAATGAGTCGAATTACTTATCCCTCGGTC

TATTAGTGAACTATCTCTCCAGAGATTGTGAAAGATGTTCCACTAGAAATATTCTTCTTA

TTGCTTCGACTCATATTCCCCAAAAAGTGGATCCCGCTCTAATAGCTCCGAATAAATTAA

ATACATGCATTAAGATACGAAGGCTTCTTATTCCACAACAACGAAAGCACTTTTTCATTC

TTTCATATACTAGGGGATTTCACTTGGAAAAGAAAATGTTCCATACTAACGTATTCGGGT

CCATAACCATGGGTTCCAATGCACGAGATCTTGTAGCACTTACCAATGAGGCTCTATCAA

TTAGTATTACACAGAAGAAATCAATTATAGACACTAATACAATTCGATCAGCTATTCATA

GACAAACTTGGGATTTGCGATCCCAGGTGAGATGGGTTCAGGATCATGGGATCCTTTTCT

ATCAGATAGGAAGGGCTGTTGCACAAAATGTACTTCTAAGTGATTGCCCCATAGATCCTA

TATCTATCTATATGAAGAAGAAATCATGTAAGGAAGGGGATTCTTATTTGTACAAATGGT

ACTTCGAGCTTGGAACGAGCATGAAGAAATTAACGATACTTCTTTATCTTTTGAGTTGTT

CTGCCGGATCGGTCGCTCAAGATCTTTGGTCTCCACCCGGACCCGATGAAAGAAATTGGA

TCACTTCTTATGGATTCGTTGAGAATGATTCTGATCTAGTTCATGGCCTATTAGAAGTAG

AAGGCGCTTTGGTGGGATCCTCACGGACAGAAAAAGATTGCAGTCAGTTTGATAATGATC

GAGTGATATTGCTTCTTCGGTCCGAACCAAAGAATCCGTTCGATATGATACAAAATGGAT

CTTGTTCTATCGTTGATCAGAGATTTCTATATGAAAAATACGAATCGGAGTTGGAAGAAG

GGGAAGGAGAAGGAGCCCTCGACCCGCAACAGATAGAGGAGGATTTATTCAATCACATAG

TTTGGGCTCCTAGAATATGGCGCCCTTGTGGCAATCTATTTGATTGTATCGAAAGACCCA

ATGAATTGGGATTTCCCTATTGGTGGACCAGGTCATTTCGGGGCAAGCGGATCCTTTATC

ATAAAGAGGATGCGCTTCAAGAGAATGATTCGGAGTTCTTTCAGAGTGGAACCGTGCGGT

ACCAGAGACTAGATAGATCTTCCAAAGAACAAGGCTTTTTTCGAATAAGCCAATTCATTT

GGGACCCTGCAGATCCATTCTTTTTCCTATTCAAAGATCAGCCCTTTGTCTTTTCACGTC

GAGAATTCTTTGCAGATGAAGAGATGTCAAAGGGGCTTATTACTTCCCAAACAAATCCTA

CTACATCTATATATAAACGCTGGTTCATCAAGAATACGCAACAAAACCACTTCGAATTGT

TGATTCATCGCCAGAGATGGCTTAGAACCAATAGTTTATTATCGAATGGATCTTTCCGTT

CTAATACTCTATCCGAGAGTTATCAGTATTTATCAAATCTGTTCCTATCTAACAGAACGC

TATTGGATCAAATGACAAAGGCGTTGTTGAGAAAGAGATGGCTTTTCCCGGATGAAATGA

AACATTGGATTCATGTAACAGGAGAAAGATTTCCCATTCCTTAACCGGAGGCTATGTAGC

CATGAAAGAGGGATTAAGCGGAACAGAATTGACCGGGTCGTGGAAACACTTGTTTCTTCC

ATATTTTGGACCTTAACTCCATGGAACGATATGCTACTACTGAAACATGGAAGAATTGAA

ATCTTAGATCAAAACACTATGGATAGGTGGTATGAACTGCCTAAACAAGAATTCTTGAAC

GGCGAACAACCAGAAGAGCCTATTTATTACTCACTACATCAAACAATTTCCATTAATGAA

ACATGTAAATCCATTGGAAAATCAAAAAAAAAGACGCATGTCCGATGAAAAGGTTGTTGC

TGTCTGCTCCAATAACGAATCATTGGTTGAACTGAATAACTAAATAAAATAGATAGGCCT

TTCTCTTCGTCTCAGGTCGATGGATCTTCTCAATTGGAAGATCTCCTATATGGAGAATAC

ACATTCCAGTTGTCCGAGCCTAATTCGAATTGTTCCGAAGCAAAGATATTCACGGGGGCC

GGTTCGTCCTATTCAGATATTCACGACCGAGAAGTACTGGATTCTGTTTCGGATAGGCCC

TGAAAGGAGAGGGAAGGCTCGAATGCCAACAGACGTCTATTATCTAATTCACCCGACCCG

ATAGTACCCATTTTGGGTACGTCCAGTGCCAAAGTCGCTGAATGGGGAAGTCGCCAATGC

CTAAAACGGACTATGTAATGTACTTTATCTGCTGGGCGACGGGCGGGCATTTTACCAGAG

GTTTCTATTGTATCAATCTACCTGTTGAAGCATATACTCGCGGGGTGGGTGCAGGGCGGA

CGATTTCAAAGCGGACTCCTCATTCATTAGATAGAGAAGATCGCCAAGATTTCGTGATCC

GCTGTCGAACCTATTCCAACAGCCCAGACTCGGATCTGGGGATCGCCGGAAGACTTCGTA

TCAACAGAGGCTCGGTATATCAATATCGATTAGATCCGAGATCTGTTATTGAATTGCTCA

TTCAATGAGCATTTTCAATATTATGCCTTGAAGAGGACTCGAACCTCCACGCTCTTTAGC

ACGAGATTTTGAGTCTCGCGTGTCTACCATTTCACCATCAAGGCATTTTGAAAGTGCATC

GTATTCCATCAATATGATATCTATCTAGTGTGATATATGGAATATATGGCAAAGGTGAAG

TGTTGGGGTATTTCTATCGATCGGCCGTGCCATATATATAGGCCCGATCGGACAGCAAAT

TGCTTCGATTTGAATTATCCGGAGGATACTTTATATATATATCAAAAAGATATACAATCA

AACCTATTTCTCGATTCAATCGAGTTCATATGGTACCCAAATAACGATAGAATAGATATG

TAAAAAGCAGGTCAGGTCCGATTACTCCTATTCCTAATCCTAAATGGAATGTGACGACGT

AGGGATCCATATGGAAACATAGTATCTATTTCTATACGCTCTTCTAATGACTCCTTCTCA

TAATGAGAATGGACATAACCCTATTCCGGCCTGGTCCGGTATGGAATGAACTTATAATCA

TGGAATCGACTCGATCATCCGATTATAAGTTCATTCCATACCGGACCATTCCCATTTTGG

GCGGAATAGATCGACTAATTCTTTTATTCCAGTTAGTAAGAGGGATCTTGAACTAAGAAA

TAGACCCTAGAAGCTAAAAGAGGGTATCCTGAGCAATTGCAATAATGGGGTTCATTGATA

TTCCTGGTATAGTAGATGCTATCACACATAGAATCATACTCAATTCGATGGAATTGTTTG

ATCTTAAAGGGTATCTTCTATAATTTCGCACGTGAGGGGTTATTTCTTGGTTTCGTCCAG

TCATTAATAACTTGATTATTTTTAGATAATAGTAGATAGAAACAACGCTCATAAGGAGTC

CTATTGAAACCAAGAAATATAGGCCTGCCTGCCACCCACACCAGAATAGATAGAGTTTTC

CGAAAAAACCTGCTAGTGGAGGAAGACCTCCTAGGGATAAGAGACATAGGGCTAAAGAGA

GAGCCAAAAAAGGATCTTTTGTGTATAATCCTGCATAATCTCGAATGTTATCAGTTCCGG

TACGTAGACCAAATAAGACAATGCAAGCAAAAGTGCCTAGATTCATGGAGATATAGAACA

GCATATAAGTTATCATGCTTGCATATCCATCATTTGAGTAGCCAACAATTATTCCAATAA

TTACATATCCAATTTGACCTATGGACGAATATGCAAGCATACGTTTCATGCTTGTTTGAG

TAATAGCAATGAGATTCCCCAATATCATGCTAAGAATAGCTAGGATTTCCAGAAGAAGAT

GCCATTCGTTTGATGAGAAATAAAAAGGAATATCGAAAATTCGAGTGGCTAAAGCTGAGG

CAGCTACTTTCGAAGTAACAGAAAGAAAAGCAACGACTGGAGTGGGAGAGTCAGAGTCGA

AAAGAGGATTCCTCACTTCTTTCTCTCATTCAAAACCGTGCATGAGACTTTCATCTCGCA

CGGCTCCTAAGTGATAAAAGAAAGAAGAACTCATCTTCTTTCTTTTTTGATTACCTTCCT

CGCGTATGTATAAGACCGAATCCATTCGATTTCTAAAAAGGATTACTAATCCTTAACTTT

TCGAGGAATCCTTCATCAGTGGTTGTGAATGACCGACTTTTTCAATCTTTTCGTCCTTGG

TTCTGTAGGAGCAGGTCAGAAAGATTGAGAAATAGAACCGTCTGATTTGATTCGTTCTCA

ATAGCCATGAGATAATCATCTTAGGGTGATCCTTTTGTCGACGGATGCTCCTATTACACT

CGTAGTCTCTGAAGGATGAGAACCAACGATGTAGCATCTACATCGAGAATTCAAGTATTG

TATACGTCATTAGCCCGATCCTTTGTAGGAACTACCCGTAATAACGAACTTGCAAAATGG

ATCTGTTTATCATAAAGAGATTCGTTGTTCCTGACCCTGCTTCACCTTAATTGTTATTTG

AACAAAAGGATCACAATAAACTTTTAGTCAAAGTGATGTCTTAGTCCGAGTGGGGATAGC

ATTTCTCTTCTGCATGTCCATGGAGTTTTGAAAAATCCAAACATCTCGGAGATAGATATA

GAGGTAGGAATTTGTCGAACGAACCGCACTCCTTCGTATACGTCAGGAGTCCATTGATGA

AAAGGGGCTGGGGAAAGCTTGAACCCAATTCCTACAGTGATGAATATAAGCGCAATTGAA

ATTCCTGGGGAGTTGTACATTTGTGTATTGATAAGACCATTCACTATTTCTTGAAGCTCG

ATCTCTCCCCCGGATGAACCATATAGCCAAGAGAAACCATGAACCAGAATAGAAGAGCTT

GCCCCACCCATGAGTAAATATTTCGTAGTAGCCTCATTAGACCGTACATCTCTCTTGGTA

TATCCAGATAATAGGTAGGAGCATAAACTGAAACATTCTGGAGCTACAAAGATAGTTATT

AAATCGTTAGCACCACATAAAAACATTCCTCCTAGAGTGGCTGTTAATACGAATAACAGA

AACTCTGTTATAGCCATTTCTGTACATTCAATGTACTCTACGGATAGAGGAATACATAGA

GTTGAACATAGTAAAATAAGAAATTGAAAGATTTCGTTGAAATTGTTCGTTTGGAAATTT

CCCGAAAAGCTAATCATAGGTTCTTCTCTCCATCGGAACAATAGGGCCGTTATGCTCATT

ACTAAACTTGTCGAAGAGATGAAATATAACCAAGGTATATCTTTTTGATCAGAGGTTGAA

TCGATCATCAGAAGAAGAATTAGGCCAAAAATGAGGATACATTCTGGGAAAATGAAACTT

CCATGGAATAGAAGCAAATGAAACGCTTTCATAAAAATTCTCGTAGAATCGAGAATGAAG

TTTTCATTCTGTACATGCCAGATCATGAATTAGTAACTGCATCCAATCCCCGAAAAAGTC

CAATTGTTTCGAACTTTCGATTTTTGGAATGGGATATTTACGGAATCCCCAAGTAGAGGA

TCAAACCTTATTCCATGGTATTTCCATGAGATTCCTCTTTCTTATTCTTAATTAAGCAAG

CCCCCGAGAGCACTTAGTTGATCCATGATTTATCTTTCATCTTTCCTTTTCGTTTGTTTC

GAGAAAGATATCGATCAATTCCGATTCTTTCTTTTTCTATTGATTCTTTTCCGATCGAGA

TGTATGGATCCATGGATCTATGTGTCTATATAGATCCTGTTCATGGATTAACGAAAATGT

GCAAAAGCTCTATTTGCCTCTGCCATTCTATGAGTCTCTTCCTTTTTGCGTATGGCATCG

CCACTCCCCTTGGCAGCATCCACTAATTCGGAACTTAATTTGAAAGCCATATTTCGACCC

GGACGCTTTCGGGATGCCCATAATAACCAACGAATGGCAAGTGCTTTTCCTTGTGTAGAT

CCTATTTCAATGGGAACTTGATGAGTCGATCCGCCTACACGTCTTGCTTTTACTGCTATA

TCGGGAGTTACTCCATGTATTGCTTGACGTAAAACAGATAGTGGATTTGTTTCTGTCTTT

TGTTGAATTTTTTTCACGGCTCGATAGATAATTTGATAAGCCAATGATTTTTTTCCGTGT

TTCAGAATACGGTTAACCAACATGTTAACTAATCGATTACGATAAATTGGATCGGATTTT

GCAGTTTTTTCTTCTGTAGTACCTCGACGTGACATGAGCGTGAAAGGGATTCAAGAATCG

GTTTTCTTTTTATAAGGGCTAAAAACGAATCACTTATTTTGGCTTTTTGACCCCATATTG

TAGGGTGGATCTCGAAAGATATGAAAGATCTCCCTCCAAGCCGTACATACGACTTTCATC

GAATACGGCTTTCCACAGAATTCTATATGTATCTATGAGATCGAGTATGGAATTCTGTTT

ACTCACTTCAAATTGAGTATCCGTTTCCCTCCCTTTCCTACTAGGATTGGAAATCCTGCA

TTTTACATATCCATACAATCGAGTCCTTGGGTTTCCGAAATAGTGGAAAAAGAAGTGCTT

CGAATCATTGCTATTTGACTCGGACCTGTTCTAAAAAAGTCGAGGTATTTCGAATTGTTT

CTTGACACGGACAAAGTAAGGGAAAACCTCTGAAATGATTTCAATACTGGACCTTGGACA

TATAATAGTTCCGAATCGAATCTCTTTAGAAAGAAGATCTTTTGTCTCATGGTAGCCTGC

TCCAGTCCCCTTACGAAACTTTCGTGATTGGGTTAGCCATACACTTCACATGTTTCTAGC

GATTCACATGGCATCATCAAATGATACAAGTCTTGGATAAGAATCTACAACGCACTAGAA

CGCCCTTGTTGACGATCCTTTACTCCGACAGCATCTAGGGTTCCCCGAACAATGTGATAT

CTCACACCGGGTAAATCCTTAACCCTTCCTCCTCTTACTAAGACTACAGAATGTTCTTGT

AAATTATGGCCAATTCCAGGTATATAAGCAGTGATTTCAAATCCAGAGGTTAATCGTACT

CTGGCAACTTTACGTAAGGCAGAGTTTGGTTTTTTGGGGGTGATAGTGGAAAAGTTGACA

GATAAGTCACCCTTACTGTCACTCTACAGAACCGTACATGAGATTTTCACCTCATACGGC

TCCTCGTTCAATTCTTTCGAAATAATGGGATCCTTTTCCTCGTTCGAGAATCTCCTCCCT

TCTTCCACTCCGTCCCGAAGAGTAACTAGGACCAATTCAGTCGCGTTTTCATGTTCCAAT

TGAACACTTTCCATACATTTTTTTTGGATTATGATCAAAGAATTCTTCTTTTTACCAAAA

TAGGCGGATCAGATCACGATCTTATAATAAGAACAAGGGATCCTTAAGAACAAGAGATCT

TTCTCAATCAATCCCTTTGCCCCTCATTCTTCGAGAATCAGAAAAATCCTTTTCGAGTTT

GAATTTGTTCATTTGGAATCTGGGTTCTTCTACTTATACTTTTACTTTACTTAACTTAAC

TTAATTTACATTTACTTATACTTATACTTAATTTCATATTTACTTAATTTCATATTTCAT

ATTTCATATTTCATTTCTATTTATTTACTTACTTTATCTTTTTATCTTTATTTTTTTTTT

AGTAGTTTCTTTATTTCGATTTTCTTTCCCTCTCTTTTCTTTTTATTCCCTTCTATCATT

CCTTAAGTCCCATAGGTTTTATCCTGTAGAATCTCACCCATTTTCTCATCGAGCGAGGGG

TACGAAATCAATCAGATTCATTTTTCGATCAAAAGTACTATGTTAAATCTTCGGTTTCTC

CTCTTCCTCTATCCCTATCCGATAGGTACAGCGTTTGAATCAATAGAGAACATTTTCTTC

TGTATGAATCTATATTATTACATTCCTTCCCGATACCTCCCAAGGAAAATCCCGAATTGG

ATCCCAAATTGACGGGTTGGTGTGAGCTTATCCATGCGGTTATGCACTCTTCGAATAGGA

ATCCATTTTCTGAAAGATCCTGGCTTTCGTGCTTTGGTGGGTCGTCCGAGATCCTTTCGA

TGACCTATGCTAAGGGATATCTATATGATCCGATCGATTGCGTAAGGCCCGCGGTAGCAA

CAGAACCGGGGAAAATATACAGAAAAGACAGTTCTTTTCTATTATATTAGTATTAGTTAG

TGATCCCGGCTCGGTGAGTCCTTTCTTCCGTGATGAACTGTTGGCACCAGTCCTACATTT

TGTCTCTGTGGACCGAGGAGGAAGGGGGCTCCGCGGGAATAGGATTGTACCATGAGAGAA

GCGAGGAGGTCAACCTGTTTCAAATAAATATACAACATGGATTCTGGCAATGCAATGGAG

TTGGACCCTCATGTCGATCCGAATGAATCGGTCTTTCCACGGAGGTTAATCTTTGCCTGC

TAGGCAAGAGGATAGCAGGTTAGAAATTCTGTCTCGGTCGGACATGTATTTCTATTTCTA

TTACTATATTACTATTACTATATTACTATGAAATTCATAGAAATTCATAAATGAAGTAGT

TAATGGTGGGGTTCTCATTATCCTTTTTGTAGTGACGAATCTTGTATGTGTTCCTAAGAA

AAGGAATTTGTCCATTTCTCGAGGTCTCAAAAGGGCGTGGAAACAAATAAGAACTCTTGA

ATGGAAATTGAAAAGAGATGTAGCTCCCGTTCCGAAGGAACTGGTAAGATCTTTGGCGCA

ACGCAAGAAGAAGGGGTTGATCCGTATCATCTTGACTTGGTTCTGCTTCCTCTCTTTTTT

AACAATACCTAATCAGGTTCTTCTTCCACCAGTATCGAATAGAACATGCTCAAGAAAATC

TTCTTCCTGTAAAACCTGCTCGAGTTAGATCGGGAAAATCAATCGTACGGATTTTATGAA

ACCATGTGCTATGGCTCTATGGCTCGAATCCGTAGTAAATCTTATTTCCGATAGGAGCAG

TCGACAATTGAATCCAATTTTTCCATTCTTTTCGTATCCGTAATAGTGCGAAAAGAAGGC

CCGGCTCCAAGTTGTTCAGGAATAGTGGCGTTGAGTTTCTCGACCCTTTGACTTAGGATT

AGTCAGTTCTATTTCTCGATGGGGGCGGGGAAGGGATATAACTCAGCGGTAGAGTGTCAC

CTTGACGTGGTGGAAGTCATCAGTTCGAGCCTGATTATCCCTAAACCCAGTGTAAGTTTT

TCTATTTTGACTTGCTTCCCCGCCGTGATCGAATGAGAATGGATAAGAGGCTCGTGGGAT

TGACGTGAGAGGGCAGGGATGACTATATTGCTGGGAGCGAACTCCAGTATAATATGAAGT

GTATGGATACAAGTTATGCCTTGGAATGAAAGACAATTCCGAATCCACTTTGTCTACGAA

CAAGGAAGCTATAAGTAATGCAACTATGAATCTCATGGAGAGTTCGATCCTGGCTCAGGA

TGAACGCTGGCGGCATGCTTAACACATGCAAGTCGGACGGGAAGTGGTGTTTCCAGTGGC

GGACGGGTGAGTAACGCGTAAGAACCTGCCCTTGGGAGGGGAACAACAGCTGGAAACGGC

TGCTAATACCCCGTAGGCTGAGGAGCAAAAGGAGGAATCCGCCCGAGGAGGGGCTCGCGT

CTGATTAGCTAGTTGGTGAGGCAATAGCTTACCAAGGCGATGATCAGTAGCTGGTCCGAG

AGGATGATCAGCCACACTGGGACTGAGACACGGCCCAGACTCCTACGGGAGGCAGCAGTG

GGGAATTTTCCGCAATGGGCGAAAGCCTGACGGAGCAATGCCGCGTGGAGGTAGAAGGCC

CACGGGTCGTGAACTTCTTTTTTCGGAGAAGAAGCAATGACGGTATCTGAAGAATAAGCA

TCGGCTAACTCTGTGCCAGCAGCCGCGGTAAGACAGAGGATGCAAGCGTTATCCGGAATG

ATTGGGCGTAAAGCGTCTGTAGGTGGCTTTTCAAGTCCGCCGTCAAATCCCAGGGCTCAA

CCCTGGACAGGCGGTGGAAACTGCCAAGCTGGAGTACGGTAGGGGCAGAGGGAATTTCCG

GTGGAGCGGTGAAATGCGTAGAGATCGGAAAGAACACCAACGGCGAAAGCACTCTGCTGG

GCCGACACTGACACTGAGAGACGAAAGCTAGGGGAGCGAATGGGATTAGATACCCCAGTA

GTCCTAGCCGTAAACGATGGATACTAGGCGCTGTGCGTATCGACCCGTGCAGTGCTGTAG

CTAACGCGTTAAGTATCCCGCCTGGGGAGTACGTTCGCAAGAATGAAACTCAAAGGAATT

GACGGGGGCCCGCACAAGCGGTGGAGCATGTGGTTTAATTCGATGCAAAGCGAAGAACCT

TACCAGGGCTTGACATGCCGCGAATCCTCTTGAAAGAGAGGGGTGCCTTCGGGAACGCGT

ACACAGGTGGTGCATGGCTGTCGTCAGCTCGTGCCGTAAGGTGTTGGGTTAAGTCCCGCA

ACGAGCGCAACCCTCGTGTTTAGTTGCCACCATGGGGTTTGGAACCCTGAACAGACTGCC

GGTGATAAGCCGGAGGAAGGTGAGGATGACGTCAAGTCATCATGCCCCTTATGCCCTGGG

CGACACACGTGCTACAATGGCCGGGACAAAGGGTCGCGATCCCGCGAGGGTGAGCTAACT

CCAAAAACCCGTCCTCAGTTCGGATTGCAGGCTGCAACTCGCCTGCATGAAGCCGGAATC

GCTAGTAATCGCCGGTCAGCCATACGGCGGTGAATTCGTTCCCGGGCCTTGTACACACCG

CCCGTCACACTATGGGAGCTGGCCATGCCCGAAGTCGTTACCTTAACCGCAAGGAGGGGG

ATGCCGAAGGCGGGGCTAGTGACTGGAGTGAAGTCGTAACAAGGTAGCCGTACTGGAAGG

TGCGGCTGGATCACCTCCTTTTCAGGGAGAGCTAATGCTTGTTGGGTATTTTGGTTTGAC

ACTGCTTCACATCCAAAAAGAAAAGAAGCGAGCTACGTCTGAGCTAAGCTTGGAGATGGA

AGTCTTTTTTCGTTTCTCGACGGTGAAGTAAGACCAAGCTCATGAGCTTATTATCCTAGG

TCGGAACAAGTTGATAGGATCCCCCCTTTTTACGTCCCCATGCCACTCCCGTGTGGCGAC

ATGGGGGCGCAAAAAAAAAAGGAAAGAGAGGGATGGGCTTTCTCTCTTGGCATAGCGGGC

CCCCGTGGGAGGCCCGCAGGGCTATTAGCTCAGTGGTAGAGCGCGCCCCTGATAATTGCG

TCGTTGTGCCTGGGCTGTGAGGGCTCTCAGCCACATGGATAGTTCAATGTGCTCATCAGC

GCCTGACCCGGAGATGTAGATCATCCAAGGCACAGTAGCATGGCGTTCTCCTCCTGTTCG

AACCGGAGTTTTAAACCAAACTTCTCCTCAGGAGGATAGATGGGGCGGTTCAGGTGAGAT

CCAATGTAGATCCAACTTTCTATTCACTCGTGGGATCCGGGCGGTCCGGGGGGGACCACC

ACGGCTCCTCTCTTCTCGAGAATCCATACATCCCTTATCAGTGTATGTACAGCTATCTCT

CGAGCACAGGTTTAGGTTCGGCCTCAATGCGAAAATGGAGCACCTAACAACGCATCTTCA

CAGACCAAGAACTACGAGATCGCCCCTTTCATTCTGGGGTGACGGAGGGATCGTACCATT

CGAGCCTTTTTTTTCATGCTTTTCCCGGAGGTCTGGAGAAAGCAGCAATCAATAGGATTT

CCCTAATCCTTCCTTCCCGAAAGGAAGAACGTGAAATTCTTTTTTTTTTCCTTTCCGCAG

GGACCAGGAGATTGGATCTAGCCATAAGAAGAATGCTTGGTATAAATAAACGGACTTCTT

GGTCTTCGACCCCCTCAGTCACTACGAACGCCCCCCGATCAGTGCAAAGGGATGTGTCTA

TTTATCTATCTCTTGACTCAAAATGGGGGCAGGTTTGAAAAAGGATCTTAGAGTGTCTAG

GATTGGGCCAGGAGGGTCTCTTAACGCCTTCTTTTTCTTCTCATCGGAGTTATTTCACAA

AGACTTATCATGGTAAGGAAGAAGGGGAGAACAAGCACACTTGGAGAGCGCAGTACAACG

GAGAGTTGTATGCTGCGTTCGGGAAGGATGAATCGCTCCCGAAAAAGAATCTATTGATTC

TCTCCCAATTGGTTGGATCGTAGGTGCGATGATTTACTTCACGGGCGAGGTCTCTGGTTC

AAGTCCAGGATGGCCCAGCAGCGCCGGGGAAAAGAATCGAAGAAGCATCTGACTCCTTCA

TGCATGCTCCACTCGGCTCGGGGGGATATAGCTCAGTTGGTAGAGCTCCGCTCTTGCAAT

TGGGTCGTTGCGATTACGGGTTGGATGTCTAATTGTCCAGGCGGTAATGATAGTATCTTG

TACCTGAACCGGTGGCTCACTTTTTCTAAGTAATGGGGAAGAGGACCGAAACATGCCACT

GAAAGACTCTACTGAGACAAAGATGGGCTGTCAAGAACGTAGAGGAGGTAGGATGGGCAG

TTGGCCAGATCTAGTATAGATCGTACATGGACGATAGTTGGAGTCGGCGGCTCCCCTAGG

GTTCCCTCATCTGGGATCCCTGGGGAAGAGGATCAAGTTGGCCCTTGCTAACAGCTTGAT

GCACTATCTCCCTTCAACCCTTTGAGCGAAATGTGGCAAAAGGAAGGAAAATCCATGGAC

CGACCCCATCGTCTCCACCCCGTAGGAACTACGAGATCACCCCAAGGACGCCTTCGGCAT

CCAGGGGTCACAGACCGACCATAGACCCTGTTCAATAAGCGGAACGCATTAGCTGTCCGC

TCTCCGGTTGGGCAGTAAGGGTCGGAGAAGGGCAATCACTCATTCTTAAAACCAGCATTC

TTAAGACCAAACCAAAGAGTCGGGCGGAAGCGGAAAAAGGGGGGAGAGCTCTCTGTTCCT

GGTTCTCCTGTAGCTGGATCCTCCGGAACCACAAGAATCCTTAGAATGGGATTCCAACTC

AGCACCTTTTGAGAAGAGTTGCTCTTTGGAGAGCACAGTACGATGAAAGTTGTAAGCTGT

GTTCGGGGGGGAGTTATTGTCTATCGTTGGCCTCTATGGTAGAATCAGTCGGGGAGGCCC

GAGAGGCGGTGGTTTACCCTGTGGCGGATGTCAGCGGTTCGAGTCCGCTTATCTCCAGCT

CGTGACCTTAGCTGATACAAAGGTATATATATGATAGCACCCAATTTTTCCGATTCGGCG

GTTCGATCTATGATTTCTCATTCATGGACGTTGATAAGATCCTTCCATTTAGCAGCACCT

TAGGATGGCATAGCCTTAACGTTAATGGCGAGGTTCAAACGAGGAAAGGCTTACGGTGGA

TACCTAGGCACCCAGAGACGAAGAAGGGCGTAGTAAGCGACGAAATGCTTCGGGGAGTTG

AAAATAAGCATAGATCCGGAGATTCCCGAATAGGTCAACCTTTCGAACTGCTGCCGAATC

CATGGGCAGGCAAGAGACAACCTGGCGAACTGAAACATCTTAGTAGCCAGAGGAAAAGAA

AGCAAAAGCGATTCCCGTAGTAGCGGCGAGCGAAATGGGAGCAGCCTAAACCGCGAAAAC

GGGGTTGTGGGAGAGCAATACAAGCGTCGTGCTGCTAGGCGAAGCGGTGGAGTACTGCAC

CCTAGATGGCGAGAGTCCAGTAGCCGAAAGCATCACTAGCTTACGCTCTGACCCGAGTAG

CATGGGGCACGTGGAATCCCGTGTGAATCAGCAAGGACCACCTTGCAAGGCTAAATACTC

CTGGGTGACCGATAGCGAAGTAGTACCGTGAGGGAAAGGTGAAAAGAACCCCCATCGGGG

AGTGAAATAGAACATGAAACCGTGAGCTCCCAAGCAGTGGGAGGAGCAATCTCTGACCGC

GTGCCTGTTGAAGAATGAGCCGGCGACTCATAGGCAGTGGCTTGGTTAAGGGAACCCACC

GGAGCCGTAGCGAAAGCGAGTCTTCATAGGGCAATTGTCACTGCTTATGGACCCGAACCT

GGGTGATCTATCCATGACCAGGATGAAGCTTGGGTGAAACTAAGTGGAGGTCCGAACCGA

CTGATGTTGAAGAATCAGCGGATGAGTTGTGGTTAGGGGTGAAATGCCACTCGAACCCAG

AGCTAGCTGGTTCTCCCCGAAATGCGTTGAGGCGCAGCAGTTGACTGGACATCTAGGGGT

AAAGCACTGTTTCGGTGCGGGCCGCGAGAGCGGTACCAAATCGAGGCAAACTCTGAATAC

TAGATATGACCCCCAAAAAGGGGGTAAAGGTCGGCCAGTGAGACGATGGGGGATAAGCTT

CATCGTCGAGAGGGAAACAGCCCGGATCACCAGCTAAGGCCCCTAAATGACCGCTCAGTG

ATAAAGGAGGTAGGGGTGCAGAGACAGCCAGGAGGTTTGCCTAGAAGCAGCCACCCTTGA

AAGAGTGCGTAATAGCTCACTGATCGAGCGCTCTTGCGCCGAAGATGAACGGGGCTAAGC

GATCTGCCGAAGCTGTGGGATGTCAAAATGCATCGGTAGGGGAGCGTTCCGCCTTAGATG

AAAGCGCCCGCGTGAGCAGGTGTGGACGAAGCGGAAGCGAGAATGTCGGCTTGAGTAACG

CAAACATTGGTGAGAATCCAATGCCCCGAAAACCTAAGGGTTCCTCCGCAAGGTTCGTCC

ACGGAGGGTGAGTCAGGGCCTAAGATCAGGCCGAAAGGCGTAGTCGATGGACAACAGGTG

AATATTCCTGTACTACCCCTTGTTGGTCCCGAGGGACGGAGGAGGCTAGGTTAGCCGAAA

GATGGTTATCGGTTCAAGGACGCAAGGTGACCTTGCTTTTTAAGGGCGGGGTAAGAGGGG

GTAGAGAAAATGCCTCGAGCCAATGTCCGAGTACCAGGCGCTACGGCGCTGAAGTAACCC

ATGCCATACTCCCAGGAAAAGCTCGAACGACCTTCAACAAAAGGGTACCTGTACCCGAAA

CCGACACAGGTGGGTAGGTAGAGAATACCTAGGGGCGCGAGACAACTCTCTCTAAGGAAC

TCGGCAAAATAGCCCCGTAACTTCGGGAGAAGGGGTGCCTCCTCACAAAGGGGGTCGCAG

TGACCAGGCCCGGGCGACTGTTTACCAAAAACACAGGTCTCCGCAAAGTCGTAAGACCAT

GTATGGGGGCTGACGCCTGCCCAGTGCCGGAAGGTCAAGGAAGTTGGTGACCTGATGACA

GGGGAGCCGGCGACCGAAGCCCCGGTGAACGGCGGCCGTAACTATAACGGTCCTAAGGTA

GCGAAATTCCTTGTCGGGTAAGTTCCGACCCGCACGAAAGGCGTAACGATCTGGGCACTG

TCTCGGAGAGAGGCTCGGTGAAATAGACATGTCTGTGAAGATGCGGACTACCCGCACCTG

GACAGAAAGACCCTATGAAGCTTTACTGTTCCCTGGGATTGGCTTTGGGCCTTTCCTGCG

CAGCTTAGGTGGAAGGCGAAGAAGGCCCCCTTGCGGGGGGGCCCGAGCCATCAGTGAGAT

ACCACTCTGGAAGAGCTAGAATTCTAACCTTGTGTCAGGACCTACGGGCCAAGGGACAGT

CTCAGGTAGACAGTTTCTATGGGGCGTAGGCCTCCCAAAAGGTAACGGAGGCGTGCAAAG

GTTTCCTCGGGCCGGACGGAGATTGGCCCTCGAGTGCAAAGGCAGAAGGGAGCTTGACTG

CAAGACCCACCCGTCGAGCAGGGACGAAAGTCGGCCTTAGTGATCCGACGGTGCCGAGTG

GAAGGGCCGTCGCTCAACGGATAAAAGTTACTCTAGGGATAACAGGCTGATCTTCCCCAA

GAGTTCACATCGACGGGAAGGTTTGGCACCTCGATGTCGGCTCTTCGCCACCTGGGGCTG

TAGTATGTTCCAAGGGTTGGGCTGTTCGCCCATTAAAGCGGTACGTGAGCTGGGTTCAGA

ACGTCGTGAGACAGTTCGGTCCATATCCGGTGTGGGCGTTAGAGCATTGAGAGGATCTTT

CCCTAGTACGAGAGGACCGGGAAGGACGCACCTCTGGTGTACCAGTTATCGTGCCCACGG

TAAACGCTGGGTAGCCAAGTGCGGAGCGGATAACTGCTGAAAGCATCTAAGTAGCAAGCC

CACCCCAAGATGAGTGCTCTCCTATTCCGACTTCCCTGGAGCCTCCGGTAGCACAGCCGA

GACAGCGACGGGTTCTTCTCTGCCCCTGGGGGGATGGAGCGACAGAAGTCTTGAGAATCC

AAGATAAGGTCACGGCGAGACGAGCCGTTTATCATTACGATAGGTGTCAAGTGGAAGTGC

AGTGATGTATGCAGCTGAGGCATCCTAACAGACCGAGAGATTTGAACCTTGTTCCTACAT

GACCCGATTAATTCGATCAGGCACTCGCCATCTATTTTCATTGTTCAACTGTTTGACAAC

ATGAAAAAAACCAAAAGCTCTGCCCTCCCTCTCTATCTATCCAAGGGATGGAAGGGCAGA

GGCCTTTGTTTGGTGTCCCTTCCAGTCAAGAATTGGGGCCTCACAATTACTAGCCAATAG

GCTTTTCTCTCATGCCTTTCTTCGTTTATGGTTCGATATTCTGGTGTCCTAGGCGTAGAG

GAACCACACCAATCCATCCCGAACTTGGTGGTTAAACTCTACTGCGGTGACGATACTGTA

GGGGAGGTCCTGCGGAAAAATAGCTCGACGCCAGGATGATAAAAAGCTTAACACCTATTA

TTCATTTTTTTTTTTTTTCTTAAAAAAAAAAAAATGAAAAATGAAAAGGTCGTCTTATTC

AAAACCCCAATTATGACACCCCTTCTCCCCCACTTCACACCTCGGAACGCACTGTTCTTA

TATAGAGAAACGCGCTTTCATATCTTCTTAACCCGAAATGAAATGGCTGAGGGGAGGAAA

GGTTCCTTTTGAGGGTACTCCCGGGAACAGATCCAGTGGAGATGGGGTGGGGCCTGTAGC

TCAGAGGATTAGAGCACGTGGCTACGAACCACGGTGTCGGGGGTTCGAATCCCTCCTCGC

CCACAATCGGCCAAAAAGAACCTTTCCTTTACCTATGTGTGGTGGGTAGGAAAATCATGA

TCGGGATAGCGGATGCAAAGCTATAGAACTTGGGTCTTTTGTCGAAATGGAATGGCCTTA

TTATTTATCGTGAAGGAAAAATGCGTATAGTATGCTATGCAGGGGTCGAATCATTTGTGT

TTTACTCCCCGTAACTCTTCCTCAGCCAGGCTTGGGCAGAATAGCAGAGCAAATACAAGT

ATTATTTAGTATTAGTAGCATAGCAAAGCAAAAATGCATTCCTCGTCATTAATATGTTTG

CTCGCGAGAATTGTGGCCTCTCGGGAGAATCGATGACTCCACCTTTGATGCACTGCCAGT

ACATCCATCTGAGAATTCTTAATTGGCTAGTTGTAAATAGCCCCGTCGGGGCTATGGAAC

CAAGGATTATCCCGGATCGGGGTATTGACGGTGATTCTCAAATCTCGCAGAACAGAATGT

GATACGATGAGATAGAATGCAATAGAAACAAAGACAAGCAACGGGTTACCTACTCCTAAC

GGTCAAAGCGAGCCTTTGCATTCTGAATTCAAAATGAAAGAATTCAGAATTCATCAAATC

TCCCCAAGTAGGATTCGAACCTACGACCAGTCAGTTAACAGCCGACCGCTCTACCACTGA

GCTACTGAGGAATAACAGGAGATTAGATCTCATAGAGTTCAACTCCCGTTCTCAACCCAT

GACCAATATGAGCCCGAAGCTCCCTTCGTAACTCCCGTAACTTCTTCGTAGTGGCCCCCT

TCCATGCCTCATTTCATAGGGAACCTAAAAGTGGCTCTATTTCATTATATTCCATCCATA

TCCCAATTCCATTCATTTAATATCCCTTTGGCGTCCTTGACATCAGAGATGTCATTCAAT

CGAAGAAGATGGTATCATTGACATAAGAGATGTCATTTCTAGTCTATCTGTTTCCATATA

TGGAAAGTTAAGAAATCATCATATAATAATCGAGAAATTGCAATAGAAAAGAAAAAAGGG

AGGTTTGTGATGATTTTGAAATCTTTTCTACTAGGTAATCTATTATCCTTATGCATGAAG

ATAATCAATTCGGTCGTTGTGGTCGGATTCTATTATGGATTTCTGACCACATTCTCCATA

GGGCCCTCTTATCTCTTCCTTCTCCGAGCTCAGGTTATGGAAGAAGAAACCGAGAAGGAG

ATATCATCAACAACTGGTTTTATTACGGGGCAGCTCATGATGTTTATATCGATCTATTAT

GCGCCTCTGCACCTAGCATTGGGTAGACCTCATACAATAACTGTCCTAGTTCTACCGTAC

CTTTTGTTTCATTTCTTCTGGAACAATCACAAACACTTTTTGGATTATGGATCTACTACC

AGAAATTCCATGCGTAATCTCAGCATTCAATGTGTATTCCTGAATAATCTCATTTTTCAA

TTATTCAACCATTTCATTTTACCAAGCTCAACGTTAGCCAGATTAGTCAACATTTATCTG

TTTCGATGCAACAATAAGATCTTATTTGTAACAAGTAGTTTTGTTGGTTGGTTAATTGGT

CACATTTTATTCATGAAATGGGTTGGATTGGTATTATCCTGGATACGGCAAAATCATTCT

ATTCAATCTAATGTACTTATTCGATCTAATAAGTACCTTGTGTCAGAATTGAGAAATTCT

ATGGCTCGAATCTTTAGTATTCTCTTATTTATCACCTCTGTCTACTATTTAGGCAGAATG

CCATCGCCTATTGTTACTAAGAAACTGAAAGAAACCTCAGAAATGGAAGAAAGAGGGGAA

AGTGAGGAAGAAACAGATGTAGAAATAGAAACGACTTCCGAAACGAAGGGGACTAAACAG

GAACAAGAGGGATCCACCGAAGAAGACCCTTCTCTTTGTTCGGAAGAACAGGAGGATCCG

GACAAACTAGATGAAACGGAAGAGATCCGAGTGAATGGAAAGGAAATACTTAAAGATGAG

GAAGATAAAGACCTCTTTTGGTTTGAAAAACCTCTTGTAACTCTTCTTTTCGACTATAAG

CGATGGAATCGTCCATTACGATATATAAAAAATGATCGATTTGAAAATGCTGTAAGCAAT

AAAATGTCACAATATTTCTTTCACACATGTCCAAGTGATGGAAAAAAAATCATATCTTTT

ACATATCCACCCAGTTTGTCGACTATTGAGGAAATAATACAAAAAAAGATGTCTTTATAC

ACGACAGAAAAAGGATCCCCAGAGGACCTGTATAATAATTGGGTTTATACAAATAAAAAA

AAAAAAAAAAACTTGAGTAATGAGTTAATAAATCGAATAGAAGCTATAGAGAAGGGGTCT

GTTTCAATAGATGTACTTGAAAAAAGGATCCGATTGTGCCATGATGAGAATGAACAAAAA

TGCTTGCCTAAATGGTATGATCCGTTTTTCAACGGACCATATCGTGGAACAATTACAAAG

GTTGATTCACAAAACAAAAAAGTAGTAAATTCAAAAGAAGAACATCCGTTTGAAGAGAAA

AGAAATACTTTTCATTTGAATAGAAAATTGTTATCAACAGACATTGATAATCCTTTTACC

TCAATCGGTGAATTCGACGACGAATCGGCGTCTAGTTTTCATTTAAAAGAAATTTTTTTA

GATAACGAAGAAAAAAGTCTTGATTCAGAAAATAAAAAAAAAAACTTCAAACAACTCTTT

GATGCAATTAAAAACGATTCAAATGATCAAGCGCTTAGAAAACATGAAATTGAGATAGAA

AAAATGAACAAAAAACTTCCCCGGTGGTCATATAAATTGATCGATGATTTGGAACAACAG

GAAGAAGAAAATCCGGAGGAATCACCGGGTAATCCTGGTATTCGTTCAAGAAAAGCTAAA

CGTGTAGTAATTTATACTGATAAAGATCAGAATATACATACCGATACTAGTACCGATAGT

GATCAAGCAGAAGAAATAGCTTTGATACGTTACTCACAACAATCGGATTTTCGTCGTGAT

CTAATCAAAGGCTCGACGCGGGCTCAAAGGCGTAAAACCCTTATTTTGGAAATGTTTCAA

GCAAACGCGCACTCCCCACTTTTTTTGGACAGACTTAATAAAACATCTTTTTCTTTTGAT

ATCTCCAAAATGAGTATGGATAAAGGGCTGGAATTCAAAACCGCCAATTCAGAAAATGCT

GAAAAAATGGAGGCAGCTATTAAAGAAAAAACAGATAAAAGTGATCGATTAGCAATAGCG

GAAACTTGGGATTCTGTTACATTTGCTCAACCCATACGGGGTTGTATGTTAGTAGCCCAA

TCGATTGTTAGAAAATATATAATTTTGCCCACACTGATAATAGGTAAAAACCTCAGCCGT

ATCTTATTATGGCAATCGCCAGAGTGGGACGAAGATTTTCAAGAATGGAAAAAAGAAATG

CACATTAAATGCACCTATAATGGTGTTCAATTATCAGAAACAGAATTTCCCCAAGCCTGG

TTGACAGATGGTATTCAGATAAAAATCCTGTTTCCTTTTTCACTAAAGCCTTGGCATAAA

TCTAAACAAACATCTCTTCAAGTGGATCCAATAAAAAAGAAAGTAAAAAAACAAAATTTT

TGTTTTTTAACGGTATGGGGTCGCGAAACTGAACAACCCTTTGGCCCTCCCCGAAAAAAC

CCTCCTTTTTTTAAACCCATTTTAAAAGAATTAAAAAAAGAAATGAGAAATAGATTTTTT

ATAGTCCCAAAAACATTAAAAAATGGAACAAAAGGATTTATGAAAGTTGAAAAAGAAAAG

ACAACATCGATTATCCAAATCGTTTTCTTTTTAAAAAGAAAAATGAAAGAATTTATATTT

GTATTTGTAAAAGTGAATCCTACTAAAGGAGTAGGATCACAGAAAATATATGAATCGAAC

GAAAATGAAAAGAATTCTATCCTAAGTAATAAGATTACCTATGAATCAAACATTCAAAAA

AAATCGACGATAGATTTGACAAATTATTCATTGACAGAAAAAAAAATAAAAGATCTGTCC

GATCATACAATCATAATCAGAAATCAAATCGAAAGAGTTGCAAAGGATAAAAAAAGAATA

TTTCTAATTTCAGATAGAAATCCTAGTCCTGACGAAATGGGTTGGTATGAAAAGAAGAAA

GGGCGAGAATCCAAAAAAGGTATTTGGCAGATATTAAAAAAGATATTCAAAAAAAGAAGT

ACGAGATTAATACGTAAATGGTCTTATTTTTTTAAATCTTTGATTCAAAAAATATACATG

GAAACTCTTCTATTTACCATTATTAGTACCAAAGACTATGCAAAATTTTTCATTGAATCG

ATAAAAAAATCTCTCAATAAACATATTTATAATGATGAAAAAGACAAAAGGGTAATTGAT

GAAACAAATCAAAACACAATTGAATTTATTTCAACTATAAATAGATCATTTTCTAATATT

ACCAATATTATTTATAATAGTAATAAGAATTCACTGACTTATTACGACTTATTTTCTTTA

TCTCAAGCATATGTATTTTTAAAATTATCACAAACTCAAGTTATTAACTTATACCGCTTG

GGATCTTTACTTCAATATCACGGAGCATCCACTCTTATCAAGAAGAAAATAAAGGATTAC

TTAGGAAGCCAGGGAATATTTGATTTCAAATTAAAACAGAAGAAACTTCTGAATTCTGGA

ATAAATGACTGGAAAAATTGGTTAAGGAGTCATTATCAATATAATTTACCTAAGATTCGA

TGGTCGAGAATAGTACCACAAAAATGGCGAAATAGAATCAATCAACGTCGTACAATTAAA

AAGAAAGGCTCCAAATTTTCGCATTCATATGCAAAAGAAAAAGACCAATTCATTTATTAC

AAAAAACAAAAGAATTTTAGAATAAATTCATTGACAAATCAAAAAGAAAAATTAAAAAAA

AATTACAGATATGATCTTTCATCATACAAATATCTTTATTATGAAGATGTGAAGAACTCA

CTACCCATTTCTCGGTCTCCATTCCAAATAAGGGGAGGCCGAGAAATTTCGTATAATTAC

AATAATTACAACAGACCAAAATCCGAATCATTCTTTTATGTACCAGAAAAAATAGCCATT

GATGATTATTTAATAATGGGAGAGTATGTTTTTAATACAGGTAAAAATCTGGATAGAAAA

TATTTGGATTGGGGAATTCTCAATTTGTGTCTTAAAAAAAATATCAATATTGAATACTGG

ACTCATATGGATACCAGGACCTTTAGTCAAGACACTAGGACTCGATCCAATGATTATCGA

ATTGTTGAGAACAAAAATCTTTTTTTTCTTAGGATTCATCAGGAAATCAACCCATCCAAT

CAAAAAAAAAAACTTTTTGATTGGATGGGAATGAATGAGGAAATGCTATATTATTCCATA

TCGAGTATCGAGCCTTGGTTCTTTCTAGAATTTGTACTACGATATGATACATATAAGATG

AAACCTTGGATCATACCGATCAAATCACTTTTTTTTAATTTTTATGGGAAGGAAAGCATT

AGTAAAAACATCAACGTAAATCGAAAGGCAAATCATGATACGTCATCTAATTTTAAAAAG

CACCCTGGATTCAAAAATATAAATCAAGAAGAAAAAGAGGGGCAGGGACAAGAAGCTCTT

CGATCAAATGCACAAAACCAACAAAAATCTTTTATTGAAAAGGATTACACAGAATCAGAC

ATTCAAAAGCCTAGAAAGAAAGCTCAATCCAAAAATAATAAGGAAACGGGACTCGACTCT

TTTTTGAAAAAATATTTGCTTTTTCAATTAAGATGGAATAACTCTTTGAATCAAAAAGTT

ATTGACAATATCAAAGTGTATTCTCTCCTGCTTAGATTAAAAAATCCAAGAGAAATTGCT

ATATCCTCTATTCAAAGAGGAGAAATGCGCCTGGATGTAATGCTTTTTCAAAAGGATCTA

ACCCTTACAGGATTGATAAAGGGGGGACTTTTGGTTATCGAACCAATTCGTCTATATCTA

AAATGGGATTTCCTATTTTTTATGTATCAAATTGTAGGTATTTCATTGGTTCATAAGGAT

AAACAGCAAATTAAAACAAGATGTCGAATAAAAGAATATGTTGATAAGATTGATTTCGAT

GGATCCATTTTACCATACGGAAAAACTCTTGTGAATGGGCACGAAAAGCATTATGATTTG

CTTGTTCCTGAACATATTCTATCTCCTAGGCGTCGTAGAGAATTACGAATTCTAATGAGT

TTTAATTCAGGAAAGGGGAATGTTATGGGTAGGGGTCCATTCATTTTGAATGGGAAAAGC

GTAAGGGATTGTGAACAATTTTTTTATGAAGACAAATATCTTGATACAGATACAAATAAA

TTCATTCAATTCAAATTATTTCTTTGGCCCAATTATCGATTAGAAGAATTGGCTTGTATG

AATCGCTATTGGTTTAATAGTAATAATGGAAGTCGTTTCAGTATGTTAAGGATACGTATG

TATCCACGACACAGAATTTTGTAATTGATGGTCAATTTTCTATATATTCATCACTATATC

CAGTATAGAAAGGCATGAAGATGTATACACACATATTGTGTTAGATGTCATTCTAAAACA

AAAACTGCTTCAATGACGTGCTAATTAAATATAGAAAAGAATTGGCGAAATCCTCTTAAT

TAAGTCAATTTTCTGTTTCACAGGTTCTAAAAAAAGGCAATCCCCCTATTCGAATTTTGT

TGGGATTGAAATTCCATTTGTTAATTTAATTTTTATTTTCTAGAAAACAAAGTAAAATCT

ATCTATTTGTGTACCCCAAACCAAAAAACGACTATTATTCTTGATCGGTAAAATCCATAT

ATATTTGTGGAAAAAAGAAATTCTTTTCTTTTTTTATTTTATGGTAAAAAATTCATTCAT

CTCAGCTATTCCGCAAGAAGAAAAAGAAAAAAACAAAGGATCTACCGAATTTCAAATATT

GAATTTCACTAAAAGGATACGTAGACTTACTTCACATTTGGAATTGCACAAAAAAGATTA

TTTATCTCAGAGGGGGCTTCGGAAGATTTTAGGAAAACGTCAACGGCTACTGACCTATTT

GTCAAATAAAAATAGAGTACGTTATAAAGAATTAATTGGTCAACTGGATATTCGGGAACC

AAAAACTCGTTAATTTGAATTTGAAGATTCGCTTGCATTTTTTGGTTTCTAAAATGAGAT

CTTTAATTTTGATGAACTTTGTTTCGTTTTCAGAAATTTGTAGAATAATGAATCGGGAAA

AAACATATGACTGTACCGGCTACAAGAAAAGATCTGATGATAGTCAATATGGGTCCGCAC

CACCCATCAATGCACGGTGTTCTTCGGCTCATCGTCACTCTAGATGGTGAAGATGTTATT

GACTGTGAACCCGTATTGGGTTATTTACACAGAGGGATGGAAAAAATTGCGGAAAATCGA

ACAATTATACAATATTTACCTTATGTAACCCGTTGGGATTATTTAGCTACTATGTTCACA

GAGGCAATAACGGTAAATGCACCAGAGCAGTTGGGAAATATTCAAGTACCTAAAAGAGCT

AGCTATATCAGAGTCATTATGCTGGAGCTGAGTCGTATAGCTTCTCATTTATTATGGCTT

GGACCTTTTATGGCGGATATCGGTGCACAAACTCCTTTCTTCTACATTTTTAGAGAGAGG

GAATTACTGTATGATCTATTTGAAGCTGCCACGGGTATGCGAATGATGCATAATTACTTC

CGTATCGGGGGGGTAGCTACCGATCTGCCTTATGGTTGGATAGATAAATGTTTTGATTTC

TGTGATTATTTTTTAACAGGAGTGGTGGAATATCAAAAGCTTATCACGCAGAATCCCATT

TTTTTGGAACGAGTTGAAGGGATAGGCATTATTGGTGGAGAAGAAGCAATAAATTGGGGT

TTATCAGGGCCGATGTTACGAGCTTCCGGCATCCAATGGGATCTTCGTAAAGTTGATCAT

TATGAGTGTTATGATGAATTCAATTGGGAAGTCCAATGGCAAAAAGAGGGGGATTCATTA

TCTCGTTATTTAGTACGAATCGGTGAAATGACGGAGTCCATAAAAATTATTCAACAGGCT

CTAGAAGGAATCCCAGGGGGGCCCTATGAGAATTTGGAAGTACGGCGCTTTGATAAAACA

AAAGATTCCGAATGGAATGATTTTGAATATCGATTCATTAGTAAAAAGCCTTCCCCCTCT

TTTGAATTGTCTAAACAAGAACTTTATGTAAGAGTAGAAGCCCCAAAGGGAGAATTGGGA

ATTTTTTTGATAGGGGATAATAGTGTTTTTCCCTGGAGATGGAAAATCCGTCCGCCCGGT

TTCATTAATTTGCAAATTCTTCCTCAGCTGGTTAAAAGAATGAAATTGGCCGATATCATG

ACGATACTAGGTAGTATAGATATCATTATGGGGGAAGTTGACCGTTGAAATGATAATTGA

TACGACAAAAGTACAAGCTATCAATTCTTTTTCCAGATCGGAATCTTTAAAGGAGGTCAT

AGGGCTCATATGGATGCTTGTCCCTATTTTGATTCTTGTATTGGCAATAACAATAGGAGT

ACTCGTGATCGTGTGGCTAGAAAGGCAAATCTCCGCGGGGATACAACAACGTATTGGTCC

TGAATATGCCGGTCCCCTGGGAATTCTTCAAGCTATAGCGGACGGAACTAAACTACTTTT

CAAAGAGGATATCCTCCCATCTAGGGGGGATATTCGTTTATTCAGCATTGGGCCATCTAT

AGCTGTCATATCCATAATACTAAGTTATTCAGTAATTCCTTTTAGTTATCACCTTGTTCT

AGCCGATCTTGGTATAGGTGTTTTTTTATGGATTGCCGTTTCCAGTATAGCTCCTATTGG

GCTTCTTATGTCAGGATATGGATCAAATAATAAGTATTCCTTTTCAGGTGGTCTACGAGC

TGCTGCTCAATCAATTAGTTATGAAATACCATTAACTCTATGCGTATTATCAATATCTCT

ACGTGTGATTCGTTGGAACATACATCAACCTTTATTTTCTTTCTTTATTTCTAGGAAATT

AATGGAATGTATTGAATAAATATCTTTATTTTTTTATTCAGCATTAGGGTCGATGAATTA

AACCCGATAGTTATATGAGTGAAACAAAACGGCTTATAAATTAGCAGTAATAGGATTAAT

TCTCATTCCCTATGTACAGGAGTAAAATTAAAGTACACATAAGCAGTATAAATGGGTTAC

CCCAAGGTTGGTTGATTAATCATCATGCCTTGAAGCGGGTACACAAAAAGAAAAACTGTA

TCAAGTTTTTACAATTTTTCTGTATTACTATACAGGGACAGGTAATCGGTTAAAAATGGG

TGGACAGTTAGGAACACCAAGATACGCAAAGGATTAGTAATGGAGATAATATAAGGTATC

CAAAGAAGTATTTTTAGATAAAAGGAATCATAATGAGGACTTTAAGTTGGTAGAAATGAC

CAAGCAGTACTTCCCCCTGATTCCGATCCAGAGTATGTTCCTATCCACTGATGAAAGAAA

TGACTATCAGGAACGAAGTAATACCTTATTTCATTTTCTTTTAGAGGAAAAACAACCCTT

CTGAGAAAGAAGAAAAGGAACGAAATAAATGGAAGAAATGGAATACAATAAAAAAAAAAA

TCTTTTTTTTTTATTTTCACTTTTTCTTTTCCTCGTATAATTCATACAGATATGATTATT

ATCATGATTCATGGAACTGGTGCAGTGTCTAATGATTTTTCGTAATATAAAGAAATAAGT

CAAAATATTTATTGATGCAAGGCATATACTATTGAAAGATTCAGTTATTACTGAAACAAA

GATAAATGGATAATTAAAAAAAATATTAAATATTATGAAATAAGATATGAGATCAATTCA

GAAGCATTATTATTAATATAGCAAACAGAATTTCATTGGTCTAATTTAATTAGGAACTTT

ACGATACATTTTTATTCTATCTAATCGACAAAAGCATGCGCAGGTAACGCCGAATTAGAT

TTATTTGCGACCATTGAGGAGCCGTATGAGGCGCGGGTCTCATGTACGGTTCTGTAATAG

CGATGGGAGAGCAGTAATGTTATCATCGACTATGATTATCTAACAGTTCAAGTACAATTG

ATATTGTTGAAGTACAATCCAAATATGGAATTTTGGGATGGAATCTTTGGCGTCAACCCG

TAGGGTTTATAGTTTTTCTAATTTCATCCCTAGCAGAATGTGAAAGATTGCCCTTTGATT

TACCAGAGGCAGAAGAAGAATTAGTTGCAGGTTATCAAACCGAATACTCAGGTATCAAAT

TTGGTTTATTTTATGTTGCTTCTTACCTAAATCTACTAGTTTCTTCATTATTTGTAACAG

TTCTGTACTTTGGGGGGTGGAATCTTTCTATTCCGTACACATCCTTTTTTGGACTTTTTG

GAATAAATCAAATCAGCGGAGTTTTTGGAACAACAATGGGTATGTTTATTACACTAGCCA

AAACTTATTTATTCCTCTTCATTTCGGTTGCAACAAGATGGACTTTGCCTAGAATGAGAA

TGGATCAACTATTAAATCTCGGATGGAAATTTCTTTTGCCTATTTCTCTAGGTAATTTTT

TATTGACAACTTCTTTCCAACTCCTTTCGCTATAACAAAATAGTAAGTTGTTCAGAGAGT

TAGAAACCCTCTCAAGAGAAAGAAATGGAAAAATATCATGAATATCGGCGAAGATGTTCC

CTATGATAACTGGGTTCATGAATTATGGTCAACAAACAGTACGAGCCGCAAGATACATTG

GTCAGAGTTTCACGATTACCTTATCCCACACGAATCGGTTACCTGTAACTATTCAATATC

CTTATCAAAAATCAATCACATCAGAGCGTTTCCGCGGCCGAATCCACTTTGAATTTGATA

AATGTATTGCTTGTGAAGTATGTGTTCGTGTATGTCCCATAGATCTACCTGTTGTAGATT

GGAAATTAGAAACAGATATTCGAAAGAAAAGGTTGCTTAATTATAGTATTGATTTTGGAA

TATGTATATTTTGTGGTAATTGCGTCGAGTATTGTCCCACAAACTGTTTATCAATGACTG

AAGAGTATGAACTTTCTACTTATGATCGTCACGAATTGAACTATAATCAAATTGCTTTGG

GTCGCTTACCAATATCAGTAATTGGAGACTATACAATTCAAATAAACAATTATGAATTGG

ACTCATATAAAAAAACGACAGGCAAACCTCTAGATTCAGAAAAGATTACCAATTAATTAC

TAAGACTCTGTTTGTTTTGATGGAAAAAAGGGGGGGGGTTCTTTTTGGATGGTTAAGCAG

TAACCACAAAAATCTGTATTTTTCATTTTTATTTATTTATTGATTTTATGATAATTGTGA

CTTGAATCTAGAATAGGTTCATATATCTGCGACAATTTCGAAATTTACAAACCATCCCGA

ACTACCCTTTCAGACAAGAAAGCGGGATTGGTGCATGAATGTGTCTGCATGAATTCACAT

CGCATTAAGATTCCATTAAGAACGTATTACATAGAATAATAAAAAAGGGCAATCTCCTTT

TCCTGAATCAATCATTAGGATCATGAAATATTTCTACTTTTTATTCTTTATTCTATTTCA

CATAATGGGTTTACCTGGACCAATACACGATATTCTTTTAGTATTTTTTGGGTCGGGTAT

TGTATTAGGAAGTCTAGGAGTAATATTACTTACCAATACAATCTATTCTGCTTTTTCTTT

GGGTTTAGTTCTTGTTTGTATATCCTTATTTTATATTCTATCGAATTCCTATTTTGTAGC

CGCCGCGCAGCTCCTTATTTACGTGGGAGCCATAAATGTTTTAATAATATTTGCTGTGAT

GTTTATGAAAGGTTCAGAATATTCCAAAGATTTTTCTATTTGGACTGTTGGGGATAGGGT

TACTTCATTAGTTTGTACAAGTATCTTTTTTTCATTAATTACTACTATCTTGGATACGTC

ATGGTACGGTATTATTTGGACTACAAGATCAAACCAGATTATGGAGCAAGACCTTTTAAG

TAACGTTCAACAAATTGGAATTCATCTAGTAACCGATTTTATTCTTCCATTTGAACTTAT

TTCAATAGTTCTTTTAGTTGCTTTGATAGGTGCAATTGCTATGGCTCGTCAGTAATGAAC

ATTACATTTTCTTTTTTTCGAAGAAAAAAGACAAATATTTGTCTTTTTTTATAGCGGAAA

ATTTCTCTCAGCCCTTTTTCACACCGTTTCTATATTTTTTTCACATTGTTTCCATATAGA

AAGTGGAAATTTTCGTTCTATCCATTACCAATATTTTTCTGTCCCATACCTTTTATACGC

GAGTTGAATCAACCAAATCGGTGAAATTGTTATTCATATTGAAATGAGTCGAGATTGATG

AGGAGTTGGTTAATGATGCTCGAGCATGTACTTTTTTTGAGTGCCTATTTATTTTCTATC

GGTATTTTTGGATTGATTACAAGTCGAAACATGGTTAGAGCACTTATGTGCCTTGAGCTT

ATTCTGAATGCGGTTAATCTAAATCTTGTAACATTTTCTCATTTATTTGATAGTCGTCAA

TTAAAGGGCGACATTTTTTCTATTTTTGTTATAACTATTGCAGCTGCTGAAGCAGCTATT

GGACTGGCCATTGTTTCATCAATCCATCGTAACAGAAAATCAACTCGTATCAATCAATCC

AATTTGTTAAATAAATAGTCATAATAATCGAAATAAAGAGAAATATTAATCTATCCTATC

TATAGATAAAAAATTTTATAATTCAAGAACTTCAGTTAGTATGTACATGTATCAGTCATA

TGATCATGTTTCCTAAAACGTAGGAAATCAAAGTATCTTGGACCTTTCTCATGAGCAGAT

TTAGAAGTCGATTGAATATGAAAAAAAAGATTCTGATACTTTACTGATTCGTTTGGTATC

TACAATAAATGATTTTTTTATTAATGATATACCAGATCGAAAATTGAAAACTCTCAAAAA

CTCAATAGACCCAATGTCGCACTCAGTAAAAATTTATGATACATGTATAGGATGTACTCA

ATGTGTACGAGCCTGCCCCACGGATGTGTTGGAAATGATACCTTGGGACGGATGTAAAGC

TAAGCAAATAGCTTCGGCTCCAAGAACAGAGGACTGTGTAGGTTGTAAAAGATGCGAATC

CGCCTGTCCAACGGATTTCTTGAGCGTTCGAGTTTATTTATGGCATGAAACAACTCGCAG

CATGGGTCTAGCTTATTGATACGTTTTAAAAAAATTCGACTTGAATCCATTTGATTCCTC

TTTACCGAGAAAAATCTGCACTCGACAAAAAAAATCGAGTGCGGATTTTTTTGGACAAAA

CCTATCTTGTCTTTACTACGAGTAATTTTCCTTGGTTAACAATAATTGTTGTTTTTCCGA

TATTCGCGGGTTTATTCATTTTCTTTCTGCCACATAGAGGGAATAAAGTGGTACGGTGGT

ATACAATTTCTATATGTTTATTAGAACTCCTTCTAACAACCTATGTTTTTTGTTATCATT

TCAAATTGGACGATCCGTTAATTCAATTGGAGGAGAATTATAAATGGATAACCATTTTTG

ATTTTCACTGGAAACTGGGAGTTGATGGGCTTTCCATAGGACCCATTTTACTGACAGGAT

TCATCACTACTTTAGCTACCTTAGCGGCCTGGCCAGTTACTCGATATTCGCGATTGTTCT

ATTTCCTGATGTTGGCAATGTACAGCGGTCAAATAGGCTCATTTTCTTCTCGAGACCTTT

TACTTTTTTTCCTAATGTGGGAGTTAGAATTAATTCCTGTTTACCTTCTTTTATCTATGT

GGGGGGGGGGGAAACGCCTTTACTCAGCTACAAAGTTTATTTTGTACACAGCAGGAGGTT

CCATTTTTCTCTTAATAGGAGTTCTGGGTATGGGCTTATATGGTTCCAATGAACCAACAT

TAAATTTGGAAACATTAGCTAATCAATCATATCCCCTAGCATTGGAAATAATATTCTATA

TTGGCTTTCTTATTGCTTATGCTGTCAAATTACCAATTATACCTCTGCATACATGGTTAC

CGGATACCCATGGAGAAGCACATTACAGTACATGTATGCTTCTAGCTGGAATCTTATTAA

AAATGGGAGCATATGGGCTGATTCGGATCAATATGGAATTATTGCCTCATGCCCATTCTA

TATTTTCTCCTTGGTTGATTCTAGTAGGGGCTATTCAAATAATCTATGCAGCTTCAACCT

CGCTTGGTCAACGCAATTTAAAAAGGAGAATAGCTTATTCTTCTATATCTCACATGGGTT

TCACAATTATTGGAATTGGTTCTATAACTGATACAGGACTTAATGGAGCCCTTTTACAAA

TAATTTCTCATGGATTTATTGGTGCTGCACTTTTTTTCTTGGCAGGAACAAGTTACGATA

GAATACGTCTTGTTTATCTCGACGAAATGGGGGGAATAGCTATCCCAATGCCTAAAATAT

TTACCATGTTCAGTAGTTTTTCAATGGCTTCTCTTGCATTGCCAGGAATGAGTGGTTTTG

TTGCGGAGTTGGTAATATTTTTTGGAATAATTACCAGCCCACAATATCTTTTAATCCCCA

AAATTGTAATTACTTTTGTAGTGGCTATTGGAATGATATTAACTCCTATTTATTCATTAT

CTATGTTACGGCAGATGTTCTATGGATACAAGCTATTCAATTGCAAAAAGTCTCATTTTT

TCGATTCTGGACCACGAGAACTTTTTATTTCAATCTGTATCCTTTTACCTGTAATAGGTA

TTGGTATTTATCCAGATTTTGTTCTCTCGTTATCAGTTGACAAGGTAGAAACTATTTTAT

CTAATTATTTTTAATTAATTAAAATTAATTAAATAAATTAAAAAAAATAAAATATAATAT

AAATAAATAAAAATATAAAAATAAATGAAAAAAAAAAAGTTTTCATGAATAATATGTAAA

ATGAAGTAAAAAAAAAAAACTCCTGTGATATTTAAAAGGAATTGCCGTAAATAAAAAAAT

GAAAAGAAAAAAACATCATGAATTAAAATAAAATCAAATACATCGGAAGTTTTTGAGAAC

CATTAAATTATAATTTAATGGTTCTCAAAAACTCGCAAAACTTTCGTTATATATGACATG

GAACAGTGTTCTTATGTATTTATCAAGTCATTTTTTTCTTTAATTAGAGGTGAATGCACC

ATAACTATGCAGTCCTATTCCTAATAGATTTACTCCGAAATAACATATCCAAATAATAAG

AAATCCAATCGAAGCTACAATTGCTGAATGCATATCCTGCAAACGTTGATTTGTTCTCGT

ATGTAAATAAATAGCGAATATGGTCCAAGTAATAAATGCCCAGGTTTCCTTGGGGTCCCA

ATTCCAATAAGATCCCCACGCCTCGTTCGCCCATACTGCTCCTGATAGAATACCTATGGT

TAACAAAGTAAATCCTAGATTAATAATCCGATAACTCCAATGATCCAACTGTTGAGTCAA

TTGATATTTATGATAATTTTTAAATGAAAGAAAAGAAGTGTTTTGTAAAAAACTTTTTTT

CTTTTTTAAAGGACGTACTTCGCTAAGTAAAAATGACTCAATTAAAAAAGAAAATGGATT

CATTTTCTCCAAGATTTCTATGTTTTTTCGAAATGTAATAACTAAAAGAGATACTGATAA

TAAGGATCCGCACAAAAGGGCTCCATAACTTAATAACATCATACTTACGTGCATCATTAG

CCATTGAGATTGTAGAGCGGGTACTAGTATTGCGGGTCGATGCATTTCATTTAAAATACC

TGAAGTTGCAAAGGCTTGGCAAAAAATAGCACTTGGGGCCGTAATTGTGCTTAAATCATT

TTGATGGTTCCATATTTTGGAAAACATATGAATAGTGGAGAAACCCCATGAAAGGAACAT

TAATGATTCATATAAATTACTTAACGGTAAATGTCTTGAATAAACCCAACGAGTAATTAA

TAAACCTGTTATGCAAAAAGAGGTAGTTATTATGCCCTTTTCTAACGAGTTACATAGTCC

TATGATTCCAGGAACTAATAATTTTATTAAATGAACCGAAATCACAATTGAAATAATCGA

AAAAGAGATGTGAGTTAATACATGTTCTAAAGCTATAGATATCATATAATAATGATTATA

TTCGTTAATGTAATTCAATAAATAAAAAAGACTTTCTTAGAATCAAATTAGGATTAGGAT

AGAAGAAAAAAATGCAAAATCGAATTTTCTATAGGATCTAATCTATTGTGCTCTCTTTCT

TTTTTTTTTTATTAGGGGGGTGCCGCCACTCGGACTCGAACCGAGATGCTCTAGCACTGC

TTCCTAAGAGCAGCGTGTCTACCAATTTCACCATAGCGGCTTGGTCAAAATTATAATAGC

ACATATTCGATTTAATTGTCTAGATTGATGGAATCAATGCAATTTATTTTTGTAAACATT

TGAATTGATGAATCCAACAAATTTTCAAATATAAATTTGAACGTTGAATAATACTTACCT

TAGCTTTTGTATGGTCTTATGTTAAAAACGAAAGAAAAAGGAGTGGAGTATTTCACATAT

CATAAGTTAACCCGTTCCGGTTAGACTGAGATTATTACGACACCCTTCTCCTTATTGAAT

CGAATTAAGAATTTTTTTTTTCTGTTTCTGTTTTTTATGCCTTAATTCTTATTTTATTTA

TCTCTTTCATAAACAAATACAAAATGGAAAAAGAAATACAGATTCTTTTTTAATGAAAAA

AAAGAGTTTCAACCTGATATGATTTCAATCCTAGCGTACTTATACAACCTTTTAGAACCT

TTAGATTAGAGTATTTTAAATATGAAATTTATTTCTAAGAAAATAGACATATATCCCCCT

ATATACATATATAGATTATAGGTAGATAAATAGGAATAGAATCCATATTGATCTATTTAT

AGATCAATATGGAGATTCCATCTAGATCCATAAAAAACAGAAAAAAATAAAACTTTTTTA

AAAAGAGATAATACTTTTTCTAAAACCCCTTTAGACAGATAAATTTTCATTCTACATATC

AAAATACCCCGGAAAATCCCGTTTATATGGATTGAAAGTTGGAATTAATGTAAATTATTC

ATTTTATTTATAATAAGTCGTCGACTTTCTAATTCATAGAGTAAGATTTATTGCTTATTT

TTTTGTAACACAAAAAAACTCTTTGAACGTCCAGTAGAAATGGATTTCGCTAAGGAAAAA

GCCCTTTTTGCCTCCCAATATCCCCGTTTTTTCCAAATATTTCTACGAATACGTTTTTTT

GATATAGAAGTACGTTTCTTTGGAACCGCCATTTCAAAGATGTTAGTTATTTTTCTTTTA

TAGTTATATGTGAAAGACATGTATTATTCAAAAAGGATTCTTTTTCTATATCTATATTTA

TTCCATACTGAATACTTCGTTCGTTTATATCTTAAAGATAGGCGCATATTCGAATAGAAT

ATTCTTAAGAAAAAAAGAAATAGAAGAAAAGGGTATGTTTATTTAATAGAATGCAATCCT

TTTCACATCTCTATTATATGGAATAATGCGAAAAGAAAGAAAAATTCGTATTCATTGGTA

CCTTGATATCTTTATATGGGTCCATGTCTATGGGATCCATTTAATTGAAGTGCAACCAAT

TCCCTTTTCTTATAAATAGAAAGAAAAAGATTCTAATTAATGTCTTAGTCCTATACTGTT

GCCTTATATTTTTTAAGATAGAAAGGTTTAGATTAAGATAAGAACCCTTACCTATACTAA

ATGAAGAAAACGATAATAAAACACTTTTCTATTAATTGATCATTCTCGAGGATAAAGTAC

TTCTAGTCTAATTAGAATGAAATAAGACTAGAAATAAATTTCTTAAAGAATACATTTTTT

TTTGAAGCGGAAAGTTATTTTAGTAAAAATACATCTTAGTTCTATCTAAAATTATGAATA

CACTAGTCATAAGATTTCAAAAAAAAAAAGAAACCACAGGTTTCTTTGATTAGGATAAAA

AAAGCCAATCAATTAGTTTTACAATAAATTAGACAATGACTATGAATAAAATATGATTAA

ATTGCGCCGTCTTGGGTAGAGTCGAGTTTTTGTTGGATATCATGTCCCCGAATCATTCCC

TTTTATGTTATTTTTTTCTTACTATAAATATAAAGAAAAGATATAAATCGGCATCTAACT

AATAAACTAAGAGAATAGACAAGCGATTGAAAATTTCATACTCTCTCTTATTATATTATT

TTATTAATTAATATTATATTAATATTTTAATTATGAAAAAATAATAATTAACCCAATTTT

TCAAATTAAAAAAATAACCAAGCAAGGCGATTCCTCTATTTGTTTCTTTTTTTTTTATCT

CACTATAGATGGATTCTATAGATTGATTCTTTTTATTTAATTCTTTTTATTTAATTATTG

TTACTATTGTAAAGATAAGAGCTGGTGAGTCAGAAGCAATTAATAAGAAAAAAAATTGGA

TTTTCCTATGGAACGTACATACCCATATGCGTGGATCATACCTTTTATTCCACTCCCGGT

TACTGTGTTAGTAGGATTAGGACTTCTATTTGTTCCGAATGCAACAAAAAATCTTCGCCG

TATGTGGGCTTTTATTAGTGTTTTATTATTAAGCATAGCTTTACTTTTTTCGATCGATAT

ATCCATTCAACAAATAAGGGGCAGCTCCGTTTGTCAATATCTATGGTCTTGGACCATAAA

TAATGATTTTTCTTTGGAGTTTGGCTACTTGATTGATTCGCTTACTTCTATTATGTTAAT

ACTAATCACTACTGTTGGAATCATGGTTCTTATTTATAGTGATAATTACATGTCTCATGA

CCAAGGATATTTGAGATTTTTTGCGTATCTGAACTTTTTCAATGCTTCCATGCTTGGATT

AGTTACTAGTTCCAATTTAATACAAATTTATATTTTTTGGGAACTTGTAGGAATGTGCTC

TTATTTACTAATAGGTTTTTGGTTCACACGACCCATTGCAGCAAATGCTTGTCAAAAAGC

CTTTGTAACTAATCGTGTAGGGGATTTTGGTTTATTATTAGGAATCTTAGGACTTTATTG

GATAACGGGCAGTTTTGAATTTCGGGATTTATTCAAAATTTTCAATATCTTGGTCCATAA

TAATACAATAAATCTTTTTTTTGCAGCTCTGTGTGCCTCTCTATTATTTCTTGGTGCAAT

TGCGAAATCCGCACAATTCCCTCTTCATGTATGGTTGCCTGATGCAATGGAAGGTCCTAC

GCCTATCTCCGCTCTTATACATGCTGCGACTATGGTCGCAGCGGGAATTTTTCTTGTTGC

TCGACTTTTTCCTCTTTTTATCGACTTACCATACATAATGTATTTTATTTCTTTGATAGG

CATAATCACAGTATTATTAGGAGCTACTTTAGCTCTTGCGCAAAGAGACATTAAGAGAAG

TTTGGCCTATTCGACAATGTCACAATTGGGTTATATTATGCTAGCTATGGGGATAGGTTC

TTATCGAGCTGCTTTATTTCATTTGATTACTCATGCCTATTCGAAAGCATTATTGTTTTT

GGGATCCGGATCCATTATTCATTCTATGGAACCCATAGTTGGATATTCGCCAGACAAAAG

TCAGAACATGGTTCTTATGGGTGGTTTAACAAAATATGTGCCAATTACAAAAACCACTTT

TTTATTAGGTACACTCTCTCTTTGTGGTATTCCACCTCTTGCTTGTTTTTGGTCGAAAGA

TGAAATTCTTAATGATAGCTGGTTATATTCACCAATTTTTGCGATAATAGCTTATTCCAC

AGCTGGTTTAACCGCATTTTATATGTTTCGAGTATATTTACTTACTTTTGATGGACATTT

GCGCATTCATTTTACAAATTTGAATGGAGCTAAAAGCCGTTCACTCTATTCAATATCTAT

ATGGGGAAAAGAAGAATCAAACTTGAATAATCCAAATTGTTTTTTATCAGAAATCAACAA

AAATGATGAAAAGGTTTTCTTTTTTTCAAAAAAAACATATAAAATGGGTAATTATGCGAA

AAACATAATGTTCTCTTTTAGTATTTATTTTGGTAATGAAATTCTTTTTCCATATCCACA

TGAATCGGACAGTACCATGTTATTATCAATATATGTATTGGGTCTGTTTACTTTGTTCGT

TGGATTCATAGGAATTCCTTTGGATCAAGGAATCATTTATTTTGATATACTATCAAAATG

GTTAACTCCATCAATAAACCTTTTGCATAAAAATTCACATTATTCTGTGGATTGGTATGA

GTTTGTGATAAATGCCATTTTTTCAGTTAGTATATCATTGTTTGGAATATTCATAGCGTC

TTTTTTATATGGGTCTGTTTTTTCATCTTTTCGAAGTTTGGACTTAATTAATTCATTTGA

AACAAAAGGCCAAAAAAGCATTTTTTTAGACCGAATTCTAAATGTAATATACAATTGGTC

ATATAATCGTGGTTATATAGACAACTTTTTCACACTCTATTTAACTAGAAATATAAGGAA

GTTGTCCGAACTAACCCATTCTTTTGACAAACGGCTGATTGATGGAATTACCAACGGGGT

TGGTGTTGCGAGTTTTTTTGTGGGAGAGGGAATAAAATACGTAGGAGGGGGGCGAATTTC

TTCTTACTTGTTTGTGTATTTATCTTATGTATCAATCTTTTTAGTAGGAATTTACTACTT

TTTTGTTTTGTGAATCAACCTTTGTAATTGTTCCACGATATGGTCCGTTGAAAAACGGAT

CATACCATTTAGGCAAGCATTTTTGTTCATTCTCATCATGGCACAATCGGATCCTTTTTT

CAAGTACATCTATTGAAACAGACCCCTTCTCTATAGCTTCTATTCGATTTATTAACTCAT

TACTCAAGTTTTTTTTTTTTTTTTTAAAAGTATAAACCCAATTATTATACAGGTCCTCTG

GGGATCCTTTTTCTGTCGTGTATAAAGACATCTTTTTTTGTATTATTTCCTCAATAGTCG

ACAAACTGGGTGGATATGTAAAAGATATGATTTTTTTTCCATCACTTGGACATGTGTGAA

AGAAATATTGTGACATTTTATTGCTTACAGCATTTTCAAATCGATCATTTTTTATATATC

GTAATGGACGATTCCATCGCTTATAGTCGAAAAGAAGAGTTACAAGAGGTTTTTCAAACC

AAAAGAGGTCTTTATCTTCCTCATCTTTAAGTATTTCCTTTCCATTCACTCGGATCTCTT

CCGTTTCATCTAGTTTGTCCGGATCCTCCTGTTCTTCCGAACAAAGAGAAGGGTCTTCTT

CGGTGGATCCCTCTTGTTCCTGTTTAGTCCCCTTCGTTTCGGAAGTCGTTTCTATTTCTA

CATCTGTTTCTTCCTCACTTTCCCCTCTTTCTTCCATTTCTGAGGTTTCTTTCAGTTTCT

TAGTAACAATAGGCGATGGCATTCTGCCTAAATAGTAGACAGAGGTGATAAATAAGAGAA

TACTAAAGATTCGAGCCATAGAATTTCTCAATTCTGACACAAGGTACTTATTAGATCGAA

TAAGTACATTAGATTGAATAGAATGATTTTGCCGTATCCAGGATAATACCAATCCAACCC

ATTTCATGAATAAAATGTGACCAATTAACCAACCAACAAAACTACTTGTTACAAATAAGA

TCTTATTGTTGCATCGAAACAGATAAATGTTGACTAATCTGGCTAACGTTGAGCTTGGTA

AAATGAAATGGTTGAATAATTGAAAAATGAGATTATTCAGGAATACACATTGAATGCTGA

GATTACGCATGGAATTTCTGGTAGTAGATCCATAATCCAAAAAGTGTTTGTGATTGTTCC

AGAAGAAATGAAACAAAAGGTACGGTAGAACTAGGACAGTTATTGTATGAGGTCTACCCA

ATGCTAGGTGCAGAGGCGCATAATAGATCGATATAAACATCATGAGCTGCCCCGTAATAA

AACCAGTTGTTGATGATATCTCCTTCTCGGTTTCTTCTTCCATAACCTGAGCTCGGAGAA

GGAAGAGATAAGAGGGCCCTATGGAGAATGTGGTCAGAAATCCATAATAGAATCCGACCA

CAACGACCGAATTGATTATCTTCATGCATAAGGATAATAGATTACCTAGTAGAAAAGATT

TCAAAATCATCACAAACCTCCCTTTTTTCTTTTCTATTGCAATTTCTCGATTATTATATG

ATGATTTCTTAACTTTCCATATATGGAAACAGATAGACTAGAAATGACATCTCTTATGTC

AATGATACCATCTTCTTCGATTGAATGACATCTCTGATGTCAAGGACGCCAAAGGGATAT

TAAATGAATGGAATTGGGATATGGATGGAATATAATGAAATAGAGCCACTTTTAGGTTCC

CTATGAAATGAGGCATGGAAGGGGGCCACTACGAAGAAGTTACGGGAGTTACGAAGGGAG

CTTCGGGCTCATATTGGTCATGGGTTGAGAACGGGAGTTGAACTCTATGAGATCTAATCT

CCTGTTATTCCTCAGTAGCTCAGTGGTAGAGCGGTCGGCTGTTAACTGACTGGTCGTAGG

TTCGAATCCTACTTGGGGAGATTTGATGAATTCTGAATTCTTTCATTTTGAATTCAGAAT

GCAAAGGCTCGCTTTGACCGTTAGGAGTAGGTAACCCGTTGCTTGTCTTTGTTTCTATTG

CATTCTATCTCATCGTATCACATTCTGTTCTGCGAGATTTGAGAATCACCGTCAATACCC

CGATCCGGGATAATCCTTGGTTCCATAGCCCCGACGGGGCTATTTACAACTAGCCAATTA

AGAATTCTCAGATGGATGTACTGGCAGTGCATCAAAGGTGGAGTCATCGATTCTCCCGAG

AGGCCACAATTCTCGCGAGCAAACATATTAATGACGAGGAATGCATTTTTGCTTTGCTAT

GCTACTAATACTAAATAATACTTGTATTTGCTCTGCTATTCTGCCCAAGCCTGGCTGAGG

AAGAGTTACGGGGAGTAAAACACAAATGATTCGACCCCTGCATAGCATACTATACGCATT

TTTCCTTCACGATAAATAATAAGGCCATTCCATTTCGACAAAAGACCCAAGTTCTATAGC

TTTGCATCCGCTATCCCGATCATGATTTTCCTACCCACCACACATAGGTAAAGGAAAGGT

TCTTTTTGGCCGATTGTGGGCGAGGAGGGATTCGAACCCCCGACACCGTGGTTCGTAGCC

ACGTGCTCTAATCCTCTGAGCTACAGGCCCCACCCCATCTCCACTGGATCTGTTCCCGGG

AGTACCCTCAAAAGGAACCTTTCCTCCCCTCAGCCATTTCATTTCGGGTTAAGAAGATAT

GAAAGCGCGTTTCTCTATATAAGAACAGTGCGTTCCGAGGTGTGAAGTGGGGGAGAAGGG

GTGTCATAATTGGGGTTTTGAATAAGACGACCTTTTCATTTTTCATTTTTTTTTTTTTAA

GAAAAAAAAAAAAAATGAATAATAGGTGTTAAGCTTTTTATCATCCTGGCGTCGAGCTAT

TTTTCCGCAGGACCTCCCCTACAGTATCGTCACCGCAGTAGAGTTTAACCACCAAGTTCG

GGATGGATTGGTGTGGTTCCTCTACGCCTAGGACACCAGAATATCGAACCATAAACGAAG

AAAGGCATGAGAGAAAAGCCTATTGGCTAGTAATTGTGAGGCCCCAATTCTTGACTGGAA

GGGACACCAAACAAAGGCCTCTGCCCTTCCATCCCTTGGATAGATAGAGAGGGAGGGCAG

AGCTTTTGGTTTTTTTCATGTTGTCAAACAGTTGAACAATGAAAATAGATGGCGAGTGCC

TGATCGAATTAATCGGGTCATGTAGGAACAAGGTTCAAATCTCTCGGTCTGTTAGGATGC

CTCAGCTGCATACATCACTGCACTTCCACTTGACACCTATCGTAATGATAAACGGCTCGT

CTCGCCGTGACCTTATCTTGGATTCTCAAGACTTCTGTCGCTCCATCCCCCCAGGGGCAG

AGAAGAACCCGTCGCTGTCTCGGCTGTGCTACCGGAGGCTCCAGGGAAGTCGGAATAGGA

GAGCACTCATCTTGGGGTGGGCTTGCTACTTAGATGCTTTCAGCAGTTATCCGCTCCGCA

CTTGGCTACCCAGCGTTTACCGTGGGCACGATAACTGGTACACCAGAGGTGCGTCCTTCC

CGGTCCTCTCGTACTAGGGAAAGATCCTCTCAATGCTCTAACGCCCACACCGGATATGGA

CCGAACTGTCTCACGACGTTCTGAACCCAGCTCACGTACCGCTTTAATGGGCGAACAGCC

CAACCCTTGGAACATACTACAGCCCCAGGTGGCGAAGAGCCGACATCGAGGTGCCAAACC

TTCCCGTCGATGTGAACTCTTGGGGAAGATCAGCCTGTTATCCCTAGAGTAACTTTTATC

CGTTGAGCGACGGCCCTTCCACTCGGCACCGTCGGATCACTAAGGCCGACTTTCGTCCCT

GCTCGACGGGTGGGTCTTGCAGTCAAGCTCCCTTCTGCCTTTGCACTCGAGGGCCAATCT

CCGTCCGGCCCGAGGAAACCTTTGCACGCCTCCGTTACCTTTTGGGAGGCCTACGCCCCA

TAGAAACTGTCTACCTGAGACTGTCCCTTGGCCCGTAGGTCCTGACACAAGGTTAGAATT

CTAGCTCTTCCAGAGTGGTATCTCACTGATGGCTCGGGCCCCCCCGCAAGGGGGCCTTCT

TCGCCTTCCACCTAAGCTGCGCAGGAAAGGCCCAAAGCCAATCCCAGGGAACAGTAAAGC

TTCATAGGGTCTTTCTGTCCAGGTGCGGGTAGTCCGCATCTTCACAGACATGTCTATTTC

ACCGAGCCTCTCTCCGAGACAGTGCCCAGATCGTTACGCCTTTCGTGCGGGTCGGAACTT

ACCCGACAAGGAATTTCGCTACCTTAGGACCGTTATAGTTACGGCCGCCGTTCACCGGGG

CTTCGGTCGCCGGCTCCCCTGTCATCAGGTCACCAACTTCCTTGACCTTCCGGCACTGGG

CAGGCGTCAGCCCCCATACATGGTCTTACGACTTTGCGGAGACCTGTGTTTTTGGTAAAC

AGTCGCCCGGGCCTGGTCACTGCGACCCCCTTTGTGAGGAGGCACCCCTTCTCCCGAAGT

TACGGGGCTATTTTGCCGAGTTCCTTAGAGAGAGTTGTCTCGCGCCCCTAGGTATTCTCT

ACCTACCCACCTGTGTCGGTTTCGGGTACAGGTACCCTTTTGTTGAAGGTCGTTCGAGCT

TTTCCTGGGAGTATGGCATGGGTTACTTAAGCGCCGTAGCGCCTGGTACTCGGACATTGG

CTCGAGGCATTTTCTCTACCCCCTCTTACCCCGCCCTTAAAAAGCAAGGTCACCTTGCGT

CCTTGAACCGATAACCATCTTTCGGCTAACCTAGCCTCCTCCGTCCCTCGGGACCAACAA

GGGGTAGTACAGGAATATTCACCTGTTGTCCATCGACTACGCCTTTCGGCCTGATCTTAG

GCCCTGACTCACCCTCCGTGGACGAACCTTGCGGAGGAACCCTTAGGTTTTCGGGGCATT

GGATTCTCACCAATGTTTGCGTTACTCAAGCCGACATTCTCGCTTCCGCTTCGTCCACAC

CTGCTCACGCGGGCGCTTTCATCTAAGGCGGAACGCTCCCCTACCGATGCATTTTGACAT

CCCACAGCTTCGGCAGATCGCTTAGCCCCGTTCATCTTCGGCGCAAGAGCGCTCGATCAG

TGAGCTATTACGCACTCTTTCAAGGGTGGCTGCTTCTAGGCAAACCTCCTGGCTGTCTCT

GCACCCCTACCTCCTTTATCACTGAGCGGTCATTTAGGGGCCTTAGCTGGTGATCCGGGC

TGTTTCCCTCTCGACGATGAAGCTTATCCCCCATCGTCTCACTGGCCGACCTTTACCCCC

TTTTTGGGGGTCATATCTAGTATTCAGAGTTTGCCTCGATTTGGTACCGCTCTCGCGGCC

CGCACCGAAACAGTGCTTTACCCCTAGATGTCCAGTCAACTGCTGCGCCTCAACGCATTT

CGGGGAGAACCAGCTAGCTCTGGGTTCGAGTGGCATTTCACCCCTAACCACAACTCATCC

GCTGATTCTTCAACATCAGTCGGTTCGGACCTCCACTTAGTTTCACCCAAGCTTCATCCT

GGTCATGGATAGATCACCCAGGTTCGGGTCCATAAGCAGTGACAATTGCCCTATGAAGAC

TCGCTTTCGCTACGGCTCCGGTGGGTTCCCTTAACCAAGCCACTGCCTATGAGTCGCCGG

CTCATTCTTCAACAGGCACGCGGTCAGAGATTGCTCCTCCCACTGCTTGGGAGCTCACGG

TTTCATGTTCTATTTCACTCCCCGATGGGGGTTCTTTTCACCTTTCCCTCACGGTACTAC

TTCGCTATCGGTCACCCAGGAGTATTTAGCCTTGCAAGGTGGTCCTTGCTGATTCACACG

GGATTCCACGTGCCCCATGCTACTCGGGTCAGAGCGTAAGCTAGTGATGCTTTCGGCTAC

TGGACTCTCGCCATCTAGGGTGCAGTACTCCACCGCTTCGCCTAGCAGCACGACGCTTGT

ATTGCTCTCCCACAACCCCGTTTTCGCGGTTTAGGCTGCTCCCATTTCGCTCGCCGCTAC

TACGGGAATCGCTTTTGCTTTCTTTTCCTCTGGCTACTAAGATGTTTCAGTTCGCCAGGT

TGTCTCTTGCCTGCCCATGGATTCGGCAGCAGTTCGAAAGGTTGACCTATTCGGGAATCT

CCGGATCTATGCTTATTTTCAACTCCCCGAAGCATTTCGTCGCTTACTACGCCCTTCTTC

GTCTCTGGGTGCCTAGGTATCCACCGTAAGCCTTTCCTCGTTTGAACCTCGCCATTAACG

TTAAGGCTATGCCATCCTAAGGTGCTGCTAAATGGAAGGATCTTATCAACGTCCATGAAT

GAGAAATCATAGATCGAACCGCCGAATCGGAAAAATTGGGTGCTATCATATATATACCTT

TGTATCAGCTAAGGTCACGAGCTGGAGATAAGCGGACTCGAACCGCTGACATCCGCCACA

GGGTAAACCACCGCCTCTCGGGCCTCCCCGACTGATTCTACCATAGAGGCCAACGATAGA

CAATAACTCCCCCCCGAACACAGCTTACAACTTTCATCGTACTGTGCTCTCCAAAGAGCA

ACTCTTCTCAAAAGGTGCTGAGTTGGAATCCCATTCTAAGGATTCTTGTGGTTCCGGAGG

ATCCAGCTACAGGAGAACCAGGAACAGAGAGCTCTCCCCCCTTTTTCCGCTTCCGCCCGA

CTCTTTGGTTTGGTCTTAAGAATGCTGGTTTTAAGAATGAGTGATTGCCCTTCTCCGACC

CTTACTGCCCAACCGGAGAGCGGACAGCTAATGCGTTCCGCTTATTGAACAGGGTCTATG

GTCGGTCTGTGACCCCTGGATGCCGAAGGCGTCCTTGGGGTGATCTCGTAGTTCCTACGG

GGTGGAGACGATGGGGTCGGTCCATGGATTTTCCTTCCTTTTGCCACATTTCGCTCAAAG

GGTTGAAGGGAGATAGTGCATCAAGCTGTTAGCAAGGGCCAACTTGATCCTCTTCCCCAG

GGATCCCAGATGAGGGAACCCTAGGGGAGCCGCCGACTCCAACTATCGTCCATGTACGAT

CTATACTAGATCTGGCCAACTGCCCATCCTACCTCCTCTACGTTCTTGACAGCCCATCTT

TGTCTCAGTAGAGTCTTTCAGTGGCATGTTTCGGTCCTCTTCCCCATTACTTAGAAAAAG

TGAGCCACCGGTTCAGGTACAAGATACTATCATTACCGCCTGGACAATTAGACATCCAAC

CCGTAATCGCAACGACCCAATTGCAAGAGCGGAGCTCTACCAACTGAGCTATATCCCCCC

GAGCCGAGTGGAGCATGCATGAAGGAGTCAGATGCTTCTTCGATTCTTTTCCCCGGCGCT

GCTGGGCCATCCTGGACTTGAACCAGAGACCTCGCCCGTGAAGTAAATCATCGCACCTAC

GATCCAACCAATTGGGAGAGAATCAATAGATTCTTTTTCGGGAGCGATTCATCCTTCCCG

AACGCAGCATACAACTCTCCGTTGTACTGCGCTCTCCAAGTGTGCTTGTTCTCCCCTTCT

TCCTTACCATGATAAGTCTTTGTGAAATAACTCCGATGAGAAGAAAAAGAAGGCGTTAAG

AGACCCTCCTGGCCCAATCCTAGACACTCTAAGATCCTTTTTCAAACCTGCCCCCATTTT

GAGTCAAGAGATAGATAAATAGACACATCCCTTTGCACTGATCGGGGGGCGTTCGTAGTG

ACTGAGGGGGTCGAAGACCAAGAAGTCCGTTTATTTATACCAAGCATTCTTCTTATGGCT

AGATCCAATCTCCTGGTCCCTGCGGAAAGGAAAAAAAAAAGAATTTCACGTTCTTCCTTT

CGGGAAGGAAGGATTAGGGAAATCCTATTGATTGCTGCTTTCTCCAGACCTCCGGGAAAA

GCATGAAAAAAAAGGCTCGAATGGTACGATCCCTCCGTCACCCCAGAATGAAAGGGGCGA

TCTCGTAGTTCTTGGTCTGTGAAGATGCGTTGTTAGGTGCTCCATTTTCGCATTGAGGCC

GAACCTAAACCTGTGCTCGAGAGATAGCTGTACATACACTGATAAGGGATGTATGGATTC

TCGAGAAGAGAGGAGCCGTGGTGGTCCCCCCCGGACCGCCCGGATCCCACGAGTGAATAG

AAAGTTGGATCTACATTGGATCTCACCTGAACCGCCCCATCTATCCTCCTGAGGAGAAGT

TTGGTTTAAAACTCCGGTTCGAACAGGAGGAGTACGCCATGCTACTGTGCCTTGGATGAT

CTACATCTCCGGGTCAGGCGCTGATGAGCACATTGAACTATCCATGTGGCTGAGAGCCCT

CACAGCCCAGGCACAACGACGCAATTATCAGGGGCGCGCTCTACCACTGAGCTAATAGCC

CTGCGGGCCTCCCACGGGGGCCCGCTATGCCAAGAGAGAAAGCCCATCCCTCTCTTTCCT

TTTTTTTTTGCGCCCCCATGTCGCCACACGGGAGTGGCATGGGGACGTAAAAAGGGGGGA

TCCTATCAACTTGTTCCGACCTAGGATAATAAGCTCATGAGCTTGGTCTTACTTCACCGT

CGAGAAACGAAAAAAGACTTCCATCTCCAAGCTTAGCTCAGACGTAGCTCGCTTCTTTTC

TTTTTGGATGTGAAGCAGTGTCAAACCAAAATACCCAACAAGCATTAGCTCTCCCTGAAA

AGGAGGTGATCCAGCCGCACCTTCCAGTACGGCTACCTTGTTACGACTTCACTCCAGTCA

CTAGCCCCGCCTTCGGCATCCCCCTCCTTGCGGTTAAGGTAACGACTTCGGGCATGGCCA

GCTCCCATAGTGTGACGGGCGGTGTGTACAAGGCCCGGGAACGAATTCACCGCCGTATGG

CTGACCGGCGATTACTAGCGATTCCGGCTTCATGCAGGCGAGTTGCAGCCTGCAATCCGA

ACTGAGGACGGGTTTTTGGAGTTAGCTCACCCTCGCGGGATCGCGACCCTTTGTCCCGGC

CATTGTAGCACGTGTGTCGCCCAGGGCATAAGGGGCATGATGACTTGACGTCATCCTCAC

CTTCCTCCGGCTTATCACCGGCAGTCTGTTCAGGGTTCCAAACCCCATGGTGGCAACTAA

ACACGAGGGTTGCGCTCGTTGCGGGACTTAACCCAACACCTTACGGCACGAGCTGACGAC

AGCCATGCACCACCTGTGTACGCGTTCCCGAAGGCACCCCTCTCTTTCAAGAGGATTCGC

GGCATGTCAAGCCCTGGTAAGGTTCTTCGCTTTGCATCGAATTAAACCACATGCTCCACC

GCTTGTGCGGGCCCCCGTCAATTCCTTTGAGTTTCATTCTTGCGAACGTACTCCCCAGGC

GGGATACTTAACGCGTTAGCTACAGCACTGCACGGGTCGATACGCACAGCGCCTAGTATC

CATCGTTTACGGCTAGGACTACTGGGGTATCTAATCCCATACGCTCCCCTAGCTTTCGTC

TCTCAGTGTCAGTGTCGGCCCAGCAGAGTGCTTTCGCCGTTGGTGTTCTTTCCGATCTCT

ACGCATTTCACCGCTCCACCGGAAATTCCCTCTGCCCCTACCGTACTCCAGCTTGGCAGT

TTCCACCGCCTGTCCAGGGTTGAGCCCTGGGATTTGACGGCGGACTTGAAAAGCCACCTA

CAGACGCTTTACGCCCAATCATTCCGGATAACGCTTGCATCCTCTGTCTTACCGCGGCTG

CTGGCACAGAGTTAGCCGATGCTTATTCTTCAGATACCGTCATTGCTTCTTCTCCGAAAA

AAGAAGTTCACGACCCGTGGGCCTTCTACCTCCACGCGGCATTGCTCCGTCAGGCTTTCG

CCCATTGCGGAAAATTCCCCACTGCTGCCTCCCGTAGGAGTCTGGGCCGTGTCTCAGTCC

CAGTGTGGCTGATCATCCTCTCGGACCAGCTACTGATCATCGCCTTGGTAAGCTATTGCC

TCACCAACTAGCTAATCAGACGCGAGCCCCTCCTCGGGCGGATTCCTCCTTTTGCTCCTC

AGCCTACGGGGTATTAGCAGCCGTTTCCAGCTGTTGTTCCCCTCCCAAGGGCAGGTTCTT

ACGCGTTACTCACCCGTCCGCCACTGGAAACACCACTTCCCGTCCGACTTGCATGTGTTA

AGCATGCCGCCAGCGTTCATCCTGAGCCAGGATCGAACTCTCCATGAGATTCATAGTTGC

ATTACTTATAGCTTCCTTGTTCGTAGACAAAGTGGATTCGGAATTGTCTTTCATTCCAAG

GCATAACTTGTATCCATACACTTCATATTAGCCTGGAGTTCGCTCCCAGCAATATAGTCA

TCCCTGCCCTCTCACGTCAATCCCACGAGCCTCTTATCCATTCTCATTCGATCACGGCGG

GGAAGCAAGTCAAAATAGAAAAACTTACACTGGGTTTAGGGATAATCAGGCTCGAACTGA

TGACTTCCACCACGTCAAGGTGACACTCTACCGCTGAGTTATATCCCTTCCCCGCCCCCA

TCGAGAAATAGAACTGACTAATCCTAAGTCAAAGGGTCGAGAAACTCAACGCCACTATTC

CTGAACAACTTGGAGCCGGGCCTTCTTTTCGCACTATTACGGATACGAAAAGAATGGAAA

AATTGGATTCAATTGTCGACTGCTCCTATCGGAAATAAGATTTACTACGGATTCGAGCCA

TAGAGCCATAGCACATGGTTTCATAAAATCCGTACGATTGATTTTCCCGATCTAACTCGA

GCAGGTTTTACAGGAAGAAGATTTTCTTGAGCATGTTCTATTCGATACTGGTGGAAGAAG

AACCTGATTAGGTATTGTTAAAAAAGAGAGGAAGCAGAACCAAGTCAAGATGATACGGAT

CAACCCCTTCTTCTTGCGTTGCGCCAAAGATCTTACCAGTTCCTTCGGAACGGGAGCTAC

ATCTCTTTTCAATTTCCATTCAAGAGTTCTTATTTGTTTCCACGCCCTTTTGAGACCTCG

AGAAATGGACAAATTCCTTTTCTTAGGAACACATACAAGATTCGTCACTACAAAAAGGAT

AATGAGAACCCCACCATTAACTACTTCATTTATGAATTTCTATGAATTTCATAGTAATAT

AGTAATAGTAATATAGTAATAGAAATAGAAATACATGTCCGACCGAGACAGAATTTCTAA

CCTGCTATCCTCTTGCCTAGCAGGCAAAGATTAACCTCCGTGGAAAGACCGATTCATTCG

GATCGACATGAGGGTCCAACTCCATTGCATTGCCAGAATCCATGTTGTATATTTATTTGA

AACAGGTTGACCTCCTCGCTTCTCTCATGGTACAATCCTATTCCCGCGGAGCCCCCTTCC

TCCTCGGTCCACAGAGACAAAATGTAGGACTGGTGCCAACAGTTCATCACGGAAGAAAGG

ACTCACCGAGCCGGGATCACTAACTAATACTAATATAATAGAAAAGAACTGTCTTTTCTG

TATATTTTCCCCGGTTCTGTTGCTACCGCGGGCCTTACGCAATCGATCGGATCATATAGA

TATCCCTTAGCATAGGTCATCGAAAGGATCTCGGACGACCCACCAAAGCACGAAAGCCAG

GATCTTTCAGAAAATGGATTCCTATTCGAAGAGTGCATAACCGCATGGATAAGCTCACAC

CAACCCGTCAATTTGGGATCCAATTCGGGATTTTCCTTGGGAGGTATCGGGAAGGAATGT

AATAATATAGATTCATACAGAAGAAAATGTTCTCTATTGATTCAAACGCTGTACCTATCG

GATAGGGATAGAGGAAGAGGAGAAACCGAAGATTTAACATAGTACTTTTGATCGAAAAAT

GAATCTGATTGATTTCGTACCCCTCGCTCGATGAGAAAATGGGTGAGATTCTACAGGATA

AAACCTATGGGACTTAAGGAATGATAGAAGGGAATAAAAAGAAAAGAGAGGGAAAGAAAA

TCGAAATAAAGAATCAAAGAAAAAAAAAATAAAGATAAAAAGATAAAGTAAGTAAATAAA

TAGAAATGAAATATGAAATATGAAATATGAAATTAAGTAAATATGAAATTAAGTATAAGT

ATAAGTAAATGTAAATTAAGTTAAGTTAAGTAAAGTAAAAGTATAAGTAGAAGAACCCAG

ATTCCAAATGAACAAATTCAAACTCGAAAAGGATTTTTCTGATTCTCGAAGAATGAGGGG

CAAAGGGATTGATTGAGAAAGATCTCTTGTTCTTAAGGATCCCTTGTTCTTATTATAAGA

TCGTGATCTGATCCGCCTATTTTGGTAAAAAGAAGAATTCTTTGATCATAATCCAAAAAA

AATGTATGGAAAGTGTTCAATTGGAACATGAAAACGCGACTGAATTGGTCCTAGTTACTC

TTCGGGACGGAGTGGAAGAAGGGAGGAGATTCTCGAACGAGGAAAAGGATCCCATTATTT

CGAAAGAATTGAACGAGGAGCCGTATGAGGTGAAAATCTCATGTACGGTTCTGTAGAGTG

ACAGTAAGGGTGACTTATCTGTCAACTTTTCCACTATCACCCCCAAAAAACCAAACTCTG

CCTTACGTAAAGTTGCCAGAGTACGATTAACCTCTGGATTTGAAATCACTGCTTATATAC

CTGGAATTGGCCATAATTTACAAGAACATTCTGTAGTCTTAGTAAGAGGAGGAAGGGTTA

AGGATTTACCCGGTGTGAGATATCACATTGTTCGGGGAACCCTAGATGCTGTCGGAGTAA

AGGATCGTCAACAAGGGCGTTCTAGTGCGTTGTAGATTCTTATCCAAGACTTGTATCATT

TGATGATGCCATGTGAATCGCTAGAAACATGTGAAGTGTATGGCTAACCCAATCACGAAA

GTTTCGTAAGGGGACTGGAGCAGGCTACCATGAGACAAAAGATCTTCTTTCTAAAGAGAT

TCGATTCGGAACTATTATATGTCCAAGGTCCAGTATTGAAATCATTTCAGAGGTTTTCCC

TTACTTTGTCCGTGTCAAGAAACAATTCGAAATACCTCGACTTTTTTAGAACAGGTCCGA

GTCAAATAGCAATGATTCGAAGCACTTCTTTTTCCACTATTTCGGAAACCCAAGGACTCG

ATTGTATGGATATGTAAAATGCAGGATTTCCAATCCTAGTAGGAAAGGGAGGGAAACGGA

TACTCAATTTGAAGTGAGTAAACAGAATTCCATACTCGATCTCATAGATACATATAGAAT

TCTGTGGAAAGCCGTATTCGATGAAAGTCGTATGTACGGCTTGGAGGGAGATCTTTCATA

TCTTTCGAGATCCACCCTACAATATGGGGTCAAAAAGCCAAAATAAGTGATTCGTTTTTA

GCCCTTATAAAAAGAAAACCGATTCTTGAATCCCTTTCACGCTCATGTCACGTCGAGGTA

CTACAGAAGAAAAAACTGCAAAATCCGATCCAATTTATCGTAATCGATTAGTTAACATGT

TGGTTAACCGTATTCTGAAACACGGAAAAAAATCATTGGCTTATCAAATTATCTATCGAG

CCGTGAAAAAAATTCAACAAAAGACAGAAACAAATCCACTATCTGTTTTACGTCAAGCAA

TACATGGAGTAACTCCCGATATAGCAGTAAAAGCAAGACGTGTAGGCGGATCGACTCATC

AAGTTCCCATTGAAATAGGATCTACACAAGGAAAAGCACTTGCCATTCGTTGGTTATTAT

GGGCATCCCGAAAGCGTCCGGGTCGAAATATGGCTTTCAAATTAAGTTCCGAATTAGTGG

ATGCTGCCAAGGGGAGTGGCGATGCCATACGCAAAAAGGAAGAGACTCATAGAATGGCAG

AGGCAAATAGAGCTTTTGCACATTTTCGTTAATCCATGAACAGGATCTATATAGACACAT

AGATCCATGGATCCATACATCTCGATCGGAAAAGAATCAATAGAAAAAGAAAGAATCGGA

ATTGATCGATATCTTTCTCGAAACAAACGAAAAGGAAAGATGAAAGATAAATCATGGATC

AACTAAGTGCTCTCGGGGGCTTGCTTAATTAAGAATAAGAAAGAGGAATCTCATGGAAAT

ACCATGGAATAAGGTTTGATCCTATTCATGGGGATTCCGTAAATATCCCATTCCAAAAAT

CGAAAGTTCGAAACAATTGGACTTTTTCGGGGATTGGATGCAGTTACTAATTCATGATCT

GGCATGTACAGAATGAAAACTTCATTCTCGATTCTACGAGAATTTTTATGAAAGCGTTTC

ATTTGCTTCTATTCCATGGAAGTTTCATTTTCCCAGAATGTATCCTCATTTTTGGCCTAA

TTCTTCTTCTGATGATCGATTCAACCTCTGATCAAAAAGATATACCTTGGTTATATTTCA

TCTCTTCGACAAGTTTAGTAATGAGCATAACGGCCCTATTGTTCCGATGGAGAGAAGAAC

CTATGATTAGCTTTTCGGGAAATTTCCAAACGAACAATTTCAACGAAATCTTTCAATTTC

TTATTTTACTATGTTCAACTCTATGTATTCCTCTATCCGTAGAGTACATTGAATGTACAG

AAATGGCTATAACAGAGTTTCTGTTATTCGTATTAACAGCCACTCTAGGAGGAATGTTTT

TATGTGGTGCTAACGATTTAATAACTATCTTTGTAGCTCCAGAATGTTTCAGTTTATGCT

CCTACCTATTATCTGGATATACCAAGAGAGATGTACGGTCTAATGAGGCTACTACGAAAT

ATTTACTCATGGGTGGGGCAAGCTCTTCTATTCTGGTTCATGGTTTCTCTTGGCTATATG

GTTCATCCGGGGGAGAGATCGAGCTTCAAGAAATAGTGAATGGTCTTATCAATACACAAA

TGTACAACTCCCCAGGAATTTCAATTGCGCTTATATTCATCACTGTAGGAATTGGGTTCA

AGCTTTCCCCAGCCCCTTTTCATCAATGGACTCCTGACGTATACGAAGGAGTGCGGTTCG

TTCGACAAATTCCTACCTCTATATCTATCTCCGAGATGTTTGGATTTTTCAAAACTCCAT

GGACATGCAGAAGAGAAATGCTATCCCCACTCGGACTAAGACATCACTTTGACTAAAAGT

TTATTGTGATCCTTTTGTTCAAATAACAATTAAGGTGAAGCAGGGTCAGGAACAACGAAT

CTCTTTATGATAAACAGATCCATTTTGCAAGTTCGTTATTACGGGTAGTTCCTACAAAGG

ATCGGGCTAATGACGTATACAATACTTGAATTCTCGATGTAGATGCTACATCGTTGGTTC

TCATCCTTCAGAGACTACGAGTGTAATAGGAGCATCCGTCGACAAAAGGATCACCCTAAG

ATGATTATCTCATGGCTATTGAGAACGAATCAAATCAGACGGTTCTATTTCTCAATCTTT

CTGACCTGCTCCTACAGAACCAAGGACGAAAAGATTGAAAAAGTCGGTCATTCACAACCA

CTGATGAAGGATTCCTCGAAAAGTTAAGGATTAGTAATCCTTTTTAGAAATCGAATGGAT

TCGGTCTTATACATACGCGAGGAAGGTAATCAAAAAAGAAAGAAGATGAGTTCTTCTTTC

TTTTATCACTTAGGAGCCGTGCGAGATGAAAGTCTCATGCACGGTTTTGAATGAGAGAAA

GAAGTGAGGAATCCTCTTTTCGACTCTGACTCTCCCACTCCAGTCGTTGCTTTTCTTTCT

GTTACTTCGAAAGTAGCTGCCTCAGCTTTAGCCACTCGAATTTTCGATATTCCTTTTTAT

TTCTCATCAAACGAATGGCATCTTCTTCTGGAAATCCTAGCTATTCTTAGCATGATATTG

GGGAATCTCATTGCTATTACTCAAACAAGCATGAAACGTATGCTTGCATATTCGTCCATA

GGTCAAATTGGATATGTAATTATTGGAATAATTGTTGGAGACTCAAATGATGGATATGCA

AGCATGATAACTTATATGCTGTTCTATATCTCCATGAATCTAGGCACTTTTGCTTGCATT

GTCTTATTTGGTCTACGTACCGGAACTGATAACATTCGAGATTATGCAGGATTATACACA

AAAGATCCTTTTTTGGCTCTCTCTTTAGCCCTATGTCTCTTATCCCTAGGAGGTCTTCCT

CCACTAGCAGGTTTTTTCGGAAAACTCTATCTATTCTGGTGTGGGTGGCAGGCAGGCCTA

TATTTCTTGGTTTCAATAGGACTCCTTATGAGCGTTGTTTCTATCTACTATTATCTAAAA

ATAATCAAGTTATTAATGACTGGACGAAACCAAGAAATAACCCCTCACGTGCGAAATTAT

AGAAGATACCCTTTAAGATCAAACAATTCCATCGAATTGAGTATGATTCTATGTGTGATA

GCATCTACTATACCAGGAATATCAATGAACCCCATTATTGCAATTGCTCAGGATACCCTC

TTTTAGCTTCTAGGGTCTATTTCTTAGTTCAAGATCCCTCTTACTAACTGGAATAAAAGA

ATTAGTCGATCTATTCCGCCCAAAATGGGAATGGTCCGGTATGGAATGAACTTATAATCG

GATGATCGAGTCGATTCCATGATTATAAGTTCATTCCATACCGGACCAGGCCGGAATAGG

GTTATGTCCATTCTCATTATGAGAAGGAGTCATTAGAAGAGCGTATAGAAATAGATACTA

TGTTTCCATATGGATCCCTACGTCGTCACATTCCATTTAGGATTAGGAATAGGAGTAATC

GGACCTGACCTGCTTTTTACATATCTATTCTATCGTTATTTGGGTACCATATGAACTCGA

TTGAATCGAGAAATAGGTTTGATTGTATATCTTTTTGATATATATATAAAGTATCCTCCG

GATAATTCAAATCGAAGCAATTTGCTGTCCGATCGGGCCTATATATATGGCACGGCCGAT

CGATAGAAATACCCCAACACTTCACCTTTGCCATATATTCCATATATCACACTAGATAGA

TATCATATTGATGGAATACGATGCACTTTCAAAATGCCTTGATGGTGAAATGGTAGACAC

GCGAGACTCAAAATCTCGTGCTAAAGAGCGTGGAGGTTCGAGTCCTCTTCAAGGCATAAT

ATTGAAAATGCTCATTGAATGAGCAATTCAATAACAGATCTCGGATCTAATCGATATTGA

TATACCGAGCCTCTGTTGATACGAAGTCTTCCGGCGATCCCCAGATCCGAGTCTGGGCTG

TTGGAATAGGTTCGACAGCGGATCACGAAATCTTGGCGATCTTCTCTATCTAATGAATGA

GGAGTCCGCTTTGAAATCGTCCGCCCTGCACCCACCCCGCGAGTATATGCTTCAACAGGT

AGATTGATACAATAGAAACCTCTGGTAAAATGCCCGCCCGTCGCCCAGCAGATAAAGTAC

ATTACATAGTCCGTTTTAGGCATTGGCGACTTCCCCATTCAGCGACTTTGGCACTGGACG

TACCCAAAATGGGTACTATCGGGTCGGGTGAATTAGATAATAGACGTCTGTTGGCATTCG

AGCCTTCCCTCTCCTTTCAGGGCCTATCCGAAACAGAATCCAGTACCTCTCGGTCGTGAA

TATCTGAATAGGACGAACCGGCCCCCGTGAATATCTTTGCTTCGGAACAATTCGAATTAG

GCTCGGACAACTGGAATGTGTATTCTCCATATAGGAGATCTTCCAATTGAGAAGATCCAT

CGACCTGAGACGAAGAGAAAGGCCTATCTATTTTATTTAGTTATTCAGTTCAACCAATGA

TTCGTTATTGGAGCAGACAGCAACAACCTTTTCATCGGACATGCGTCTTTTTTTTTGATT

TTCCAATGGATTTACATGTTTCATTAATGGAAATTGTTTGAAGTAGTGAGTAATAAATAG

GCTCTTCTGGTTGTTCGCCGTTCAAGAATTCTTGTTTAGGCAGTTCATACCACCTATCCA

TAGTGTTTTGATCTAAGATTTCAATTCTTCCATGTTTCAGTAGTAGCATATCGTTCCATG

GAGTTAAGGTCCAAAATATGGAAGAAACAAGTGTTTCCACGACCCGGTCAATTCTGTTCC

GCTTAATCCCTCTTTCATGGCTACATAGCCTCCGGTTAAGGAATGGGAAATCTTTCTCCT

GTTACATGAATCCAATGTTTCATTTCATCCGGGAAAAGCCATCTCTTTCTCAACAACGCC

TTTGTCATTTGATCCAATAGCGTTCTGTTAGATAGGAACAGATTTGATAAATACTGATAA

CTCTCGGATAGAGTATTAGAACGGAAAGATCCATTCGATAATAAACTATTGGTTCTAAGC

CATCTCTGGCGATGAATCAACAATTCGAAGTGGTTTTGTTGCGTATTCTTGATGAACCAG

CGTTTATATATAGATGTAGTAGGATTTGTTTGGGAAGTAATAAGCCCCTTTGACATCTCT

TCATCTGCAAAGAATTCTCGACGTGAAAAGACAAAGGGCTGATCTTTGAATAGGAAAAAG

AATGGATCTGCAGGGTCCCAAATGAATTGGCTTATTCGAAAAAAGCCTTGTTCTTTGGAA

GATCTATCTAGTCTCTGGTACCGCACGGTTCCACTCTGAAAGAACTCCGAATCATTCTCT

TGAAGCGCATCCTCTTTATGATAAAGGATCCGCTTGCCCCGAAATGACCTGGTCCACCAA

TAGGGAAATCCCAATTCATTGGGTCTTTCGATACAATCAAATAGATTGCCACAAGGGCGC

CATATTCTAGGAGCCCAAACTATGTGATTGAATAAATCCTCCTCTATCTGTTGCGGGTCG

AGGGCTCCTTCTCCTTCCCCTTCTTCCAACTCCGATTCGTATTTTTCATATAGAAATCTC

TGATCAACGATAGAACAAGATCCATTTTGTATCATATCGAACGGATTCTTTGGTTCGGAC

CGAAGAAGCAATATCACTCGATCATTATCAAACTGACTGCAATCTTTTTCTGTCCGTGAG

GATCCCACCAAAGCGCCTTCTACTTCTAATAGGCCATGAACTAGATCAGAATCATTCTCA

ACGAATCCATAAGAAGTGATCCAATTTCTTTCATCGGGTCCGGGTGGAGACCAAAGATCT

TGAGCGACCGATCCGGCAGAACAACTCAAAAGATAAAGAAGTATCGTTAATTTCTTCATG

CTCGTTCCAAGCTCGAAGTACCATTTGTACAAATAAGAATCCCCTTCCTTACATGATTTC

TTCTTCATATAGATAGATATAGGATCTATGGGGCAATCACTTAGAAGTACATTTTGTGCA

ACAGCCCTTCCTATCTGATAGAAAAGGATCCCATGATCCTGAACCCATCTCACCTGGGAT

CGCAAATCCCAAGTTTGTCTATGAATAGCTGATCGAATTGTATTAGTGTCTATAATTGAT

TTCTTCTGTGTAATACTAATTGATAGAGCCTCATTGGTAAGTGCTACAAGATCTCGTGCA

TTGGAACCCATGGTTATGGACCCGAATACGTTAGTATGGAACATTTTCTTTTCCAAGTGA

AATCCCCTAGTATATGAAAGAATGAAAAAGTGCTTTCGTTGTTGTGGAATAAGAAGCCTT

CGTATCTTAATGCATGTATTTAATTTATTCGGAGCTATTAGAGCGGGATCCACTTTTTGG

GGAATATGAGTCGAAGCAATAAGAAGAATATTTCTAGTGGAACATCTTTCACAATCTCTG

GAGAGATAGTTCACTAATAGACCGAGGGATAAGTAATTCGACTCATTCACATACAGATCA

TGAATGTTTGGAATCCATATGATGCAAGGAGACATTGCTTTTGCTAATTCGAATTGAAGG

GTGATATCAAATCGGTCTATTTTCGGCGTCATATACATAGTTAGCACATTCGTCATAGTT

AGCAGCTCCGTATCAAGGTCATCAATATTGTCACTATCATCAATATCGATATCGTCACTA

TCATCAATATCGATATCGTCACTATCATCAATATCGATATTATCAAAAAGATAACCTTTA

GGCTTGTCATCCAGGAACTTGTTCGGAAATACCGTAATGAAAGGAACATAGGAGTTTGTC

GCTAGGTATTTGACCAAATATGACCGTCCAGTTCCTATAGAACCTATCACTAAAATACCC

CTAGAAGGGGATAGGGCTAAGCGGAGCGAAAAGGGTTTTCCATGAGATGGGAAATGAGAA

CTATTAGTCCCACACGAGGTTTGTGAATAAGTGATTGTCTGATAATGAGCAAGGAATATC

CGTCTTTCTGCTAAACAGGATCTATTGAACTCATAATTCATTAGATACTTTTTATGAATG

TCAACTAAGTATCGTAAGTAAATTGATCCCGGTTGTTCAATCATTTGATAACCAGAGTCA

TTCTTTGATAAATGATCACTATGAGTCAGACTCAATAGAATTTGATCAATCCTTTTTTCT

GTCGTTAAGGTGGAGAACTGAACCAAGAATTCTCTTTCTTCATCATCAATCGAATCACTG

TTCGCGACCCAGGATTCGATTTTTTCATCAATCCAACCACCGTTCACATTTTTTTTTCTT

TTTCTTATCAATGAATAGATCTCTTTACTTGTATGACTTAGATGTCTCGTATTTCTCGAA

AAAGTGATTCGATTGATGGGATTTGGTATGATACTTATGAAATCAATGATATCGATGAGA

TTGATATGAAAATATTTCTTCTTAGAACGTATTGATTTGACCCCATAAGCGGGACCACCA

CCCAATAGTATGTTGCCGCCAGAAGCAGAACCGCGTATTTCTTCCAGAGAATCTCCTAAT

TGTTCCAGAGCAACTAGAAAGAGATTCTTTAACCAGAAAGAATTCAGTTCAGGTGTAGGA

TACCTATCCAGAAGTTTTCGCAACTCCATCATGTATGATGGAATCATTAAAGATTTGATC

TTTTCTAACTCTGTCTGTAACTCACTAGAGGCTCGGGAAACAAAGAGAAGATGTGTACGA

ACGAGATATCCAGCAACAAGAAGAAGGAAAAGGATTGAATAGAGTAACTCCCGAACATTT

GGTGATCTCAGATGTGTCCATATTAATAGAGCGGGTGACTCATTATTTCGATGAATGATT

TCTTCGTACAGAAGAAGATTCTGTAAACACTTACTCGACATCTCACTTATCCAATTCCTT

TGTGGAAGAATCGCCCGCCCTTTTTGCTGAGGAATTGGCCATGATATATCTGATCCATGC

ATAATATCATGAAAAATGGATACAAATTTTGGACTGTTACTTAGTATCGGCAATGGGTCT

GAAAAAGTATCTAAAAGGGTGAAATTTAGATATTTGCACCCTGTCGAAGTAAGGAACCAT

GGCATATATGTTTGGAACAGATTCCATTTTGAGAGATTTGAAAAATCATTATCTCGTTGA

AAGGTTCTATACATCTGCCATTTCTCAACGCATTTCTTTAGACAAAGAGTCCGTTTTTTC

CTCTTTCCGGATGGGAAATCTTTCTCAGAACATAGAGTGTGAATCAAACCCATGTTTGAA

TTGAAACGGAGATACTGATGCAAGTTCTTCCCTTCTGAATCAGATAGATTCATATCTGAA

AGAGGCTGACAATAAGTTCTTTGAAAATTGACTATTTGTTCCTCTGTTAGAGGTGTTCCA

GAAATGTCTGCAATCGAGTAAATAGCTCTACGAACGAATGAATCGGATCGAATTGGAAAA

TGGAAAGATTTGTACAAGTTATACGTTTCGTCACCACTTTGTGGAAAATCGTTAGGTATG

AATATGTCAGATACCTGTGACTCGATTGGTGAAATAGCATCTCTCCCCCCAAAAACATGT

TTTTTTTTACTGACGCACAAAGAAAATATTTTGTTGCGAATGAACAAGACATTGAGGAAT

TGTCCATATGTAAGATCATAATTATTGATACGGGCCTTTTCCACATCAAAAGGGAATCTT

TTGTTACAATAGAACCATAAGTGATGCGGATTATTCAAGAATCGAAGTCGATTTGCTTTA

TAAAAAGAAGATATCGATGAACTTCTATGAAATGGTTTCACGGGATTCAGCCAATTGTCT

CGATCGTGGGATATCATTGAGAAATAGGAATCCGTGTTATCAAAGGATTTCCTGCGATTC

TTTCTAGTATGGAATGAGTCAATCATCCACTTTGGTATCTTATTGAACAAAAAGGGTGAT

ATTGTTCCTCCATTGATCAAGAATTTCGATCTTTGGGAAGTATCATGATCATCCAATAAA

AAGGGTTTCAATTTATTCAAATGAACGATTTGAACACCTATTGATTCTAACAACGGATTG

CAGAGTTGATCATTCGGACCTTTCAATTCATAGATGTGGATCTCGAACCTATGAATGGGG

ATATTCCCGATACTCACAAAGAAAAAAGGGAGTGACTTAGACAAAAAGAAACGAAGTGAC

TTAGACAAAAAGAGAAGTGACTTGGACAAAAAGAAACGAAGTGACTTAGACAAATCTTGT

TTGTCGATAACCTCGGACCAATCAATCGAATATTGATTAATACGTAATCGACCGAACACT

ACTTGAAAACGGCGCTTCTGTTCAGAAACGAAATGTTCCAAATGTTCCTGGAAATTCTTG

CTCCCATTGGACCATTTGTATCTATATGCATCAGGATCCCGATTCATGCATCTCCCGGTT

CGAGAAATAAGAGGATCGAACCATTTCTTCTGACTCTTTTTCAAATTCGATAAATGTTGG

TTGATCGTATATTTGATTATAGTTAGATGATTCAGAGTATCATTTCCTATTGGATCCCCT

TGAATTCCATATTCGAAGTTGCGATCGGGTCGATTCATTAAAAAGAATCGATTCGATACA

TTTCTTATGTACTCATAGGTGCGATATTGGATTTGAATCAGATTTCGGATCAATCTATAT

TGATTGACCGCCCCCATTATGTTGTTGCTAGCAAATACCACTATTTTTGGGTTTGGATCT

TCCAAATCATTGCCGCAGGAGATCCGGGCCGATTTTTTTCTGATCCTTCCGTAAAAAGAT

TCATTCTCTTCATCAAAAATAGGAGGTAGAAACCATAAAGATTTCTTTTTCGATTCATCC

CTGGAGTTGAATACCTCATTCAAGAATTTTTTTTGATCCAATCCGTAGGAATCAATAGAA

AAGGTAAATCCCTTATGATATACCAGATCCGCCTCGGTTATTGATAGAGTGAATAGATCT

GCCATTTCTTGAAATCTCTCTTCTGATTCAAAATCGTGGTGTAACGTGTATCCCCCTTTG

TTCCGGTCATGGAATAGATGAAATAAATCCAAAAATGGATTTTTATTCAAGAATGAAATC

TTATTGGAACTGTCTATATCTGGTTCATCCTTCGGAACCATACCACATCCCGGATCTGAT

GAAATAGGATGAATTGAGACGGTATTTTGTAAATACGTAATTATCTTGAATATAGCAACC

ATTTCTTTATTTTCCGATCGCCTGGAAGGGACAAAAGAAACATCTTGTTGTTTCTTCAAC

AATTTCTGATCTCTAGTGGACCTCTCAGTAGGATTCGAGCCCAGACGAAGTTCTGACCAT

CTGTCAGAGAAAAAAGAACGAATTGATCTTGTAGGATTCCCAAGAAATTCTTCGATTTCT

TCCGGAAGCAGATGATTATTCATCTGCTTCTCACGTTCCGTGAATAGCCGGGATATTGAG

GAATATCCAGAAAGGCATTTCGGGAATCGATCTGATTCTATCTCTGTTCGTTCCGTTTGA

AGAAAGGAAGGATCCCAAAGAATCGATCTTTCTTTTAGTTGCTGAATCTCTTTGATCAAT

GTGTGATATTCCGAATTCTCATTCCTAATGGAATCGAAATGATCTCCGGATTGATCAGAA

GATCCTTTCAATTGGCTAGAATCCGTTACTTGAACGAAAATAGATCTTGTGGAATCATAT

TGAATATTTGACGATACATTGCGTACCTTGCTAAAAAACCGATCCTTGTTTACCAACCAC

ACATTGTCTAACCAAATCCAATTATCCCTTGATACGTTCCTCAAAAAATCCGACTCGTGC

TGATTCTTTCCCCAACTAACGAAGAGATCTTGGTAGAATTGCCACATATGAAATTGAGCA

CAATTTTGCAAAAAAATACCCCACTTCTTTCTCGAGAAGAGATGGGAAACATGCTCAATA

TCCTTTGATTGAATAGTTGCCCCAGCTCCTCCTTGTTGTTTGAAGAACCCCCCCATTTCA

ATTGGTCTTTTTTCACGAAAAGCAGACATGAGATAACAAATCAAGTGTTTCACTAAGATT

TCGAATAGCTGTCCCGAATTCAAGTTGATTATGTTTCGCTTCTTCCTCGGAGAAAGACGA

TCAAAGAATTCCCAATCATGGTCCTTGCGGATCGGATCATCCGTATAGGATACAAAAAGA

AACTTCAGATATTGGATATCTTTCTCTTTGAATGAGATCTCAATTCCAGCTATGGTTTCA

TTAGATATCTTACAACTAGAATCCCTCTTTTTTCCGATCCGGTTCCTCCACCACCGAGAA

CCCCAGTTAGATTCAGGCATGATACACTTTTTTTTAGTTATTGGGAGAACCCAAGCACTC

TCTTTCGAATCCATGAAACAACTCTCAGAGATCTTTTTCCCTTTTGGAAGATACAGGAGC

GAAACAATCAACCTATTGATATTGGAAGACCAAAAAGATTCTTCCAATGTATCATTTCTG

GGTCCAATCGAATTCATAGGTATAGGAAGAATAGGTATAGGAAGAAGCCCCCTCAAATAG

AGATTTTTTCTTTCGACTATATTTCGATTGTTAATACGATATATAAGGACCGCTACTACA

AGCAGTACTACACCTTTGACCGTAAAATATCGATTGCTTGTTGAACCCTGTGAATCGCGT

GAAAGTAGGATACTCCAAATTCGGGGGTCAAAGAGTTTCATAAAACGTTCTTGGTGGAAA

AAAATGTGAGTGAAAGATCCCACTGAATCGAATTGCGTCCATGAATCTAAGAAATAGTGA

GAATTCTTGACCTCTCTCAATATCTCTCTCAATTCGAAGATCCAGGATTGGAATTGATGT

CCTTTCATTGATTCCTCCTAAAGATTTCATTTCAATTGGAATTTGGTTATTTACGATGAT

CCCTGTTAAGCATCCATGGCTGAATGGTTAAAGCGCCCAACTCATAATTGGCAAATTCGT

AGGTTCAATTCCTGCTGGATGCACGCCAATGGGAACGTTCAACAAGTCTAGTGGAATTGG

CTCTGTATCAATGGAATCTCATCATCCATACATAACGAATTGGTATGGTATATTCATACC

ATAACATATGAAGAGTAAGAACTAGAATTCTTATCGATACTGGAACTTATAGGGAATAAA

ATGGATTTATGGATGGAATCAAATATATAGTCTTTACAGAAAAAAGTATTCGGTTATTGG

GGAACAATCAATATACTTCTAATGTCGAATCAGGATCAACTAGGACAGAAATCAAGCATT

GGGTCGAACTCTTCTTTGGTGTCAAGGTAATAGCTATGAATAGTCATCGACTCCAGGGAA

AGGGTAGAAGAATGGGACCTATTATAGGACATACAATGCATTACAGACGTATGATCATTA

CGCTTCAACCGGGTTATTCTATTCCACCTCTTATAGAGAAAAGAACTTAAATGAAAATAC

TTAATAACACGGCGATCAATTTATACAAAACTTCTATCCCGAGCACACGCAAAGGAGCCG

TAGACAGTCAAGTGAAATCCAATCCACGAAATAATTTGATCTATGGACAGCATCGTTGTG

GTAAAGGTCGTAATGCCAGAGGAATCATTACCGCAGGGCATAGAGGGGGAGGTCATAAGC

GTCTATATCGTAAAATCGATTTTCGACGGAATGACAAAGACATATCTGGTAGAATCGTAA

CCATAGAATACGACCCTAATCGAAATGCATACATTTGTCTCATACACTATGGGGATGGTG

AGAAGAGATATATTTTACATCCCAGAGGGGCTATAATTGGAGATACCATTGTTTCTGGTA

CAGAAGTTCCTATATCAATGGGAAATGCCCTACCTTTGAGTGCGGTTTGAACTATTGATT

TACGTAATTGGAAGTAACCAATTAGGTTTACGACGAAACCTAGAAATCGATCACTGATCC

AATTTGAGTACCTCTACGGGATAGACCTCAACAGAAAACTGAAGAGTAACGGCAGCAAGT

GATTGAGTTCAGTAGTTCCTCATATCAAATTATTGACTCTAGAGATATGGTAATATGGAG

AAGACAAAATTGTTTGAAGCACGGACAGAGCCGGAAGCGCCCCTTGTTTCAAAGAGAGGA

GGACGAGTTATTCACATTTCATTTGATGGTCAGAGGCGAATTGAAAGCTAAGCAGTGGTA

ATTCTAAAGATCCCCCGGGGGAAAAATAGAGATGTCTCCTACGTTACCCGTAATATGTGG

AAGTATCGACGTAATTTCATAGAGTCATTCGGTCTGAATGCTACATGAAGAACATAAGCC

AGATGACGGAACGGGGAGACCTAGGATGTATAAGATCCTAACATGAGCGATTCAGCAGAT

TGGAATTCCTCTATATCCACTCGTGTGGTACTTCATCATACGATTCATATAAGATCCATC

TGTCTAGATATCATCATATACATCCGGAAAGCCGTATGCTTTGGAAGAAGCTTGTACGGT

TTGGGAAGGGGTTTTTATTGATCAAAAAGAAGAATCTACTTCAACCGATATGCCCTTAGG

CACGGCCATACATAACATAGAAATCACACTTGGAAAGGGTGGACAATTAGCTAGAGCTGC

GGGGGCTGTATCGAAACTGATTGCAAAAGAGGGTAAATCAGCTACATTAAGATTACCATC

TGGGGAGGTCCGTTTGATATCCAAAAACTGCTCAGCAACAGTCGGACAAGTGGGTAATGT

TGAGGTGAACCAGAAAAGTTTGGGTAGAGCCGGTTCTAAGTGTTGGCTAGGTAAGCGTCC

TGTAGTAAGAGGAGTAGTTATGAACCCTGTAGACCATCCCCATGGGGGTGGGGAAGGAAG

GGCCCCAATTGGTAGAAAAAAACCCGCAACCCCCTGGGGTTATCCTGCGCTTGGAAGAAG

AAGTAGGAAAAGGAATAAATATAGTGATCGTTTTATTCTTCGCCGCCGTAAATAGGAATT

TGGATATGGAAAAGAAAATCGATAAGAATCCATTCGATTATGTTTCGTTCGTTTCTTCGT

TTCGTATTTACAAATCTTCCTTCCGAATTACAAAACCAAAACAAATTTTTTTGGGAATAA

TTT
